# Property-Biased Covalent DNA-Encoded Library Screening Enabled the Discovery of AM-8719, A Structurally Novel, CNS-Penetrant KRAS G12C Inhibitor

**DOI:** 10.1021/acs.jmedchem.6c01357

**Published:** 2026-07-29

**Authors:** Slavko Rast, Marie Morgan-Fisher, Sarah D. Blomquist, Jorge Peiró Cadahía, Sanne Cowland, Thomas Franch, Emil Glibstrup, Alex Gouliaev, Margit Haahr Hansen, Aleksejs Kontijevskis, Titi Kronborg, Loris Moretti, Anna Nadali, Søren Nielsen, Sebastian Leth-Petersen, Michael Rabe, Adili Alafate, Jennifer R. Allen, Abhisek Banerjee, Shon K. Booker, John R. Butler, Imelda Hot, David Huang, Matthew R. Kaller, Rajiv Kapoor, Qingyian Liu, Patricia Lopez, Vu Ma, Francesco Manoni, Jose M. Medina, Alexander J. Pickrell, Hui-Ling Wang, Jingjing Xie, Wenhan Zhang, Christopher Mohr, Kui Chen, Anne Y. Saiki, Paul Wang, Monica Leavitt, Karen Rex, Guo Zhong, Ling Zou, Julie Lade, Upendra P. Dahal, Nashid Farhan, Prashant Agarwal, Borna Zandkarimi, Kai Zhu, Gitte Husemoen, Nuria A. Tamayo, Brian A. Lanman

**Affiliations:** † Amgen Research, One Amgen Center Drive, Thousand Oaks, CA 91320, United States; ‡ Amgen Research, Rønnegade 8, Copenhagen 2100, Denmark; § Amgen Research, 750 Gateway Boulevard, Suite 100, South San Francisco, California 94080, United States; ∥ Syngene Amgen Research & Development Center (SARC), Biocon Park, Jigani Link Road, Bengaluru 560099, India

## Abstract

Activating mutations in the Kirsten rat sarcoma (*KRAS*) gene are prevalent oncogenic drivers in nonsmall cell lung cancer (NSCLC). Patients harboring *KRAS*-mutant lung cancers frequently develop central nervous system (CNS) metastases. Although approved KRAS G12C inhibitors (i.e., sotorasib and adagrasib) show promising clinical CNS activity, these agents demonstrate low preclinical brain-to-plasma ratios, raising the question of whether compounds with elevated preclinical *K*_p,uu,brain_ values might show enhanced clinical performance. Here, we report the first successful application of DNA-encoded library (DEL) screening technology to the identification of CNS-penetrant covalent inhibitors of KRAS G12C. In this effort, a *property-biased* covalent DEL-screening approach enabled the discovery of a structurally novel series of hydrogen bond donor-free KRAS G12C inhibitors with improved CNS exposure. Leveraging structure-based design, we refined this hit series to deliver lead compound **AM-8719**, a CNS-penetrant, orally efficacious KRAS G12C inhibitor exhibiting 200-fold improved potency with respect to initial screening hits.

## Graphical abstract

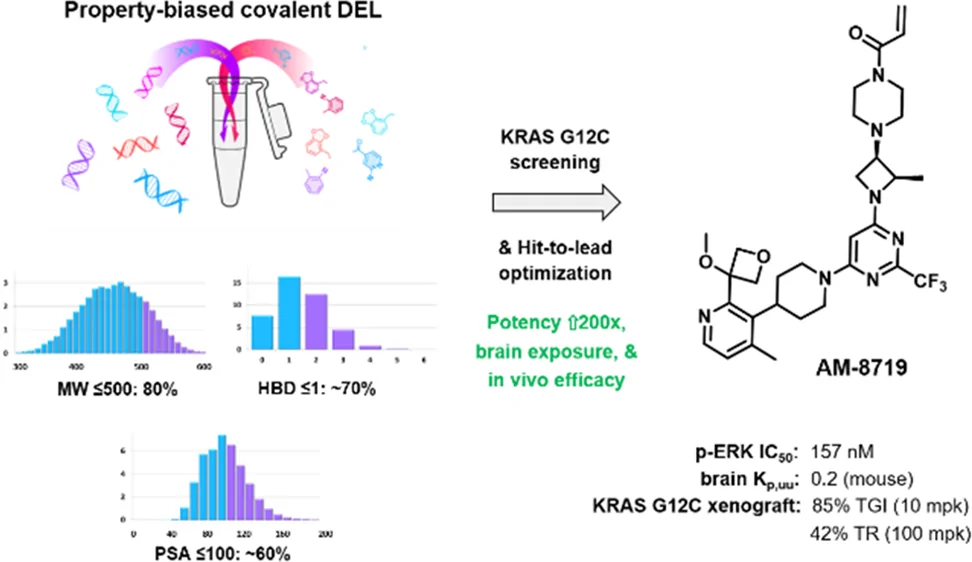

## Introduction

The RAS proteins (KRAS, HRAS, and NRAS) are small GTPases that serve as molecular switches, cycling between an inactive guanosine diphosphate (GDP)-bound form and an active guanosine-5′-triphosphate (GTP)-bound form, and play a central role in transmitting extracellular signals to diverse intracellular growth and survival pathways.^1,2^ The KRAS isoform (Kirsten rat sarcoma viral oncogene homologue) is one of the most frequently mutated human oncogenes and is a key driver in many human cancers.^3^ Despite structural and physiological factors that have historically stymied its direct inhibition, extensive efforts in recent years have resulted in clinically approved therapies for the treatment of patients with cancers harboring specific oncogenic mutations (e.g., *KRAS* G12C),^4–6^ and significant efforts are underway to develop therapies targeting other oncogenic mutants.^7–9^

KRAS functions downstream of receptor tyrosine kinases (RTKs). When RTKs are activated by growth factors such as epidermal growth factor (EGF), guanine nucleotide exchange factors (GEFs) promote the exchange of KRAS-bound GDP for GTP, activating KRAS and enabling it to interact with downstream effectors.^10,11^ Oncogenic mutations of KRAS impede the ability of GTPase-activating proteins (GAPs) [e.g., neurofibromin-1 (NF-1)] to catalyze the return of active KRAS to the inactivated state,^12,13^ leading to accumulation of the GTP-bound, active state and to an increase in pro-proliferative signaling via hyperactivation of the downstream mitogen-activated protein kinase (MAPK) pathway. This hyperactivation increases c-RAF (cellular rapidly accelerated fibrosarcoma proto-oncogene serine/threonine-protein kinase) and ERK (extracellular signal regulated kinase) phosphorylation, which, upon ERK nuclear translocation, leads to tumorigenesis and metastasis.^14^

*KRAS* mutations are an early genomic event in carcinogenesis,^15^ with the most prevalent oncogenic mutations located within the catalytic domain of KRAS (Gly12 and Gly13).^16^ Glycine-12 to cysteine mutation (*KRAS* G12C) is the most prevalent *KRAS* mutation in nonsmall cell lung cancer (NSCLC).^17–19^ *KRAS* G12C mutation predicts poor survival and significantly reduced quality of life for NSCLC patients,^20,21^ with one major complicating factor being the high frequency of brain metastases in such patients (≥40%).^22,23^

Therapeutic targeting of KRAS is a fast-growing field, with multiple companies investigating agents in different phases of clinical development.^24,25^ Although clinical KRAS G12C inhibitors have demonstrated evidence of CNS activity (e.g., control of intracranial metastases),^22,26,27^ recent studies^28^ have found such agents to show limited preclinical CNS penetration (*K*_p,uu,brain_ < 0.02), raising the question of whether agents with improved preclinical CNS penetration may lead to superior clinical CNS activity.

Low preclinical *K*_p,uu,brain_ values with current clinical KRAS G12C inhibitors could be rationalized by the suboptimal physicochemical properties of these compounds, which could restrict CNS permeability, enhance efflux, and contribute to brain-tissue binding. In 2010, Wager and colleagues introduced the CNS multiparameter optimization (MPO) model to help map the physicochemical property space associated with improved CNS penetration.^29^ This model laid out desirability functions for six key physicochemical properties [clogP, clogD, molecular weight (MW), topological polar surface area (TPSA), number of hydrogen bond donors (HBD), and p*K*_a_] and a composite MPO score in an effort to integrate desirability across these six properties into a single score.

Figure 1 illustrates how current clinical-stage KRAS G12C inhibitors^30^ map across these six parameters, with relative desirability designated as favorable (green), unfavorable (red), or intermediate (yellow). As seen in this figure, while clinical KRAS G12C inhibitors can achieve desirable TPSA and p*K*_a_ values, such inhibitors only rarely achieve favorable values with respect to lipophilicity (clogP and clogD), MW, and HBD count. As a result, the CNS MPO scores of clinical KRAS inhibitors are at best moderate (1.4–3.4), in line with the low *K*_p,uu,brain_ values observed for these compounds preclinically.

**Figure 1:**
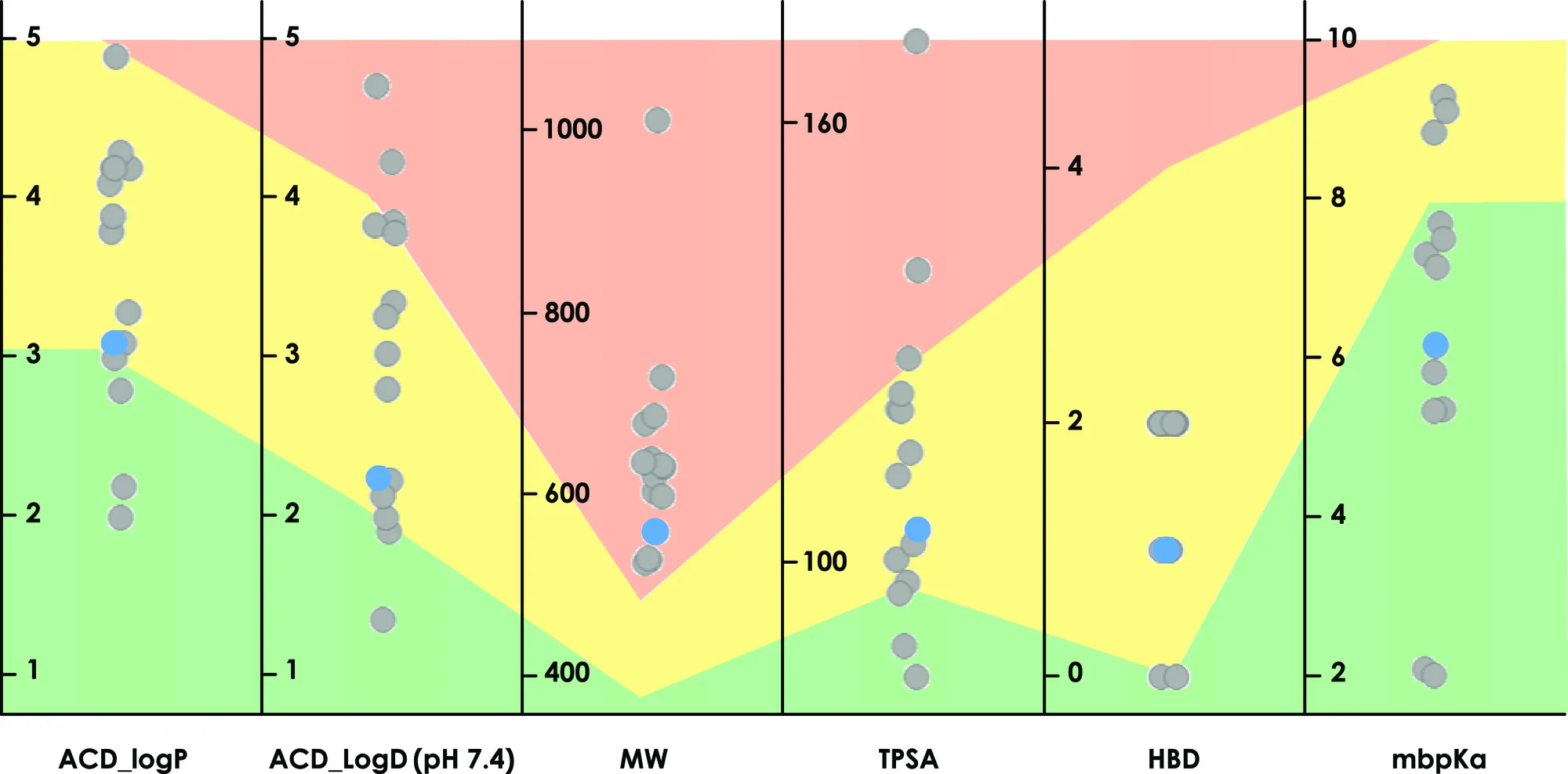
CNS MPO properties^29^ of clinical KRAS G12C inhibitors.^31^ Clinical inhibitors (gray dots, sotorasib in blue) have elevated log*P* and log*D* values, molecular weights, and hydrogen bond donor counts relative to preferred values for CNS-penetrant inhibitors (indicated by background colors; green = favorable, yellow = intermediate, and red = unfavorable), accounting for their suboptimal CNS MPO scores (1.4–3.4, see the Supporting Information).

Although the depicted property distributions likely arise from intrinsic challenges in exploiting a key allosteric binding pocket on the surface of KRAS (i.e., a shallow binding groove, necessitating higher-MW, higher-log*P*/log*D* chemical matter to drive requisite binding affinity), we hoped that through an appropriately designed hit-finding campaign we might identify new inhibitor chemotypes that could exploit this allosteric pocket while populating more favorable physicochemical property space, thereby leading to enhanced preclinical CNS penetration.

Historically, the identification of novel chemical inhibitors of KRAS has proven extremely challenging due to the picomolar affinity of KRAS for its cognate nucleotides (making orthosteric inhibition challenging) and the lack of readily druggable surface pockets (making allosteric ligand identification challenging). We recently reported success in overcoming these challenges through the use of a DNA-encoded library (DEL) to screen a 16 million-membered covalent (Cys-reactive) compound collection to identify a potent and structurally novel allosteric inhibitor of KRAS G12C.^32^

Here, we report an orthogonal DEL-screening effort that aimed to identify starting points better suited to the discovery of brain-penetrant KRAS inhibitors. Figure 2 illustrates this effort, which employed a “property-biased” library design strategy focused on the optimization of CNS MPO parameters. Whereas our prior DEL-screening strategy^32^ (“structurally biased DEL”) leveraged pharmacophoric features of past KRAS inhibitor chemotypes to guide the search for structurally novel KRAS inhibitors, in this orthogonal effort (“property-biased DEL”), we avoided pharmacophore-guided library design and instead prioritized building block (BB) selection based on physicochemical properties (as laid out by the CNS MPO model) to assemble a DEL library enriched in compounds with favorable CNS-penetrant properties (that still surveyed structurally diverse chemical space to enable the identification of novel inhibitor chemotypes). Subsequent to this work, two other groups have also reported the discovery of CNS-penetrant KRAS G12C inhibitors: AstraZeneca (AZD4747)^28^ and Genentech (compound 15),^33^ both of whom instead leveraged structure- and property-based design to optimize earlier KRAS G12C inhibitor scaffolds to identify inhibitors with significantly enhanced preclinical CNS exposure.

**Figure 2:**
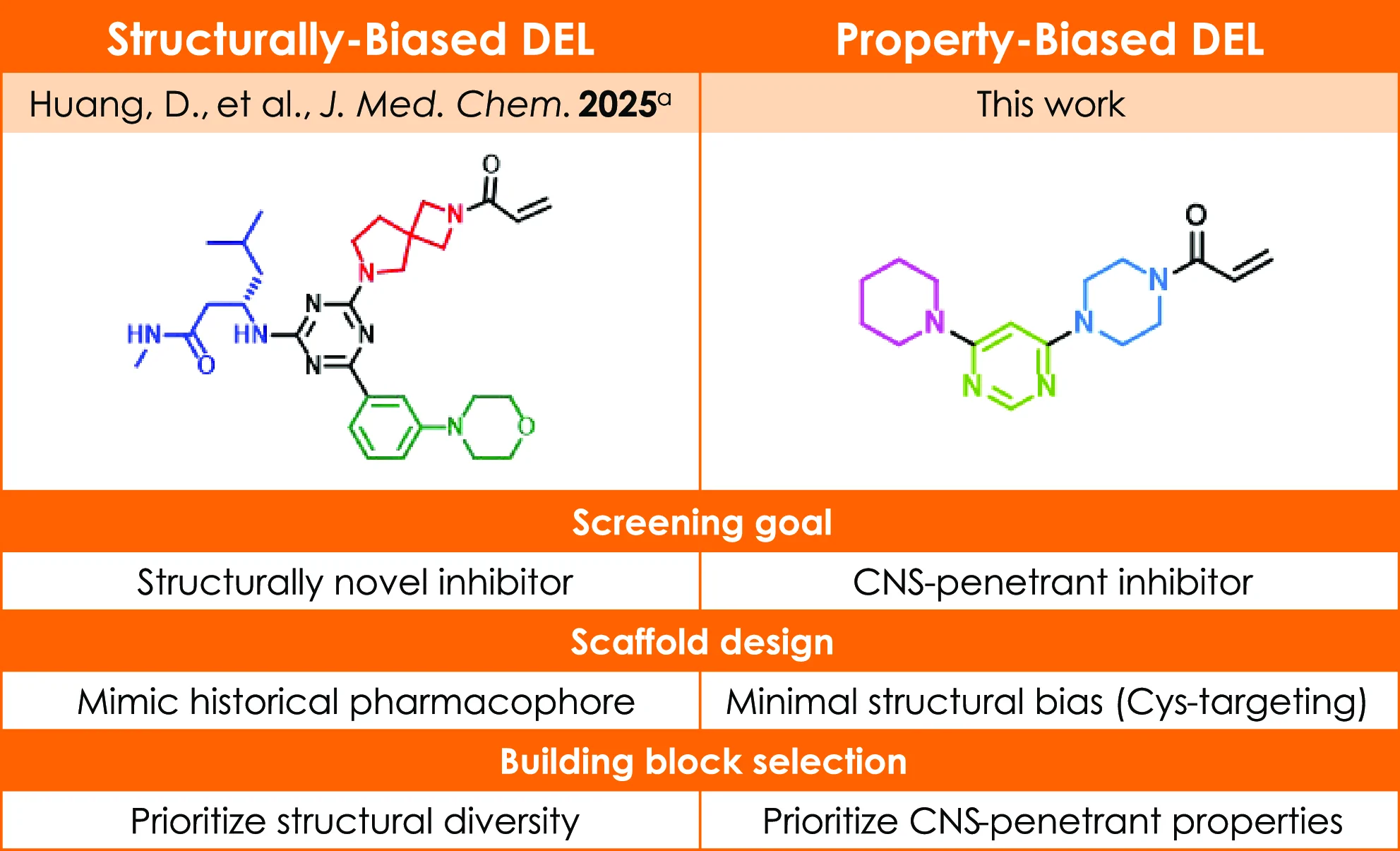
Comparison of DEL design strategies for the identification of structurally novel KRAS G12C inhibitors. Prioritizing structural diversity^a^ vs physicochemical property considerations (this work) in building block selection leads to divergent library designs, each tailored to divergent screening goals. ^*a*^See ref 32.

## Results and Discussion

### DEL Design, Screening, and Hit Validation

To implement our screening strategy, we designed a trimeric DEL^35,36^ (Figure 3A) comprising three synthetic building blocks (positions 1, 2, and 3). In position 1, we incorporated a range of *N*-protected amino acids, with the carboxylic acid group serving as the attachment point for the building block-encoding DNA tag and the protected amine serving as the synthetic handle for subsequent library elaboration. Position 2 employed diverse bis-haloarenes and monohaloarene carboxylic acids, supporting structural diversity (aminoarene or arylamide linkage between Pos 2 and Pos 1) while providing a handle for position 3 elaboration via S_N_Ar substitution. Position 3 incorporated a range of *N*-protected diamines, with the protected amino group providing a site for the final acrylamide introduction.

**Figure 3:**
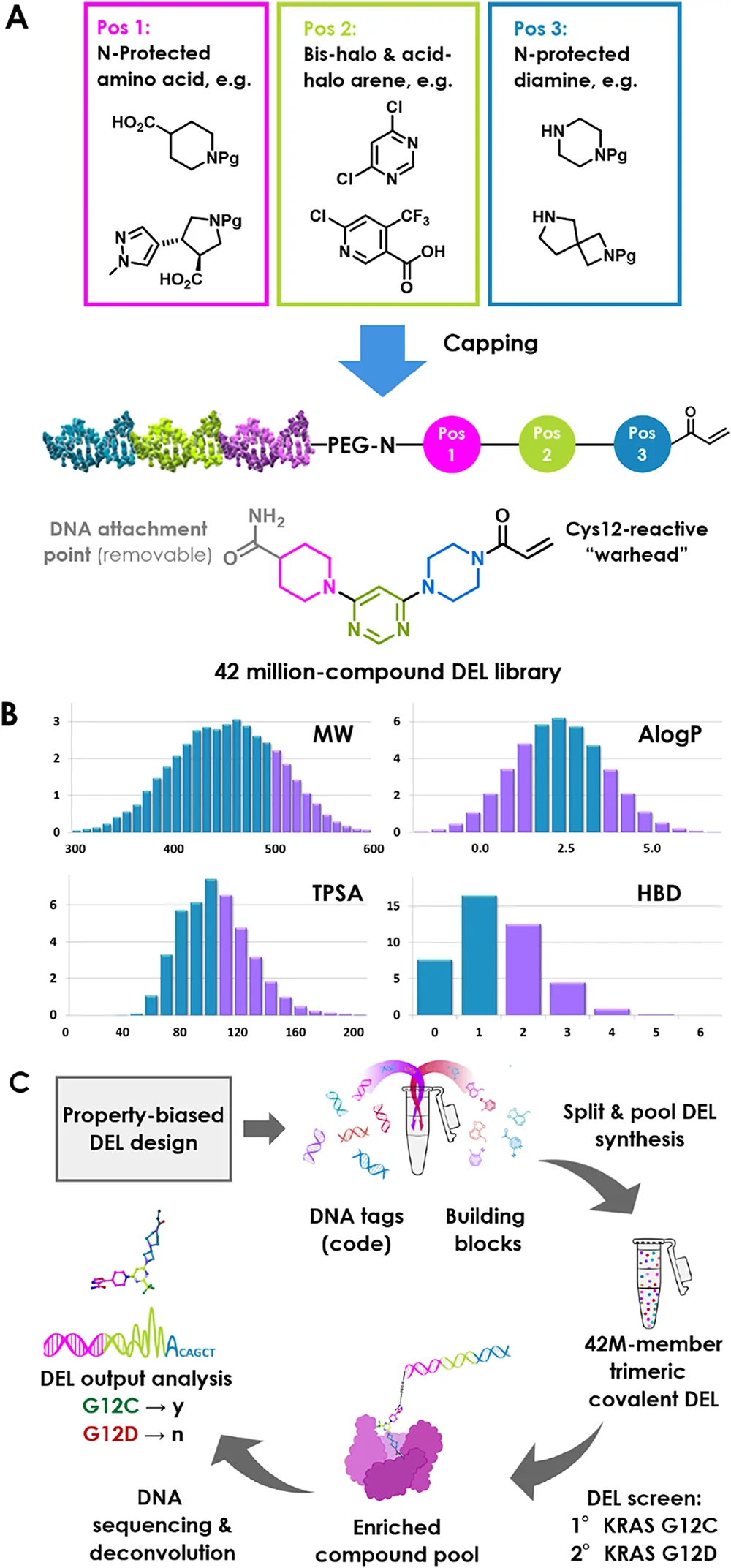
(A) DEL assembly strategy, with building block selection (positions 1–3) guided by physicochemical property constraints to bias fully elaborated DEL compounds toward favorable CNS MPO property space. (B) DEL physicochemical property distribution (based on a random sample of one million DEL trimers, excluding the amide DNA-attachment point). Library members in CNS MPO-favorable physicochemical property space are depicted in blue. *Y*-axis values indicate the number of DEL compounds in millions.^34^ (C) DEL synthesis, screening, and deconvolution process.

The selection of building block coupling chemistries for the assembly of this property-based library was a key consideration, as it defined both the range of building blocks (and linkage geometries) compatible with this design as well as the physicochemical properties of the resulting library. Although amide formation has been the dominant attachment chemistry for historical DELs due to its reliability, robustness, and diversity of amine and acid inputs,^37–39^ in this library, we elected to principally employ S_N_Ar coupling for library assembly due to the inherently lower molecular weight (MW) and reduced polar surface area (PSA) of the resulting library members. Although this decision reduced the number of building blocks compatible with our library design, it aligned with our strategy of prioritizing library physicochemical properties over simple building-block diversity.

We further fine-tuned the balance between structural diversity and physicochemical properties of the DEL prepared in this work by carefully evaluating the building blocks to be employed at each position in our library. For example, >85% of amino acids used in position 1 had a MW < 150, as did ∼60% of bis-halo or acid-haloarenes used in position 2, and ∼50% of diamines in position 3 had a MW < 175. To further control the property profile of the library, we focused on HBD count. At position 1, ∼60% of the BBs would contribute 1 HBD or less, while at positions 2 and 3, >80 and >60% of the BBs would contribute no HBD to the assembled compounds, respectively. The quality of our building blocks was reflected in their low PSA contribution, where BBs in positions 1 and 3 had PSA < 30 Å^2^ in ∼70 and ∼60% of the cases, respectively, while the incorporation of acid-haloarenes in position 2 resulted in ∼60% of BBs in this position having slightly higher PSA (< 50 Å^2^).^40^

The result of these library design choices can be seen in Figure 3B, where over half of all library members occupy CNS MPO-favorable physicochemical space (blue bars). Notably, more than 66% of library members contained one or fewer hydrogen bond donors (a key determinant of CNS penetrance), and a similar fraction fell within “rule-of-5” molecular weight space, collectively positioning hit compounds from this library for improved CNS penetrance and for efficient optimization into potent, bioavailable lead molecules.

Screening of the resulting 42 million-compound library was performed via incubation with either KRAS G12C (target protein) or KRAS G12D (counterscreen to exclude noncovalent binders) in five replicates each. DEL hits enriched in at least two of the five KRAS G12C screens and not enriched in the KRAS G12D screens were clustered by chemotype in the output analysis (Figure 4A). Six chemotype clusters were identified, and representatives from five chosen chemotypes were synthesized off-DNA. Their activity was confirmed using an in vitro biochemical assay that measured the inhibition of SOS1-catalyzed GDP/GTP exchange [coupled exchange (CE) assay].^41^

**Figure 4:**
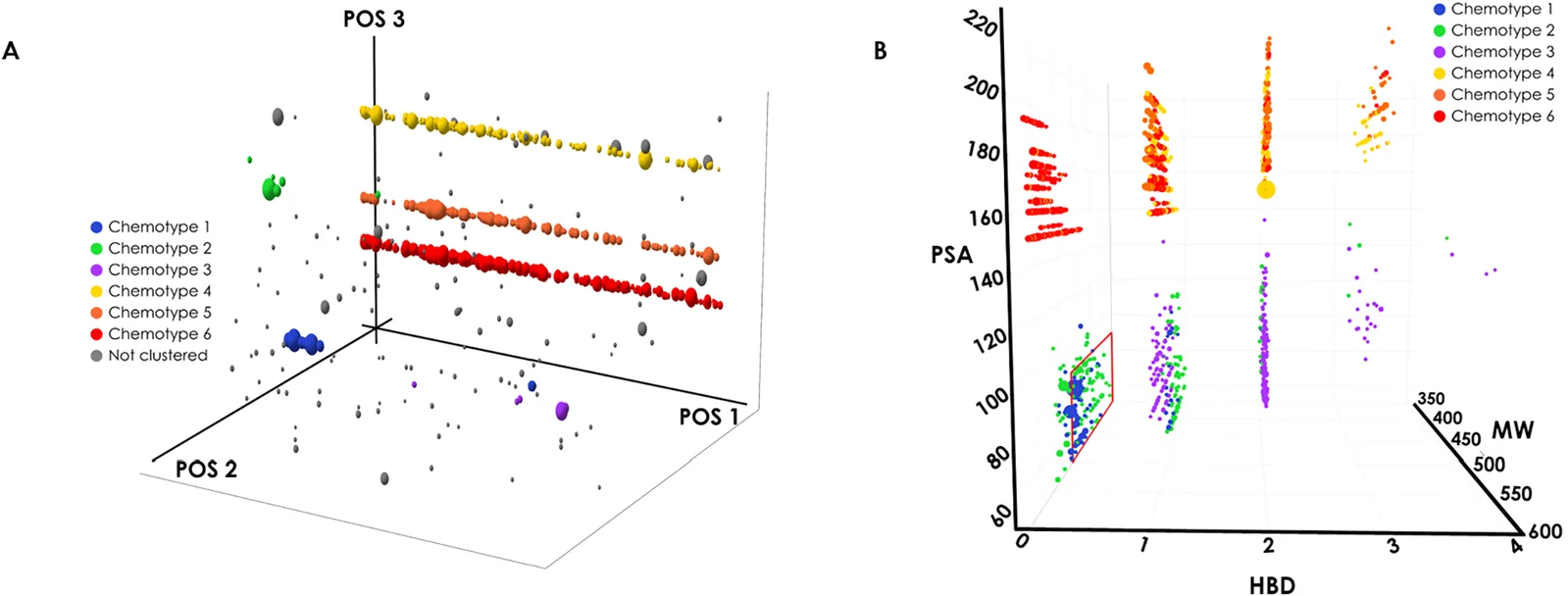
(A) 3D-plot of “hit” compounds enriched in KRAS G12C selections and not enriched in KRAS G12D selections, arrayed by structural principal component analysis (Pos 1–3). Each dot represents one compound; dot size represents the average enrichment factor in KRAS G12C selections (larger = higher enrichment). Structurally related compounds (Chemotypes 1–6) are depicted using shared colors. (B) 3D-plot of “hit” compounds in 3D property space (axes: hydrogen-bond donor count (HBD), polar surface area (PSA), and molecular weight (MW); properties calculated excluding the amide group linking hit compounds to DNA). Each dot represents one compound. Dot size represents the average enrichment factor in KRAS G12C selections. Structurally related compounds are depicted using shared colors. The red box highlights compounds with PSA < 90, MW < 500, and HBD = 0 ^34^ (ChatGPT 3.o was used to assist in the generation and refinement of graphical elements in (panels A & B).

### Hit Series Selection and Validation

To identify preferred hit series for subsequent follow-up, we further assessed our hit chemotypes according to several key physicochemical properties (MW, PSA, and HBD; see Figure 4B). Although the specific molecular characteristics most predictive of enhanced CNS penetration remain a topic of active discussion,^42,43^ there is general consensus that control of molecular weight (MW), polar surface area (PSA), and hydrogen bond donor (HBD) count are key factors in modulating CNS penetration.

On the basis of our prior experience in optimizing KRAS G12C inhibitor CNS exposure, we chose to heavily weight HBD count minimization in our series selection priorities. Accordingly, we initially focused our attention on chemotype 1 (blue spheres, Figure 4B), as most compounds in this cluster possessed very attractive physicochemical properties (PSA < 90, MW < 500, and HBD = 0, red box), with the majority of the library members belonging to this chemotype having HBD only from the secondary amide-based DNA linkages (linkage points that were potentially unrelated to KRAS binding and dispensable in subsequent optimization).

Following resynthesis and testing of chemotype 1 compounds in the SOS1-catalyzed coupled exchange assay, compound **1** (CE IC_50_ ∼ 5 μM) was identified as the top compound for subsequent follow-up (cf. Table 1). Gratifyingly, removal of the amide handle used for DNA-tethering (compound **2**) resulted in only a 10-fold decrease in potency, while significantly decreasing PSA and MW.^44^ In an effort to restore lost potency, we subsequently prepared a series of five-membered heteroaryl analogues of oxazole **2**. Replacing the oxazole with a 2-substituted thiazole afforded compound **3**, which both enhanced CE potency and reduced PSA, but most importantly confirmed the favorable biophysical properties of this scaffold by showing a brain-to-plasma partition coefficient (*K*_p,uu_) of 0.2. The corresponding 4-methylthiazole analogue **4** further enhanced CE potency (CE IC_50_ = 0.927 μM) and yielded a compound with measurable activity in a cell-based ERK phosphorylation (p-ERK) assay while maintaining a promising mouse brain *K*_p,uu_ value of 0.15. Further optimization ultimately identified dimethylpyrazole **5** as the most potent analogue in this series, showing low-micromolar activity in the p-ERK assay, albeit with an increased Pgp efflux ratio (ER)^45^ in MDCK cells and a decreased mouse brain *K*_p,uu_ value.

**Table 1:**
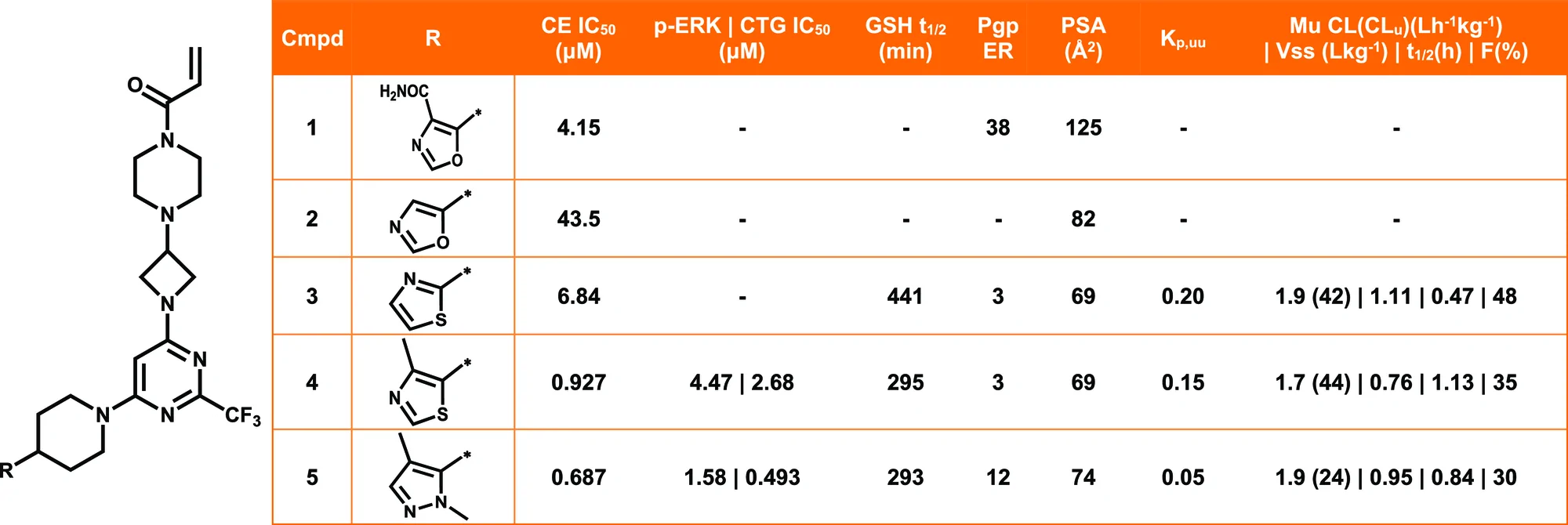
Chemotype 1 Heteroarene Optimization Yielded Enhanced Potency Analogues with Promising Brain *K*_p,uu_ ^a^

a CE, SOS1-catalyzed GDP/GTP exchange (AlphaScreen, KRAS G12C/c-RAF Ras binding domain, 2 h incubation); p-ERK, p-ERK1/2 MSD immunoassay, MIA PaCa-2 (*KRAS* G12C) cells, 2 h incubation; CTG, viability assessed by CellTiter-Glo luminescence assay (Promega; MIA PaCa-2 cells; 72 h incubation). All compounds tested in MIA PaCa-2 cells had CTG IC_50_ values >5 μM in A549 cells (*KRAS* G12S); GSH, *t*_1/2_, parent compound depletion (mass spectrometry, 5 mM GSH, 37 °C, pH 7.4 phosphate buffer); Pgp ER, Pgp efflux ratio in MDCK-hMDR1 cells (1 μM test concentration); *K*_p,uu_, ratio of brain-to-plasma drug concentrations (unbound) in CD1 mice (30 mg/kg, p.o. dose, exposures 1 h postdosing); in vivo PK, iv/p.o. dosing in CD1 mice (vehicle: iv, 1 mg/kg, DMSO; p.o., 5 mg/kg, 1% Tween 80, 2% HPMC, 97% water); PSA values were calculated using Canvas software omitting sulfur atoms for compounds **3** & **4**.

Early pharmacokinetic characterization of compound **5** in mice (100 mg/kg, p.o. (oral)) showed >2 h coverage of cellular p-ERK IC_50_ levels, prompting us to evaluate the pharmacodynamic effects of compound **5** in a human *KRAS* G12C xenograft mouse model (Figure 5).^46^ Gratifyingly, compound **5** showed p-ERK reduction comparable to a first-generation covalent KRAS G12C inhibitor positive control (*M*-(*S*)-4-(4-acryloyl-2-methylpiperazin-1-yl)-6-chloro-1-(2-cyclopropyl-4-methylpyridin-3-yl)-7-(2-fluorophenyl)­pyrido­[2,3-*d*]­pyrimidin-2­(1*H*)-one; p-ERK IC_50_ = 0.038 μM)^47^ albeit with ∼20× higher unbound plasma/tumor concentration.^48^

**Figure 5:**
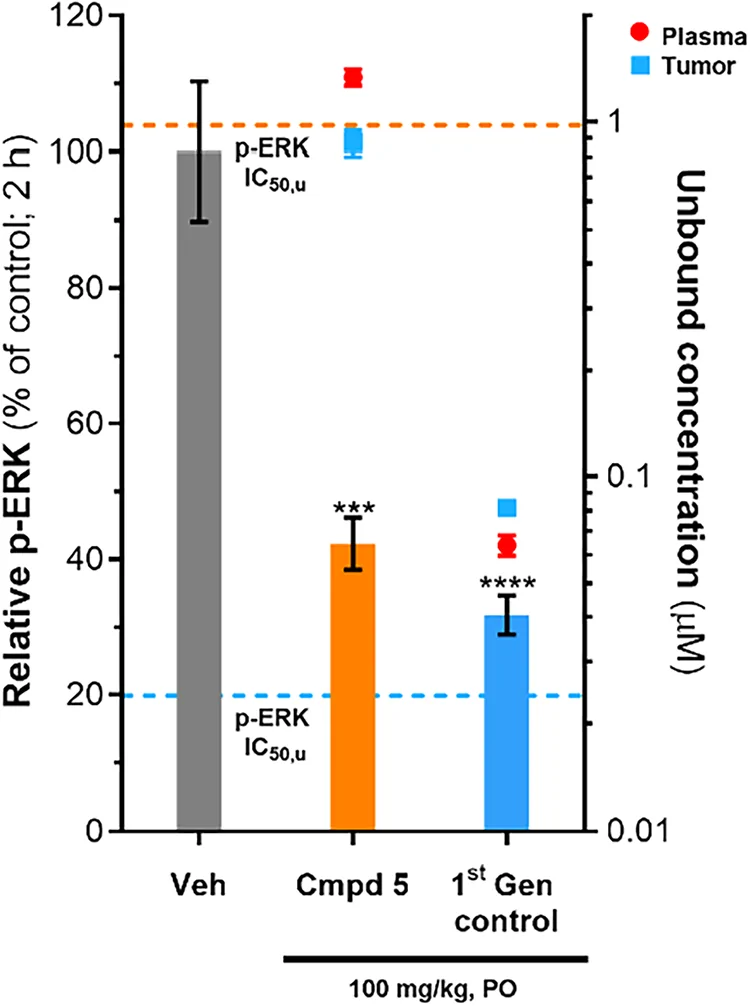
ERK1/2 phosphorylation levels in MIA PaCa-2 T2 tumors following oral administration (100 mg/kg) of compound **5** (orange bar), a 1st generation KRAS G12C inhibitor (blue bar) or vehicle (gray bar). Tumors were harvested 2 h postdosing. p-ERK levels were determined by electrochemical luminescent detection (Meso Scale Discovery). Data represent the mean ± SEM (*n* = 3/group). Unbound plasma and tumor concentrations are indicated by red circles and blue squares, respectively. All groups were compared to vehicle control by one-way ANOVA followed by Dunnett’s post hoc analysis, with *** *p* < 0.001 and **** *p* < 0.0001. Unbound p-ERK IC_50_ = in vitro cellular IC_50_·f_u,media_ (fraction unbound in cell culture media).

### Cocrystallization with KRAS G12C—Binding Mode Characterization

To guide further optimization of the compounds in Table 1, we attempted cocrystallization of several of these compounds with GDP-bound KRAS G12C. Figure 6 shows the resulting crystal structure of thiazole **3** covalently bound (via cysteine 12) to GDP-KRAS G12C. Compound **3** adopts a binding mode (panel B) with several differences relative to sotorasib (panel A). Like sotorasib, compound **3** induces formation of a ‘cryptic’ pocket (magenta) within the larger Switch-II pocket through side chain rotation of His95.

**Figure 6:**
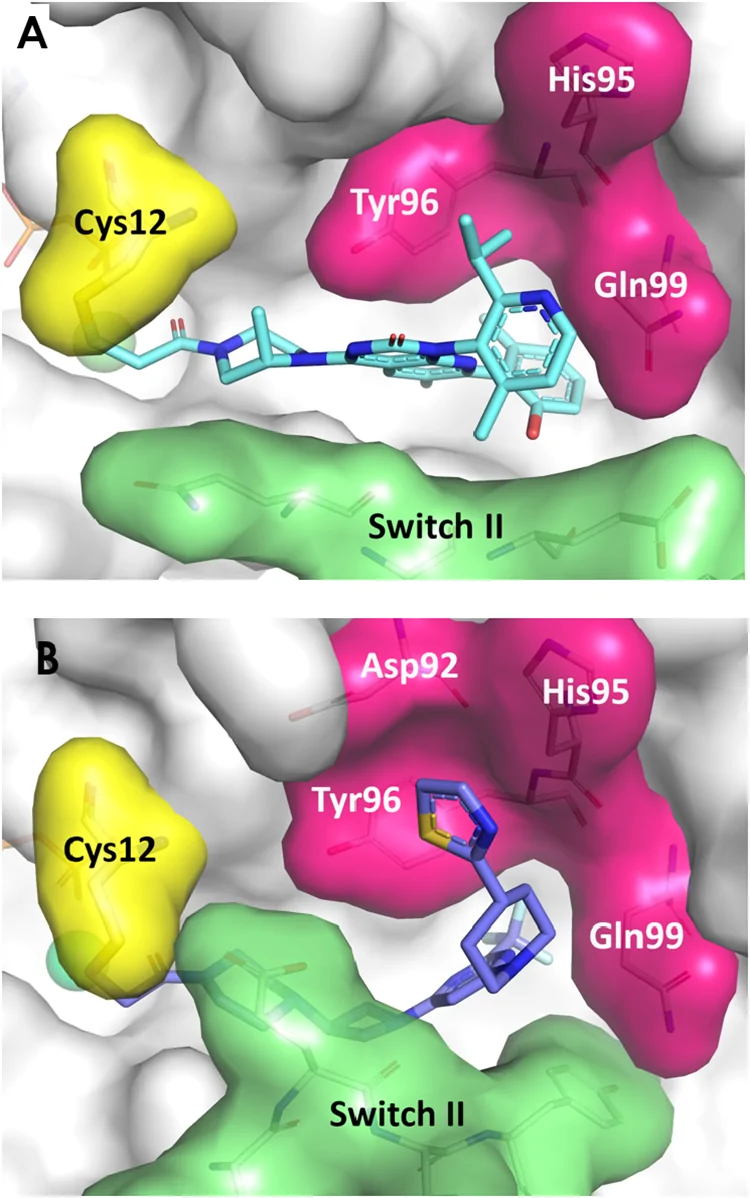
Comparison of the crystallographically determined binding modes of (A) sotorasib (PDB 6OIM) and (B) compound **3** (PDB 12KZ) to GDP-KRAS G12C. The KRAS protein and switch-II allosteric binding pocket are depicted in gray, the His95 cryptic pocket in magenta, the switch-II loop motif in green, and Cys12 in yellow.

The thiazole ring of compound **3** more completely fills this cryptic pocket, however, making van der Waals contacts not only with His95 and Tyr96 but also with Asp92. The trifluoromethylpyrimidine “core” of compound **3** also extends deeper into the lipophilic subpocket of KRAS occupied by the fluorophenol ring of sotorasib, making additional hydrophobic interactions. Additionally, the switch-II loop of KRAS (green) adopts an altered conformation in the presence of compound **3**, more completely enveloping **3** and contributing to additional van der Waals contacts with the inhibitor.

A crystal structure of the more potent pyrazole analogue **5** bound to KRAS G12C (Figure 7) provided additional insight into the binding mode of this chemotype. The piperazine ring of **5** engaged in a water-mediated hydrogen-bonding interaction with Tyr96,^49^ and the piperazine–azetidine “linker” region of **5** did not displace the frequently observed water molecule bound between Gly10 and Thr58. As with compound **3**, the trifluoromethyl group of **5** made extensive hydrophobic contacts with Val9, Met72, and Ile100, and the pyrimidine “core” of this scaffold was positioned between Tyr64 and Tyr96, making edge-to-face interactions with the phenolic side chains of both residues (and additional van der Waals interactions with Gln99).

**Figure 7:**
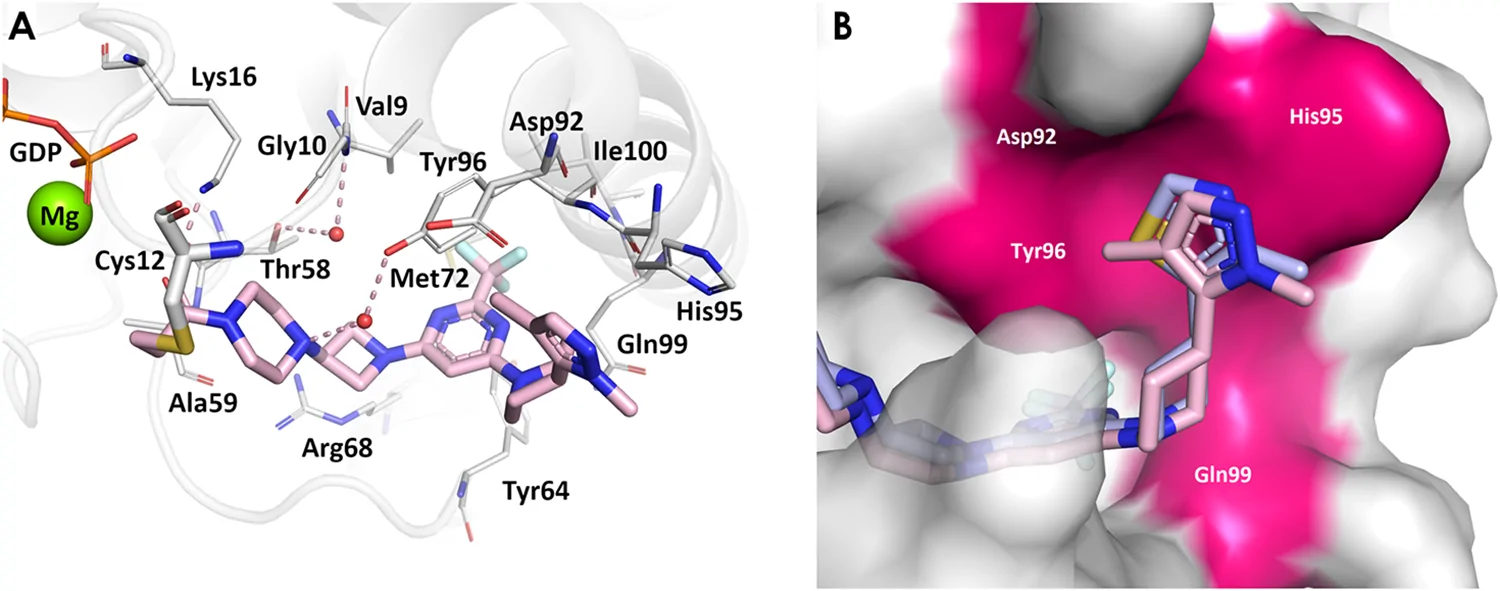
X-ray crystal structure of compound **5** (pink) bound to GDP-KRAS G12C (PDB 12LB). (A) Compound **5** bound to the switch-II pocket of KRAS. Ligand-proximal residues shown as sticks, with crystallographically resolved water molecules shown as red spheres, and hydrogen-bonding interactions shown as dashed pink lines. (B) Comparison of the binding modes of dimethylpyrazole **5** (pink) and methylthiazole **4** (blue; PDB 12LA). van der Waals surface representation of the Switch-II pocket is shown in gray and the His95 cryptic pocket in magenta.

Comparing inhibitor interactions with the cryptic pocket of KRAS, dimethylpyrazole **5** was found to generally preserve the binding mode observed with thiazole-containing compounds (cf. compound **4**, Figure 7B), with the C4-pyrazole methyl group of compound **5** replacing a sulfur atom of compound **4** in van der Waals contacts with Tyr96, but with both compounds otherwise generally making similar lipophilic contacts with the surrounding pocket. Based on modeling studies, the enhanced activity of compound **5** toward KRAS G12C versus thiazoles **3** and **4** appears attributable to the bound-conformation reinforcing effect of dimethyl substitution and to enhanced van der Waals interactions with Asp92 and His95.^50^

### Hit Optimization

Encouraged by the cellular activity seen with pyrazole **5**, we continued our effort to enhance the potency of this scaffold. Inspired by prior KRAS inhibitor optimization efforts,^51^ we sought to achieve this by displacing the Gly10-bound water molecule (see Figure 7A) via substitution of the azetidine ring. Examination of the compound **5** X-ray structure indicated limited space for the introduction of such substituents, leading us to explore mono- and bis-substitution of **5** with small alkyl substituents, a strategy which simultaneously aligned with our goal of limiting molecular weight increases to favor high CNS penetration (Table 2).

**Table 2:**
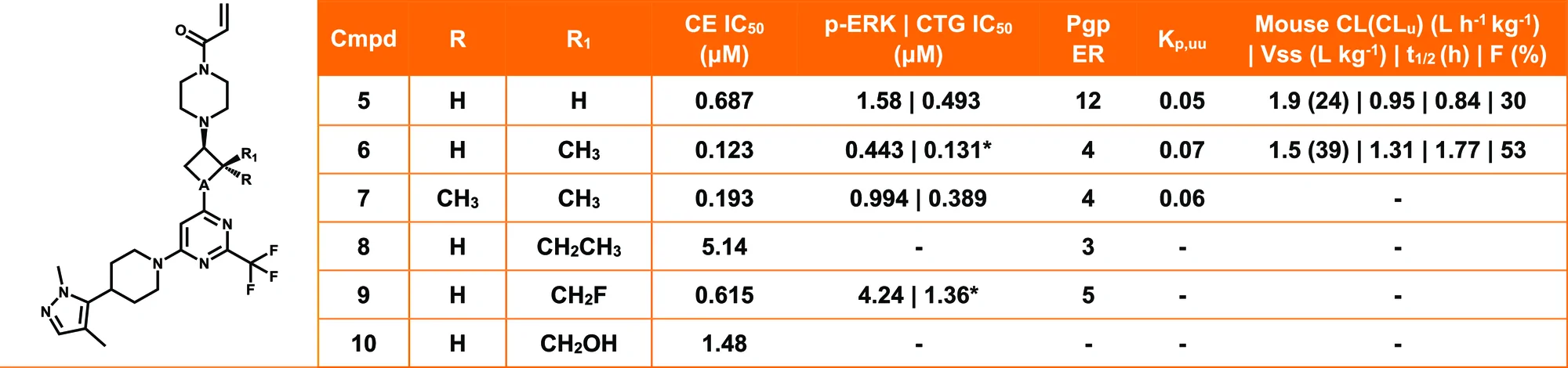
Azetidine Substitution Enhanced Potency, While Maintaining CNS-Penetrant Properties^a^

a CE, SOS1-catalyzed GDP/GTP exchange (AlphaScreen, KRAS G12C/c-RAF Ras binding domain, 2 h incubation); p-ERK, p-ERK1/2 MSD immunoassay, MIA PaCa-2 (KRAS G12C) cells, 2 h incubation; CTG, viability assessed by CellTiter-Glo luminescence assay (Promega; MIA PaCa-2 cells; 72 h incubation). All compounds tested in MIA PaCa-2 cells (except compound **9**, untested) had CTG IC_50_ values >5 μM in A549 cells (*KRAS* G12S); Pgp ER, Pgp efflux ratio in MDCK-hMDR1 cells (1 μM test concentration); K_p,uu_, ratio of brain-to-plasma drug concentrations (unbound) in CD1 mice (30 mg/kg, p.o. dose, exposures 1 h postdosing); in vivo PK, i.v./p.o. dosing in CD1 mice (vehicle: i.v., 1 mg/kg, DMSO; p.o., 5 mg/kg, 1% Tween 80, 2% HPMC, 97% water);

* *n* = 1.

*cis*-Monomethyl substitution of pyrazole **5** provided *(R)*-methylazetidine **6**, which showed an approximately 5-fold increase in biochemical and cellular potency while leading to a 3-fold reduction in Pgp efflux ratio. Although methyl substitution did not meaningfully enhance brain *K*_p,uu_, compound **6** also showed nearly twice the oral bioavailability of compound **5**. Notably, although *bis*-methyl substitution (compound **7**) largely recapitulated the activity of monomethyl analogue **6**, *trans-*methyl substitution and *cis*-(*S*)-methylation of the epimeric (*3S*)-piperazinylazetidine ring both led to analogues with significantly reduced potency (see the Supporting Information). Increasing the size of the substituent to ethyl resulted in a 10× loss of activity (compound **8**). Hypothesizing that additional activity might be achieved by not only displacing the Gly10 water but by introducing ligand substituents to mimic this water’s interaction with Gly10, we prepared fluoromethyl analogue **9** and hydroxymethyl analogue **10**, both of which led to reduced biochemical and cellular activity.

Leveraging insights from the crystal structure of pyrazole **5** (Figure 7), we also explored replacement of the solvent-exposed pyrazole *N*-methyl group to modulate potency and physicochemical properties (Table 3). Although many methyl replacements, e.g., cyclobutyl (**11**), difluorocyclobutyl (**12**), and 2-fluoroethyl (**13**), failed to meaningfully improve biochemical and cellular potency and showed elevated in vitro clearance, oxetanyl analogue **14** led to modest improvements in biochemical and cellular potency, and methyoxyethyl analogue **15** led to a meaningful improvement in brain *K*_p,uu_ (∼3-fold).

**Table 3:**
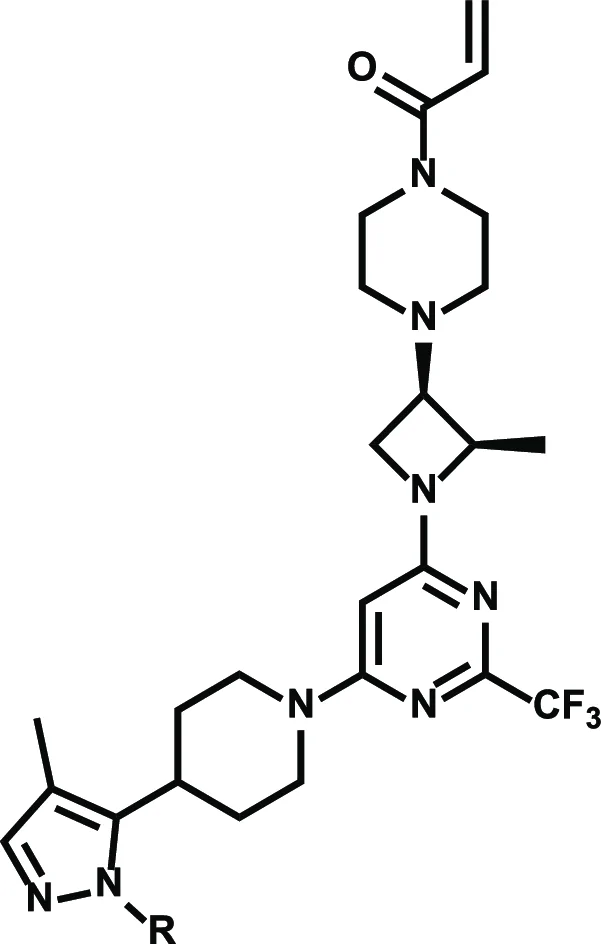

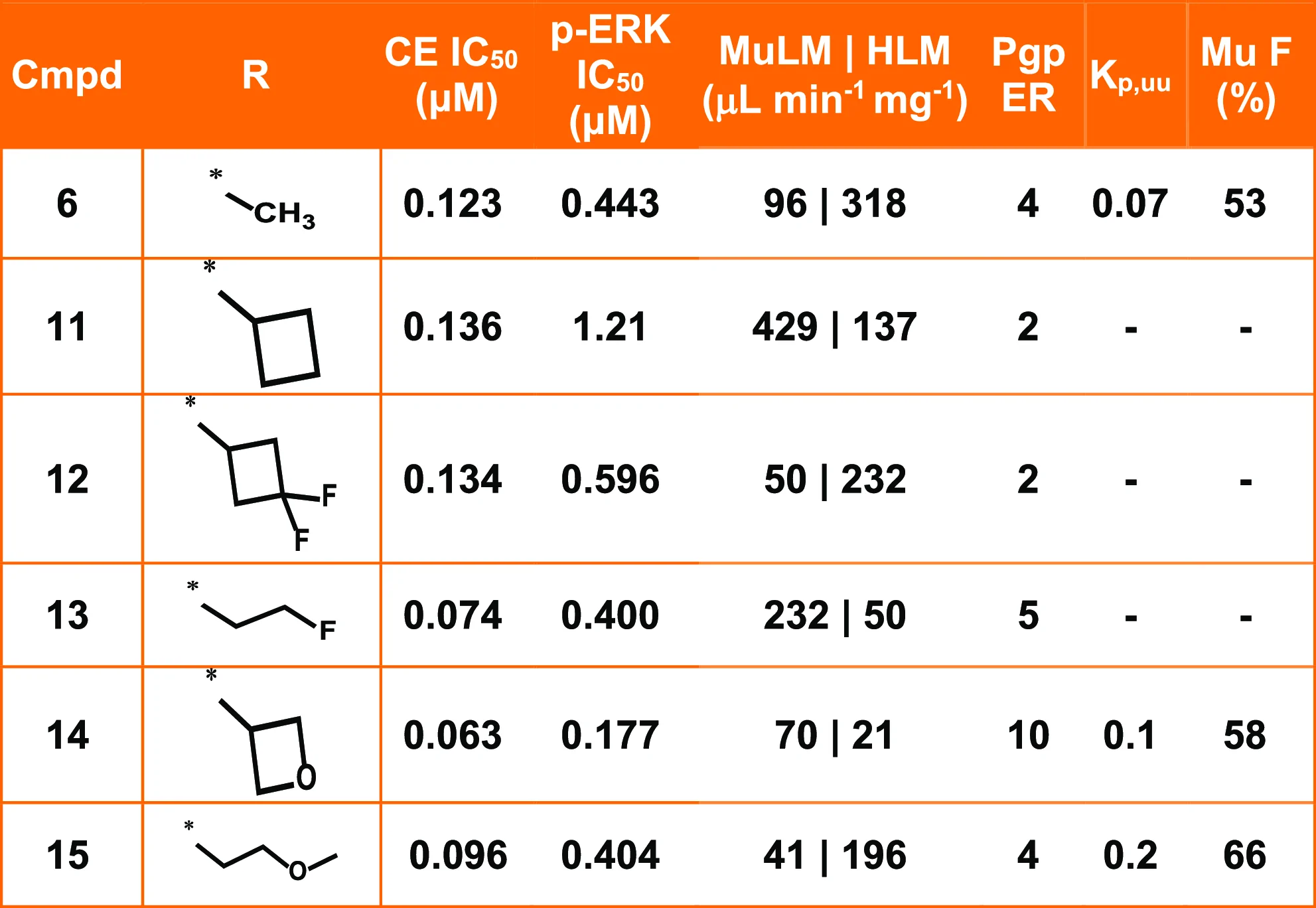
Impact of Pyrazole *N*-Substitution on Potency and CNS Penetrance^a^

a CE, SOS1-catalyzed GDP/GTP exchange (AlphaScreen, KRAS G12C/c-RAF Ras binding domain, 2 h incubation); p-ERK, p-ERK1/2 MSD immunoassay, MIA PaCa-2 (KRAS G12C) cells, 2 h incubation; MuLM/HLM, mouse/human liver microsome in vitro clearance; Pgp ER, Pgp efflux ratio in MDCK-hMDR1 cells (1 μM test concentration); *K*_p,uu_, ratio of brain-to-plasma drug concentrations (unbound) in CD1 mice (30 mg/kg, p.o. dose, exposures 1 h postdosing); Mu F, mouse oral bioavailability;

Taking compound **15** as a starting point, we subsequently examined whether alteration of this compound’s pyrimidine core (Table 4) could impart added potency through modulation of the π–π stacking interaction between this ring, Tyr96, and Tyr64 (cf. Figure 7A). Unfortunately, replacement of the pyrimidine ring with a pyridine ring (**16**) or other isomeric pyrimidines (**17**–**19**) led only to compounds with reduced potency.

**Table 4:**
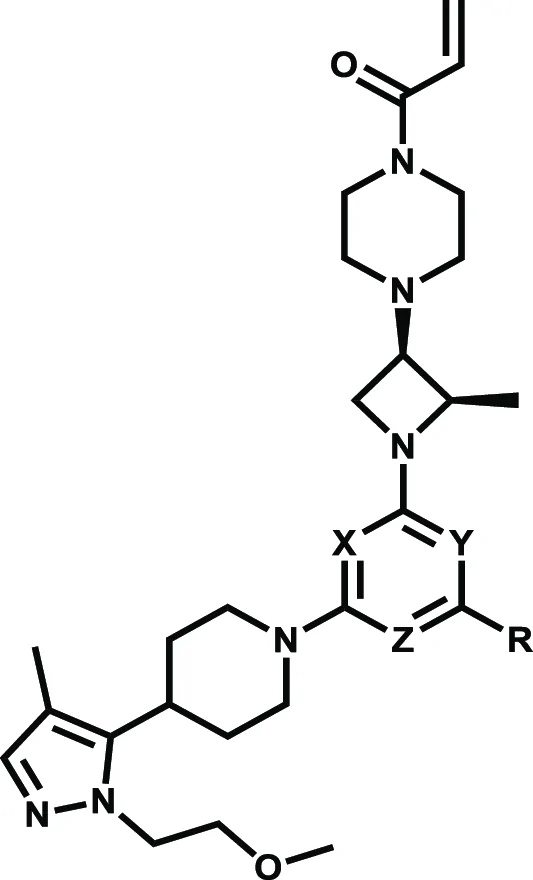

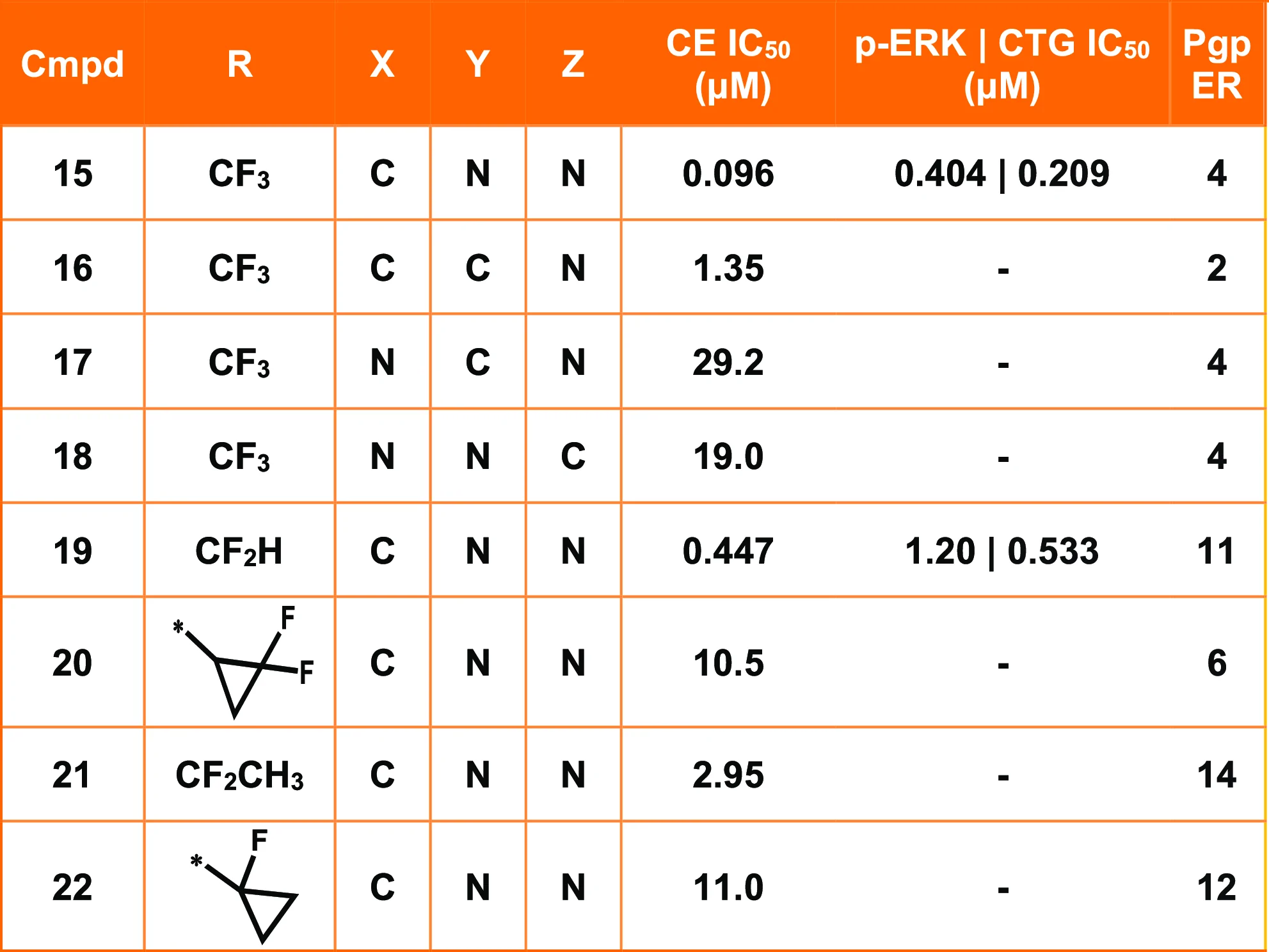
Azine Core SAR^a^

a CE, SOS1-catalyzed GDP/GTP exchange (AlphaScreen, KRAS G12C/c-RAF Ras binding domain, 2 h incubation); p-ERK, p-ERK1/2 MSD immunoassay, MIA PaCa-2 (KRAS G12C) cells, 2 h incubation; CTG, viability assessed by CellTiter-Glo luminescence assay (Promega; MIA PaCa-2 cells; 72 h incubation); Pgp ER, Pgp efflux ratio in MDCK-hMDR1 cells (1 μM test concentration).

We next turned our attention to the optimization of lipophilic contacts between the trifluoromethyl group of compound **15** (*R* = CF_3_) and the switch-II pocket, with a focus on small, fluorinated substituents that mimicked the noncovalent interactions between CF_3_ and the KRAS protein seen in prior X-ray structures. Replacement of the trifluoromethyl substituent with a difluoromethyl substituent (**19**) resulted in a 3-fold loss in potency, and larger fluorinated substituents (e.g., compounds **20**–**22**) proved even less well-tolerated. Less highly fluorinated/less electron-withdrawing substituents also led to increased Pgp efflux (an effect we attributed to increased basicity of the pyrimidine nitrogens), further discouraging us from further exploration of such substituents.

Having successfully optimized inhibitor potency and properties through prior modulation of the cryptic pocket-engaging heteroaryl ring, we returned our attention to optimization of this ring, next exploring the impact of six-membered ring introduction on inhibitor potency and biophysical properties (Table 5). Initial efforts with *bis-ortho*-dimethylated pyridyl (**23**) and pyridazyl (**24**) rings showed both substituents to deliver analogues with comparable biochemical and cellular potency to 5-membered ring analogues (albeit with significantly increased Pgp-mediated efflux in the case of pyridazine **24**). Interestingly, the corresponding pyrimidyl analogue, **25**, was ∼5-fold less active in in vitro assays, presumably due to less favorable positioning of its polar nitrogen atoms within the cryptic pocket (e.g., proximal to Asp92).

**Table 5:**
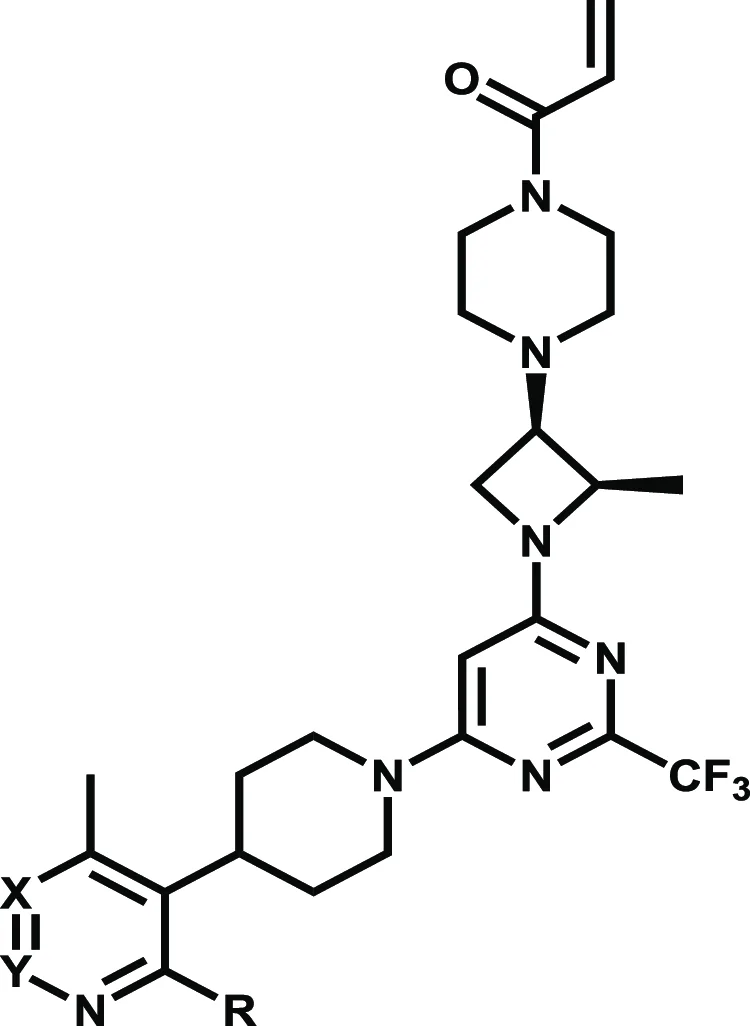

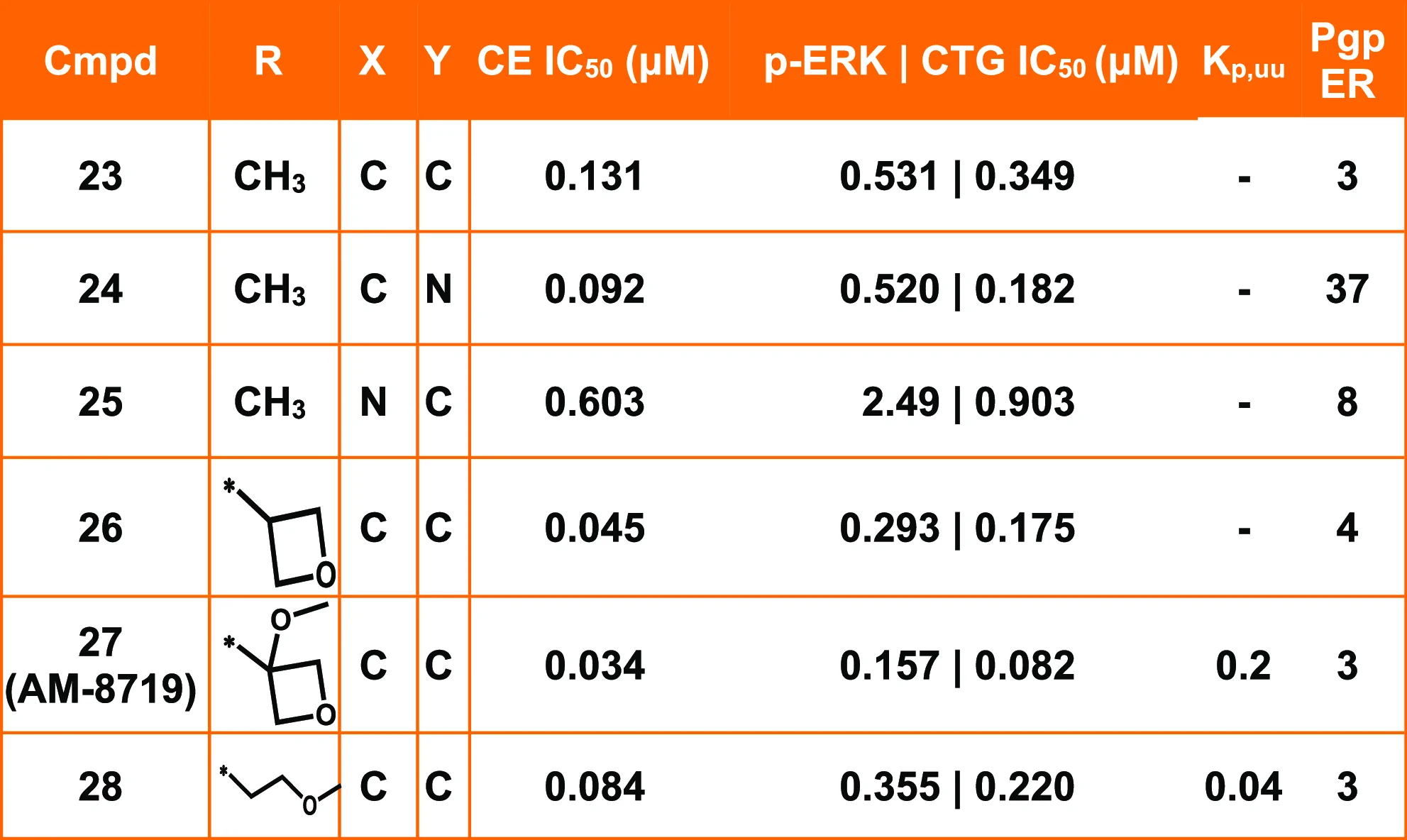
Heteroarene Exploration Revisited^a^

a CE, SOS1-catalyzed GDP/GTP exchange (AlphaScreen, KRAS G12C/c-RAF Ras binding domain, 2 h incubation); p-ERK, p-ERK1/2 MSD immunoassay, (MIA PaCa-2 (*KRAS* G12C) cells; 2 h incubation); CTG, viability assessed by CellTiter-Glo luminescence assay (Promega; MIA PaCa-2 cells; 72 h incubation); *K*_p,uu_, ratio of brain-to-plasma drug concentrations (unbound) in CD1 mice (30 mg/kg, p.o. dose, exposures 1 h postdosing); Pgp ER, Pgp efflux ratio in MDCK-hMDR1 cells (1 μM test concentration).

Attempting to leverage SAR trends observed with earlier 5-membered cryptic pocket-engaging groups, we next examined whether small, ethereal substituents could again impart enhanced potency and improved CNS penetration. Gratifyingly, preparation of the 2-(3-oxetanyl) analogue **26** led to significant gains in in vitro potency (without an adverse impact to Pgp-mediated efflux), and the corresponding methoxyoxetanyl derivative (**27**) performed similarly. Methoxyethyl substitution (**28**) was likewise well-tolerated from a potency perspective, however this compound proved less brain penetrant than compound **27**. Ultimately, methoxyoxetane **27** (**AM-8719**) was the most promising analogue to emerge from these optimization efforts, affording a molecule with both strong cellular activity (p-ERK IC_50_ = 157 nM) and very promising CNS penetration (brain *K*_p,uu_ = 0.2).

Crystallization with KRAS G12C (Figure 8) revealed **AM-8719** to adopt a similar binding mode to that observed with earlier azole analogues. The piperazine ring of **AM-8719** again formed a water-bridged hydrogen-bonding interaction with Tyr96, but as planned, the azetidine methyl substituent displaced the Gly10-bound water molecule, presumably contributing to the potency of this analogue. The ether-rich methoxyoxetane substituent was found to be oriented away from the lipophilic His95 cryptic pocket, where it could interact with bulk solvent, with the increased steric bulk of this substituent likely further favoring the preferred perpendicular orientation of the pyridine and piperidine rings, reinforcing numerous hydrophobic contacts between the pyridine ring and His95 pocket.

**Figure 8:**
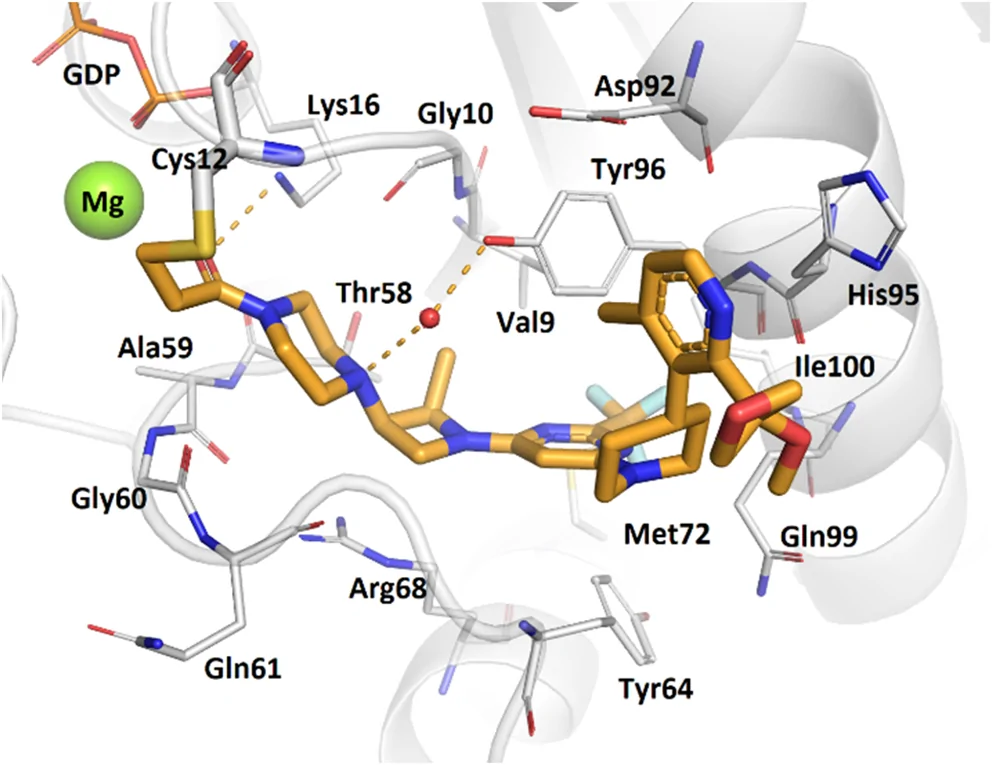
X-ray crystal structure of compound **27** (**AM-8719**) bound to GDP-KRAS G12C (PDB 12LC). Crystallographically resolved water is shown as a red sphere, and hydrogen-bonding interactions are shown as dashed orange lines.

Gratifyingly, the optimization of property-biased DEL-screening hit **1** to **AM-8719** was achieved while maintaining favorable CNS MPO parameters (Figure 10). In addition to achieving promising KRAS inhibitory activity in a hydrogen-bond donor-free scaffold, **AM-8719** (white markers, Figure 10) achieved a 200-fold potency increase relative to DEL-derived starting point **3** while preserving CNS MPO-favorable log*P*, log*D*, TPSA, and mbpKa properties and only modestly increasing MW (+20%). This resulted in a KRAS G12C inhibitor with a class-leading CNS MPO score (4.6) that showed promising CNS exposure not only in mouse (brain *K*_p,uu_ = 0.2) but also in rat (brain *K*_p,uu_ = 0.1).^52^

As illustrated in Table 6, **AM-8719** additionally demonstrated promising cross-species PK properties (oral bioavailability = 33–78%, *t*_1/2_ = 2.4–3.5 h), excellent stability toward nontarget thiols (GSH *t*_1/2_ = 267 min),^53^ and efficient alkylation of KRAS G12C (*k*_inact_/*K*_I_ = 1,123 M^–1^ s^–1^). Based on the strength of this profile, **AM-8719** was advanced to mouse xenograft pharmacodynamic and efficacy studies (Figure 9). Oral administration of **AM-8719** (30 mg/kg, single dose) led to significant suppression of ERK phosphorylation in *KRAS* G12C tumors (MIA PaCa-2 T2; 2 h postdosing; Figure 9A).^48^ Gratifyingly, unbound brain drug exposure in this study was within 2-fold of the cellular p-ERK IC_50_ value in this experiment, again confirming the promising CNS penetrance of **AM-8719** (Figure 10).

**Figure 9:**
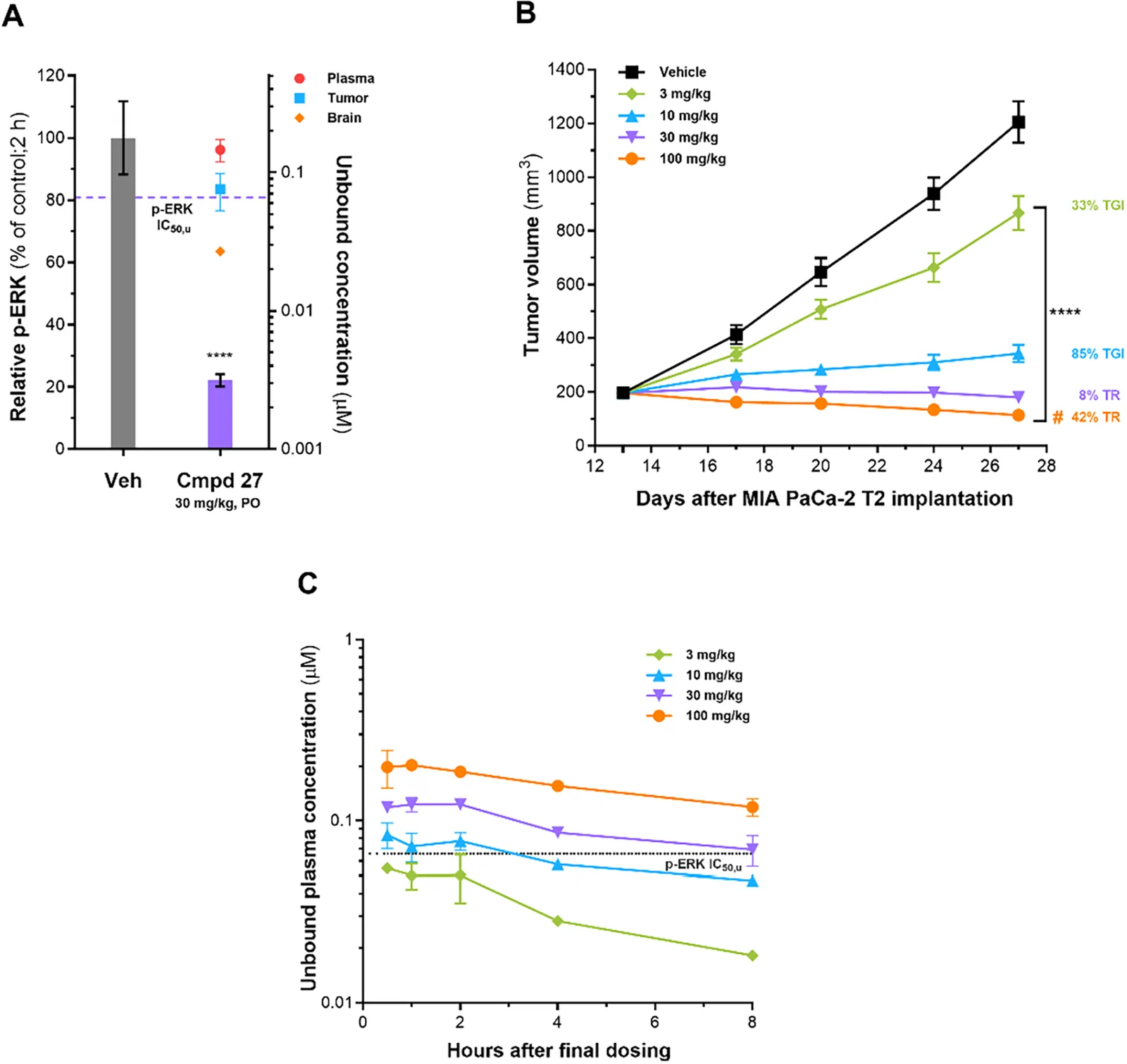
Once-daily, oral dosing of compound **27** (**AM-8719**) results in a robust decrease in p-ERK levels and tumor regression in a *KRAS* G12C tumor xenograft model (MIA PaCa-2 T2). (A) Inhibition of ERK1/2 phosphorylation following administration (30 mg/kg, p.o., single dose) of **AM-8719** (purple bar) or vehicle (gray bar). Tumors were harvested 2 h postdosing. p-ERK levels were determined by electrochemical luminescent detection (MSD). Data represent mean ± SEM (*n* = 3/group); ****p < 0.0001 by one-way ANOVA followed by Dunnett’s post hoc analysis. Plasma, tumor, and brain exposures of **AM-8719** are shown by red circles, blue squares, and orange diamonds, respectively. (B) Tumor growth inhibition (TGI) or regression (TR) following once-daily oral administration of **AM-8719** (3, 10, 30, or 100 mg/kg). Data represent the mean tumor volume ± SEM (*n* = 10/group); ****p < 0.0001 by RMANOVA followed by Dunnett’s post hoc analysis, #*p* < 0.0001 regression by paired *t-test*. (C) Mean unbound plasma exposures (±SEM, *n* = 2/group) of **AM-8719** following final dosing in the efficacy study shown in panel B. Unbound p-ERK IC_50_ = in vitro cellular IC_50_·f_u,media_ (fraction unbound in cell culture media). Tissue density was assumed to be 1 mg/mL.

**Figure 10:**
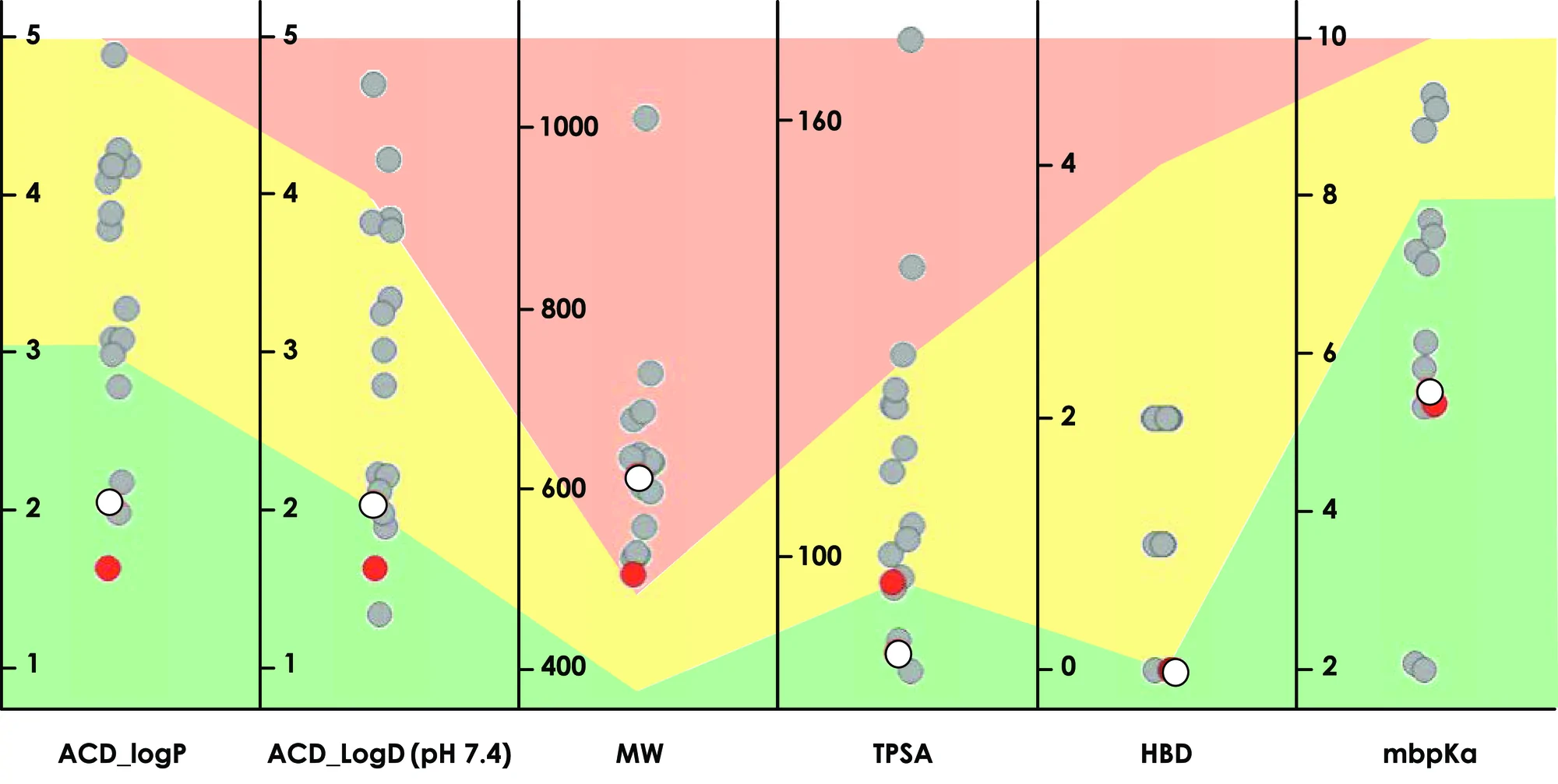
CNS MPO properties of thiazole screening hit **3** (red) and **AM-8719** (white) as compared to clinical KRAS G12C inhibitors (gray). As shown, property-biased DEL-screening and hit optimization both lead to the identification of covalent KRAS G12C inhibitors with significantly improved CNS MPO properties and CNS MPO scores (4.2–4.6) vs clinical KRAS G12C inhibitors (see the Supporting Information).

**Table 6:** In Vitro & In Vivo ADME Characterization of Compound 27 (AM-8719)

**mouse CL** (L h^–1^ kg^–1^) | **Vss** (L kg^–1^) | **t**1/2 (h) | **F** (%)^a^	**0.3** | **0.64** | **2.4** | **53**
**rat CL** (L h^–1^ kg^–1^) | **Vss** (L kg^–1^) | **t**1/2 (h) | **F** (%)^b^	**2.9** | **5.0** | **3.1** | **78**
**dog CL** (L h^–1^ kg^–1^) | **Vss** (L kg^–1^) | **t**1/2 (h) | **F** (%)^c^	**1.3** | **2.8** | **3.5** | **33**
**Hep CL Mu** | **R** | **D** | **Hu** (μL min^–1^ 10^–6^ cells)^d^	**47** | **29** | **42** | **27**
**PPB Mu** | **R** | **D** | **Hu** (0.25 μM, UC, *f*_u_)	**0.01** | **0.07** | **0.03** | **0.06**
**KRAS G12C *k***_**inact**_ **/*K***_**I**_ (M^–1^ s^–1^)^e^	**1123**
**GSH t**1/2 (min)^f^	**267**
**Solubility PBS** | **HCl** | **SIF** (μM)^g^	**39** | **>500** | **206**
**Log *P*** | **p** *K*_**a**_ ^h^	**2.8** | **3.5**
**Pgp ER** | **BCRP ER** | ***P***_**app**_ (μcm/s)^i^	**3** | **1** | **13**
**mouse** | **rat K**_**p,uu,brain**_ ^j^	**0.2** | **0.1**

a i.v./p.o. dosing in CD1 mice (vehicle: i.v., 1 mg/kg, DMSO; p.o., 5 mg/kg, 1% Tween 80, 2% HPMC, 97% water).

b i.v./p.o. dosing in fed Sprague–Dawley rats (vehicle: i.v., 1 mg/kg, DMSO; p.o., 5 mg/kg, 1% Tween 80, 2% HPMC, 97% water).

c i.v./p.o. dosing in Beagle dog (vehicle: i.v., 0.5 mg/kg, 30% Captisol, 70% water; p.o., 2 mg/kg, 1% Tween 80, 2% HPMC, 97% water).

d See the Supporting Information for details.

e *k*_inact_/*K*_I_ was determined from time-course relative %bound data at varied inhibitor concentrations, as assessed by mass spectrometry.^56^

f *t*_1/2_ determined by parent depletion (MS detection) in 5 mM GSH, 37 °C, pH 7.4 aqueous phosphate buffer.^53^

g PBS = phosphate-buffered saline; SIF = simulated intestinal fluid.

h Generated by Analiza Inc. (Cleveland, OH).

i Pgp ER, Pgp efflux ratio in MDCK-hMDR1 cells (1 μM test concentration), *P*_app_, passive permeability *P*_A2B_ (1 μM test concentration, 10 μM elacridar) in MDCK-hMDR1 cells, see the Supporting Information for BCRP assay details.

j *K*_p,uu,brain_, ratio of brain-to-plasma drug concentrations (unbound) in CD1 mice or fed Sprague–Dawley rats (30 mg/kg, p.o. dose, exposures 1 h postdosing);

Subsequent assessment of **AM-8719** in a mouse xenograft efficacy study (MIA PaCa-2 T2; QD, oral dosing) led to 85% tumor growth inhibition at 10 mg/kg and significant tumor regression at 30–100 mg/kg, on par with the preclinical efficacy observed with sotorasib in this same model.^54,55^ In line with the observed 85% tumor growth inhibition, plasma PK measurements following completion of dosing showed that, at the 10 mg/kg dose level, **AM-8719** unbound plasma concentration exceeded the in vitro unbound p-ERK IC_50_ (2 h) value for 3 h and provided >8 h coverage at higher doses.

## Conclusions

In this work, we demonstrate the successful application of a property-biased DEL-screening approach in the identification of CNS-penetrant covalent KRAS G12C inhibitors. By designing a DEL library not with the aim of covering the largest *structural* space but rather with the aim of providing broad coverage of a targeted biophysical *property* space (i.e., that space defined by favorable CNS MPO properties), we identified structurally novel covalent inhibitors of KRAS G12C that demonstrated significantly enhanced preclinical CNS penetration relative to clinical KRAS G12C inhibitors.

Subsequent structure-based optimization of these CNS-penetrant leads, with a focus on maintaining favorable CNS MPO properties, culminated in the identification of a novel covalent KRAS G12C inhibitor, **AM-8719**, which showed promising in vivo brain exposure and potent antiproliferative activity in a mouse xenograft model. Key strategies in optimizing DEL leads toward **AM-8719** included targeted displacement of a water molecule bound to Gly10 and ortho substituent optimization of the His95 ‘cryptic pocket’-occupying aryl ring to provide improved noncovalent contacts with KRAS. Further optimization of this lead scaffold will be reported in due course.

## Experimental Section

### General Synthetic Procedures

All materials were obtained from commercial suppliers and used without further purification unless otherwise noted. Anhydrous solvents were obtained from Acros Organics and Sigma-Aldrich. Reactions involving air- or moisture-sensitive reagents were performed under a nitrogen or argon atmosphere. Silica gel chromatography was performed using prepacked silica gel cartridges (Redi-Sep Rf, Teledyne ISCO or Biotage Sfår, Biotage Isolera). Reverse-phase high-performance liquid chromatography (HPLC) purification was performed using Gilson (Middleton, WI) workstations. NMR spectra were acquired on Bruker Avance 400, 500, or 600 MHz spectrometers equipped with 5 mm BBFO probes. All compounds tested in *in vitro* and *in vivo* assays were >95% pure by HPLC analysis as determined by an Agilent 1100 or 1260 multiwavelength detector (215 nm detection) and an Advanced Materials Technology HALO C18 column (50 × 3.0 mm, 2.7 μm) at 40 °C with a 2.0 mL/min flow rate using a 5 to 95% gradient of acetonitrile/water with 0.1% trifluoroacetic acid over 1.5 min. Low-resolution MS data were obtained concurrently with UV chromatography using an Agilent G1956B LC MSD SL (6120B, 6130B, or 6140A) quadrupole MS in positive electrospray ionization mode. Enantiomeric and diastereomeric excesses were determined by SFC (Waters Acquity UPC2 or Agilent 1260 Infinity analytical systems). The following abbreviations are used to designate NMR peak multiplicities: s = singlet, d = doublet, t = triplet, q = quartet, m = multiplet, quint = quintet, br = broad. High-resolution mass spectra (HRMS) and ultrahigh-pressure liquid chromatography (UHPLC) chromatograms were recorded on an Orbitrap Fusion Tribrid MS Thermo Fisher coupled to a Thermo Fisher Vanquish UHPLC with PDA. Column: BEH C18 Column (2.1 × 50 mm, 1.7 μmm) by Waters at 45 °C. Solvent A: 0.1% formic acid in water and Solvent B: MeCN with 0.1% formic acid. Gradient, starting at 95% A to 100% B over 3.25 min; flow rate: 600 μL/min. MS settings: positive mode; resolution: 12,000; MS spectrum from 150–1000 *m*/*z*. UV detection at 254 nm (Scheme 1).

**Scheme 1:**
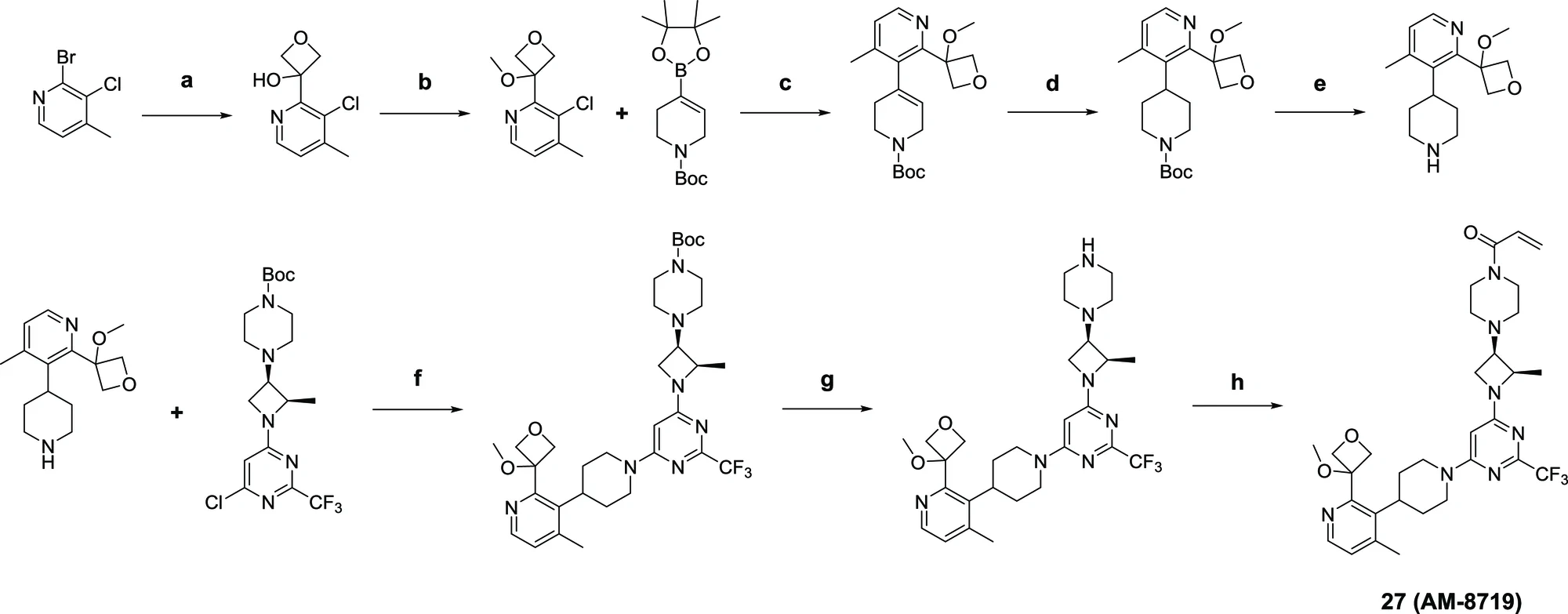
Synthesis of 27 (AM-8719)^a^

a Reagents and conditions: (a) *n*BuLi, oxetan-3-one, THF, −78 °C, 55%; (b) NaH, MeI, THF, 0 °C to RT, 93%; (c) S-Phos Pd G3, K_2_CO_3_, 1,4-Dioxane:H_2_O (3:1), 100 °C, 73%; (d) Pd/C, Pd­(OH)_2_/C, H_2_ (1 atm), MeOH, RT, 46%; (e) TFA, DCM, RT, quant.; (f) DIPEA, DMA, 100 °C, 66%; (g) TFA, DCM, RT, quant.; (h) DIPEA, acryloyl chloride, DCM, 0 °C, 61%.

### *Synthesis of AM-8719 (Compound 27)

#### Step 1. 3-(3-Chloro-4-Methylpyridin-2-yl)­Oxetan-3-ol

To a solution of 2-bromo-3-chloro-4-methylpyridine (15 g, 72.7 mmol) in THF (225 mL), a 2.5 M solution of *n*BuLi in hexanes (29.6 mL, 74.1 mmol) was added dropwise, at −78 °C. The resulting mixture was stirred at −78 °C for 30 min. A solution of oxetan-3-one (4.71 g, 65.4 mmol) in THF (15 mL) was then added dropwise over 2 min at −78 °C, and the resulting mixture was stirred for 30 min. The reaction mixture was diluted with a saturated aqueous solution of ammonium chloride at −78 °C and extracted with ethyl acetate (3 × 250 mL). The combined organic layers were dried over sodium sulfate, filtered, and concentrated in vacuo. The crude material was absorbed onto a plug of silica gel and chromatographically purified using a Redi-Sep prepacked silica gel column (350 g), eluting with a gradient of 60% to 80% ethyl acetate in hexanes. 3-(3-Chloro-4-methylpyridin-2-yl)­oxetan-3-ol (8 g, 40.1 mmol, 55% yield) was obtained as an off-white solid. *m*/*z* (ESI) = 200.1 (M + H)^+^.

#### Step 2. 3-Chloro-2-(3-Methoxyoxetan-3-yl)-4-Methylpyridine

To a solution of 3-(3-chloro-4-methylpyridin-2-yl)­oxetan-3-ol (4 g, 20.0 mmol) in tetrahydrofuran (40 mL) was added sodium hydride (55 wt % dispersion in mineral oil) (1.31 g, 30.1 mmol) at 0 °C. The resulting mixture was stirred at 0 °C for 30 min, and methyl iodide (1.63 mL, 26.0 mmol) was added. The reaction mixture was stirred at room temperature for 16 h. The reaction mixture was then diluted with a saturated aqueous solution of ammonium chloride and extracted with ethyl acetate (3 × 100 mL). The combined organic layers were dried over sodium sulfate, filtered, and concentrated in vacuo. The crude material was absorbed onto a plug of silica gel and purified by chromatography through a Redi-Sep prepacked silica gel column (40 g), eluting with a gradient of 10% to 40% ethyl acetate in hexane. 3-Chloro-2-(3-methoxyoxetan-3-yl)-4-methylpyridine (4.0 g, 18.7 mmol, 93% yield) was obtained as an off-white gum. *m*/*z* (ESI): 214.3 (M + H)^+^. ^1^H NMR (DMSO-*d*_6_, 400 MHz): δ ppm 8.43 (d, *J* = 4.8 Hz, 1 H), 7.47 (dd, *J* = 4.9, 0.9 Hz, 1 H), 5.09 (dd, *J* = 7.4, 1.0 Hz, 2 H), 4.76 (dd, *J* = 7.4, 1.0 Hz, 2 H), 2.98 (s, 3 H), 2.40 (s, 3 H).

#### Step 3. *tert*-Butyl 2-(3-Methoxyoxetan-3-yl)-4-Methyl-3′,6′-Dihydro-[3,4′-Bipyridine]-1′(2′*H*)-Carboxylate

A mixture of 3-chloro-2-(3-methoxyoxetan-3-yl)-4-methylpyridine (4 g, 18.7 mmol), *tert*-butyl 4-(4,4,5,5-tetramethyl-1,3,2-dioxaborolan-2-yl)-3,6-dihydropyridine-1­(2H)-carboxylate (6.95 g, 22.5 mmol; Enamine Ltd.), and K_2_CO_3_ (7.76 g, 56.2 mmol) in 1,4-dioxane (30 mL)/water (10 mL) was sparged with nitrogen for 5 min. S-Phos Pd G3 (0.730 g, 0.936 mmol) was added at room temperature and sparging with nitrogen was continued for 5 min. The resulting mixture was stirred at 100 °C for 16 h. The reaction mixture was then diluted with water (50 mL) and extracted with ethyl acetate (3 × 100 mL). The combined organic layers were dried over sodium sulfate, filtered, and concentrated in vacuo. The crude material was absorbed onto a plug of silica gel and purified by chromatography through a Redi-Sep prepacked silica gel column (40 g), eluting with a gradient of 25% to 50% ethyl acetate in hexane. *tert*-Butyl 2-(3-methoxyoxetan-3-yl)-4-methyl-3′,6′-dihydro-[3,4′-bipyridine]-1′(2′H)-carboxylate (4.9 g, 13.6 mmol, 73% yield) was obtained as a pale-yellow solid. *m*/*z* (ESI): 360.3 (M + H)^+^. ^1^H NMR (DMSO-*d*_6_, 400 MHz): δ ppm 8.35 (d, *J* = 5.0 Hz, 1 H), 7.28 (d, *J* = 5.0 Hz, 1 H), 5.50 (s, 1 H), 5.25 (d, *J* = 7.3 Hz, 1 H), 4.86 (d, *J* = 7.3 Hz, 1 H), 4.58 (d, *J* = 7.3 Hz, 1 H), 4.48 (d, *J* = 7.4 Hz, 1 H), 3.93 (s, 2 H), 3.50 (s, 2 H), 2.94 (s, 3 H), 2.28 (d, *J* = 17.2 Hz, 1 H), 2.21 (s, 3 H), 2.17 (s, 1 H), 1.44 (s, 9 H).

#### Step 4. *tert*-Butyl 4-(2-(3-Methoxyoxetan-3-yl)-4-Methylpyridin-3-yl)­Piperidine-1-Carboxylate

To a degassed solution of *tert*-butyl 2-(3-methoxyoxetan-3-yl)-4-methyl-3′,6′-dihydro-[3,4′-bipyridine]-1′(2′*H*)-carboxylate (6.50 g, 18.0 mmol) in methanol (130 mL), Pd/C (10 wt % of Pd) (1.92 g, 1.80 mmol) and Pd­(OH)_2_ (20 wt % of Pd) (1.27 g, 1.80 mmol) were added under nitrogen atmosphere. The resulting mixture was degassed and stirred under H_2_ atmosphere (1 atm) at ambient temperature for 5 days. The reaction mixture was then filtered through a pad of Celite and washed with methanol (100 mL). The filtrate and washes were combined and concentrated under reduced pressure. The resulting concentrate was purified by column chromatography using a gradient of 15 to 20% ethyl acetate in petroleum ether. *tert*-Butyl 4-(2-(3-methoxyoxetan-3-yl)-4-methylpyridin-3-yl)­piperidine-1-carboxylate (3.00 g, 8.28 mmol, 46% yield) was obtained as a white solid. *m*/*z* (ESI): 363.1 (M + H)^+^. ^1^H NMR (DMSO-*d*_6_, 400 MHz): δ ppm 8.28 (d, *J* = 4.9 Hz, 1 H), 7.18 (d, *J* = 5.0 Hz, 1 H), 5.11 (d, *J* = 7.1 Hz, 2 H), 4.79 (d, *J* = 7.1 Hz, 2 H), 4.05 (d, *J* = 13.1 Hz, 2 H), 2.93 (s, 3 H), 2.73 (s, 1 H), 2.56–2.66 (m, 1 H), 2.42 (s, 3 H), 1.93 (qd, *J* = 12.6, 4.3 Hz, 2 H), 1.46–1.55 (m, 2 H), 1.42 (s, 9 H).

#### Step 5. 4-(3-Methoxyoxetan-3-yl)-2-Methyl-3-(Piperidin-4-yl)­Pyridine

TFA (3.43 mL, 44.6 mmol) was added to a stirred solution of *tert*-butyl 4-(2-(3-methoxyoxetan-3-yl)-4-methylpyridin-3-yl)­piperidine-1-carboxylate (1.9 g, 4.46 mmol) in DCM (19 mL) at 0 °C, and the resulting mixture was stirred at room temperature for 3 h. The reaction mixture was then concentrated under reduced pressure and azeotropically dried with toluene (10 mL). Crude 4-(3-methoxyoxetan-3-yl)-2-methyl-3-(piperidin-4-yl)­pyridine was obtained and used in the next step without further purification, assuming quantitative yield. *m*/*z* (ESI): 263.1 (M + H)^+^.

#### Step 6. *tert*-Butyl 4-((2*R*,3*R*)-1-(6-(4-(2-(3-Methoxyoxetan-3-yl)-4-Methylpyridin-3-yl)­Piperidin-1-yl)-2-(Trifluoromethyl)­Pyrimidin-4-yl)-2-Methylazetidin-3-yl)­Piperazine-1-Carboxylate

To a stirred solution of *tert*-butyl 4-((2*R*,3*R*)-1-(6-chloro-2-(trifluoromethyl)­pyrimidin-4-yl)-2-methylazetidin-3-yl)­piperazine-1-carboxylate (2.2 g, 5.05 mmol) in DMA (11 mL) was added crude 4-(3-methoxyoxetan-3-yl)-2-methyl-3-(piperidin-4-yl)­pyridine and DIPEA (4.41 mL, 25.2 mmol). The resulting mixture was stirred at 100 °C for 16 h. The reaction mixture was then diluted with ice-cold water and extracted with ethyl acetate (2 × 50 mL). The combined organic layers were washed with brine (50 mL), dried over sodium sulfate, filtered, and concentrated under reduced pressure. The crude material was purified on silica gel, eluting with a gradient of 20% to 35% ethyl acetate in petroleum ether. *tert*-Butyl 4-((2*R*,3*R*)-1-(6-(4-(2-(3-methoxyoxetan-3-yl)-4-methylpyridin-3-yl)­piperidin-1-yl)-2-(trifluoromethyl)­pyrimidin-4-yl)-2-methylazetidin-3-yl)­piperazine-1-carboxylate (2.2 g, 3.32 mmol, 66% yield) was obtained as a light-yellow semi-solid. *m*/*z* (ESI): 662.3 (M + H)^+^.

#### Step 7. 4-(4-(2-(3-Methoxyoxetan-3-yl)-4-Methylpyridin-3-yl)­Piperidin-1-yl)-6-((2*R*,3*R*)-2-Methyl-3-(Piperazin-1-yl)­Azetidin-1-yl)-2-(Trifluoromethyl)­Pyrimidine

TFA (1.0 mL, 13.0 mmol) was added to a solution of *tert*-butyl 4-((2*R*,3*R*)-1-(6-(4-(2-(3-methoxyoxetan-3-yl)-4-methylpyridin-3-yl)­piperidin-1-yl)-2-(trifluoromethyl)­pyrimidin-4-yl)-2-methylazetidin-3-yl)­piperazine-1-carboxylate (2.2 g, 3.32 mmol) in DCM (4 mL), and the resulting mixture was stirred at room temperature for 1 h. The reaction mixture was then concentrated, and crude 4-(4-(2-(3-methoxyoxetan-3-yl)-4-methylpyridin-3-yl)­piperidin-1-yl)-6-((2*R*,3*R*)-2-methyl-3-(piperazin-1-yl)­azetidin-1-yl)-2-(trifluoromethyl)­pyrimidine was obtained as a yellow gum, which was used in the following step without further purification, assuming quantitative yield.

#### Step 8. 1-(4-((2*R*,3*R*)-1-(6-(4-(2-(3-Methoxyoxetan-3-yl)-4-Methylpyridin-3-yl)­Piperidin-1-yl)-2-(Trifluoromethyl)­Pyrimidin-4-yl)-2-Methylazetidin-3-yl)­Piperazin-1-yl)­Prop-2-en-1-one

To a stirred solution of crude 4-(4-(2-(3-methoxyoxetan-3-yl)-4-methylpyridin-3-yl)­piperidin-1-yl)-6-((2*R*,3*R*)-2-methyl-3-(piperazin-1-yl)­azetidin-1-yl)-2-(trifluoromethyl) in DCM (18 mL), DIPEA (2.39 mL, 13.7 mmol) was added at 0 °C, followed by acryloyl chloride (0.221 mL, 2.73 mmol). The resulting mixture was stirred at 0 °C for 10 min. The reaction mixture was then diluted with ice-cold water (50 mL) and extracted with DCM (2 × 100 mL). The combined organic layers were washed with brine (100 mL), dried over sodium sulfate, filtered, and concentrated under reduced pressure. The crude material was purified by prep-HPLC (X-Bridge C18, 250 mm × 19 mm, 5.0 μm column) eluting with a gradient of 30% to 100% acetonitrile (0.1% formic acid) in water (0.1% formic acid) (15 mL/min). 1-(4-((2*R*,3*R*)-1-(6-(4-(2-(3-methoxyoxetan-3-yl)-4-methylpyridin-3-yl)­piperidin-1-yl)-2-(trifluoromethyl)­pyrimidin-4-yl)-2-methylazetidin-3-yl)­piperazin-1-yl)­prop-2-en-1-one (1.25 g, 2.03 mmol, 61% yield) was obtained as a white solid. *m*/*z* (ESI): 616.3 (M + H)^+^. ^1^H NMR (400 MHz, Methanol-*d*_4_) δ 8.26 (d, *J* = 4.9 Hz, 1H), 7.16 (d, *J* = 5.0 Hz, 1H), 6.76 (dd, J = 16.9, 10.7 Hz, 1H), 6.20 (dd, *J* = 16.8, 1.9 Hz, 1H), 5.78–5.72 (m, 1H), 5.51 (s, 1H), 5.27 (d, *J* = 7.2 Hz, 2H), 4.94–4.89 (m, 2H), 4.64–4.48 (m, 3H), 4.07–3.87 (m, 3H), 3.77–3.63 (m, 4H), 3.36–3.32 (m, 1H), 3.05 (s, 3H), 2.98–2.78 (m, 3H), 2.50–2.33 (m, 7H), 2.17 (qd, *J* = 12.7, 3.5 Hz, 2H), 1.66 (br d, *J* = 11.1 Hz, 2H), 1.49 (d, *J* = 6.4 Hz, 3H). HRMS *m*/*z* (ESI): calcd for C_31_H_40_F_3_N_7_O_3_ (M + H)^+^: 616.3217, found: 616.3215.

## Supplementary Material





## Notes

Description of assays and studies, synthetic procedures for compounds **1**–**28**, ^1^H NMR data for compounds **1**–**28**, HPLC traces for compounds used in animal studies, activity data for compounds referred to in the article or used for stereochemical assignment, X-ray crystallographic data for compounds **3**, **4**, **5**, and **AM-8719**, and KINOME*scan* data for compound **15** (PDF)
Molecular formula strings (SMILES) for compounds **1**–**28** and associated assay data (CSV)

## Abbreviations Used

ADME: absorption, distribution, metabolism, and excretion
BBB: blood–brain barrier
CE: coupled nucleotide exchange
CNS: central nervous system
CTG: CellTiter-Glo
DCM: dichloromethane
DEL: DNA-encoded library
DIPEA: diisopropylethylamine
DMA: *N*,*N*-dimethylacetamide
DMSO: dimethyl sulfoxide
EGF: epidermal growth factor
ER: efflux ratio
ERK: extracellular signal regulated kinase
ESI: electrospray ionization
GAP: GTPase-activating protein
GDP: guanosine diphosphate
GEF: guanine nucleotide exchange factor
GSH: glutathione
GTP: guanosine-5′-triphosphate
G12C: glycine-to-cysteine substitution at codon 12
G12D: glycine-to-aspartate substitution at codon 12
HBD: number of hydrogen bond donors
hMDR1: human multidrug resistance gene 1
HPMC: hydroxypropyl methylcellulose
HRMS: high-resolution mass spectrometry
iv: intravenous
KRAS: Kirsten Rat Sarcoma
MAPK: mitogen-activated protein kinase
MDCK: Madin–Darby canine kidney
MIA PaCa-2 T2: Miami pancreatic carcinoma clone number 2 tumor generation 2
MPO: multiparameter optimization
MS: mass spectrometry
MW: molecular weight
*n*BuLi: *n*-butyllithium
NMR: nuclear magnetic resonance
NF: Neurofibromin
NSCLC: nonsmall cell lung cancer
PDB: protein data bank
PEG: polyethylene glycol
PK: pharmacokinetic
p.o.: per os
QD: quaque diem
RAF: rapidly accelerated fibrosarcoma
RT: room temperature
RTK: receptor tyrosine kinase
SEM: standard error of the mean
S_N_Ar: nucleophilic aromatic substitution
SOS: son of sevenless
S-Phos Pd G3: (2-dicyclohexylphosphino-2′,6′-dimethoxybiphenyl)­[2-(2′-amino-1,1′-biphenyl)]­palladium­(II) methanesulfonate
Sw-II: switch-II
TFA: trifluoroacetic acid
TGI: tumor growth inhibition
THF: tetrahydrofuran
TPSA: topological polar surface area
wt %: weight percent
Tween 80: polyethylene sorbitol ester
UC: ultracentrifugation
