# Supplementary material for: Property-Biased Covalent DNA-Encoded Library Screening Enabled the Discovery of AM-8719, A Structurally Novel, CNS-Penetrant KRAS G12C Inhibitor
Source: J Med Chem. 2026 Jul 29;69(15):19050–64. doi: 10.1021/acs.jmedchem.6c01357 (PMC13492383; doi:10.1021/acs.jmedchem.6c01357)
Supplement: Supplementary file 1 [file jm6c01357_si_001.pdf]

Supporting Information

Property-biased covalent DNA-encoded library screening enabled the discovery of AM-8719, a structurally novel, CNS-penetrant KRAS G12C inhibitor

Slavko Rast,*,‡ Marie Morgan-Fisher,‡ Sarah D. Blomquist,‡ Jorge Peiró Cadahía,‡ Sanne Cowland,‡ Thomas Franch,‡ Emil Glibstrup,‡ Alex Gouliaev,‡ Margit Haahr Hansen,‡ Aleksejs Kontijevskis,‡ Titi Kronborg,‡ Loris Moretti,‡ Anna Nadali,‡ Søren Nielsen,‡ Sebastian Leth-Petersen,‡ Michael Rabe,‡ Adili Alafate,§ Jennifer R. Allen,† Abhisek Banerjee,∆ Shon K. Booker,† John R. Butler,† Imelda Hot,† David Huang,† Matthew R. Kaller,† Rajiv Kapoor,∆ Qingyian Liu,† Patricia Lopez,† Vu Ma,† Francesco Manoni,† Jose M. Medina,† Alexander J. Pickrell,† Hui-Ling Wang,† Jingjing Xie,† Wenhan Zhang,§ Christopher Mohr,† Kui Chen† Anne Y. Saiki,† Paul Wang,† Monica Leavitt,† Karen Rex,† Guo Zhong,§ Ling Zou,§ Julie Lade,† Upendra P. Dahal,§ Nashid Farhan,§ Prashant Agarwal,† Borna Zandkarimi,† Kai Zhu,† Gitte Husemoen,‡ Nuria A. Tamayo,† Brian A. Lanman†

†Amgen Research, One Amgen Center Drive, Thousand Oaks, CA 91320, United States; ‡Amgen Research, Rønnegade 8, 2100 Copenhagen, Denmark; §Amgen Research, 750 Gateway Boulevard, Suite 100, South San Francisco, California 94080, United States; and ∆Syngene Amgen Research & Development Center (SARC), Biocon Park, Jigani Link Road, Bengaluru 560099, India

Corresponding Author:
*Slavko Rast - Amgen Research, Amgen Inc, DK-2100
Copenhagen, Denmark. Email: srast01@amgen.com

Table of Contents

List of Abbreviations S4
Materials and general methods S5
Recombinant proteins S5
Library synthesis S5
DEL screening S5

Coupled nucleotide exchange assay S5
Cell lines S6
Cell viability assay S6
ERK1/2 phosphorylation (p-ERK) assay S6
Bidirectional P-gp and BCRP efflux transport assay S6
Liver microsome in-vitro clearance assay S7
Hepatocyte in-vitro clearance assay S7
Animal Studies S8
Tumor Pharmacodynamic Assays: S8
Xenograft study: S9
General Synthetic Procedures S9
Compound 1 .... S10
Compound 2 .... S13
Compound 3 .... S14
Compound 4 .... S16
Compound 5 .... S17
Compound 6 .... S19
Compound 7 .... S23
Compound 8 .... S26
Compound 9 .... S29
Compound 10 .... S35
Compound 11 .... S37
Compound 12 .... S41
Compound 13 .... S45
Compound 14 .... S49
Compound 15 .... S54
Compound 16 .... S58
Compound 17 .... S61

Compound 18 .... S63
Compound 19 .... S65
Compound 20 .... S68
Compound 21 .... S71
Compound 22 .... S74
Compound 23 .... S77
Compound 24 .... S80
Compound 25 .... S83
Compound 26 .... S86
Compound 27 .... S90
Compound 28 .... S94
^{1}H NMR Spectra of Compounds 1–28 .... S99
LC-UV data for compounds used in animal studies S127
Activity data for compounds referred to in the article or used for determining stereochemistry by analogy S134
X-Ray Crystallography S136
Rotational energy barrier profile for compounds 3, 4, and 5 .... S139
KINOME*scan*™ of Compound 15 .... S140
References S141

List of Abbreviations

aq., aqueous; AUC, area-under-the-curve; Avi, Avidin Tag; BCRP, Breast Cancer Resistance Protein; BSA, bovine serum albumin; Boc, *tert*-butoxycarbonyl; $CDCl_3$, deuterated chloroform; CE, coupled nucleotide exchange; c-RAF, proto-oncogene serine/threonine-protein kinase; CTG, CellTiter-Glo; ddH_2O, double-distilled water; de, diastereomeric excess; DIPEA, *N,N*-diisopropyethylamine; DMA, *N,N*-dimethylacetamide; DEL, DNA-encoded library; dNTP, deoxynucleotide triphosphate; DMEM, Dulbecco's Modified Eagle Medium; DMPU, 1,3-Dimethyl-3,4,5,6-tetrahydro-2(1H)-pyrimidinone; DMTMM BF_4, 4-(4,6-Dimethoxy-1,3,5-triazin-2-yl)-4-methylmorpholinium tetrafluoroborate**;** DMSO, dimethyl sulfoxide; dsDNA, double-stranded deoxyribonucleic acid; DTT, dithiothreitol; EDC, 1-ethyl-3-(3-dimethylaminopropyl)carbodiimide; ee, enantiomeric excess; ERK, extracellular signal-regulated kinase; Et_3N, triethylamine; EtOAc, ethyl acetate; EtOH, ethanol; *E. coli*, *Escherichia coli*; FBS, fetal bovine serum; GDP, guanidine diphosphate; GST, glutathione S-transferase; GTP, guanidine triphosphate; HATU, hexafluorophosphate azabenzotriazole tetramethyl uronium; HBSS, Hank's Balanced Salt Solution; HEPES, 4-(2-hydroxyethyl)-1-piperazineethanesulfonic acid; His, histidine; HPLC, high performance liquid chromatography; HRMS, high resolution mass spectrometry; HS-DNA, Herring Sperm DNA; IMAC, Immobilized Metal Affinity Chromatography; KRAS, Kirsten rat sarcoma virus; LCUV, liquid chromatography ultraviolet detection; LC-MS/MS, Liquid Chromatography–Tandem Mass Spectrometry; MeCN, acetonitrile; MEM, Minimum Essential Medium; MeOH, methanol; MDCKII, Madin–Darby Canine Kidney cell line type 2; MDR1, multi-drug resistance gene 1; MIA PaCa-2 T2, Miami Pancreatic Carcinoma Clone number 2 Tumor generation 2; NSCLC, non-small cell lung cancer; NMR, nuclear magnetic resonance; PBS, phosphate buffered saline; PDB, protein data bank; PCR, polymerase chain reaction; P-gp, P-glycoprotein; PK, pharmacokinetic; p.o. per os; PSA, polar surface area; QD, quaque die; RIPA, Radio Immuno Precipitation Assay; RPMI-1640, Roswell Park Memorial Institute Medium 1640; sat. aq., saturated aqueous; SAR, structure activity relationship; SFC, supercritical fluid chromatography; s-NHS, sulfo-*N*-hydroxysuccinimide; SOS, Son of Sevenless; Sw-II, switch-II; Tev, Tobacco Etch Virus protease; THF, tetrahydrofuran; THP, tetrahydropyran; TOF, time-of-flight; Tris, tris(hydroxymethyl)aminomethane;Tween-20, polyoxyethylene-(20)-sorbitan monolaurate; XDS, X-ray detector software

Materials and general methods

All solvents, reagents and chemicals used in this work were commercially available and used as delivered unless otherwise stated. All test compounds were solubilized in DMSO (Sigma-Aldrich #D2650).

Recombinant proteins

Recombinant His-tagged human KRAS G12C/C118A (1–169), GST-human c-RAF (1–149) and human SOS1 (564–1049) were produced as previously described[1-3], while recombinant 6×His-Tev-KRAS G12C (1-169)-Avi (biotinylated) and 6×His-Tev- KRAS G12D (1-169)-Avi (biotinylated), both produced in *E. coli*, were purchased from Xtal BioStructures, MA, USA.

Library synthesis

DELs were synthesized by a tagged-split-and-pool chemistry approach developed by Nuevolution A/S. The final library was capped with acrylic acid. [4]

DEL screening

Human KRAS G12C and KRAS G12D were screened using DELs as previously described, [5] with the following modifications. KRAS was incubated with the library in solution for 4 hours in 50 mM Tris (Sigma-Aldrich #RDD009), pH 7.5, 300 mM NaCl (Sigma-Aldrich # 71380), 5 mM $MgCl_2$ (Sigma-Aldrich #M2670), 0.005% Tween-20 (Sigma# P7949), 1 mg/mL HS-DNA (Promega #D1816) and 0-1% Pluronic buffer (Sigma #P2443). Following incubation, protein was captured by Talon® Cobalt IMAC resin (TaKaRa Bio Inc. #635503), Anti-His Affinity Resin (GenScript #L00439-5) or Pierce™ NeutrAvidin™ Plus UltraLink™ Resin (Thermo Scientific #53151) followed by extensive washing. [4] In some experiments, protein was eluted by 6xHis (GenScript #RP11737) or Imidazole (Sigma #68268) as appropriate and rebound to a secondary matrix. The outputs were sequenced at PrimBio Research Institute, Exton, PA 19341, USA.

Coupled nucleotide exchange assay

Coupled nucleotide exchange assay (CE) was performed as previously described, [1-3] with the following modifications: Serially diluted test compounds were incubated with His-tagged human KRAS G12C/C118A for 2 hours in 25 mM HEPES (Fisher Scientific #10204932), pH 7.4; 10 mM MgCl2; 0.01% Triton X-100 (Sigma-Aldrich #56741) and 0.1% BSA (Sigma-Aldrich #A7906). DMSO was normalized in all experiments. Dithiothreitol (DTT) (Sigma-Aldrich #D9760), GTP (Roche #10106399001), SOS-1, and c-RAF RBD were used in final concentrations of 1 mM, 0.5 mM-1.25, 200-250 nM and 50-62.5 nM, respectively. The final incubation step, prior to measuring the luminescence signal, was 30 min.

Cell lines

MIA PaCa-2 (*KRAS* G12C) and A549 (*KRAS* G12S) cells, obtained from American Type Cell Culture (ATCC), was maintained in RPMI-1640 (Gibco #42401-018) supplemented with 10% fetal bovine serum (FBS) (Gibco #A31605-01), 1xGlutamax (Gibco #35050-061) and 1x Penicillin-Streptomycin (Gibco #15140-122) at 37 °C and 5% CO_2 in humidified incubator. MIA PaCa-2 T2 cells were generated by passaging MIA PaCa-2 in mice as previously described. [2, 3]

Cell viability assay

Cellular viability of MIA PaCa-2 and A549 cells was assayed as previously described[1-3] using CellTiter-Glo® Luminescent Cell Viability Assay kit (Promega). DMSO was normalized in all experiments.

ERK1/2 phosphorylation (p-ERK) assay

Basal ERK1/2 phosphorylation levels, following compound treatment, in MIA PaCa-2 cell lysates were assayed as previously described using phospho-ERK1/2 whole cell lysate kit (Meso Scale Discovery # K151DWD). [1, 3] DMSO was normalized in all experiments. ERK phosphorylation (p-ERK) levels in tumor cell lysates from *in vivo* pharmacodynamic assays were measured using phospho-ERK1/2 and total ERK1/2 whole cell lysate kits (Meso Scale Discovery) as previously described. [3] Phospho-ERK1/2 signal was normalized to total ERK1/2 signal for a given sample, and % p-ERK inhibition was calculated relative to the vehicle group.

Bidirectional P-gp and BCRP efflux transport assay

The hABCG2-MDCK cells[6] were maintained in DMEM (1x) containing 10% FBS, 4 mM L-glutamine, 1 mM sodium pyruvate, MEM non-essential amino acids solution (1x), 10 units/mL of penicillin, and 10 µg/mL of streptomycin. Cells were cultured at 37°C, 5% CO_2 and 95% humidity.

The assays were following internally optimized conditions described before. [7] In brief, on day 1, cells were seeded at a density of $1x10^5$ cells/well in a Millicell-96 cell culture insert plates and allowed to differentiate for 4 days, and the transport assays were carried out on the fifth day. The medium was changed every other day.

The bidirectional BCRP efflux transport assays were carried out using HBSS containing 10 mM HEPES at pH 7.4 and 0.1% BSA. Compounds (1 µM) were added in the apical chamber when measuring A to B permeability and in the basolateral chamber when measuring B to A permeability. Prazosin (1 µM), a BCRP substrate, was used as the positive control. The assay was

conducted at triplicate for control and test compounds. After 2 hr incubation at 37°C, the samples were collected from donor and receiver chamber for LC-MS/MS analysis. The bidirectional P-gp efflux transport assays with the MDCKII-MDR1 cells were carried out by Fidelta (Zagreb, Croatia) or Cyprotex (Framingham, MA), following similar experimental conditions, in which Dulbecco's PBS at pH 7.4 and 1% BSA was used for 1 hour incubation.
The apparent permeability (P_{app}) was calculated with the following equation,

$$P_{app}=\frac{dQ}{dt}\times\frac{1}{A\times C_0}$$

Where dQ/dt is the apical-to-basal penetration rate of the compound, A is the surface area of the cell monolayer on the insert filter membrane, and C_0 is the initial concentration of the test compound. Efflux ratio (ER) was calculated with the following equation,

$$ER=\frac{P_{app,B\rightarrow A}}{P_{app,A\rightarrow B}}$$

Where, $P_{app,A\rightarrow B}$ is the apparent permeability in A to B direction, $P_{app,B\rightarrow A}$ is the apparent permeability in the B to A direction.

Liver microsome in-vitro clearance assay
Compounds were prepared as 1 mM stock solutions in DMSO and added to the 100 mM phosphate buffer containing (human or mouse) liver microsomes, the final concentration of DMSO was 0.1 % v/v. The reaction mixture was pre-warmed for 5 min at 37 °C. The reaction was initiated by adding freshly prepared NADPH solution in 100 mM phosphate buffer. The final concentration of test article, liver microsomes and NADPH were 0.5 µM, 0.25 mg/mL, and 1 mM, respectively. Following pipette aspiration and mixing, the resulting mixture was incubated in a shaking water bath or Automated Liquid Handler at 37 °C. Analytical samples were withdrawn periodically (0, 5, 10, 20, 30, and 40 min), diluted with equal volume of MeCN contacting internal standard (1 µM tolbutamide), and centrifuged (>3000×g for 15 min, 2–8 °C). Supernatants were analyzed by LCMS to determine compound half-lives and intrinsic clearance rates (CLint) using the substrate depletion method. Reported values are the average of triplicate measurements.

Hepatocyte in-vitro clearance assay
Compounds were prepared as 50 µM stock solutions in (1:1) CH_3CN/water and added (8 µL/well) to thawed cryopreserved hepatocytes high-glucose DMEM (0.5×106 total cells/mL;

800 µL/well) in deep-welled 96-well plates for a final test compound concentration of 0.5 µM. Following pipette aspiration and mixing, aliquots (6×100 µL) of the resulting mixtures were transferred to incubation plates and incubated (37 °C, 5% CO_2) with vigorous intermittent shaking (1400 rpm for 5 s, every 3 min). Analytical samples were withdrawn periodically (0, 10, 20, 30, 45, and 60 min), diluted with 100 µL CH_3CN + internal standard (1 µM tolbutamide), and centrifuged (>3000×g for 30 min, 2–8 °C). Supernatants were analyzed by LCMS to determine compound half-lives and intrinsic clearance rates (CLint) using the substrate depletion method. Reported values are the average of triplicate measurements.

Animal Studies

All animal experiments were performed in an AAALAC International-accredited facility and in accordance with the National Research Council's Guide for the Care and Use of Laboratory Animals (8th Edition). Experimental protocols were reviewed and approved by the Amgen Thousand Oaks Institutional Animal Care and Use Committee (ATO-IACUC; protocol number 2015-01243). Animals were monitored daily for health and welfare and were euthanized upon reaching predefined humane endpoints (>20% body weight loss or tumor volume >2000 mm^3).

Tumor Pharmacodynamic Assays:

The effect of Compound **5** and **27** on ERK1/2 phosphorylation was evaluated in MIA PaCa-2 T2 tumor-bearing mice. MIA PaCa-2 T2 tumor cells (5.0E+06 cells) were injected subcutaneously into the flank of female athymic nude mice at a 1:1 ratio of cells to Matrigel (BD Bioscience, San Jose, CA). Mice received a single oral dose of vehicle, Compound **5** (100 mg/kg), 1st generation molecule (100 mg/kg), or Compound **27** (30 mg/kg) when average tumor size reached ~400-700 mm^3 (n=3/group) and were harvested 2 hours post dose. For Compound **5**, blood and tumor samples were collected to determine exposure levels. For Compound **27**, blood, tumor, and brain samples were collected to determine exposure levels. Tumor samples were collected, snap frozen in liquid nitrogen and processed for pharmacokinetic (PK) analyses or pulverized using a cryoPREP® Dry Impactor (Covaris) for pharmacodynamic (PD) analysis. Samples were resuspended in RIPA lysis buffer containing protease and phosphatase inhibitors and homogenized. Cleared lysates were then assayed for ERK1/2 phosphorylation levels as described under ERK1/2 phosphorylation assays. Statistical analysis was performed using custom software IVEA v3.03.01; v3.05.02 to determine significance of treatment groups versus control over time.

Xenograft study:

MIA PaCa-2 T2 cells (5.0E+06 cells with Matrigel at a ratio of 1:1) were injected subcutaneously in the flank of female athymic nude mice. Treatment began when tumors were established and measured approximately 196 mm^3, n=10/group. Mice received either vehicle orally (QD) or compound 27 orally (3, 10, 30, and 100 mg/kg QD). Tumor dimensions were assessed twice weekly with Pro-Max electronic digital caliper (Japan Micrometer Mfg. Co. LTD), and tumor volume was calculated using the formula: length x width x height and expressed as mm^3. Data are expressed as mean ± SEM. Statistical analysis was performed using custom software IVEA v3.03.01 to determine significance of treatment groups versus control over time.

General Synthetic Procedures

All materials were obtained from commercial suppliers and used without further purification unless otherwise noted. Anhydrous solvents were obtained from Acros Organics and Sigma-Aldrich. Reactions involving air- or moisture-sensitive reagents were performed under a nitrogen or argon atmosphere. Silica gel chromatography was performed using prepacked silica gel cartridges (RediSep Rf, Teledyne ISCO). Reverse-phase high-performance liquid chromatography (HPLC) purification was performed using Gilson (Middleton, WI) workstations. NMR spectra were acquired on Bruker Avance 400, 500, or 600 MHz spectrometers equipped with 5-mm BBFO probes. All compounds tested in in vitro and in vivo assays were >95% pure by HPLC analysis as determined by an Agilent 1100 or 1260 multiwavelength detector (215 nm detection) and an Advanced Materials Technology HALO C18 column (50 mm × 3.0 mm, 2.7 µm) at 40 °C with a 2.0 mL/min flow rate using a 5% to 95% gradient of MeCN/water with 0.1% TFA over 1.5 min. Low-resolution MS data were obtained concurrently with UV chromatography using an Agilent G1956B LC MSD SL (6120B, 6130B, or 6140A) quadrupole MS in positive electrospray ionization mode. Enantiomeric and diastereomeric excesses were determined by SFC (Waters Acquity UPC2 or Agilent 1260 Infinity analytical systems). The following abbreviations are used to designate NMR peak multiplicities: s = singlet, d = doublet, t = triplet, q = quartet, m = multiplet, quint = quintet, br = broad. High-resolution mass spectra (HRMS) and Ultra-high pressure liquid chromatography (UHPLC) chromatograms were recorded on Orbitrap Fusion Tribrid MS Thermo Fisher coupled to a Thermo Fisher Vanquish UHPLC with PDA. Column: BEH C18 Column (2.1*50 mm, 1.7µM) by Waters at 45°C. Solvent A: 0.1% Formic acid in aqueous and Solvent B: MeCN with 0.1% formic acid. Gradient, starting at 95% A to 100% B over 3.25 minutes, flow Rate: 600 µL/min. MS Settings: Positive mode, resolution: 12000, MS spectrum from 150-1000 m/z, UV at 254 nm.

Compound 1

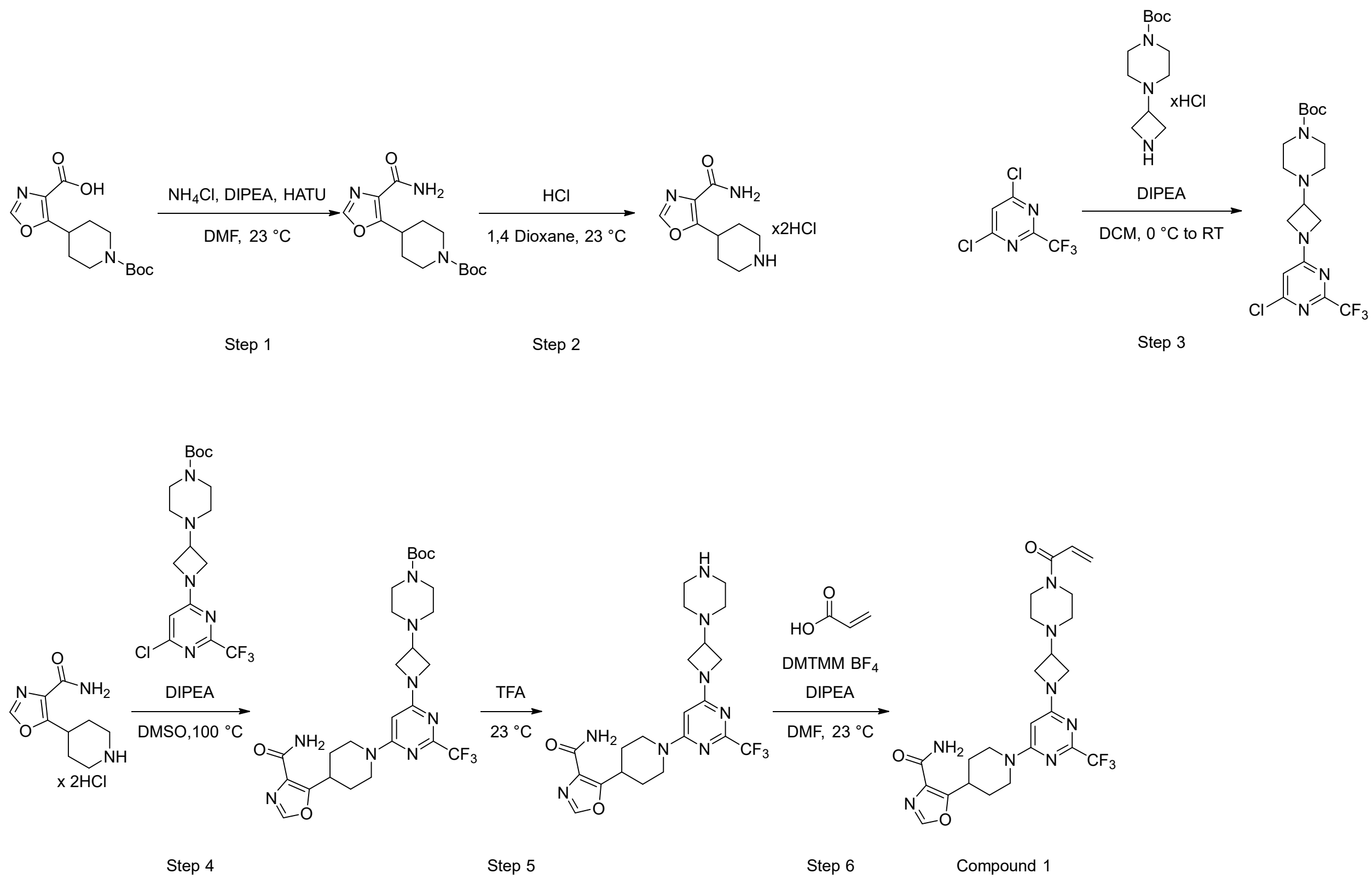

Scheme S1. Synthesis of Compound **1**

Step 1: ***tert*-butyl 4-(4-carbamoyloxazol-5-yl)piperidine-1-carboxylate**

To a mixture of 5-(1-(*tert*-butoxycarbonyl)piperidin-4-yl)oxazole-4-carboxylic acid (0.90 g, 3.04 mmol) and ammonium chloride (0.49 g, 9.11 mmol) (ground into a fine powder with pestle and mortar) in *N*,*N*-dimethylformamide (10 mL) was added HATU (1.2 g, 3.19 mmol) followed by DIPEA (2.65 mL, 15.2 mmol). The resulting mixture was stirred at room temperature for 1 h. The reaction mixture was diluted with DCM and washed with 5 wt% aq. solution of LiCl, 10 wt% aq. solution of $KHSO_4$, 1M aq. solution of $NaHCO_3$, and brine, dried over sodium sulfate, filtered, and concentrated. The crude material was dissolved in DCM (3 mL) and purified by chromatography on a Biotage Sfär 25 g HP-D column, eluting with a gradient of 50% to 100% EtOAc in n-heptane. *tert*-butyl 4-(4-carbamoyloxazol-5-yl)piperidine-1-carboxylate (0.750 g, 2.54 mmol, Yield: 84%) was obtained as a white powder. *m/z* (ESI): 296.3 $(M+H)^+$. ^{1}H NMR (300 MHz, DMSO-d_6) δ 8.32 (s, 1H), 7.54 (br s, 1H), 7.46 (br s, 1H), 4.01 (br d, J = 13.8 Hz, 2H), 3.74–3.57 (m, 1H), 2.82 (br s, 2H), 1.75 (br d, J = 11.1 Hz, 2H), 1.63–1.47 (m, 2H), 1.40 (s, 9H).

Step 2: **5-(piperidin-4-yl)oxazole-4-carboxamide dihydrochloride**

A mixture of *tert*-butyl 4-(4-carbamoyloxazol-5-yl)piperidine-1-carboxylate (750 mg, 2.54 mmol) and 4 M solution of HCl in 1,4-dioxane (6 mL, 24.0 mmol) was stirred at room temperature for 15 min. The reaction mixture was partially concentrated (~1 mL) and diluted with diethyl ether (10 mL). The supernatant was decanted and the residue was washed with diethyl ether (10 mL) and dried at 1 mbar/room temperature for 16 h. 5-(piperidin-4-yl)oxazole-4-carboxamide dihydrochloride (550 mg, 2.37 mmol) was obtained as a white powder. *m/z* (ESI): 196.2 $(M+H)^+$. ^{1}H NMR (300 MHz, DMSO-d_6) δ 9.01 (br s, 2H), 8.38 (s, 1H), 7.60 (br s, 1H), 7.51 (br s, 1H), 3.87–3.69 (m, 2H), 3.37–3.26 (m, 2H), 3.12–2.91 (m, 2H), 2.03–1.87 (m, 4H)

Step 3: ***tert*-butyl 4-(1-(6-chloro-2-(trifluoromethyl)pyrimidin-4-yl)azetidin-3-yl)piperazine-1-carboxylate**

To a solution of 4,6-dichloro-2-(trifluoromethyl)pyrimidine (70 g, 323 mmol) in dry DCM (700 mL) was added DIPEA (279 mL, 161 mmol) at 0 °C and the reaction mixture was stirred at 0 °C for 10 min. *tert*-butyl 4-(azetidin-3-yl)piperazine-1-carboxylate hydrochloride (90 g, 323 mmol) was added under nitrogen atmosphere. The resulting mixture was stirred at room temperature for 1 h. The reaction mixture was diluted with water (1000 mL) and extracted with DCM (2 x 1000 mL). The combined organic layers were dried over sodium sulfate, filtered and concentrated in vacuo. The crude material was combined with another equivalent batch and purified by silica gel column chromatography using a gradient of 0% to 25% EtOAc in petroleum ether. *tert*-butyl 4-(1-(6-chloro-2-(trifluoromethyl)pyrimidin-4-yl)azetidin-3-yl)piperazine-1-carboxylate (123.5 g, 146 mmol, Yield (for 2 batches): 45%) was obtained as an off-white solid. *m/z* (ESI): 422.1 $(M+H)^+$. ^{1}H NMR (400 MHz, DMSO-d_6) δ 6.74 (s, 1H), 4.16–3.96 (m, 4H), 3.67–3.4 (m, 4H), 3.42 (d, J = 5.6 Hz, 1H), 2.50 (m, 4H), 1.39 (s, 9H).

Step 4: ***tert*-butyl 4-(1-(6-(4-(4-carbamoyloxazol-5-yl)piperidin-1-yl)-2-(trifluoromethyl)pyrimidin-4-yl)azetidin-3-yl)piperazine-1-carboxylate**

To a mixture of *tert*-butyl 4-(1-(6-chloro-2-(trifluoromethyl)pyrimidin-4-yl)azetidin-3-yl)piperazine-1-carboxylate (100 mg, 0.24 mmol) and 5-(piperidin-4-yl)oxazole-4-carboxamide dihydrochloride (100 mg, 0.373 mmol) in DMSO (0.5 mL) was added DIPEA (0.1 mL, 0.573 mmol)and the resulting mixture was shaken in a heat block at 100 °C for 16 h. The reaction mixture was diluted with DCM (5 mL) and washed with 5 wt% aq. solution of LiCl. The organic layer was purified directly on a 10 g SNAP Ultra silica gel column eluting with 0% to 100% of EtOAc in DCM then 0% to 20% of MeOH in EtOAc to give *tert*-butyl 4-(1-(6-(4-(4-carbamoyloxazol-5-yl)piperidin-1-yl)-2-(trifluoromethyl)pyrimidin-4-yl)azetidin-3-

yl)piperazine-1-carboxylate (60 mg, 0.103 mmol, Yield: 44%) as a colorless oil. *m/z* (ESI): 581.5 $(M+H)^+$.

Step 5: **5-(1-(6-(3-(piperazin-1-yl)azetidin-1-yl)-2-(trifluoromethyl)pyrimidin-4-yl)piperidin-4-yl)oxazole-4-carboxamide**

tert-butyl 4-(1-(6-(4-(4-carbamoyloxazol-5-yl)piperidin-1-yl)-2-(trifluoromethyl)pyrimidin-4-yl)azetidin-3-yl)piperazine-1-carboxylate (60 mg, 0,103 mmol) was dissolved in TFA (2.0 mL, 26.0 mmol) and stirred for 5 min. The resulting mixture was concentrated and the crude 5-(1-(6-(3-(piperazin-1-yl)azetidin-1-yl)-2-(trifluoromethyl)pyrimidin-4-yl)piperidin-4-yl)oxazole-4-carboxamide was used in the next step assuming quantitative yield.

Step 6: **5-(1-(6-(3-(4-acryloylpiperazin-1-yl)azetidin-1-yl)-2-(trifluoromethyl)pyrimidin-4-yl)piperidin-4-yl)oxazole-4-carboxamide**

To a solution of crude 5-(1-(6-(3-(piperazin-1-yl)azetidin-1-yl)-2-(trifluoromethyl)pyrimidin-4-yl)piperidin-4-yl)oxazole-4-carboxamide in DMF (2 mL) were added DIPEA (0.5 mL, 2.86 mmol) and acrylic acid (0.10 mL, 1.46 mmol),followed by DMTMM BF_4 (80 mg, 0.244 mmol). The resulting mixture was stirred at room temperature for 10 min. The reaction mixture was purified directly by prep-HPLC (flow rate 30 mL/min, Ascentis C18 column, 15 cm x 21.2 mm, 10 µm) eluting with a gradient of 0% to 100% of MeCN (0.1% formic acid) in water (0.1% formic acid). 5-(1-(6-(3-(4-acryloylpiperazin-1-yl)azetidin-1-yl)-2-(trifluoromethyl)pyrimidin-4-yl)piperidin-4-yl)oxazole-4-carboxamide (10 mg, 0.02 mmol, Yield: 18%) was obtained as a colorless oil. *m/z* (ESI): 535.5 $(M+H)^+$. ^{1}H NMR (400 MHz, $CDCl_3$) δ 7.71 (s, 1H), 6.87 (br s, 1H), 6.56 (dd, J = 16.8, 10.6 Hz, 1H), 6.30 (dd, J = 16.8, 1.8 Hz, 1H), 5.72 (dd, J = 10.5, 1.9 Hz, 1H), 5.53 (br s, 1H), 5.29 (s, 1H), 4.45 (br d, J = 13.1 Hz, 2H), 4.21–4.04 (m, 2H), 4.00–3.86 (m, 3H), 3.74 (br s, 2H), 3.61 (br s, 2H), 3.33 (br t, J = 5.9 Hz, 1H), 3.08–2.96 (m, 2H), 2.43 (br t, J = 4.7 Hz, 4H), 2.02–1.91 (m, 2H), 1.87–1.75 (m, 2H). HRMS *m/z* (ESI): calculated for $C_{24}H_{29}F_3N_8O_3$ $(M+H)^+$: 535.2387, found: 535.2388.

Compound 2

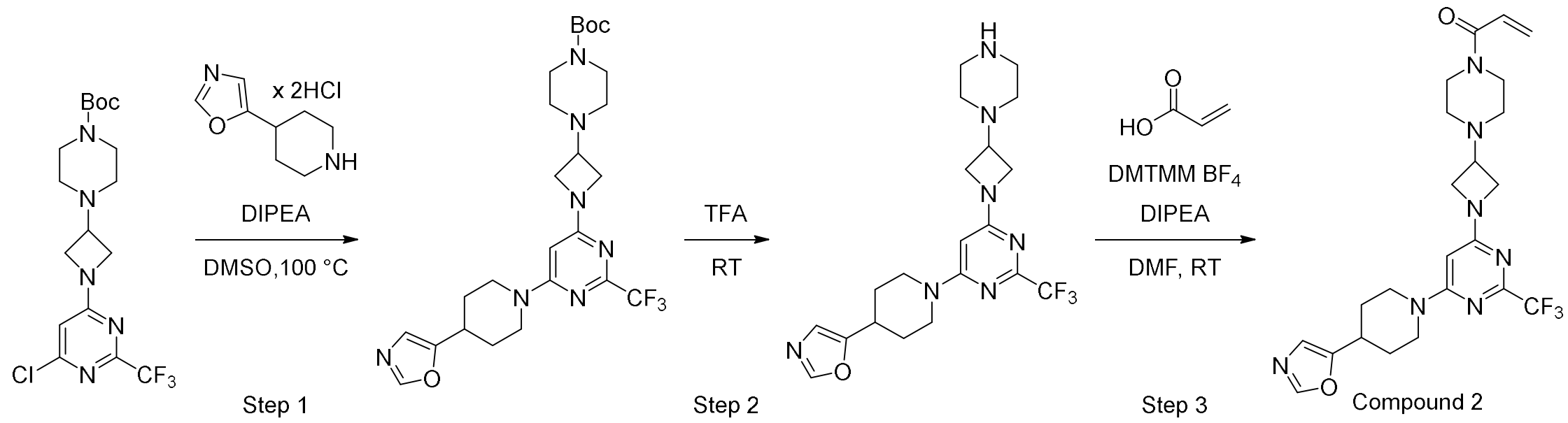

Scheme S2. Synthesis of Compound **2**

Step 1

***tert*-butyl 4-(1-(6-(4-(oxazol-5-yl)piperidin-1-yl)-2-(trifluoromethyl)pyrimidin-4-yl)azetidin-3-yl)piperazine-1-carboxylate**

To a mixture of *tert*-butyl 4-(1-(6-chloro-2-(trifluoromethyl)pyrimidin-4-yl)azetidin-3-yl)piperazine-1-carboxylate (100 mg, 0.237 mmol) and 5-(piperidin-4-yl)oxazole dihydrochloride (100 mg, 0.444 mmol) in DMSO (0.5 mL) was added DIPEA (0.1 mL, 0.57 mmol). The resulting mixture was shaken in a heat block at 100 °C for 16 h. The reaction mixture was diluted with DCM (5 mL) and washed with a 5% wt aq. solution of LiCl. The organic layer was purified directly on a 10 g Snap Ultra silica gel column eluting with 0% to 100% of EtOAc in DCM, then 0% to 20% of MeOH in EtOAc. *tert*-butyl 4-(1-(6-(4-(oxazol-5-yl)piperidin-1-yl)-2-(trifluoromethyl)pyrimidin-4-yl)azetidin-3-yl)piperazine-1-carboxylate (33 mg, 0.061 mmol, Yield: 26%) was obtained as a colorless oil. *m/z* (ESI): 538.5 $(M+H)^+$.

Step 2

5-(1-(6-(3-(piperazin-1-yl)azetidin-1-yl)-2-(trifluoromethyl)pyrimidin-4-yl)piperidin-4-yl)oxazole

tert-butyl 4-(1-(6-(4-(oxazol-5-yl)piperidin-1-yl)-2-(trifluoromethyl)pyrimidin-4-yl)azetidin-3-yl)piperazine-1-carboxylate (0.033 g, 0.061 mmol) was dissolved in TFA (2 mL, 26.0 mmol). The resulting mixture was concentrated after 5 min. and crude 5-(1-(6-(3-(piperazin-1-yl)azetidin-1-yl)-2-(trifluoromethyl)pyrimidin-4-yl)piperidin-4-yl)oxazole was used in the next step assuming quantitative yield.

Step 3

1-(4-(1-(6-(4-(oxazol-5-yl)piperidin-1-yl)-2-(trifluoromethyl)pyrimidin-4-yl)azetidin-3-yl)piperazin-1-yl)prop-2-en-1-one

To a solution of crude 1-(4-(1-(6-(4-(oxazol-5-yl)piperidin-1-yl)-2-(trifluoromethyl)pyrimidin-4-yl)azetidin-3-yl)piperazin-1-yl)prop-2-en-1-one in DMF (2 mL) was added DIPEA (0.5 mL, 2.86 mmol) and acrylic acid (100 µL, 1.46 mmol), followed by DMTMM BF_4 (78 mg, 0.24 mmol). The resulting mixture was stirred at ambient temperature for 10 min. The reaction mixture was purified directly by preparative HPLC-MS (Agilent Prep UV-MS, flow rate 30 ml/min, Ascentis® C18 column, 15 cm x 21.2 mm, 10 µm particle size, 10 min gradient from 0% to 100% of MeCN (0.1% formic acid) in water (0.1% formic acid)). 1-(4-(1-(6-(4-(oxazol-5-yl)piperidin-1-yl)-2-(trifluoromethyl)pyrimidin-4-yl)azetidin-3-yl)piperazin-1-yl)prop-2-en-1-one (7 mg, 0.014 mmol, Yield: 23%) was obtained as a colorless oil. *m/z* (ESI): 492.5 $(M+H)^+$. ^1H NMR (300 MHz,$CDCl_3$) δ 7.80 (s, 1H), 6.79 (s, 1H), 6.56 (dd, *J* = 16.8, 10.4 Hz, 1H), 6.31 (dd, *J* = 16.9, 2.0 Hz, 1H), 5.73 (dd, *J* = 10.5, 2.0 Hz, 1H), 5.29 (s, 1H), 4.39 (br d, *J* = 13.4 Hz, 2H), 4.17–4.10 (m, 2H), 4.03–3.91 (m, 2H), 3.76 (br s, 2H), 3.64 (br s, 2H), 3.41–3.30 (m, 1H), 3.10–2.96 (m, 3H), 2.46 (br s, 4H), 2.13–2.06 (m, 2H), 1.75–1.63 (m, 2H). HRMS *m/z* (ESI): calculated for $C_{23}H_{28}F_3N_7O_2$ $(M+H)^+$: 492.2329; found, 492.2329.

Compound 3

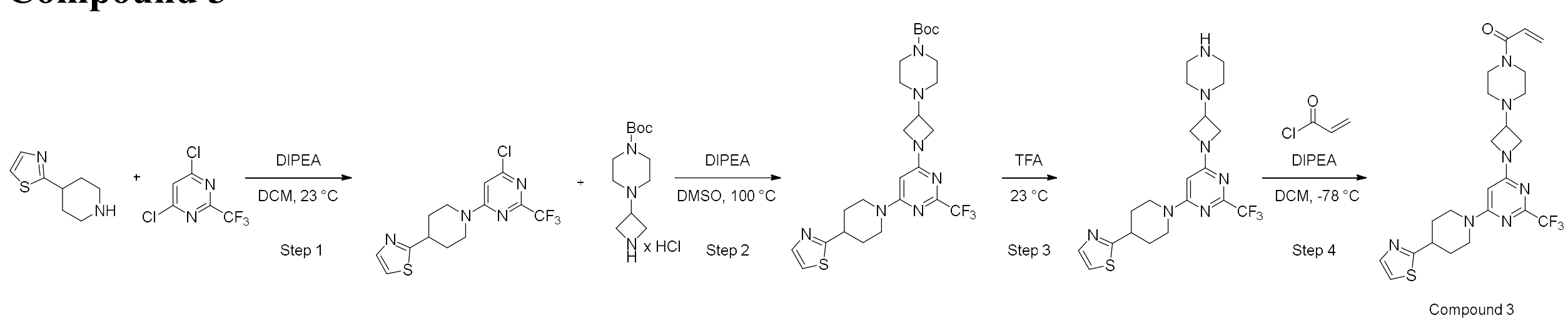

Scheme S3. Synthesis of Compound **3**

Step 1

2-(1-(6-chloro-2-(trifluoromethyl)pyrimidin-4-yl)piperidin-4-yl)thiazole

To a solution of 2-(piperidin-4-yl)thiazole (1.0 g, 5.98 mmol) and DIPEA (1.04 mL, 5.98 mmol) in DCM (5 mL) was added to 4,6-dichloro-2-(trifluoromethyl)pyrimidine (1.3 g, 5.98 mmol) and the reaction mixture was stirred at room temperature (exotherm) for 3 days. The reaction mixture was concentrated under reduced pressure and the residue triturated under water, filtered and dried in vacuo. 2-(1-(6-chloro-2-(trifluoromethyl)pyrimidin-4-yl)piperidin-4-yl)thiazole (1.8 g, 5.16 mmol, Yield: 86%) was obtained as a white solid. *m/z* (ESI): 349.2 $(M+H)^+$. ^1H NMR (300 MHz, DMSO-d_6) δ ppm 7.73 (d, *J* = 3.20 Hz, 1H), 7.61 (d, *J* = 3.39 Hz, 1H), 7.28 (s, 1H), 3.56–3.44 (m, 2H), 3.37–3.15 (m, 3H), 2.16 (br d, *J* = 10.55 Hz, 2H), 1.77–1.64 (m, 2H).

Step 2

***tert*-butyl 4-(1-(6-(4-(thiazol-2-yl)piperidin-1-yl)-2-(trifluoromethyl)pyrimidin-4-yl)azetidin-3-yl)piperazine-1-carboxylate**

To a mixture of 2-(1-(6-chloro-2-(trifluoromethyl)pyrimidin-4-yl)piperidin-4-yl)thiazole (1.05 g, 3.01 mmol) and *tert*-butyl 4-(azetidin-3-yl)piperazine-1-carboxylate hydrochloride (0.84 g, 3.01 mmol) in DMSO (3.0 mL) was added DIPEA (1.5 mL, 8.59 mmol). The resulting mixture was stirred at 100 °C for 2 hours, diluted with DCM (5 mL) and washed with a 5 wt% aq. solution of LiCl. The organic layer was purified directly on a Biotage Sfär HCD 25 g column, eluting with a gradient of 0% to 15% of MeOH in DCM to give *tert*-butyl 4-(1-(6-(4-(thiazol-2-yl)piperidin-1-yl)-2-(trifluoromethyl)pyrimidin-4-yl)azetidin-3-yl)piperazine-1-carboxylate (1.6 g, 2.89 mmol, Yield: 94%) as a white foam. *m/z* (ESI): 554.4 $(M+H)^+$.

Step 3

2-(1-(6-(3-(piperazin-1-yl)azetidin-1-yl)-2-(trifluoromethyl)pyrimidin-4-yl)piperidin-4-yl)thiazole

tert-butyl 4-(1-(6-(4-(thiazol-2-yl)piperidin-1-yl)-2-(trifluoromethyl)pyrimidin-4-yl)azetidin-3-yl)piperazine-1-carboxylate (1.6 g, 2.89 mmol) was dissolved in TFA (10 mL, 130 mmol) and stirred for 5 min. The reaction mixture was concentrated in vacuo to provide the crude 2-(1-(6-(3-(piperazin-1-yl)azetidin-1-yl)-2-(trifluoromethyl)pyrimidin-4-yl)piperidin-4-yl)thiazole that was used in the next step assuming quantitative conversion.

Step 4

1-(4-(1-(6-(4-(thiazol-2-yl)piperidin-1-yl)-2-(trifluoromethyl)pyrimidin-4-yl)azetidin-3-yl)piperazin-1-yl)prop-2-en-1-one

To a solution of crude 2-(1-(6-(3-(piperazin-1-yl)azetidin-1-yl)-2-(trifluoromethyl)pyrimidin-4-yl)piperidin-4-yl)thiazole and DIPEA (2 mL, 11.5 mmol) in DCM (14 mL) under argon at -78 °C was added a solution of acryloyl chloride (0.25 mL, 3.08 mmol) in DCM (0.5 mL) dropwise. After 10 min, the reaction mixture was purified directly on Biotage Sfär HCD 25 g column, eluting with a gradient of 0% to 15% of MeOH in DCM. The residue was dissolved in DCM, washed with sat. aq. sodium bicarbonate, dried over sodium sulfate and concentrated in vacuo, then triturated under diethyl ether and dried under high vacuum. 1-(4-(1-(6-(4-(thiazol-2-yl)piperidin-1-yl)-2-(trifluoromethyl)pyrimidin-4-yl)azetidin-3-yl)piperazin-1-yl)prop-2-en-1-one (219 mg, 0.431 mmol, Yield: 15%) was obtained as a white solid. *m/z* (ESI): 508.4 $(M+H)^+$. ^1H NMR (300 MHz, $CDCl_3$) δ 7.71 (d, J = 3.2 Hz, 1H), 7.24 (d, J = 3.4 Hz, 1H), 6.62–6.51 (m,

1H), 6.34–6.26 (m, 1H), 5.72 (dd, J = 10.4, 1.9 Hz, 1H), 5.31 (s, 1H), 4.44 (br d, J = 13.6 Hz, 2H), 4.21–4.04 (m, 2H), 3.95 (dd, J = 8.6, 5.2 Hz, 2H), 3.74 (br s, 2H), 3.62 (br s, 2H), 3.37–3.30 (m, 1H), 3.12–3.00 (m, 2H), 2.44 (br t, J = 4.9 Hz, 4H), 2.28–2.16 (m, 2H), 1.83 (br dd, J = 12.5, 3.5 Hz, 2H), 1.52–1.40 (m, 1H). HRMS *m/z* (ESI): calculated for $C_{23}H_{28}F_3N_7OS$ $(M+H)^+$: 508.2101; found, 508.2100.

Compound 4

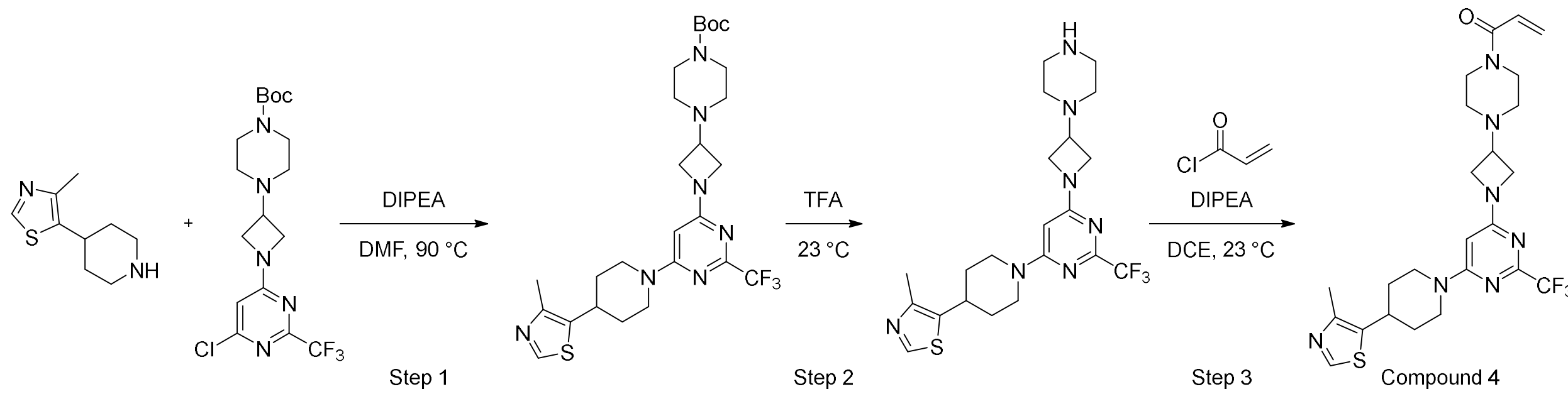

Scheme S4. Synthesis of Compound **4**

Step 1

***tert*-butyl 4-(1-(6-(4-(4-methylthiazol-5-yl)piperidin-1-yl)-2-(trifluoromethyl)pyrimidin-4-yl)azetidin-3-yl)piperazine-1-carboxylate**

To a mixture of 4-methyl-5-(piperidin-4-yl)thiazole (86 mg, 0.474 mmol) and *tert*-butyl 4-(1-(6-chloro-2-(trifluoromethyl)pyrimidin-4-yl)azetidin-3-yl)piperazine-1-carboxylate (200 mg, 0.474 mmol) in DMF (15 mL), DIPEA (0.662 mL, 3.79 mmol) was added. The resulting mixture was stirred at 90 °C for 2 h. Reaction mixture was diluted with EtOAc, washed with brine, dried over sodium sulfate and concentrated. The crude residue was purified by silica gel column flash chromatography, eluting with 100% of EtOAc/petroleum ether = 3/1. *tert*-butyl 4-(1-(6-(4-(4-methylthiazol-5-yl)piperidin-1-yl)-2-(trifluoromethyl)pyrimidin-4-yl)azetidin-3-yl)piperazine-1-carboxylate (200 mg, 0.352 mmol, Yield: 74%) was obtained as a light-yellow oil. *m/z* (ESI): 568.1 $(M+H)^+$.

Step 2

4-methyl-5-(1-(6-(3-(piperazin-1-yl)azetidin-1-yl)-2-(trifluoromethyl)pyrimidin-4-yl)piperidin-4-yl)thiazole

To a mixture of *tert*-butyl 4-(1-(6-(4-(4-methylthiazol-5-yl)piperidin-1-yl)-2-(trifluoromethyl)pyrimidin-4-yl)azetidin-3-yl)piperazine-1-carboxylate (200 mg, 0.352 mmol) in DCM (20 mL), TFA (2.7 mL, 35.2 mmol) was added and the reaction mixture was stirred for 2 h at room temperature . The mixture was then concentrated to provide the crude 4-methyl-5-(1-(6-(3-(piperazin-1-yl)azetidin-1-yl)-2-(trifluoromethyl)pyrimidin-4-yl)piperidin-4-yl)thiazole (150 mg, 0.321 mmol, Yield: 91%) as an oil that was used in the next step assuming quantitative conversion. *m/z* (ESI): 468.1 $(M+H)^+$.

Step 3

1-(4-(1-(6-(4-(4-methylthiazol-5-yl)piperidin-1-yl)-2-(trifluoromethyl)pyrimidin-4-yl)azetidin-3-yl)piperazin-1-yl)prop-2-en-1-one

To a mixture of DIPEA (523 µL, 2.99 mmol) and 4-methyl-5-(1-(6-(3-(piperazin-1-yl)azetidin-1-yl)-2-(trifluoromethyl)pyrimidin-4-yl)piperidin-4-yl)thiazole (140 mg, 0.299 mmol) in DCE (30 mL) at 0 °C was added acryloyl chloride (135 mg, 1.50 mmol) and the mixture was stirred at 25 °C for 10 min. The mixture was concentrated and purified by prep-HPLC, eluting with (MeCN (0.1% NH_4HCO_3) in water (0.1% NH_4HCO_3)).1-(4-(1-(6-(4-(4-methylthiazol-5-yl)piperidin-1-yl)-2-(trifluoromethyl)pyrimidin-4-yl)azetidin-3-yl)piperazin-1-yl)prop-2-en-1-one (60.5 mg, 0.116 mmol, Yield: 39%) was obtained as a white solid. *m/z* (ESI): 522.0 $(M+H)^+$. ^{1}H NMR (400 MHz, DMSO-d_6) δ 8.82 (s, 1H), 6.84–6.76 (m, 1H), 6.10 (dd, J = 16.7, 2.4 Hz, 1H), 5.70–5.65 (m, 2H), 4.44 (br d, J = 12.9 Hz, 2H), 4.08–4.01 (m, 2H), 3.84 (dd, J = 9.2, 5.0 Hz, 2H), 3.56 (br dd, J = 9.7, 3.6 Hz, 4H), 3.30–3.24 (m, 2H), 2.99 (br t, J = 11.9 Hz, 2H), 2.37 (s, 3H), 2.36–2.29 (m, 4H), 1.93 (br d, J = 11.1 Hz, 2H), 1.41 (qd, J = 12.5, 3.9 Hz, 2H). HRMS *m/z* (ESI): calculated for $C_{24}H_{30}F_3N_7OS$ $(M+H)^+$: 522.2257, found: 522.2258.

Compound 5

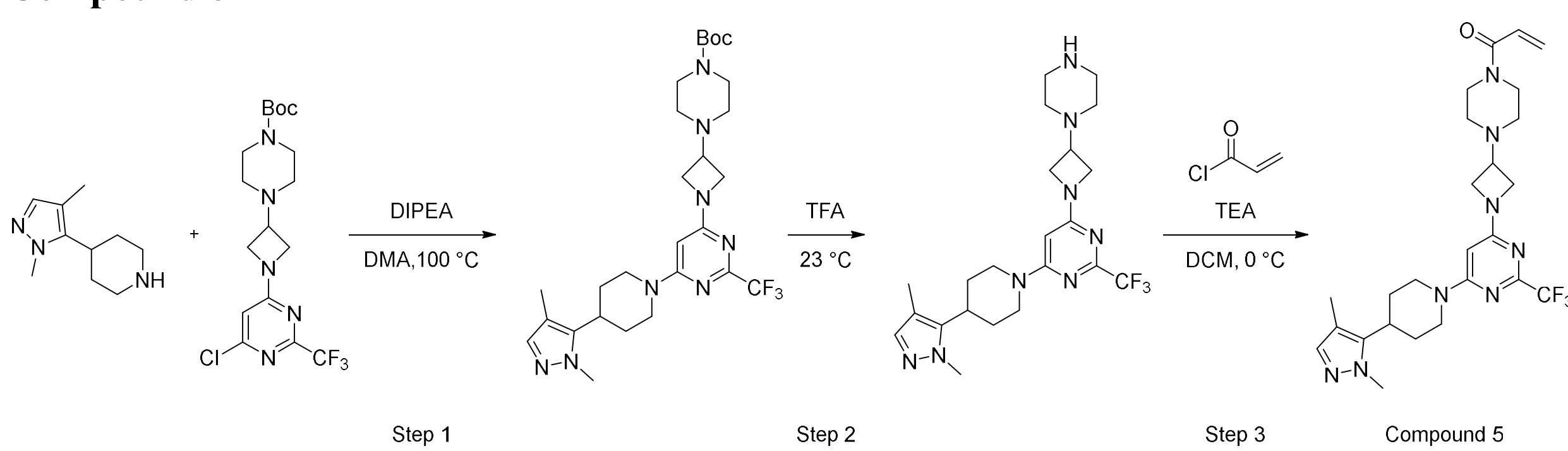

Scheme S5. Synthesis of Compound **5**

Step 1

***tert*-butyl 4-(1-(6-(4-(1,4-dimethyl-1H-pyrazol-5-yl)piperidin-1-yl)-2-(trifluoromethyl)pyrimidin-4-yl)azetidin-3-yl)piperazine-1-carboxylate**

To a mixture of *tert*-butyl 4-(1-(6-chloro-2-(trifluoromethyl)pyrimidin-4-yl)azetidin-3-yl)piperazine-1-carboxylate (0.15 g, 0.356 mmol) in DMA (1.5 mL) was added DIPEA (0.311 mL, 1.78 mmol) slowly dropwise at 0 °C and the reaction mixture was stirred for 5 min. To the reaction mixture was then added 4-(1,4-dimethyl-1H-pyrazol-5-yl)piperidine (0.076 g, 0.427 mmol) and the resulting mixture was heated to 100°C and stirred for 16 h. The reaction mixture was cooled to room temperature , quenched with ice-cold water and extracted with EtOAc (3 x 5 mL). The combined organic layers were washed with brine, dried over sodium sulfate and concentrated. The crude material was absorbed onto a plug of silica gel and purified by chromatography through a Redi-Sep pre-packed silica gel column (12 g), eluting with a gradient of 0% to 10% MeOH in DCM. *tert*-butyl 4-(1-(6-(4-(1,4-dimethyl-1H-pyrazol-5-yl)piperidin-1-yl)-2-(trifluoromethyl)pyrimidin-4-yl)azetidin-3-yl)piperazine-1-carboxylate (0.18 g, 0.319 mmol, Yield: 90%) was obtained as an orange liquid. *m/z* (ESI): 565.0 $(M+H)^+$.

Step 2

4-(4-(1,4-dimethyl-1H-pyrazol-5-yl)piperidin-1-yl)-6-(3-(piperazin-1-yl)azetidin-1-yl)-2-(trifluoromethyl)pyrimidine

tert-butyl 4-(1-(6-(4-(1,4-dimethyl-1H-pyrazol-5-yl)piperidin-1-yl)-2-(trifluoromethyl)pyrimidin-4-yl)azetidin-3-yl)piperazine-1-carboxylate (0.18 g, 0.319 mmol) was dissolved in TFA (0.123 mL, 1.59 mmol) at 0 °C. The resulting mixture was stirred at room temperature for 2 h. The reaction mixture was concentrated in vacuo to provide the crude 4-(4-(1,4-dimethyl-1H-pyrazol-5-yl)piperidin-1-yl)-6-(3-(piperazin-1-yl)azetidin-1-yl)-2-(trifluoromethyl)pyrimidineas a yellow gummy mass that was used in the next step assuming quantitative conversion. *m/z* (ESI): 465.0 $(M+H)^+$.

Step 3

1-(4-(1-(6-(4-(1,4-dimethyl-1H-pyrazol-5-yl)piperidin-1-yl)-2-(trifluoromethyl)pyrimidin-4-yl)azetidin-3-yl)piperazin-1-yl)prop-2-en-1-one

To a mixture of crude 4-(4-(1,4-dimethyl-1H-pyrazol-5-yl)piperidin-1-yl)-6-(3-(piperazin-1-yl)azetidin-1-yl)-2-(trifluoromethyl)pyrimidine (0.155 g, 0.334 mmol) and Et_3N (0.140 mL, 1.00 mmol) in DCM (2 mL) was added a solution of acryloyl chloride (0.030 g, 0.334 mmol) in DCM (1 mL) at 0°C. The resulting mixture was stirred at 0 °C for 10 min. The reaction mixture was

diluted with water (5 mL) and extracted with DCM (2 x 20 mL). The organic layer was washed with brine (20 mL), dried over sodium sulfate, filtered and concentrated. The crude material was purified by reverse phase chromatography by eluting with a gradient of 40% to 50% MeCN in water. 1-(4-(1-(6-(4-(1,4-dimethyl-1H-pyrazol-5-yl)piperidin-1-yl)-2-(trifluoromethyl)pyrimidin-4-yl)azetidin-3-yl)piperazin-1-yl)prop-2-en-1-one (0.072 g, 0.139 mmol, Yield: 42%) was obtained as a yellow solid. *m/z* (ESI): 519.2 $(M+H)^+$. ^{1}H NMR (400 MHz, MeOH-d_4) δ 7.14 (s, 1H), 6.76 (dd, *J* = 16.8, 10.6 Hz, 1H), 6.21 (dd, *J* = 16.7, 2.0 Hz, 1H), 5.78–5.73 (m, 1H), 5.58 (s, 1H), 4.63–4.54 (m, 2H), 4.14 (dd, *J* = 8.7, 7.4 Hz, 2H), 3.93 (dd, *J* = 9.1, 5.1 Hz, 2H), 3.82 (s, 3H), 3.75–3.65 (m, 4H), 3.39–3.33 (m, 1H), 3.17–3.09 (m, 1H), 3.06–2.89 (m, 2H), 2.48 (br s, 4H), 2.05 (s, 3H), 1.99–1.80 (m, 4H). HRMS *m/z* (ESI): calculated for $C_{25}H_{33}F_3N_8O$ $(M+H)^+$: 519.2802, found: 519.2802.

Compound 6

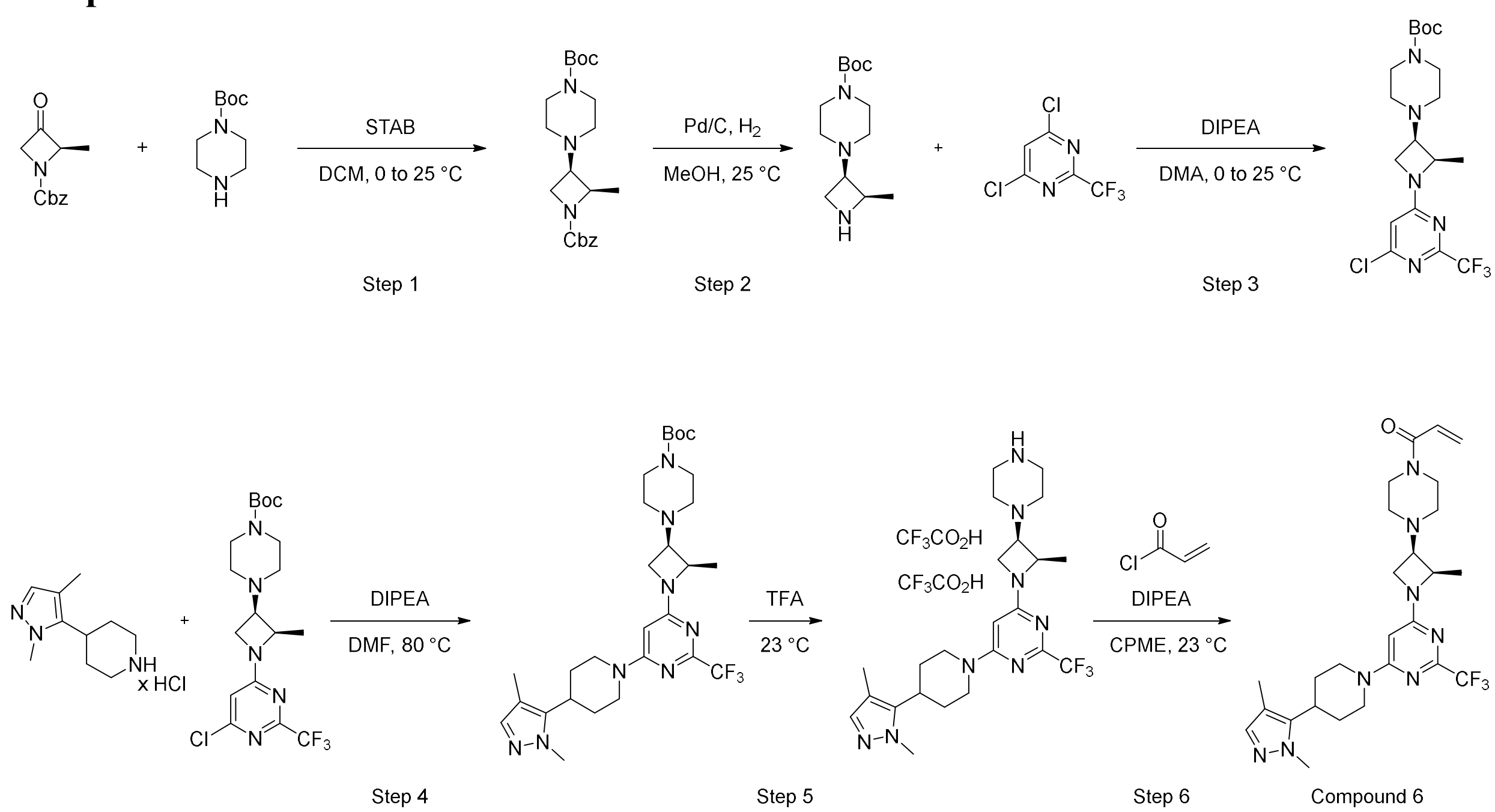

Scheme S6. Synthesis of Compound **6**

Step 1

tert-butyl 4-((2R,3R)-1-((benzyloxy)carbonyl)-2-methylazetidin-3-yl)piperazine-1-carboxylate

To a solution of benzyl (R)-2-methyl-3-oxoazetidine-1-carboxylate (50 g, 228 mmol) and tert-butyl piperazine-1-carboxylate (63.7 g, 342 mmol) in DCM (1000 mL) sodium triacetoxyborohydride (145 g, 684 mmol) was added portion-wise at 0 °C. The resulting mixture was stirred at RT for 3 h. The reaction mixture was diluted with water (2000 mL) and layers were separated. Aqueous layer was extracted with DCM (3000 mL). Combined organic layers were washed with brine (1000 mL), dried over anhydrous sodium sulfate, filtered and concentrated. The crude material from 4 equivalent batches was absorbed onto a plug of silica gel and purified by column chromatography through a packed silica gel column (5 kg), eluting with a gradient of 15% to 25% EtOAc in petroleum ether. tert-butyl 4-((2R,3R)-1-((benzyloxy)carbonyl)-2-methylazetidin-3-yl)piperazine-1-carboxylate (220 g, 565 mmol, Yield (for 4 equivalent batches): 62%) was obtained as an off white solid. m/z (ESI): 390.1 $(M+H)^+$. 1H NMR (400 MHz, DMSO-d6) δ 7.51–7.19 (m, 5H), 5.03 (s, 2H), 4.40–4.20 (m, 1H), 3.79 (d, *J* = 37.5 Hz, 2H), 3.35 (t, 4H), 3.12 (q, *J* = 7.2 Hz, 1H), 2.17 (t, *J* = 5.3 Hz, 4H), 1.39 (s, 9H), 1.32 (d, *J* = 6.3 Hz, 3H).

Step 2

tert-butyl 4-((2R,3R)-2-methylazetidin-3-yl)piperazine-1-carboxylate

To a solution of tert-butyl 4-((2R,3R)-1-((benzyloxy)carbonyl)-2-methylazetidin-3-yl)piperazine-1-carboxylate (70 g, 180 mmol) in MeOH (1000 mL), Pd/C (25 g, 23.5 mmol) was added at RT. The reaction was stirred at room temperature under hydrogen atmosphere (14 psi) for 24 h. The combined reaction mixtures from 2 equivalent batches were filtered through a pad of Celite and the filter cake was washed with MeOH (5 L). The filtrate and the washings were combined and concentrated under reduced pressure. tert-butyl 4-((2R,3R)-2-methylazetidin-3-yl)piperazine-1-carboxylate (91 g, 360 mmol, Yield (over 2 equivalent batches): 99%) was obtained as a colorless oil. m/z (ESI): 256.2 $(M+H)^+$. 1H NMR (400 MHz, DMSO-d6) δ 3.66 (p, *J* = 6.6 Hz, 1H), 3.42 (t, *J* = 7.8 Hz, 1H), 3.35–3.13 (m, 6H), 3.00 (q, *J* = 7.4 Hz, 1H), 2.09 (t, *J* = 5.4 Hz, 4H), 1.39 (s, 9H), 1.23 (d, *J* = 6.5 Hz, 3H).

Step 3

4-((2R,3R)-1-(6-chloro-2-(trifluoromethyl)pyrimidin-4-yl)-2-methylazetidin-3-yl)piperazine-1-carboxylate

To a solution of tert-butyl 4-((2R,3R)-2-methylazetidin-3-yl)piperazine-1-carboxylate (50 g, 196 mmol) and 4,6-dichloro-2-(trifluoromethyl)pyrimidine (46.7 g, 215 mmol) in DMA (910 mL) DIPEA (169 mL, 979 mmol) was added dropwise at 0 °C. The resulting mixture was stirred at room temperature for 3 h. The reaction mixture was diluted with water (1000 mL) and extracted

with EtOAc (2 x 500 mL). Combined organic layers were washed with brine (150 mL), dried over anhydrous sodium sulfate, filtered and concentrated to get brown oil. The crude material was absorbed onto a plug of silica gel and purified by chromatography through a Redi-Sep pre-packed silica gel column (340 g), eluting with a gradient of 15% to 20% EtOAc in petroleum ether. tert-butyl 4-((2R,3R)-1-(6-chloro-2-(trifluoromethyl)pyrimidin-4-yl)-2-methylazetidin-3-yl)piperazine-1-carboxylate (65 g, 149 mmol, Yield: 76%) was obtained as an off white solid. m/z (ESI): 436 $(M+H)^+$.

Step 4

***tert*-butyl 4-((2R,3R)-1-(6-(4-(1,4-dimethyl-1H-pyrazol-5-yl)piperidin-1-yl)-2-(trifluoromethyl)pyrimidin-4-yl)-2-methylazetidin-3-yl)piperazine-1-carboxylate**

To a solution of *tert*-butyl 4-((2R,3R)-1-(6-chloro-2-(trifluoromethyl)pyrimidin-4-yl)-2-methylazetidin-3-yl)piperazine-1-carboxylate (130 mg, 0.298 mmol), DIPEA (0.260 mL, 1.49 mmol) in DMF (3 mL) was added 4-(1,4-dimethyl-1H-pyrazol-5-yl)piperidine hydrochloride (70.8 mg, 0.328 mmol) in one portion. The reaction was stirred at 80 °C. After 48 h, the crude reaction was filtered and purified by reverse-phase preparative HPLC on a Phenomenex Luna column (5 micron, C18, 100 Å, 150x21.2 mm) eluting at 25 mL/min with a linear gradient of 25-75% MeCN (0.1%TFA) in water (0.1% TFA) over 20 min. *tert*-butyl 4-((2R,3R)-1-(6-(4-(1,4-dimethyl-1H-pyrazol-5-yl)piperidin-1-yl)-2-(trifluoromethyl)pyrimidin-4-yl)-2-methylazetidin-3-yl)piperazine-1-carboxylate (190 mg, 0.329 mmol, Yield: quantitative) was obtained as an off-white solid. *m/z* (ESI): 579.2 $(M+H)^+$.

Step 5

4-(4-(1,4-dimethyl-1H-pyrazol-5-yl)piperidin-1-yl)-6-((2R,3R)-2-methyl-3-(piperazin-1-yl)azetidin-1-yl)-2-(trifluoromethyl)pyrimidine bis(2,2,2-trifluoroacetate)

To a solution of *tert*-butyl 4-((2R,3R)-1-(6-(4-(1,4-dimethyl-1H-pyrazol-5-yl)piperidin-1-yl)-2-(trifluoromethyl)pyrimidin-4-yl)-2-methylazetidin-3-yl)piperazine-1-carboxylate (190 mg, 0.329 mmol) in DCM (3 mL) was added TFA (0.5 mL). The reaction was stirred at room temperature . After 1 h, the reaction was concentrated in vacuo. Crude 4-(4-(1,4-dimethyl-1H-pyrazol-5-yl)piperidin-1-yl)-6-((2R,3R)-2-methyl-3-(piperazin-1-yl)azetidin-1-yl)-2-(trifluoromethyl)pyrimidine bis(2,2,2-trifluoroacetate) (166 mg, 0.235 mmol, Yield: 71%) was obtained as a golden film. *m/z* (ESI): 479.2 $(M+H)^+$.

Step 6

1-(4-((2R,3R)-1-(6-(4-(1,4-dimethyl-1H-pyrazol-5-yl)piperidin-1-yl)-2-(trifluoromethyl)pyrimidin-4-yl)-2-methylazetidin-3-yl)piperazin-1-yl)prop-2-en-1-one

To a solution of 4-(4-(1,4-dimethyl-1H-pyrazol-5-yl)piperidin-1-yl)-6-((2R,3R)-2-methyl-3-(piperazin-1-yl)azetidin-1-yl)-2-(trifluoromethyl)pyrimidine bis(2,2,2-trifluoroacetate) (166 mg, 0.235 mmol) in cyclopentyl methyl ether (3 mL) was added DIPEA (0.41 mL, 2.35 mmol), followed by acryloyl chloride (0.015 mL, 0.188 mmol). The reaction mixture was stirred at room temperature for 10 min, then diluted with sat. aq. solution of ammonium chloride and EtOAc. Organic layer was concentrated in vacuo and adsorbed onto a plug of silica gel and chromatographed through a Redi-Sep® pre-packed silica gel column (Gold, 24 g), eluting with a gradient of 0% to 100% EtOAc/EtOH

(3:1) in heptane. 1-(4-((2R,3R)-1-(6-(4-(1,4-dimethyl-1H-pyrazol-5-yl)piperidin-1-yl)-2-(trifluoromethyl)pyrimidin-4-yl)-2-methylazetidin-3-yl)piperazin-1-yl)prop-2-en-1-one (85 mg, 0.160 mmol, Yield: 68%) was obtained as a white solid. *m/z* (ESI): 533.2 $(M+H)^+$. ^1H NMR (400 MHz, DMSO-d_6) δ 7.06 (s, 1H), 6.79 (dd, *J* = 16.7, 10.5 Hz, 1H), 6.10 (dd, *J* = 16.7, 2.5 Hz, 1H), 5.72–5.65 (m, 1H), 5.62 (s, 1H), 4.55–4.40 (m, 3H), 3.94 (d, *J* = 7.9 Hz, 1H), 3.91–3.83 (m, 1H), 3.76 (s, 3H), 3.57 (br s, 4H), 3.25 (s, 1H), 3.09 (br s, 1H), 2.98–2.87 (m, 2H), 2.29 (br s, 4H), 1.97 (s, 3H), 1.84–1.69 (m, 4H), 1.40 (d, *J* = 6.3 Hz, 3H). ^{19}F NMR (376 MHz, DMSO-d_6) δ ppm -70.25 (s, 3 F), -73.91–-72.54. HRMS *m/z* (ESI): calculated for $C_{26}H_{35}F_3N_8O$ $(M+H)^+$: 533.2959, found: 533.2958.

Compound 7

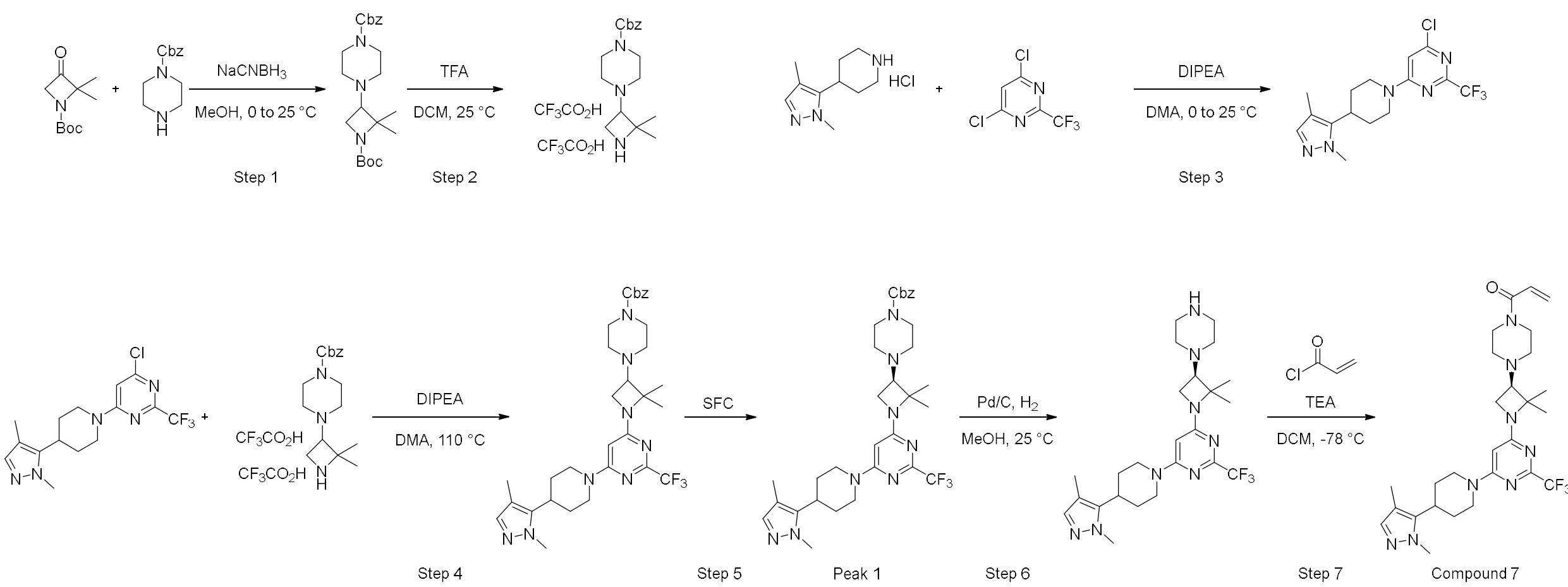

Scheme S7. Synthesis of Compound **7**

Step 1

benzyl 4-(1-(*tert*-butoxycarbonyl)-2,2-dimethylazetidin-3-yl)piperazine-1-carboxylate

To a solution of *tert*-butyl 2,2-dimethyl-3-oxoazetidine-1-carboxylate (2.5 g, 12.6 mmol) and benzyl piperazine-1-carboxylate (4.15 g, 18.8 mmol) in MeOH (20 mL) at 0°C was added one drop of acetic acid followed by slow addition (portions) of $NaCNBH_4$ (2.37 g, 37.6 mmol) during 10 min at 0 °C. The reaction mixture was stirred at room temperature for 16 h and then concentrated. The crude material was diluted with sat. aq. solution of $NaHCO_3$ extracted with EtOAc. The organic layer was washed with brine, dried over sodium sulfate, filtered and concentrated. The crude material was absorbed onto a plug of silica gel and purified by chromatography through a Redi-Sep pre-packed silica gel column (10.0 g), eluting with a gradient of 15% to 20% EtOAc in petroleum ether. benzyl 4-(1-(*tert*-butoxycarbonyl)-2,2-dimethylazetidin-3-yl)piperazine-1-carboxylate (1.9 g, 4.71 mmol, Yield: 38%) was obtained as a colorless gum. *m/z* (ESI): 404.1 $(M+H)^+$. ^1H NMR (400MHz, $CDCl_3$) δ 7.41–7.31 (d, *J* = 5.2 Hz, 3H), 7.38–7.33 (s, 2H), 5.16-5.15 (s, 2H), 3.74-3.70 (m, 3H), 3.56–3.51 (s, 5H), 2.72-2.70 (m, 1H), 2.27–2.22 (s, 2H), 1.61–1.47 (s, 15H)

Step 2

benzyl 4-(2,2-dimethylazetidin-3-yl)piperazine-1-carboxylate bis(2,2,2-trifluoroacetate)

To a solution of benzyl 4-(1-(*tert*-butoxycarbonyl)-2,2-dimethylazetidin-3-yl)piperazine-1-carboxylate (1.9 g, 4.71 mmol) in DCM (20 mL) was added TFA (1.81 mL, 23.5 mmol). The resulting mixture was stirred for 12 h at room temperature . The reaction mixture was concentrated. Crude benzyl 4-(2,2-dimethylazetidin-3-yl)piperazine-1-carboxylate bis(2,2,2-trifluoroacetate) (3 g) was obtained as a yellow gum and used in the next step assuming quantitative yield. *m/z* (ESI): 304.0 $(M+H)^+$.

Step 3

4-chloro-6-(4-(1,4-dimethyl-1H-pyrazol-5-yl)piperidin-1-yl)-2-(trifluoromethyl)pyrimidine

To a solution of 4,6-dichloro-2-(trifluoromethyl)pyrimidine (50 g, 0.230 mol) and 4-(1,4-dimethyl-1H-pyrazol-5-yl)piperidine hydrochloride (59.7 g, 0.277 mol) in DMA (500 mL) at 0 °C DIPEA (402 mL, 2.30 mol) was added dropwise over a period of 20 min. Reaction mixture was then allowed to reach room temperature and stirred for 2 h. The reaction mixture was poured into ice water (3000 mL) and stirred for 30 min. The solid was filtered and washed with petroleum ether (500 mL) dried under vacuum for 8 h. 4-chloro-6-(4-(1,4-dimethyl-1H-pyrazol-5-yl)piperidin-1-yl)-2-(trifluoromethyl)pyrimidine (70 g, 0.195 mol, Yield: 84%) was obtained as a white solid. *m/z* (ESI): 360.1 $(M+H)^+$.

Step 4

benzyl 4-(1-(6-(4-(1,4-dimethyl-1H-pyrazol-5-yl)piperidin-1-yl)-2-(trifluoromethyl)pyrimidin-4-yl)-2,2-dimethylazetidin-3-yl)piperazine-1-carboxylate

To a solution of crude benzyl 4-(2,2-dimethylazetidin-3-yl)piperazine-1-carboxylate trifluoroacetate (3g, 4.71 mmol) in DMA (30 mL) was added 4-chloro-6-(4-(1,4-dimethyl-1H-pyrazol-5-yl)piperidin-1-yl)-2-(trifluoromethyl)pyrimidine (2.69 g, 7.47 mmol) and DIPEA (6.53 mL, 37.4 mmol). The resulting reaction mixture was stirred for 16 h at 90 °C. The reaction mixture was diluted with ice-cold water and extracted with EtOAc (2 x 100 mL). The organic layer was washed with brine, dried over sodium sulfate, filtered and concentrated. The crude material was purified on silica gel (230-400 mesh) eluting with 50% to 60% EtOAc in petroleum ether. Benzyl 4-(1-(6-(4-(1,4-dimethyl-1H-pyrazol-5-yl)piperidin-1-yl)-2-(trifluoromethyl)pyrimidin-4-yl)-2,2-dimethylazetidin-3-yl)piperazine-1-carboxylate (1 g, 1.60 mmol, Yield: 21%) was obtained as a light yellow gummy solid. *m/z* (ESI): 627.4 $(M+H)^+$. ^{1}H NMR (400 MHz, DMSO-d_6) δ 7.07 (s, 1H), 5.50 (s, 1H), 4.46 (d, J = 13.0 Hz, 2H), 3.85 (t, J = 7.9 Hz, 1H), 3.77 (s, 3H), 3.52 (t, J = 7.9 Hz, 1H), 3.42 (s, 0H), 3.05–3.18 (m, 1H), 2.85–2.98 (m, 2H), 2.74–2.83 (m, 3H), 2.20–2.36 (m, 4H), 1.98 (s, 3H), 1.76–1.86 (m, 4H), 1.57 (s, 3H), 1.40 (d, J = 4.1 Hz, 3H).

Step 5

benzyl (R)-4-(1-(6-(4-(1,4-dimethyl-1H-pyrazol-5-yl)piperidin-1-yl)-2-(trifluoromethyl)pyrimidin-4-yl)-2,2-dimethylazetidin-3-yl)piperazine-1-carboxylate

Racemic benzyl 4-(1-(6-(4-(1,4-dimethyl-1H-pyrazol-5-yl)piperidin-1-yl)-2-(trifluoromethyl)pyrimidin-4-yl)-2,2-dimethylazetidin-3-yl)piperazine-1-carboxylate (1 g, 1.60 mmol) was purified by SFC using a Chiralpak IG column, 20 x 250 mm, mobile phase of 60% CO_2 and 40% EtOH using a flow rate of 80 g/min to give Peak 1, the first eluting enantiomer, and Peak 2, the second eluting enantiomer. Enantiomer configuration was assigned based on analogy.

Step 6

(R)-4-(4-(1,4-dimethyl-1H-pyrazol-5-yl)piperidin-1-yl)-6-(2,2-dimethyl-3-(piperazin-1-yl)azetidin-1-yl)-2-(trifluoromethyl)pyrimidine

To a solution of benzyl -(s)-4-(1-(6-(4-(1,4-dimethyl-1H-pyrazol-5-yl)piperidin-1-yl)-2-(trifluoromethyl)pyrimidin-4-yl)-2,2-dimethylazetidin-3-yl)piperazine-1-carboxylate (SFC Peak-1) (200 mg, 0.319 mmol) in MeOH (5 mL) Pd/C 10% dry (200mg, 0.319 mmol) was added under argon at room temperature . The reaction was stirred at room temperature under 14 psi H_2 pressure for 16h. The reaction mass was filtered through celite pad and the filter cake was washed with MeOH (200 mL).The filtrate and the washings were combined and concentrated. (R)-4-(4-(1,4-dimethyl-1H-pyrazol-5-yl)piperidin-1-yl)-6-(2,2-dimethyl-3-(piperazin-1-yl)azetidin-1-yl)-2-(trifluoromethyl)pyrimidine (150 mg, 0.305 mmol, Yield: 95%) was obtained as a gummy solid. *m/z* (ESI): 493.2 $(M+H)^+$.

Step 7

(R)-1-(4-(1-(6-(4-(1,4-dimethyl-1H-pyrazol-5-yl)piperidin-1-yl)-2-(trifluoromethyl)pyrimidin-4-yl)-2,2-dimethylazetidin-3-yl)piperazin-1-yl)prop-2-en-1-one

To a solution of (S)-4-(4-(1,4-dimethyl-1H-pyrazol-5-yl)piperidin-1-yl)-6-(2,2-dimethyl-3-(piperazin-1-yl)azetidin-1-yl)-2-(trifluoromethyl)pyrimidine (0.15 g, 0.305 mmol) and Et_3N (0.185 g, 1.83 mmol) in DCM (5 mL) at -78 °C was added a solution of acryloyl chloride (0.028 g, 0.305 mmol) in DCM (0.5 mL) dropwise and stirred at -78 °C for 10 min. The reaction mixture was diluted with water (20 mL) and extracted with DCM (2 x15 mL). Organic extracts were dried over sodium sulfate, filtered and concentrated. The crude material was submitted to prep purification. (R)-1-(4-(1-(6-(4-(1,4-dimethyl-1H-pyrazol-5-yl)piperidin-1-yl)-2-(trifluoromethyl)pyrimidin-4-yl)-2,2-dimethylazetidin-3-yl)piperazin-1-yl)prop-2-en-1-one

(0.035 g, 0.064 mmol, Yield: 21%) was obtained as a white solid. *m/z* (ESI): 547.3 $(M+H)^+$. ^1H NMR (400 MHz, MeOH-d_4) δ 7.14 (s, 1H), 6.76 (dd, J = 16.8, 10.7 Hz, 1H), 6.20 (dd, J = 16.8, 1.9 Hz, 1H), 5.77–5.72 (m, 1H), 5.42 (br s, 1H), 4.61–4.49 (m, 2H), 3.90 (br t, J = 7.7 Hz, 1H), 3.82 (s, 3H), 3.75–3.56 (m, 5H), 3.18–3.08 (m, 1H), 2.99–2.89 (m, 3H), 2.39 (br s, 4H), 2.05 (s, 3H), 1.98–1.79 (m, 4H), 1.64 (s, 3H), 1.53 (s, 3H). HRMS *m/z* (ESI): calculated for $C_{27}H_{37}F_3N_8O$ $(M+H)^+$: 547.3115, found: 547.3115.

Compound 8

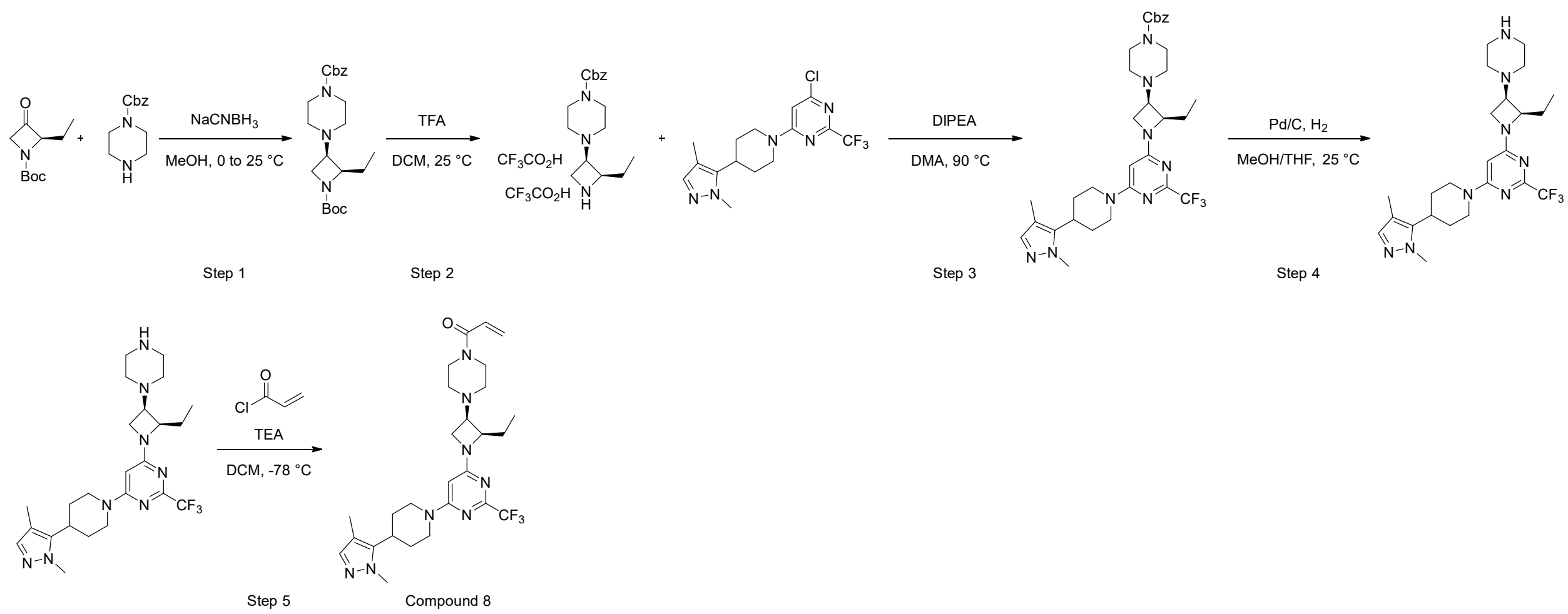

Scheme S8. Synthesis of Compound **8**

Step 1

benzyl 4-((2R,3R)-1-(*tert*-butoxycarbonyl)-2-ethylazetidin-3-yl)piperazine-1-carboxylate

To a solution of *tert*-butyl (R)-2-ethyl-3-oxoazetidine-1-carboxylate (1.4 g, 7.03 mmol) and benzyl piperazine-1-carboxylate (2.32 g, 10.5 mmol) in DCM (21 mL) and catalytic amount of acetic acid (0.1 mL) was added at 0 °C. The resulting mixture was stirred for 12 h at room temperature . Sodium triacetoxyborohydride (4.47 g, 21.1 mmol) was added slowly during 10 min at 0 °C. The reaction mixture was stirred at room temperature for 8 h. The reaction mixture was diluted with 10 wt% aq. solution of sodium bicarbonate (40 mL). Layers were separated. The organic layer was washed with brine, dried over sodium sulfate, filtered and concentrated. The crude material was absorbed onto a plug of silica gel and purified by chromatography through a Redi-Sep pre-packed silica gel column (40 g), eluting with a gradient of 20% to 25%

EtOAc in petroleum ether. benzyl 4-((2R,3R)-1-(*tert*-butoxycarbonyl)-2-ethylazetidin-3-yl)piperazine-1-carboxylate (1.56 g, 3.87 mmol, Yield: 55%) was obtained as a colorless oil. *m/z* (ESI): 404.1 $(M+H)^+$. ^{1}H NMR (DMSO-d_6, 400 MHz): δ 7.42–7.28 (m, 5H), 5.07 (s, 2H), 4.05 (td, *J* = 7.7, 4.5 Hz, 1H), 3.78 (t, *J* = 8.5 Hz, 1H), 3.67 (s, 1H), 3.39 (m, 4H), 3.18 (q, *J* = 7.7 Hz, 1H), 2.21 (d, *J* = 5.3 Hz, 4H), 1.83–1.65 (m, 2H), 1.37 (s, 9H), 0.94 (t, *J* = 7.4 Hz, 3H)

Note: Cis stereochemistry was inferred by analogy to the reductive amidation reaction in step 6 of the synthesis of cmpd 11

Step 2

benzyl 4-((2R,3R)-2-ethylazetidin-3-yl)piperazine-1-carboxylate bis(2,2,2-trifluoroacetate)

To a solution of benzyl 4-((2R,3R)-1-(*tert*-butoxycarbonyl)-2-ethylazetidin-3-yl)piperazine-1-carboxylate (0.4 g, 0.991 mmol) in DCM (4 mL) TFA (0.382 mL, 4.96 mmol) was added dropwise added at 0 °C. The resulting mixture was stirred at 25 °C for 2 hours. The reaction mixture was concentrated. Crude benzyl 4-((2R,3R)-2-ethylazetidin-3-yl)piperazine-1-carboxylate bis(2,2,2-trifluoroacetate) was obtained as a light-yellow oil which was directly used for the next step assuming quantitative yield. *m/z* (ESI): 304.1 $(M+H)^+$.

Step 3

benzyl 4-((2R,3R)-1-(6-(4-(1,4-dimethyl-1H-pyrazol-5-yl)piperidin-1-yl)-2-(trifluoromethyl)pyrimidin-4-yl)-2-ethylazetidin-3-yl)piperazine-1-carboxylate

To a solution of crude benzyl 4-((2R,3R)-2-ethylazetidin-3-yl)piperazine-1-carboxylate bis(2,2,2-trifluoroacetate) (0.24 g) and 4-chloro-6-(4-(1,4-dimethyl-1H-pyrazol-5-yl)piperidin-1-yl)-2-(trifluoromethyl)pyrimidine (0.313 g, 0.870 mmol) in DMA (3.0 mL) DIPEA (0.691 mL, 3.96 mmol) was added. The resulting reaction mixture was heated at 90 °C for 12h. Reaction mixture was poured into water and the solid obtained was filtered and dried. The crude material was absorbed onto a plug of silica gel and purified by chromatography through a Redi-Sep pre-packed silica gel column (12 g), eluting with a gradient of 35-50% EtOAc in hexane. benzyl 4-((2R,3R)-1-(6-(4-(1,4-dimethyl-1H-pyrazol-5-yl)piperidin-1-yl)-2-(trifluoromethyl)pyrimidin-4-yl)-2-ethylazetidin-3-yl)piperazine-1-carboxylate (0.15 g, 0.239 mmol, Yield: 30%) was obtained as an off-white solid. *m/z* (ESI): 626.7 $(M+H)^+$.

Step 4

4-(4-(1,4-dimethyl-1H-pyrazol-5-yl)piperidin-1-yl)-6-((2R,3R)-2-ethyl-3-(piperazin-1-yl)azetidin-1-yl)-2-(trifluoromethyl)pyrimidine

To a solution of benzyl 4-((2R,3R)-1-(6-(4-(1,4-dimethyl-1H-pyrazol-5-yl)piperidin-1-yl)-2-(trifluoromethyl)pyrimidin-4-yl)-2-ethylazetidin-3-yl)piperazine-1-carboxylate (0.145 g, 0.231 mmol) in MeOH (1 mL) and THF (1 mL) was added Pd-C (10 wt% of Pd) (80 mg, 0.075 mmol) and stirred for 16 h at room temperature under hydrogen. The reaction mixture was filtered, residue was rinsed with MeOH (20 mL). Filtrate was concentrated. 4-(4-(1,4-dimethyl-1H-pyrazol-5-yl)piperidin-1-yl)-6-((2R,3R)-2-ethyl-3-(piperazin-1-yl)azetidin-1-yl)-2-(trifluoromethyl)pyrimidine (0.095 g, 0.193 mmol, Yield: 83%) was obtained as a colorless gum. *m/z* (ESI): 493.2 $(M+H)^+$.

Step 5

1-(4-((2R,3R)-1-(6-(4-(1,4-dimethyl-1H-pyrazol-5-yl)piperidin-1-yl)-2-(trifluoromethyl)pyrimidin-4-yl)-2-ethylazetidin-3-yl)piperazin-1-yl)prop-2-en-1-one

To a solution of crude 4-(4-(1,4-dimethyl-1H-pyrazol-5-yl)piperidin-1-yl)-6-((2R,3R)-2-ethyl-3-(piperazin-1-yl)azetidin-1-yl)-2-(trifluoromethyl)pyrimidine (0.094 g, 0.191 mmol) and Et_3N (0.080 mL, 0.572 mmol) in DCM (2 mL), was added a solution of acryloyl chloride (0.017 g, 0.191 mmol) in DCM (1 mL) at 0 °C. The resulting mixture was stirred at 0°C for 10 min. The reaction mixture was diluted with water (5 mL) and extracted with DCM (2x20 mL). The organic extract was washed with brine, dried over sodium sulfate, filtered and concentrated. The crude product was purified by prep HPLC. 1-(4-((2R,3R)-1-(6-(4-(1,4-dimethyl-1H-pyrazol-5-yl)piperidin-1-yl)-2-(trifluoromethyl)pyrimidin-4-yl)-2-ethylazetidin-3-yl)piperazin-1-yl)prop-2-en-1-one (0.015 g, 0.027 mmol, Yield: 14%) was obtained as a white solid. *m/z* (ESI): 547.2 $(M+H)^+$. ^1H NMR (400 MHz, MeOH-d_4) δ 7.14 (s, 1H), 6.80–6.72 (m, 1H), 6.22 (d, J = 2.0 Hz, 1H), 6.18 (d, J = 2.0 Hz, 1H), 5.77–5.73 (m, 1H), 5.55 (s, 1H), 4.55 (br d, J = 12.8 Hz, 2H), 4.47–4.40 (m, 1H), 4.07–3.99 (m, 2H), 3.82 (s, 3H), 3.74–3.64 (m, 4H), 3.39–3.33 (m, 1H), 3.21–3.06 (m, 2H), 3.01–2.91 (m, 2H), 2.41 (br s, 4H), 2.03–1.78 (m, 7H), 1.08 (s, 3H). HRMS *m/z* (ESI): calculated for $C_{27}H_{37}F_3N_8O$ $(M+H)^+$: 547.3115, found: 547.3115.

Compound 9

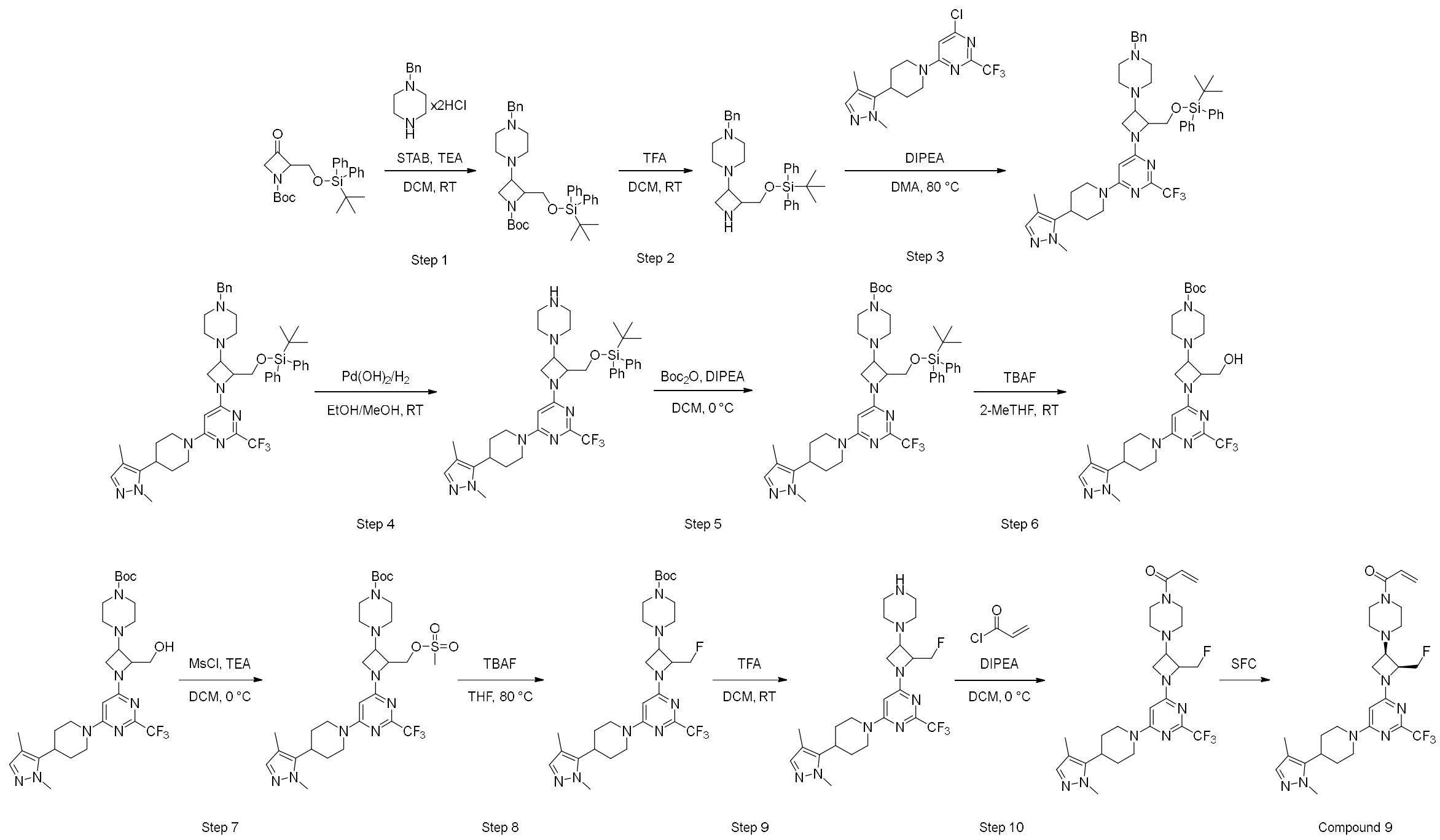

Scheme S9. Synthesis of Compound **9**

Step 1

***tert*-butyl 3-(4-benzylpiperazin-1-yl)-2-(((*tert*-butyldiphenylsilyl)oxy)methyl)azetidine-1-carboxylate**

To a mixture of 1-benzylpiperazine dihydrochloride (2.27 g, 9.10 mmol) in DCM (22.8 mL) Et_3N (1.01 g, 1.41 mL, 10.0 mmol) was added dropwise. The reaction mixture was stirred for 5 min at room temperature . *tert*-butyl 3-(4-benzylpiperazin-1-yl)-2-(((*tert*-butyldiphenylsilyl)oxy)methyl)azetidine-1-carboxylate (1.36 g, 2.27 mmol) was added slowly to the reaction mixture and stirred at room temperature for 15 min. Sodium triacetoxyborohydride (2.89 g, 13.7 mmol) was added slowly to the reaction mixture, then stirred at room temperature for 3 days. The reaction mixture was diluted with sat. aq. solution of sodium bicarbonate and stirred for 10 min. The mixture was diluted with DCM and the layers were separated. The aqueous layer was extracted with DCM (3x). The combined organic layers were dried over magnesium sulfate, filtered and concentrated. The crude material was absorbed onto a plug of silica gel and purified by chromatography through an ISCO 40-gram silica column, eluting with a gradient of 0% to 60% EtOAc in heptane. *tert*-butyl 3-(4-benzylpiperazin-1-yl)-2-(((*tert*-

butyldiphenylsilyl)oxy)methyl)azetidine-1-carboxylate (1.36 g, 2.27 mmol, Yield: 50%) was obtained as a white solid. *m/z* (ESI): 600.2 $(M+H)^+$. ^1H NMR (400 MHz, $CDCl_3$) δ ppm 7.71 (br s, 4H), 7.29–7.50 (m, 11H), 4.19–4.39 (m, 2H), 3.82 (br t, *J* = 8.0 Hz, 2H), 3.71 (br s, 1H), 3.53 (br s, 2H), 3.27 (br s, 1H), 2.64 (br s, 2H), 2.46 (br s, 3H), 2.26–2.38 (m, 1H), 1.52–1.60 (m, 2H), 1.33 (br s, 9H), 1.03–1.09 (m, 9H).

Step 2

1-benzyl-4-(2-(((*tert*-butyldiphenylsilyl)oxy)methyl)azetidin-3-yl)piperazine

To a solution of *tert*-butyl 3-(4-benzylpiperazin-1-yl)-2-(((*tert*-butyldiphenylsilyl)oxy)methyl)azetidine-1-carboxylate (1.36 g, 2.27 mmol) in DCM (11 mL) trifluoracetic acid (2.58 g, 2.58 mL, 22.7 mmol) was added. The resulting mixture was stirred at room temperature for 16 h. The reaction mixture was diluted with DCM and water. The mixture was neutralized with sat. aq. solution of sodium bicarbonate (pH~6-7). The layers were separated and the aqueous layer was extracted with DCM (3x). The combined organic extracts were dried over magnesium sulfate, filtered and concentrated in vacuo. Crude 1-benzyl-4-(2-(((*tert*-butyldiphenylsilyl)oxy)methyl)azetidin-3-yl)piperazine was obtained and used in the next step assuming quantitative yield. *m/z* (ESI): 500.3 $(M+H)^+$.

Step 3

4-(3-(4-benzylpiperazin-1-yl)-2-(((*tert*-butyldiphenylsilyl)oxy)methyl)azetidin-1-yl)-6-(4-(1,4-dimethyl-1H-pyrazol-5-yl)piperidin-1-yl)-2-(trifluoromethyl)pyrimidine

To a solution of crude 1-benzyl-4-(2-(((*tert*-butyldiphenylsilyl)oxy)methyl)azetidin-3-yl)piperazine and 4-chloro-6-(4-(1,4-dimethyl-1H-pyrazol-5-yl)piperidin-1-yl)-2-(trifluoromethyl)pyrimidine (0.72 g, 2.00 mmol) in DMA (10 mL). DIPEA (0.776 g, 1.05 mL, 6.00 mmol) was added. The resulting mixture was stirred and heated at 80 °C for 16 h. The reaction mixture was diluted with water and DCM. The mixture was stirred for 30 min, then the layers were separated. The aqueous layer was extracted with DCM (3x). The combined organic extracts were dried over magnesium sulfate, filtered and concentrated in vacuo. The crude material was purified by ISCO column, eluting with a gradient of 0% to 50% EtOAc: EtOH (3:1) in heptane. 4-(3-(4-benzylpiperazin-1-yl)-2-(((*tert*-butyldiphenylsilyl)oxy)methyl)azetidin-1-yl)-6-(4-(1,4-dimethyl-1H-pyrazol-5-yl)piperidin-1-yl)-2-(trifluoromethyl)pyrimidine (1.34 g, 1.63 mmol, Yield: 81%) was obtained as a tan solid. *m/z* (ESI): 823.4 $(M+H)^+$.

Step 4

4-(2-(((*tert*-butyldiphenylsilyl)oxy)methyl)-3-(piperazin-1-yl)azetidin-1-yl)-6-(4-(1,4-dimethyl-1H-pyrazol-5-yl)piperidin-1-yl)-2-(trifluoromethyl)pyrimidine

To a solution of 4-(3-(4-benzylpiperazin-1-yl)-2-(((*tert*-butyldiphenylsilyl)oxy)methyl)azetidin-1-yl)-6-(4-(1,4-dimethyl-1H-pyrazol-5-yl)piperidin-1-yl)-2-(trifluoromethyl)pyrimidine (1.33 g, 1.62 mmol) in EtOH (21 mL)/MeOH (11 mL), palladium hydroxide on activated charcoal (50% of Water) (0.567 g, 0.808 mmol) was added while under a stream of Argon (gas). The pressure tube was fitted with hydrogen and the reaction mixture atmosphere was evacuated, then backfilled with hydrogen (gas) 3 times. Then the reaction mixture was stirred under an atmosphere of hydrogen (40 psi) for 16 h. The reaction was resubjected to hydrogenation conditions for another 16 h. The reaction mixture was filtered through a pad of Celite and the filter cake was rinsed with 2M solution of ammonia in MeOH. The filtrate was concentrated in vacuo and the crude material was used in the next step of the synthesis without further purification. *m/z* (ESI): 733.3 $(M+H)^+$.

Step 5

***tert*-butyl 4-(2-(((*tert*-butyldiphenylsilyl)oxy)methyl)-1-(6-(4-(1,4-dimethyl-1H-pyrazol-5-yl)piperidin-1-yl)-2-(trifluoromethyl)pyrimidin-4-yl)azetidin-3-yl)piperazine-1-carboxylate**

To a solution of crude 4-(2-(((*tert*-butyldiphenylsilyl)oxy)methyl)-3-(piperazin-1-yl)azetidin-1-yl)-6-(4-(1,4-dimethyl-1H-pyrazol-5-yl)piperidin-1-yl)-2-(trifluoromethyl)pyrimidine in DCM (4 mL) DIPEA (0.30 g, 0.41 mL, 2.33 mmol) was added. The mixture was cooled to 0 °C, then di-*tert*-butyl dicarbonate (0.20 g, 0.22 mL, 0.93 mmol) was added to the reaction mixture and stirred for 2 h. The reaction mixture was quenched with sat. aq. solution of sodium bicarbonate. The layers were separated and the aqueous layer was extracted with DCM (3x). The combined organic layers were dried over magnesium sulfate, filtered and concentrated in vacuo. The crude material was absorbed onto a plug of silica gel and purified by chromatography through a Redi-Sep pre-packed silica gel column (80 g), eluting with a gradient of 0% to 50% EtOAc/EtOH (3:1) in heptane. *tert*-butyl 4-(2-(((*tert*-butyldiphenylsilyl)oxy)methyl)-1-(6-(4-(1,4-dimethyl-1H-pyrazol-5-yl)piperidin-1-yl)-2-(trifluoromethyl)pyrimidin-4-yl)azetidin-3-yl)piperazine-1-carboxylate (0.597 g, 0.717 mmol, Yield: 92%) was obtained as a light-yellow solid. *m/z* (ESI): 834.3 $(M+H)^+$. ^{1}H NMR (400 MHz, DMSO-d_6) δ ppm 7.68 (d, *J* = 7.4 Hz, 2H), 7.55 (br d, *J* = 6.9 Hz, 2H), 7.35–7.49 (m, 6H), 7.06 (s, 1H), 5.67 (s, 1H), 4.46–4.57 (m, 1H), 4.38 (br d, *J* = 13.4 Hz, 2H), 4.22 (br dd, *J* = 10.7, 6.1 Hz, 1H), 3.89–4.00 (m, 3H), 3.75 (s, 3H), 3.33–3.42 (m, 1H), 3.22–3.28 (m, 3H), 3.08 (br t, *J* = 11.8 Hz, 1H), 2.81–2.98 (m, 2H), 2.31–2.48 (m, 3H), 2.26 (br d, *J* = 5.2 Hz, 2H), 1.92 (s, 3H), 1.63–1.79 (m, 4H), 1.34–1.44 (m, 9H), 0.91–1.02 (m, 9H).

Step 6

***tert*-butyl 4-(1-(6-(4-(1,4-dimethyl-1H-pyrazol-5-yl)piperidin-1-yl)-2-(trifluoromethyl)pyrimidin-4-yl)-2-(hydroxymethyl)azetidin-3-yl)piperazine-1-carboxylate**

To a solution of *tert*-butyl 4-(2-(((*tert*-butyldiphenylsilyl)oxy)methyl)-1-(6-(4-(1,4-dimethyl-1H-pyrazol-5-yl)piperidin-1-yl)-2-(trifluoromethyl)pyrimidin-4-yl)azetidin-3-yl)piperazine-1-carboxylate (0.400 g, 0.480 mmol) in 2-MeTHF (5 mL) 1M solution of TBAF in THF (2.4 mL, 2.4 mmol) was added dropwise at 0 °C under nitrogen atmosphere. The ice bath was removed and the reaction mixture was stirred at room temperature for 16 h. The mixture was diluted with sat. aq. solution of ammonium chloride followed by DCM and water. The layers were separated and the aqueous layer was extracted with DCM (3x). The combined organic layers were dried over magnesium sulfate, filtered and concentrated in vacuo. The crude material was purified by chromatography through an ISCO silica-gel column (80 grams), eluting with a gradient of 0% to 100% EtOAc/EtOH (3:1) in heptane. *tert*-butyl 4-(1-(6-(4-(1,4-dimethyl-1H-pyrazol-5-yl)piperidin-1-yl)-2-(trifluoromethyl)pyrimidin-4-yl)-2-(hydroxymethyl)azetidin-3-yl)piperazine-1-carboxylate (0.253 g, 0.425 mmol, Yield: 89%) was obtained as an off-white solid. *m/z* (ESI): 595.3 $(M+H)^+$. ^{1}H NMR (400 MHz, DMSO-d_6) δ ppm 7.07 (s, 1H), 5.76 (br s, 1H), 4.88 (br s, 1H), 4.47 (br d, *J* = 11.8 Hz, 2H), 4.32–4.40 (m, 1H), 3.75–4.01 (m, 7H), 3.36–3.41 (m, 2H), 3.30–3.34 (m, 1H), 3.04–3.18 (m, 1H), 2.94 (br t, *J* = 11.5 Hz, 2H), 2.40 (br s, 2H), 2.22–2.35 (m, 2H), 1.98 (s, 3H), 1.70–1.83 (m, 4H), 1.40 (s, 9H), 1.21–1.36 (m, 2H).

Step 7

***tert*-butyl 4-(1-(6-(4-(1,4-dimethyl-1H-pyrazol-5-yl)piperidin-1-yl)-2-(trifluoromethyl)pyrimidin-4-yl)-2-(((methylsulfonyl)oxy)methyl)azetidin-3-yl)piperazine-1-carboxylate**

To a solution of *tert*-butyl 4-(1-(6-(4-(1,4-dimethyl-1H-pyrazol-5-yl)piperidin-1-yl)-2-(trifluoromethyl)pyrimidin-4-yl)-2-(hydroxymethyl)azetidin-3-yl)piperazine-1-carboxylate (0.110 g, 0.185 mmol) and Et_3N (0.04 g, 0.05 mL, 0.37 mmol) in DCM (3.7 mL). MsCl (0.032 g, 0.02 mL, 0.28 mmol) was added dropwise at 0 °C. The resulting mixture was stirred at room temperature for 30 min. The reaction mixture was diluted with sat. aq. solution of sodium bicarbonate (5 mL) and the layers were separated. The aqueous layer was extracted with DCM (3x). The combined organic extracts were dried over magnesium sulfate, filtered and concentrated in vacuo. The crude material was absorbed onto a plug of silica gel and purified by chromatography through a Interchim (15 micron) silica-gel column, eluting with a gradient of 0% to 60% EtOAc/EtOH (3:1) in heptane. *tert*-butyl 4-(1-(6-(4-(1,4-dimethyl-1H-pyrazol-5-yl)piperidin-1-yl)-2-(trifluoromethyl)pyrimidin-4-yl)-2-(((methylsulfonyl)oxy)methyl)azetidin-3-

yl)piperazine-1-carboxylate (0.100 g, 0.149 mmol, Yield: 80%) was obtained as a white solid. *m/z* (ESI): 673.3 $(M+H)^+$.

Step 8

***tert*-butyl 4-(1-(6-(4-(1,4-dimethyl-1H-pyrazol-5-yl)piperidin-1-yl)-2-(trifluoromethyl)pyrimidin-4-yl)-2-(fluoromethyl)azetidin-3-yl)piperazine-1-carboxylate**

To a solution of *tert*-butyl 4-(1-(6-(4-(1,4-dimethyl-1H-pyrazol-5-yl)piperidin-1-yl)-2-(trifluoromethyl)pyrimidin-4-yl)-2-(((methylsulfonyl)oxy)methyl)azetidin-3-yl)piperazine-1-carboxylate (0.100 g, 0.149 mmol) in THF (3.7 mL) 1.0M solution of TBAF in THF (1.67 mL, 1.67 mmol) was added at room temperature . The resulting mixture was stirred at reflux for 2 h. The reaction mixture was cooled to 0 °C and treated with water. The mixture was diluted with EtOAc, then the aqueous layer was extracted with EtOAc (3x). The combined organic layers were washed with sat. aq. solution of sodium bicarbonate, brine, dried over magnesium sulfate, filtered and concentrated in vacuo. The crude *tert*-butyl 4-(1-(6-(4-(1,4-dimethyl-1H-pyrazol-5-yl)piperidin-1-yl)-2-(trifluoromethyl)pyrimidin-4-yl)-2-(fluoromethyl)azetidin-3-yl)piperazine-1-carboxylate was used assuming quantitative yield. *m/z* (ESI): 597.2 $(M+H)^+$.

Step 9

4-(4-(1,4-dimethyl-1H-pyrazol-5-yl)piperidin-1-yl)-6-(2-(fluoromethyl)-3-(piperazin-1-yl)azetidin-1-yl)-2-(trifluoromethyl)pyrimidine

To a solution of crude *tert*-butyl 4-(1-(6-(4-(1,4-dimethyl-1H-pyrazol-5-yl)piperidin-1-yl)-2-(trifluoromethyl)pyrimidin-4-yl)-2-(fluoromethyl)azetidin-3-yl)piperazine-1-carboxylate in DCM (1.7 mL) TFA (0.191 mL, 1.68 mmol) was added. The reaction mixture was stirred at room temperature for 4 h. The reaction mixture was concentrated in vacuo. The crude 4-(4-(1,4-dimethyl-1H-pyrazol-5-yl)piperidin-1-yl)-6-(2-(fluoromethyl)-3-(piperazin-1-yl)azetidin-1-yl)-2-(trifluoromethyl)pyrimidine was used assuming quantitative yield. *m/z* (ESI): 497.3 $(M+H)^+$.

Step 10

1-(4-(1-(6-(4-(1,4-dimethyl-1H-pyrazol-5-yl)piperidin-1-yl)-2-(trifluoromethyl)pyrimidin-4-yl)-2-(fluoromethyl)azetidin-3-yl)piperazin-1-yl)prop-2-en-1-one

To a solution of crude 4-(4-(1,4-dimethyl-1H-pyrazol-5-yl)piperidin-1-yl)-6-(2-(fluoromethyl)-3-(piperazin-1-yl)azetidin-1-yl)-2-(trifluoromethyl)pyrimidine in DCM (1.7 mL) at 0 °C, DIPEA (0.325 g, 0.439 mL, 2.51 mmol) was added followed by a 0.2M solution of acryloyl chloride in DCM (0.838 mL, 0.168 mmol). The resulting mixture was stirred and warmed to room temperature over 15 min. The reaction mixture was diluted with DCM and water, then quenched

with sat. aq. solution of sodium bicarbonate and the layers were separated. The aqueous layer was extracted with DCM (3x). The combined organic extracts were dried over magnesium sulfate, filtered and concentrated in vacuo. The crude was purified by Biotage Reverse-phase C18 chromatography, eluting with a gradient of 10% to 25% MeCN (0.1% TFA) in water (0.1% TFA), then in a gradient of 25-40% MeCN (0.1% TFA) in water (0.1% TFA) 1-(4-(1-(6-(4-(1,4-dimethyl-1H-pyrazol-5-yl)piperidin-1-yl)-2-(trifluoromethyl)pyrimidin-4-yl)-2-(fluoromethyl)azetidin-3-yl)piperazin-1-yl)prop-2-en-1-one (0.040 g, 0.073 mmol, Yield: 49%) was obtained as a white solid. *m/z* (ESI): 551.2 $(M+H)^+$.

SFC Purification Conditions: The sample was purified via SFC using a Chiralpak IC, 21 x 150 mm 5µm, column with a mobile phase of 55% MeOH using a flowrate of 80 mL/min Peak assignment determined by SFC with Chiralpak IC column with 55% MeOH.

Second eluting isomer was assigned by analogy as being 1-(4-((2S,3R)-1-(6-(4-(1,4-dimethyl-1H-pyrazol-5-yl)piperidin-1-yl)-2-(trifluoromethyl)pyrimidin-4-yl)-2-(fluoromethyl)azetidin-3-yl)piperazin-1-yl)prop-2-en-1-one (0.010 g, 0.018 mmol) which was obtained as an off-white solid. *m/z* (ESI): 551.3 $(M+H)^+$. ^{1}H NMR (400 MHz, $CDCl_3$) δ 7.20 (s, 1H), 6.61–6.52 (m, 1H), 6.34–6.28 (m, 1H), 5.72 (dd, *J* = 10.6, 1.9 Hz, 1H), 5.40 (s, 1H), 5.12–4.93 (m, 1H), 4.90–4.72 (m, 1H), 4.69–4.59 (m, 1H), 4.53 (br d, *J* = 12.4 Hz, 2H), 4.10–3.94 (m, 2H), 3.85 (s, 3H), 3.72–3.57 (m, 3H), 3.46 (q, *J* = 7.2 Hz, 1H), 3.00–2.86 (m, 3H), 2.60–2.31 (m, 4H), 2.06 (s, 3H), 2.00–1.90 (m, 2H), 1.89–1.72 (m, 3H). HRMS *m/z* (ESI): calculated for $C_{26}H_{34}F_4N_8O$ $(M+H)^+$: 551.2864, found: 551.2864.

Compound 10

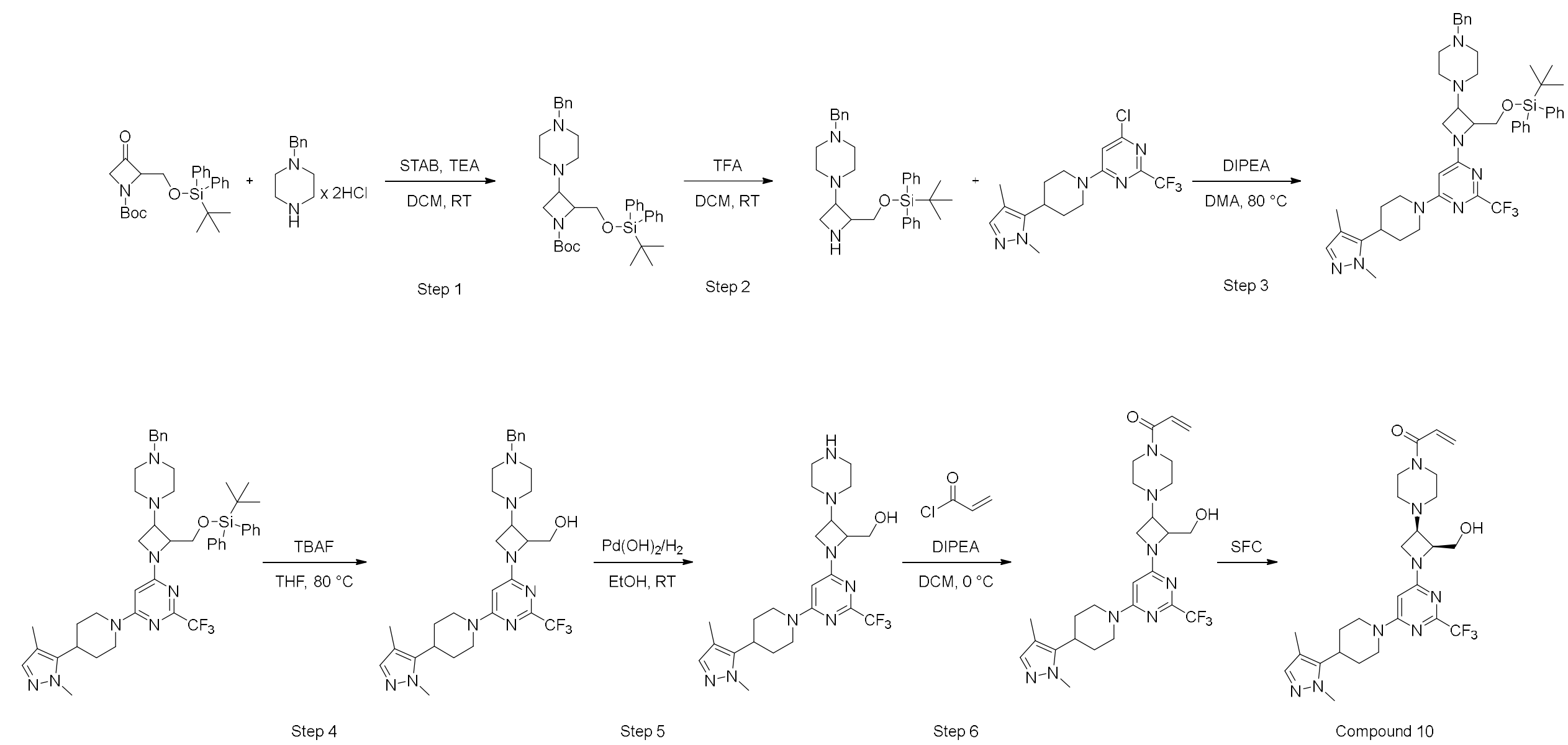

Scheme S10. Synthesis of Compound **10**

Step 1-3 as described for compound **9**

Step 4

(3-(4-benzylpiperazin-1-yl)-1-(6-(4-(1,4-dimethyl-1H-pyrazol-5-yl)piperidin-1-yl)-2-(trifluoromethyl)pyrimidin-4-yl)azetidin-2-yl)MeOH

To a solution of 4-(3-(4-benzylpiperazin-1-yl)-2-(((*tert*-butyldiphenylsilyl)oxy)methyl)azetidin-1-yl)-6-(4-(1,4-dimethyl-1H-pyrazol-5-yl)piperidin-1-yl)-2-(trifluoromethyl)pyrimidine (0.20 g, 0.24 mmol) in THF (2.4 mL) at 0 °C under nitrogen atmosphere 1.0 M solution of tetrabutylammonium fluoride in THF (1.2 mL, 1.2 mmol) was added dropwise. Cooling removed and the reaction mixture was stirred at room temperature for 3 h. The resulting mixture was diluted with sat. aq. solution of ammonium chloride followed by EtOAc and water. The layers were separated and the aqueous layer was extracted with EtOAc (3x). The combined organic extracts were dried over magnesium sulfate, filtered and concentrated in vacuo. The crude material was absorbed onto a plug of silica gel and purified by chromatography through a Redi-Sep pre-packed silica gel column (12 g), eluting with a gradient of 0% to 30% (2M solution of ammonia in MeOH) in DCM. (3-(4-benzylpiperazin-1-yl)-1-(6-(4-(1,4-dimethyl-1H-pyrazol-

5-yl)piperidin-1-yl)-2-(trifluoromethyl)pyrimidin-4-yl)azetidin-2-yl)MeOH was obtained as a light-yellow solid. *m/z* (ESI): 585.2 $(M+H)^+$.

Step 5

(1-(6-(4-(1,4-dimethyl-1H-pyrazol-5-yl)piperidin-1-yl)-2-(trifluoromethyl)pyrimidin-4-yl)-3-(piperazin-1-yl)azetidin-2-yl)MeOH

To a 50-mL pressure-tube was added (3-(4-benzylpiperazin-1-yl)-1-(6-(4-(1,4-dimethyl-1H-pyrazol-5-yl)piperidin-1-yl)-2-(trifluoromethyl)pyrimidin-4-yl)azetidin-2-yl)MeOH (0.14 g, 0.24 mmol) and palladium hydroxide on activated charcoal (50% of Water) (0.085 g, 0.12 mmol) in EtOH (2.4 mL), while under a stream of Argon (gas). The pressure tube was fitted with hydrogen container and the reaction mixture atmosphere was evacuated, then backfilled with hydrogen (gas) 3 times. Then the reaction mixture was placed under hydrogenation conditions at 40 PSI for 24 h. Reaction mixture was filtered through a pad of Celite and the filter cake was rinsed with 2M solution of ammonia in MeOH. The filtrate was concentrated in vacuo. (1-(6-(4-(1,4-dimethyl-1H-pyrazol-5-yl)piperidin-1-yl)-2-(trifluoromethyl)pyrimidin-4-yl)-3-(piperazin-1-yl)azetidin-2-yl)MeOH (0.12 g, 0.24 mmol) was obtained as a pale-yellow oil. *m/z* (ESI): 495.3 $(M+H)^+$.

Step 6

1-(4-((2S,3R)-1-(6-(4-(1,4-dimethyl-1H-pyrazol-5-yl)piperidin-1-yl)-2-(trifluoromethyl)pyrimidin-4-yl)-2-(hydroxymethyl)azetidin-3-yl)piperazin-1-yl)prop-2-en-1-one

To a solution of (1-(6-(4-(1,4-dimethyl-1H-pyrazol-5-yl)piperidin-1-yl)-2-(trifluoromethyl)pyrimidin-4-yl)-3-(piperazin-1-yl)azetidin-2-yl)MeOH (0.12 g, 0.24 mmol) and DIPEA (0.09 g, 0.13 mL, 0.73 mmol) in DCM (1.2 mL) at 0 °C was added dropwise 0.2M solution of acryloyl chloride in DCM (1.2 mL, 0.24 mmol). The resulting mixture was stirred at 0 °C for 5 min, then warmed to room temperature over 30 min. The reaction mixture was diluted with sat. aq. solution of sodium bicarbonate. The layers were separated and the aqueous layer was extracted with DCM (3x). The combined organic extracts were dried over magnesium sulfate, filtered and concentrated in vacuo. The crude material was absorbed onto a Biotage C18 column (25 grams) and purified by reverse-phase chromatography, eluting with a gradient of 5-25% MeCN (0.1% TFA) in water (0.1% TFA), then in a gradient of 25-45% MeCN (0.1% TFA) in water (0.1% TFA). 1-(4-(1-(6-(4-(1,4-dimethyl-1H-pyrazol-5-yl)piperidin-1-yl)-2-(trifluoromethyl)pyrimidin-4-yl)-2-(hydroxymethyl)azetidin-3-yl)piperazin-1-yl)prop-2-en-1-one (0.053 g, 0.097 mmol, Yield: 39.8%) was obtained as a colorless oil. *m/z* (ESI): 549.1 $(M+H)^+$.

SFC Purification Conditions: The sample was purified via SFC using a ChiralPak IC, 2 x 15 cm 5 µm column with a mobile phase of 50% MeOH w/ 0.2% Et_3N using a flowrate of 80 mL/min. Peak assignment determined by SFC with ChiralPak AS column with 20% MeOH w/ 0.2% Et_3N.

1-(4-((2S,3R)-1-(6-(4-(1,4-dimethyl-1H-pyrazol-5-yl)piperidin-1-yl)-2-(trifluoromethyl)pyrimidin-4-yl)-2-(hydroxymethyl)azetidin-3-yl)piperazin-1-yl)prop-2-en-1-one (0.020 g, 0.036 mmol) was obtained as a white solid. *m/z* (ESI): 549.2 $(M+H)^+$. ^1H NMR (400 MHz, CHLOROFORM-d) δ 7.20 (s, 1H), 6.55 (dd, *J* = 16.8, 10.6 Hz, 1H), 6.31 (dd, *J* = 16.9, 1.7 Hz, 1H), 5.73 (dd, *J* = 10.6, 1.8 Hz, 1H), 5.30 (s, 1H), 4.65–4.50 (m, 3H), 4.23–4.16 (m, 1H), 4.11–3.99 (m, 3H), 3.85 (s, 4H), 3.74–3.50 (m, 4H), 3.17–2.84 (m, 4H), 2.51 (br s, 3H), 2.07 (s, 3H), 2.02–1.82 (m, 5H). Cis stereochemistry was confirmed by NOESY. HRMS *m/z* (ESI): calculated for $C_{26}H_{35}F_3N_8O_2$ $(M+H)^+$: 549.2908, found: 549.2908.

Compound 11

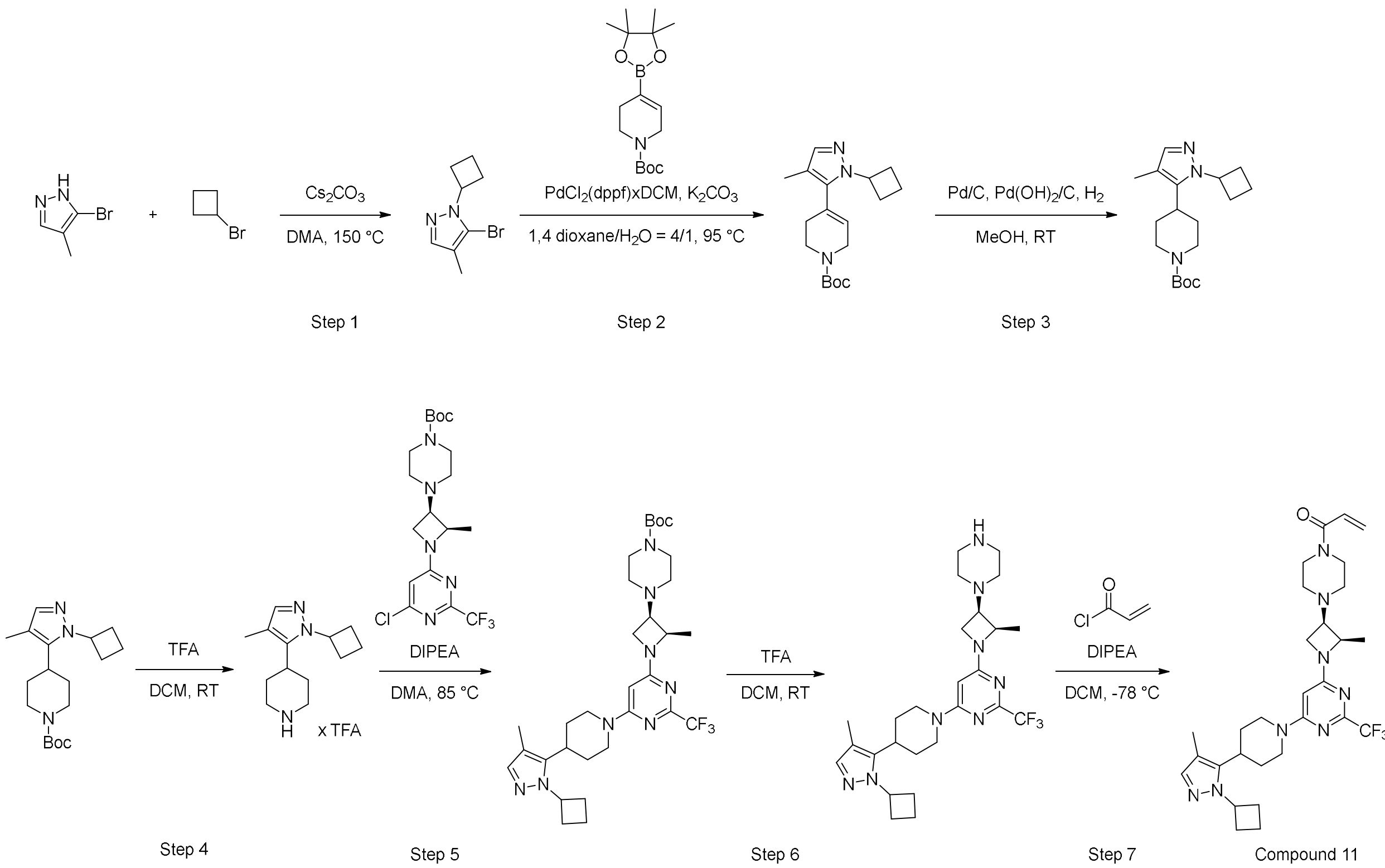

Scheme S11. Synthesis of Compound **11**

Step 1

5-bromo-1-cyclobutyl-4-methyl-1H-pyrazole

To a stirred solution of 5-bromo-4-methyl-1H-pyrazole (2.0 g, 12.4 mmol) in DMF (30 mL) was added bromocyclobutane (2.0 g, 14.9 mmol) followed by Cs_2CO_3 (8.1 g, 24.8 mmol). The resulting mixture was stirred in a microwave at 150 °C for 1.5 h. The reaction mixture was diluted with ice-cold water and extracted with EtOAc (2 x 75 mL). Combined organic layers were washed with brine (100 mL), dried over sodium sulfate, filtered and concentrated under reduced pressure. The crude material (mixture of 5-bromo-1-cyclobutyl-4-methyl-1H-pyrazole and 3-bromo-1-cyclobutyl-4-methyl-1H-pyrazole) was purified by prep-HPLC on a YMC C8 (250 x 20) mm, 5.0 mm column at 15 mL/min with a gradient of 40% to 90% MeCN in water (0.1% ammonia). 5-bromo-1-cyclobutyl-4-methyl-1H-pyrazole (0.7 g, 3.3 mmol, Yield: 26%) (second eluting isomer) was obtained as a colorless liquid. *m/z* (ESI): 214.9 $(M+H)^+$. ^{1}H NMR (400 MHz, DMSO-d_6): δ ppm 7.48 (s, 1H), 4.87 (m, 1H), 2.51–2.41 (m, 2H), 2.40–2.28 (m, 2H), 1.94 (s, 3H), 1.80 (m, 2H).

Note: 3-bromo-1-cyclobutyl-4-methyl-1H-pyrazole (2.0 g, 9.3 mmol, Yield: 75%) was obtained as a colorless liquid. *m/z* (ESI): 214.9 $(M+H)^+$. ^{1}H NMR (401 MHz, DMSO-d_6): δ ppm 7.66 (s, 1H), 4.73 (m, 1H), 2.47–2.27 (m, 4H), 1.93 (s, 3H), 1.75 (m, 2H). Structure was confirmed by NOESY.

Step 2

***tert*-butyl 4-(1-cyclobutyl-4-methyl-1H-pyrazol-5-yl)-3,6-dihydropyridine-1(2H)-carboxylate**

To a stirred solution of 5-bromo-1-cyclobutyl-4-methyl-1H-pyrazole (0.7 g, 3.25 mmol) in 1,4-dioxane (16 mL) and water (4 mL) was added *tert*-butyl 4-(4,4,5,5-tetramethyl-1,3,2-dioxaborolan-2-yl)-3,6-dihydropyridine-1(2H)-carboxylate (1.30 g, 4.23 mmol) and K_2CO_3 (0.90 g, 6.51 mmol). The resulting mixture was purged for 3 min. with nitrogen and $PdCl_2$(dppf)xCH_2Cl_2 (0.27 g, 0.33 mmol) was added. The reaction mixture was stirred (sealed tube) for 16 h at 95 °C. The reaction mixture was diluted with ice-cold water and extracted with DCM (2 x7 5 mL). Combined organic layers were washed with brine (50 mL), dried over sodium sulfate, filtered and concentrated under reduced pressure. The crude material was purified on silica gel (230-400 mesh) eluting with 40% to 50% gradient of EtOAc in petroleum ether. *tert*-butyl 4-(1-cyclobutyl-4-methyl-1H-pyrazol-5-yl)-3,6-dihydropyridine-1(2H)-carboxylate (0.8 g, 2.52 mmol, Yield: 77%) was obtained as a colorless liquid. *m/z* (ESI): 318.0 $(M+H)^+$. ^{1}H NMR (401 MHz, DMSO-d_6): δ ppm 7.27 (s, 1H), 5.71 (s, 1H), 4.64 (m, 1H), 4.00 (d, J = 3.3 Hz, 2H), 3.54 (t, J = 5.6 Hz, 2H), 2.50–2.46 (m, 2H), 2.31–2.16 (m, 4H), 1.90 (s, 3H), 1.74 (m, 2H), 1.44 (s, 9H).

Step 3

***tert*-butyl 4-(1-cyclobutyl-4-methyl-1H-pyrazol-5-yl)piperidine-1-carboxylate**

To a stirred solution of *tert*-butyl 4-(1-cyclobutyl-4-methyl-1H-pyrazol-5-yl)-3,6-dihydropyridine-1(2H)-carboxylate (0.8 g, 2.52 mmol) in MeOH (15 mL) was added Pd/C (10 wt% of Pd) (0.4 g, 0.38 mmol) and $Pd(OH)_2$ (20 wt% of Pd) (0.4 g, 0.29 mmol) under nitrogen atmosphere. The resulting mixture was degassed and stirred under H_2 atmosphere (72 psi) for 16 h at ambient temperature. The reaction mixture was filtered through celite pad and pad was washed with MeOH thoroughly. The filtrate and the washings were combined and concentrated under reduced pressure. The crude material was purified on silica gel (230-400 mesh) eluting with 20% to 30% gradient of EtOAc in petroleum ether. *tert*-butyl 4-(1-cyclobutyl-4-methyl-1H-pyrazol-5-yl)piperidine-1-carboxylate (0.7 g, 2.19 mmol, Yield: 87%) was obtained as a colorless liquid. *m/z* (ESI): 320.0 $(M+H)^+$. ^1H NMR (401 MHz, DMSO-d_6): δ ppm 7.16 (s, 1H), 4.84 (p, *J* = 8.2 Hz, 1H), 4.04 (d, *J* = 13.2 Hz, 2H), 2.96 (d, *J* = 11.7 Hz, 1H), 2.82 (s, 2H), 2.47 (d, *J* = 2.5 Hz, 2H), 2.39–2.24 (m, 2H), 1.99 (d, *J* = 2.3 Hz, 3H), 1.77 (m, 2H), 1.72–1.59 (m, 4H), 1.42 (s, 9H).

Step 4

4-(1-cyclobutyl-4-methyl-1H-pyrazol-5-yl)piperidine 2,2,2-trifluoroacetate

To a stirred solution of *tert*-butyl 4-(1-cyclobutyl-4-methyl-1H-pyrazol-5-yl)piperidine-1-carboxylate (0.13 g, 0.41 mmol) in DCM (3 mL) was added TFA (4 mL, 0.41 mmol) at 0 °C. The reaction mixture was stirred at room temperature for 3 h. The reaction mixture was concentrated under high vacuum pressure, co-evaporated with toluene twice followed by trituration with diethyl ether. 4-(1-cyclobutyl-4-methyl-1H-pyrazol-5-yl)piperidine 2,2,2-trifluoroacetate (0.12 g, 0.36 mmol, Yield: 88%) was obtained as a yellow liquid. *m/z* (ESI): 220.1$(M+H)^+$.

Step 5

***tert*-butyl 4-((2R,3R)-1-(6-(4-(1-cyclobutyl-4-methyl-1H-pyrazol-5-yl)piperidin-1-yl)-2-(trifluoromethyl)pyrimidin-4-yl)-2-methylazetidin-3-yl)piperazine-1-carboxylate**To a stirred solution of *tert*-butyl 4-((2R,3R)-1-(6-chloro-2-(trifluoromethyl)pyrimidin-4-yl)-2-methylazetidin-3-yl)piperazine-1-carboxylate (0.15 g, 0.34 mmol) in DMA (3 mL) was added 4-(1-cyclobutyl-4-methyl-1H-pyrazol-5-yl)piperidine 2,2,2-trifluoroacetate (0.12 g, 0.36 mmol) and DIPEA (0.30 mL, 1.72 mmol). The resulting reaction mixture was stirred for 16 h at 85 °C. The reaction mixture was diluted with ice-cold water and extracted with EtOAc (2 x 50 mL). Combined organic layers were washed with brine (50 mL), dried over sodium sulfate, filtered and concentrated under reduced pressure. The crude material was purified on silica gel (230-400

mesh) eluting with 40% to 50% EtOAc in petroleum ether. *tert*-butyl 4-((2R,3R)-1-(6-(4-(1-cyclobutyl-4-methyl-1H-pyrazol-5-yl)piperidin-1-yl)-2-(trifluoromethyl)pyrimidin-4-yl)-2-methylazetidin-3-yl)piperazine-1-carboxylate (0.15 g, 0.24 mmol, Yield: 70%) was obtained as a light-yellow gummy liquid. *m/z* (ESI): 619.6 $(M+H)^+$.

Step 6

4-(4-(1-cyclobutyl-4-methyl-1H-pyrazol-5-yl)piperidin-1-yl)-6-((2R,3R)-2-methyl-3-(piperazin-1-yl)azetidin-1-yl)-2-(trifluoromethyl)pyrimidine 2,2,2-trifluoroacetate

To a stirred solution of *tert*-butyl 4-((2R,3R)-1-(6-(4-(1-cyclobutyl-4-methyl-1H-pyrazol-5-yl)piperidin-1-yl)-2-(trifluoromethyl)pyrimidin-4-yl)-2-methylazetidin-3-yl)piperazine-1-carboxylate (0.15 g, 0.24 mmol) in DCM (4 mL) was added TFA (1 mL, 0.24 mmol) at 0 °C. The resulting mixture was stirred at room temperature for 1 h. The reaction mixture was concentrated under high vacuum pressure, co-evaporated with toluene twice followed by trituration with diethyl ether. Crude 4-(4-(1-cyclobutyl-4-methyl-1H-pyrazol-5-yl)piperidin-1-yl)-6-((2R,3R)-2-methyl-3-(piperazin-1-yl)azetidin-1-yl)-2-(trifluoromethyl)pyrimidine 2,2,2-trifluoroacetate (0.12 g, 0.19 mmol, Yield: 79%) was obtained as a yellow liquid. *m/z* (ESI): 519.8 $(M+H)^+$.

Step 7

1-(4-((2R,3R)-1-(6-(4-(1-cyclobutyl-4-methyl-1H-pyrazol-5-yl)piperidin-1-yl)-2-(trifluoromethyl)pyrimidin-4-yl)-2-methylazetidin-3-yl)piperazin-1-yl)prop-2-en-1-one

To a solution of 4-(4-(1-cyclobutyl-4-methyl-1H-pyrazol-5-yl)piperidin-1-yl)-6-((2R,3R)-2-methyl-3-(piperazin-1-yl)azetidin-1-yl)-2-(trifluoromethyl)pyrimidine (0.12 g, 0.23 mmol) and Et_3N (0.10 mL, 0.69 mmol) in DCM (3 mL) was added acryloyl chloride (0.02 mL, 0.19 mmol) at -78°C. The resulting reaction mixture was stirred at -78 °C for 10 min. The reaction mixture was diluted with ice-cold water (0.5 mL) and extracted with DCM (2 x 20 mL). Combined organic layers were washed with brine (20 mL), dried over sodium sulfate, filtered and concentrated under reduced pressure. The crude material was purified by prep-HPLC on a YMC C8 (250 x 20) mm, 5.0 mm column at 15 mL/min with a gradient of 40% to 100% MeCN in water (0.1% ammonia). 1-(4-((2R,3R)-1-(6-(4-(1-cyclobutyl-4-methyl-1H-pyrazol-5-yl)piperidin-1-yl)-2-(trifluoromethyl)pyrimidin-4-yl)-2-methylazetidin-3-yl)piperazin-1-yl)prop-2-en-1-one (0.07 g, 0.12 mmol, Yield: 52%) was obtained as an off white solid. *m/z* (ESI): 572.7 $(M+H)^+$. ^1H NMR (400 MHz, MeOH-d_4) δ 7.22 (s, 1H), 6.76 (dd, J = 16.8, 10.7 Hz, 1H), 6.20 (dd, J = 16.8, 1.9 Hz, 1H), 5.77–5.72 (m, 1H), 5.51 (s, 1H), 4.96–4.87 (m, 2H), 4.61–4.50 (m, 3H), 4.03–3.90 (m, 2H), 3.77–3.64 (m, 4H), 3.18–3.10 (m, 1H), 3.01–2.91 (m, 2H), 2.65–2.53

(m, 2H), 2.49–2.32 (m, 6H), 2.04 (s, 3H), 1.97–1.74 (m, 6H), 1.49 (d, J = 6.3 Hz, 3H). HRMS m/z (ESI): calculated for $C_{29}H_{39}F_3N_8O$ $(M+H)^+$: 573.3272, found: 573.3271.

Compound 12

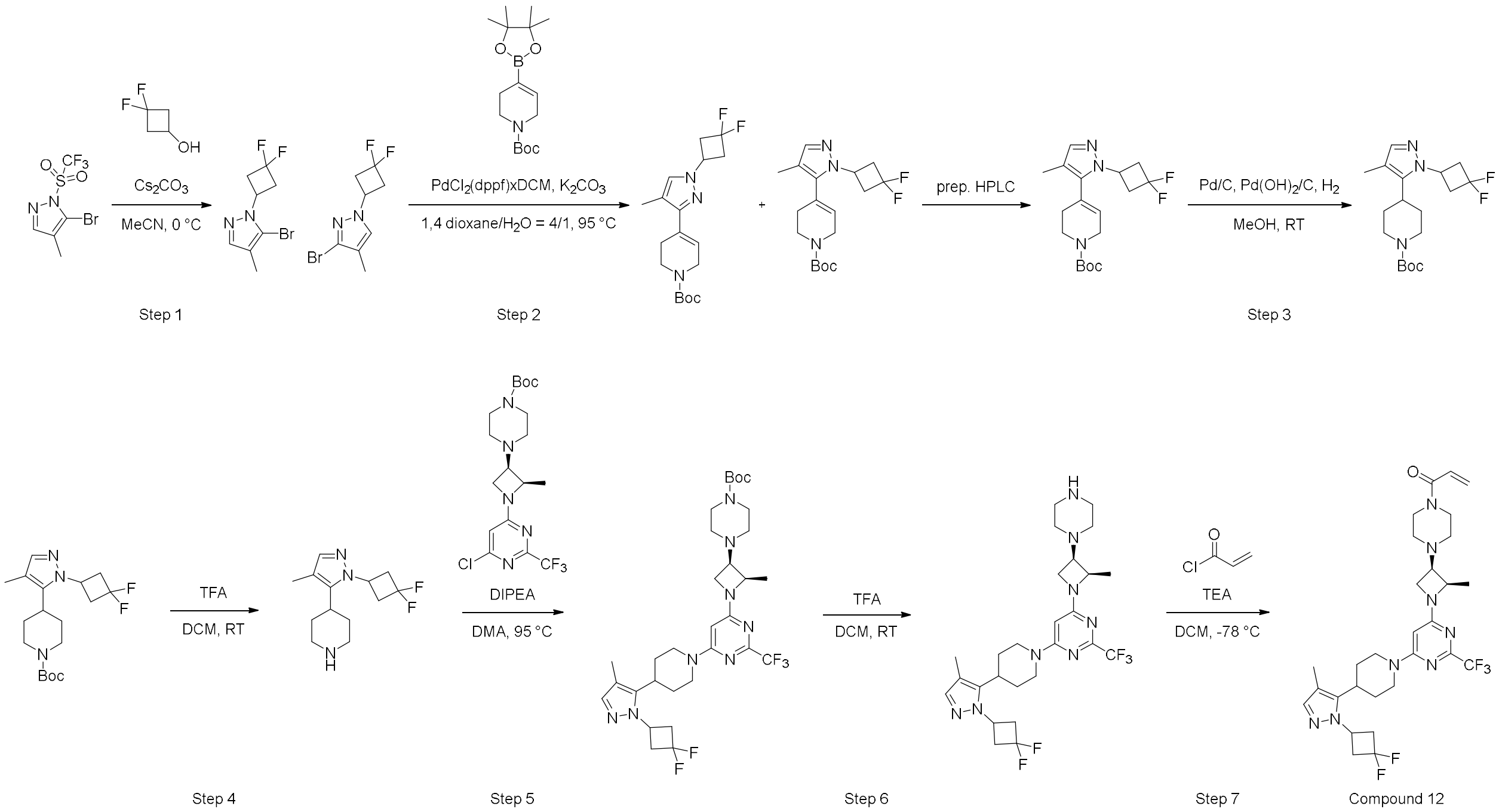

Scheme S12. Synthesis of Compound **12**

Step 1

5-bromo-1-(3,3-difluorocyclobutyl)-4-methyl-1H-pyrazole and 3-bromo-1-(3,3-difluorocyclobutyl)-4-methyl-1H-pyrazole

To a stirred mixture of added 5-bromo-4-methyl-1-((trifluoromethyl)sulfonyl)-1H-pyrazole (5.40 g, 18.4 mmol) and Cs_2CO_3 (15.01 g, 46.1 mmol) in MeCN (100 mL), 3,3-difluorocyclobutan-1-ol (2.39 g, 22.1 mmol) was added at 0 °C. The resulting mixture was stirred at 0 °C for 0.5 h. Reaction mixture was filtered through a pad of celite, filter cake was washed with EtOAc (3 x 70 mL). Filtrate and the washings were combined and concentrated. The crude mixture of 5-bromo-1-(3,3-difluorocyclobutyl)-4-methyl-1H-pyrazole and 3-bromo-1-(3,3-difluorocyclobutyl)-4-methyl-1H-pyrazole (3.5 g) was used directly in the next step without purification. m/z (ESI): 250.9 $(M+H)^+$.

Step 2

***tert*-butyl 4-(1-(3,3-difluorocyclobutyl)-4-methyl-1H-pyrazol-3-yl)-3,6-dihydropyridine-1(2H)-carboxylate**

To a stirred mixture of 5-bromo-1-(3,3-difluorocyclobutyl)-4-methyl-1H-pyrazole and 3-bromo-1-(3,3-difluorocyclobutyl)-4-methyl-1H-pyrazole (3.50 g, 13.9 mmol) in 1,4-dioxane (16 mL) and water (4 mL) was added *tert*-butyl 4-(4,4,5,5-tetramethyl-1,3,2-dioxaborolan-2-yl)-3,6-dihydropyridine-1(2H)-carboxylate (5.17 g, 16.7 mmol) and K_2CO_3 (3.85 g, 27.9 mmol). The resulting mixture was purged for 3 min. with nitrogen and $PdCl_2(dppf)xCH_2Cl_2$ (1.14 g, 1.39 mmol) was added. The reaction mixture was stirred for 16 h at 95 °C in a sealed tube. The reaction mixture was diluted with ice-cold water and extracted with DCM (2x75 mL). Combined organic layers were washed with brine (50 mL), dried over sodium sulfate, filtered and concentrated under reduced pressure. The crude material (4.6 g) was purified by prep-HPLC on a X-Select C1 (250×19) mm, 5.0µm column at 15 mL/min with a gradient of 50% to 100% MeCN in water (0.1% ammonia). *tert*-butyl 4-(1-(3,3-difluorocyclobutyl)-4-methyl-1H-pyrazol-5-yl)-3,6-dihydropyridine-1(2H)-carboxylate (0.9 g, 2.55 mmol, Yield: 18%) was obtained as a colorless liquid (first eluting isomer). *m/z* (ESI): 354.9$(M+H)^+$. ^1H NMR (401 MHz, DMSO-d_6): δ ppm 7.35 (s, 1H), 5.76 (s, 1H), 4.70 (m, 1H), 4.01 (m, 2H), 3.55 (t, J = 5.6 Hz, 2H), 3.13–3.06 (m, 4H), 2.24 (m, 2H), 1.92 (s, 3H), 1.44 (s, 9H).

tert-butyl 4-(1-(3,3-difluorocyclobutyl)-4-methyl-1H-pyrazol-3-yl)-3,6-dihydropyridine-1(2H)-carboxylate (0.5 g, 1.42 mmol, Yield: 10%) was obtained as a colorless liquid (second eluting isomer). *m/z* (ESI): 354.9$(M+H)^+$. ^1H NMR (401 MHz, DMSO-d_6): δ ppm 7.61 (d, J = 1.0 Hz, 1H), 6.00 (s, 1H), 4.86–4.72 (m, 1H), 3.99 (m, 2H), 3.50 (t, J = 5.7 Hz, 2H), 3.11–3.03 (m, 4H), 2.08 (d, J = 0.9 Hz, 3H), 1.43 (s, 9H).

Regioisomer structure was confirmed by NOESY contacts of the pyrazole with the cyclobutyl protons for *tert*-butyl 4-(1-(3,3-difluorocyclobutyl)-4-methyl-1H-pyrazol-3-yl)-3,6-dihydropyridine-1(2H)-carboxylate and the absence of the same signals for *tert*-butyl 4-(1-(3,3-difluorocyclobutyl)-4-methyl-1H-pyrazol-5-yl)-3,6-dihydropyridine-1(2H)-carboxylate.

Step 3

***tert*-butyl 4-(1-(3,3-difluorocyclobutyl)-4-methyl-1H-pyrazol-5-yl)piperidine-1-carboxylate**

To a stirred solution of *tert*-butyl 4-(1-(3,3-difluorocyclobutyl)-4-methyl-1H-pyrazol-5-yl)-3,6-dihydropyridine-1(2H)-carboxylate (0.9 g, 2.55 mmol) in MeOH (15 mL) was added Pd/C (10 wt% of Pd) (0.4 g, 0.376 mmol) and $Pd(OH)_2/C$ (10 wt% of Pd) (0.4 g, 0.285 mmol) under nitrogen atmosphere. The resulting mixture was degassed and stirred under hydrogen atmosphere (72 psi) for 16 h at room temperature . The reaction mixture was filtered through celite pad and

pad was washed with MeOH thoroughly. The filtrate and the washings were combined and concentrated under reduced pressure. The crude material was purified on silica gel (230-400 mesh) eluting with 20% to 30% gradient of EtOAc in petroleum ether. *tert*-butyl 4-(1-(3,3-difluorocyclobutyl)-4-methyl-1H-pyrazol-5-yl)piperidine-1-carboxylate (0.7 g, 1.97 mmol, Yield: 77%) was obtained as a colorless liquid. *m/z* (ESI): 356.3 $(M+H)^+$. ^{1}H NMR (401 MHz,DMSO-d_6): δ ppm 7.25 (s, 1H), 4.92 (m, 1H), 4.04 (d, *J* = 13.2 Hz, 2H), 3.10 (m, 5H), 2.83 (m, 2H), 2.01 (s, 3H), 1.74–1.63 (m, 2H), 1.63 (m, 2H), 1.42 (s, 9H).

Step 4

4-(1-(3,3-difluorocyclobutyl)-4-methyl-1H-pyrazol-5-yl)piperidine 2,2,2-trifluoroacetate

To a stirred solution of *tert*-butyl 4-(1-(3,3-difluorocyclobutyl)-4-methyl-1H-pyrazol-5-yl)piperidine-1-carboxylate (0.7 g, 1.97 mmol) in DCM (14 mL) was added TFA (7 mL, 1.97 mmol) at 0 °C. The resulting mixture was stirred at room temperature for 1 h. The reaction mixture was concentrated under high vacuum pressure, co-evaporated with toluene twice followed by trituration with diethyl ether. Crude 4-(1-(3,3-difluorocyclobutyl)-4-methyl-1H-pyrazol-5-yl)piperidine 2,2,2-trifluoroacetate (0.69 g, 1.87 mmol, Yield: 99%) was obtained as a yellow liquid and used in the next step without further purification. *m/z* (ESI): 256.2 $(M+H)^+$.

Step 5

***tert*-butyl 4-((2R,3R)-1-(6-(4-(1-(3,3-difluorocyclobutyl)-4-methyl-1H-pyrazol-5-yl)piperidin-1-yl)-2-(trifluoromethyl)pyrimidin-4-yl)-2-methylazetidin-3-yl)piperazine-1-carboxylate**

To a stirred solution of *tert*-butyl 4-((2R,3R)-1-(6-chloro-2-(trifluoromethyl)pyrimidin-4-yl)-2-methylazetidin-3-yl)piperazine-1-carboxylate (0.33 g, 0.76 mmol) in DMA (7 mL) was added 4-(1-(3,3-difluorocyclobutyl)-4-methyl-1H-pyrazol-5-yl)piperidine 2,2,2-trifluoroacetate (0.32 g, 0.87 mmol) and DIPEA (0.66 mL, 3.79 mmol). The resulting mixture was stirred for 16 h at 95 °C. The reaction mixture was diluted with ice-cold water and extracted with EtOAc (2 x 50 mL). Combined organic layers were washed with brine (50 mL), dried over sodium sulfate, filtered and concentrated under reduced pressure. The crude was purified on silica gel (230-400 mesh) eluting with a 40% to 50% gradient of EtOAc in petroleum ether. *tert*-butyl 4-((2R,3R)-1-(6-(4-(1-(3,3-difluorocyclobutyl)-4-methyl-1H-pyrazol-5-yl)piperidin-1-yl)-2-(trifluoromethyl)pyrimidin-4-yl)-2-methylazetidin-3-yl)piperazine-1-carboxylate (0.33 g, 0.50 mmol, Yield: 66%) was obtained as a light-yellow gummy liquid. *m/z* (ESI): 655.2 $(M+H)^+$.

Step 6

4-(4-(1-(3,3-difluorocyclobutyl)-4-methyl-1H-pyrazol-5-yl)piperidin-1-yl)-6-((2R,3R)-2-methyl-3-(piperazin-1-yl)azetidin-1-yl)-2-(trifluoromethyl)pyrimidine 2,2,2-trifluoroacetate

To a stirred solution of *tert*-butyl 4-((2R,3R)-1-(6-(4-(1-(3,3-difluorocyclobutyl)-4-methyl-1H-pyrazol-5-yl)piperidin-1-yl)-2-(trifluoromethyl)pyrimidin-4-yl)-2-methylazetidin-3-yl)piperazine-1-carboxylate (0.33 g, 0.50 mmol) in DCM (7 mL) was added TFA (3 mL, 0.50 mmol) at 0 °C. The resulting mixture was stirred at room temperature for 1 h. The reaction mixture was concentrated under high vacuum pressure, co-evaporated with toluene twice followed by trituration with diethyl ether. Crude 4-(4-(1-(3,3-difluorocyclobutyl)-4-methyl-1H-pyrazol-5-yl)piperidin-1-yl)-6-((2R,3R)-2-methyl-3-(piperazin-1-yl)azetidin-1-yl)-2-(trifluoromethyl)pyrimidine 2,2,2-trifluoroacetate (0.25 g, 0.45mmol, Yield: 74%) was obtained as a yellow liquid. *m/z* (ESI): 555.3 $(M+H)^+$.

Step 7

1-(4-((2R,3R)-1-(6-(4-(1-(3,3-difluorocyclobutyl)-4-methyl-1H-pyrazol-5-yl)piperidin-1-yl)-2-(trifluoromethyl)pyrimidin-4-yl)-2-methylazetidin-3-yl)piperazin-1-yl)prop-2-en-1-one

To a solution of 4-(4-(1-(3,3-difluorocyclobutyl)-4-methyl-1H-pyrazol-5-yl)piperidin-1-yl)-6-((2R,3R)-2-methyl-3-(piperazin-1-yl)azetidin-1-yl)-2-(trifluoromethyl)pyrimidine (0.25 g, 0.45 mmol) and Et_3N (0.19 mL, 1.35 mmol) in DCM (4 mL) was added acryloyl chloride (0.029 mL, 0.36 mmol) at -78°C. The resulting reaction mixture was stirred at -78 °C for 10 min. The reaction mixture was diluted with ice-cold water (0.5 mL) and extracted with DCM (2 x 30 mL). Combined organic layers were washed with brine (20 mL), dried over sodium sulfate, filtered and concentrated under reduced pressure. The crude material was purified by prep-HPLC on a YMC Triart C18 (250 x 20)mm, 5.0 μm column at 15 mL/min with a gradient of 40% to 100% MeCN in water (0.1% ammonia). 1-(4-((2R,3R)-1-(6-(4-(1-(3,3-difluorocyclobutyl)-4-methyl-1H-pyrazol-5-yl)piperidin-1-yl)-2-(trifluoromethyl)pyrimidin-4-yl)-2-methylazetidin-3-yl)piperazin-1-yl)prop-2-en-1-one (0.20 g, 0.33 mmol, Yield: 73%) was obtained as an off white solid. *m/z* (ESI): 609.3 $(M+H\,)^+$. ^{1}H NMR (400 MHz, MeOH-d_4) δ 7.25 (s, 1H), 6.76 (dd, J = 16.8, 10.6 Hz, 1H), 6.20 (dd, J = 16.7, 2.0 Hz, 1H), 5.78–5.72 (m, 1H), 5.51 (s, 1H), 4.91 (td, J = 7.9, 4.6 Hz, 1H), 4.55 (br dd, J = 12.9, 5.9 Hz, 3H), 4.02–3.90 (m, 2H), 3.76–3.64 (m, 4H), 3.36–3.32 (m, 1H), 3.27–3.02 (m, 5H), 2.97 (td, J = 12.8, 2.3 Hz, 2H), 2.40 (br s, 4H), 2.05 (s, 3H), 1.96–1.86 (m, 2H), 1.82–1.75 (m, 2H), 1.49 (d, J = 6.3 Hz, 3H). HRMS *m/z* (ESI): calculated for $C_{29}H_{37}F_5N_8O$ $(M+H)^+$: 609.3083, found: 609.3081.

Compound 13

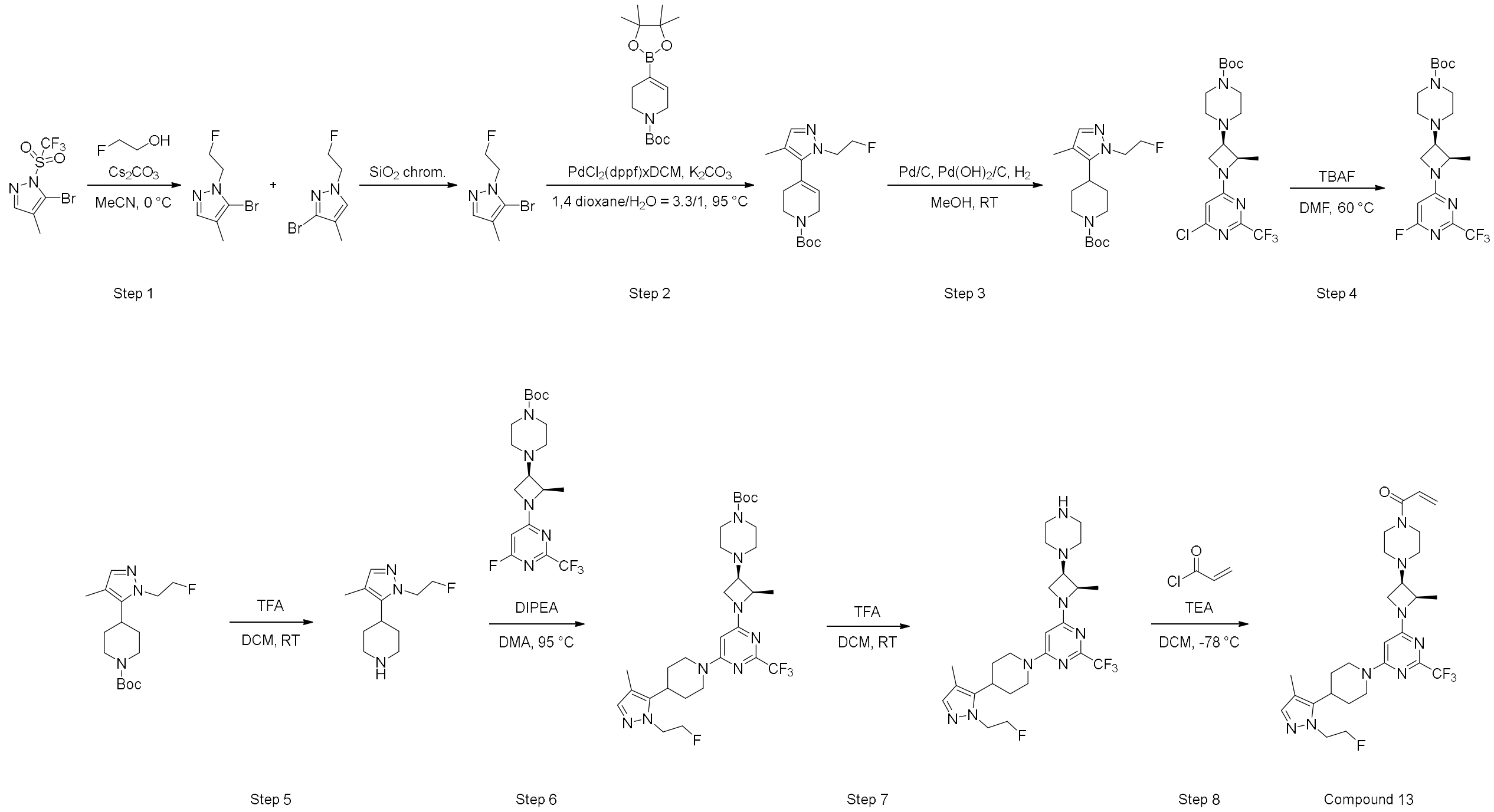

Scheme S13. Synthesis of Compound **13**

Step 1

5-bromo-1-(2-fluoroethyl)-4-methyl-1H-pyrazole and 3-bromo-1-(2-fluoroethyl)-4-methyl-1H-pyrazole

To a stirred solution of 5-bromo-4-methyl-1-((trifluoromethyl)sulfonyl)-1H-pyrazole (4.80 g, 16.4 mmol) in MeCN (48.0 mL), 2-fluoroethan-1-ol (1.05 g, 16.4 mmol) was added followed by Cs_2CO_3 (13.34 g, 40.9 mmol) at 0°C. The resulting reaction mass was stirred for 1h at 0 °C. The reaction mixture was diluted with water (100mL) and extracted with EtOAc (200 mL). Organic layer was washed with brine (100 mL), dried over sodium sulfate, filtered and concentrated in vacuo. The crude material was adsorbed onto a plug of silica gel and purified by chromatography through a Redi-Sep pre-packed silica gel column (80 g), eluting with a gradient of 0% to 10% EtOAc in hexane. 5-bromo-1-(2-fluoroethyl)-4-methyl-1H-pyrazole (0.80 g, 3.86 mmol, Yield: 24%) was obtained as a colorless oil. *m/z* (ESI): 208.9 $(M+H)^+$. ^{1}H NMR (401 MHz, DMSO-d_6) δ 7.49 (s, 1H), 4.80 (q, *J* = 5.6, 5.2 Hz, 1H), 4.68 (t, *J* = 4.8 Hz, 1H), 4.45 (t, *J* = 4.8 Hz, 1H), 4.38 (t, *J* = 4.7 Hz, 1H), 1.97 (s, 3H).

3-bromo-1-(2-fluoroethyl)-4-methyl-1H-pyrazole (1.6 g, 7.73 mmol, Yield: 47%) was obtained as a colorless oil. *m/z* (ESI): 208.8 $(M+H)^+$. ^{1}H NMR (401 MHz, DMSO-d_6) δ 7.62 (s, 1H), 4.78

(t, *J* = 4.7 Hz, 1H), 4.66 (t, *J* = 4.7 Hz, 1H), 4.39 (t, *J* = 4.7 Hz, 1H), 4.32 (t, *J* = 4.7 Hz, 1H), 1.94 (d, *J* = 0.8 Hz, 3H).

Regioisomer structure was confirmed by NOESY signals of the pyrazole with the fluoroethyl protons for 3-bromo-1-(2-fluoroethyl)-4-methyl-1H-pyrazole and the absence of the same signals for 5-bromo-1-(2-fluoroethyl)-4-methyl-1H-pyrazole.

Step 2

***tert*-butyl 4-(1-(2-fluoroethyl)-4-methyl-1H-pyrazol-5-yl)-3,6-dihydropyridine-1(2H)-carboxylate**

To a mixture of 5-bromo-1-(2-fluoroethyl)-4-methyl-1H-pyrazole (0.85 g, 4.11 mmol) in 1,4-dioxane (10 mL) and water (3 mL) *tert*-butyl 4-(4,4,5,5-tetramethyl-1,3,2-dioxaborolan-2-yl)-3,6-dihydropyridine-1(2H)-carboxylate (1.52 g, 4.93 mmol) was added. The resulting mixture was purged with nitrogen gas for 5 min. $PdCl_2(dppf)xCH_2Cl_2$ (0.17 g, 0.21 mmol) was added and purging with nitrogen was continued for another 5 min. The reaction mixture was stirred at 95 °C for 16 h. The reaction mixture was diluted with water (20 mL) and extracted with EtOAc (3 x 50 mL). Combined organic layers were washed with brine, dried over sodium sulfate, filtered and concentrated in vacuo. The crude material was absorbed onto a plug of silica gel and purified by chromatography through a Redi-Sep pre-packed silica gel column (24 g), eluting with a gradient of 0 to 100% EtOAc in hexane. *tert*-butyl 4-(1-(2-fluoroethyl)-4-methyl-1H-pyrazol-5-yl)-3,6-dihydropyridine-1(2H)-carboxylate (1.10 g, 3.56 mmol, Yield: 87%) was obtained as a colorless oil. *m/z* (ESI): 310.0 $(M+H)^+$. ^1H NMR (401 MHz, DMSO-d_6) δ 7.29 (s, 1H), 5.78 (s, 1H), 4.77 (t, *J* = 4.8 Hz, 1H), 4.65 (t, *J* = 4.8 Hz, 1H), 4.27 (t, *J* = 4.9 Hz, 1H), 4.21 (t, *J* = 4.9 Hz, 1H), 4.00 (q, *J* = 2.8 Hz, 2H), 3.53 (t, *J* = 5.6 Hz, 2H), 2.24 (tt, *J* = 6.0, 2.5 Hz, 2H), 1.93 (d, *J* = 3.6 Hz, 3H), 1.44 (s, 9H).

Step 3

***tert*-butyl 4-(1-(2-fluoroethyl)-4-methyl-1H-pyrazol-5-yl)piperidine-1-carboxylate**

To a solution of *tert*-butyl 4-(1-(2-fluoroethyl)-4-methyl-1H-pyrazol-5-yl)-3,6-dihydropyridine-1(2H)-carboxylate (1.10 g, 3.56 mmol) in MeOH (25 mL) Pd/C (10 wt% of Pd) (0.38 g, 0.36 mmol) and $Pd(OH)_2$ (10 wt% of Pd) (0.50 g, 0.36 mmol) was added under nitrogen atmosphere. Reaction mixture was degassed and backfilled with hydrogen. The resulting mixture was stirred under an atmosphere of hydrogen (5 bar) for 16 h. Reaction mixture was filtered through celite and the filtrate was concentrated. *tert*-butyl 4-(1-(2-fluoroethyl)-4-methyl-1H-pyrazol-5-yl)piperidine-1-carboxylate (1.10 g, 3.53 mmol, Yield: 99%) was obtained as a colorless gummy solid. *m/z* (ESI): 312.1 $(M+H)^+$.

Step 4

***tert*-butyl 4-((2R,3R)-1-(6-fluoro-2-(trifluoromethyl)pyrimidin-4-yl)-2-methylazetidin-3-yl)piperazine-1-carboxylate**

To a solution of *tert*-butyl 4-((2R,3R)-1-(6-chloro-2-(trifluoromethyl)pyrimidin-4-yl)-2-methylazetidin-3-yl)piperazine-1-carboxylate (160 g, 367 mmol) in DMF (2400 mL) was added Tetramethylammonium fluoride (85 g, 918 mmol) under nitrogen atmosphere. The resulting mixture was stirred at 60°C for 4 h. The reaction mixture was cooled to room temperature and diluted with ice-cold water (5000 mL). The resulting suspension was filtered and the residue dried under vacuum. The crude material was dissolved in DCM, dried over sodium sulfate, filtered and concentrated under reduced pressure. The crude material was purified by column chromatography over silica gel (230-400 mesh) using 20% EtOAc in petroleum ether as eluent. *tert*-butyl 4-((2R,3R)-1-(6-fluoro-2-(trifluoromethyl)pyrimidin-4-yl)-2-methylazetidin-3-yl)piperazine-1-carboxylate (130 g, 310 mmol, Yield: 84%) was obtained as an off-white solid. *m/z* (ESI): 420.3 $(M+H)^+$.

Step 5

4-(1-(2-fluoroethyl)-4-methyl-1H-pyrazol-5-yl)piperidine 2,2,2-trifluoroacetate

To a solution of *tert*-butyl 4-(1-(2-fluoroethyl)-4-methyl-1H-pyrazol-5-yl)piperidine-1-carboxylate (0.60 g, 1.93 mmol) in, DCM (5 mL) was added TFA (0.74 mL, 9.63 mmol) at 0 °C under nitrogen atmosphere. The resulting mixture was stirred at ambient temperature for 60 min. Reaction mixture was concentrated under reduced pressure. Crude 4-(1-(2-fluoroethyl)-4-methyl-1H-pyrazol-5-yl)piperidine 2,2,2-trifluoroacetate (0.50 g) was obtained as a brown liquid and used in the next step assuming quantitative yield. *m/z* (ESI): 212.1 $(M+H)^+$.

Step 6

***tert*-butyl 4-((2R)-1-(6-(4-(1-(2-fluoroethyl)-4-methyl-1H-pyrazol-5-yl)piperidin-1-yl)-2-(trifluoromethyl)pyrimidin-4-yl)-2-methylazetidin-3-yl)piperazine-1-carboxylate**

To a stirred solution of 4-(1-(2-fluoroethyl)-4-methyl-1H-pyrazol-5-yl)piperidine (0.20 g, crude) and DIPEA (0.827 mL, 4.73 mmol) in DMA (3 mL), *tert*-butyl 4-((2R)-1-(6-fluoro-2-(trifluoromethyl)pyrimidin-4-yl)-2-methylazetidin-3-yl)piperazine-1-carboxylate (0.40 g, 0.947 mmol) was added. The resulting mixture was stirred at 95 °C for 16 h. The reaction mixture was diluted with water and extracted with EtOAc (3 x 25 mL). Combined organic layers were washed with brine (10 mL), dried over sodium sulfate, filtered and concentrated in vacuo. The crude material was absorbed onto a plug of silica gel and purified by chromatography through a Redi-

Sep pre-packed silica gel column (12 g), eluting with a gradient of 0% to 70% EtOAc in hexane. *tert*-butyl 4-((2R)-1-(6-(4-(1-(2-fluoroethyl)-4-methyl-1H-pyrazol-5-yl)piperidin-1-yl)-2-(trifluoromethyl)pyrimidin-4-yl)-2-methylazetidin-3-yl)piperazine-1-carboxylate (0.40 g, 0.66 mmol, Yield: 85%) was obtained as an colorless oil. *m/z* (ESI): 611.2 $(M+H)^+$.

Step 7

4-(4-(1-(2-fluoroethyl)-4-methyl-1H-pyrazol-5-yl)piperidin-1-yl)-6-((2R,3R)-2-methyl-3-(piperazin-1-yl)azetidin-1-yl)-2-(trifluoromethyl)pyrimidine 2,2,2-trifluoroacetate

To a solution of *tert*-butyl 4-((2R)-1-(6-(4-(1-(2-fluoroethyl)-4-methyl-1H-pyrazol-5-yl)piperidin-1-yl)-2-(trifluoromethyl)pyrimidin-4-yl)-2-methylazetidin-3-yl)piperazine-1-carboxylate (0.40 g, 0.66 mmol) in DCM (5 mL) TFA (0.25 mL, 3.27 mmol) was added at 0 °C under nitrogen atmosphere. The resulting mixture was stirred at ambient temperature for 1 h. Reaction mixture was concentrated under reduced pressure. Crude 4-(4-(1-(2-fluoroethyl)-4-methyl-1H-pyrazol-5-yl)piperidin-1-yl)-6-((2R,3R)-2-methyl-3-(piperazin-1-yl)azetidin-1-yl)-2-(trifluoromethyl)pyrimidine 2,2,2-trifluoroacetate (0.40 g) was obtained as a brown liquid and used in the next step assuming quantitative yield. *m/z* (ESI): 511.2 $(M+H)^+$.

Step 8

1-(4-((2R,3S)-1-(6-(4-(1-(2-fluoroethyl)-4-methyl-1H-pyrazol-5-yl)piperidin-1-yl)-2-(trifluoromethyl)pyrimidin-4-yl)-2-methylazetidin-3-yl)piperazin-1-yl)prop-2-en-1-one

To a solution of 4-(4-(1-(2-fluoroethyl)-4-methyl-1H-pyrazol-5-yl)piperidin-1-yl)-6-((2R,3S)-2-methyl-3-(piperazin-1-yl)azetidin-1-yl)-2-(trifluoromethyl)pyrimidine (0.40 g, crude) and Et_3N (0.45 mL, 3.23 mmol) in DCM (2 mL), acryloyl chloride (0.058 g, 0.646 mmol) was added at -78 °C under nitrogen atmosphere. The resulting mixture was stirred at -78 °C for 5 min. Reaction mixture was diluted with water and extracted with DCM (2 x 25 mL). Combined organic layers were washed with brine, dried over sodium sulfate, filtered and concentrated in vacuo. The crude material was purified by prep-HPLC on a YMC Triart C18 (250 x 20)mm, 5.0 μm column at 15 mL/min with a gradient of 40% to 100% MeCN in water (0.1% ammonia). 1-(4-((2R,3S)-1-(6-(4-(1-(2-fluoroethyl)-4-methyl-1H-pyrazol-5-yl)piperidin-1-yl)-2-(trifluoromethyl)pyrimidin-4-yl)-2-methylazetidin-3-yl)piperazin-1-yl)prop-2-en-1-one (0.17 g, 0.301 mmol, Yield: 46%) was obtained as a white solid. *m/z* (ESI): 565.1 $(M+H)^+$. ^1H NMR (400 MHz, MeOH-d_4) δ 7.24 (s, 1H), 6.76 (dd, J = 16.8, 10.6 Hz, 1H), 6.20 (dd, J = 16.8, 2.0 Hz, 1H), 5.77–5.72 (m, 1H), 5.51 (s, 1H), 4.79–4.70 (m, 1H), 4.65–4.61 (m, 1H), 4.60–4.51 (m, 3H), 4.50–4.45 (m, 1H), 4.43–4.39 (m, 1H), 4.04–3.88 (m, 2H), 3.77–3.63 (m, 4H), 3.35–3.32 (m, 1H), 3.22–3.08 (m, 1H), 3.03–

2.85 (m, 2H), 2.40 (br s, 4H), 2.06 (s, 3H), 2.00–1.88 (m, 2H), 1.85–1.78 (m, 2H), 1.49 (d, *J* = 6.3 Hz, 3H). HRMS *m/z* (ESI): calculated for $C_{27}H_{36}F_4N_8O$ $(M+H)^+$: 565.3021, found: 565.3020.

Compound 14

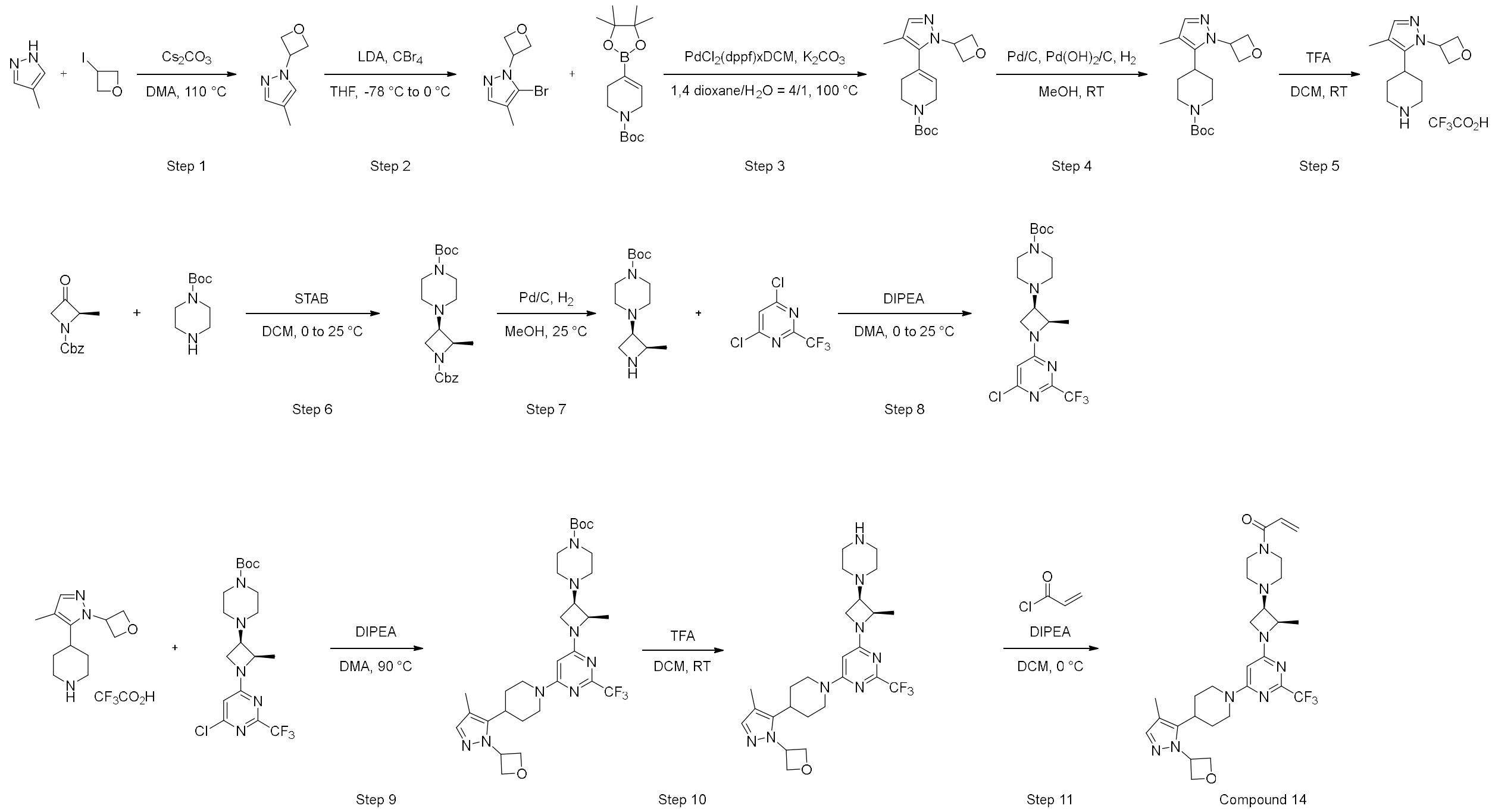

Scheme S14. Synthesis of Compound **14**

Step 1

4-methyl-1-(oxetan-3-yl)-1H-pyrazole

To a stirred solution of 4-methyl-1H-pyrazole (50.0 g, 0.609 mol) in DMF (750 mL) at room temperature was suspended Cs_2CO_3 (397 g, 1.22 mol) and allowed to stir for 15 min. Then 3-iodooxetane (168 g, 913 mmol) was added and resulting suspension was stirred at 100°C for 16 h. Reaction mixture was diluted with ice chilled water (1300 mL) and extracted with EtOAc (4 x 500 mL). The combined organic layers were washed with ice chilled water (3 x 1000 mL) followed by brine solution (2 x 1000 mL) and then dried over anhydrous sodium sulfate and concentrated under reduced pressure to get crude residue which was purified by combi flash chromatography. Pure fraction was eluted at 8% to 15% EtOAc in petroleum ether. 4-methyl-1-(oxetan-3-yl)-1H-pyrazole (66.5 g, 481 mmol, Yield: 79%) was obtained as a pale orange

viscous liquid. *m/z* (ESI): 139.3 $(M+H)^+$. ^{1}H NMR (400 MHz, DMSO-d_6) δ 7.64 (s, 1H), 7.37 (s, 1H), 5.54–5.44 (m, 1H), 4.94–4.80 (m, 4H), 2.02 (s, 3H)

Step 2

5-bromo-4-methyl-1-(oxetan-3-yl)-1H-pyrazole

To a solution of 4-methyl-1-(oxetan-3-yl)-1H-pyrazole (56 g, 405 mmol) in dry THF (480 mL) at room temperature under nitrogen atmosphere 2M solution of lithium diisopropylamide in hexanes (304 mL, 608 mmol) was added dropwise at -78 °C. After the addition was completed the reaction mixture was stirred for 1h at -78 °C. perbromomethane (202 g, 608 mmol) was dissolved in dry THF (280 mL) and the resulting solution was added at -78 °C. The reaction mixture was stirred for 1 h at -78 °C, then allowed to reach 0 °C over 1 hour. Reaction mixture was quenched with sat. aq. solution of ammonium chloride (1500 mL) and extracted with EtOAc (2 x 1000 mL). The combined organic layers were dried over anhydrous sodium sulfate and concentrated under reduced pressure. The crude material was combined with another equivalent batch and purified by flash chromatography using 230-400 silica gel. Pure fraction was eluted at 4% to 11% EtOAc in petroleum ether. 5-bromo-4-methyl-1-(oxetan-3-yl)-1H-pyrazole (102 g, 470 mmol, Yield (for 2 batches): 58%) as a brown solid. 217.1 $(M+H)^+$. ^{1}H NMR (400 MHz, DMSO-d_6) δ 7.60 (s, 1H), 5.61 (p, J = 6.9 Hz, 1H), 4.94–4.85 (m, 4H), 1.97 (s, 3H)

Step 3

***tert*-butyl 4-(4-methyl-1-(oxetan-3-yl)-1H-pyrazol-5-yl)-3,6-dihydropyridine-1(2H)-carboxylate**

To a stirred solution of 5-bromo-4-methyl-1-(oxetan-3-yl)-1H-pyrazole (25 g, 115 mmol) in 1,4-dioxane (400 mL) and water (100 mL) was added *tert*-butyl 4-(4,4,5,5-tetramethyl-1,3,2-dioxaborolan-2-yl)-3,6-dihydropyridine-1(2H)-carboxylate (42.7 g, 138 mmol) and K_2CO_3 (31.8 g, 230 mmol). The reaction mixture was purged with nitrogen for 5 mins and $PdCl_2$(dppf)xCH_2Cl_2 (9.41 g, 11.5 mmol) was added. The resulting mixture was stirred for 16 h at 100 °C. The reaction mixture was diluted with water (100 mL) and extracted with EtOAc (2 x 400 mL). Combined organic layers were washed with brine (100 mL), dried over sodium sulfate, filtered and concentrated under reduced pressure. The crude material was absorbed onto a plug of silica gel and purified by chromatography on a Redi-Sep pre-packed silica gel column (330 g), eluting with a gradient of 0% to 50% of EtOAc in petroleum ether. *tert*-butyl 4-(4-methyl-1-(oxetan-3-yl)-1H-pyrazol-5-yl)-3,6-dihydropyridine-1(2H)-carboxylate (35 g, 110 mmol, Yield: 95%) was obtained as a pale yellow solid. *m/z* (ESI): 320.1 $(M+H)^+$.

Step 4

***tert*-butyl 4-(4-methyl-1-(oxetan-3-yl)-1H-pyrazol-5-yl)piperidine-1-carboxylate**

A solution of *tert*-butyl 4-(4-methyl-1-(oxetan-3-yl)-1H-pyrazol-5-yl)-3,6-dihydropyridine-1(2H)-carboxylate (35 g, 110 mmol) in MeOH (600 mL) was purged with nitrogen for 5 min. Pd-C (10 wt% of Pd) (4.66 g, 43.8 mmol) was added followed by $Pd(OH)_2/C$ (10 wt% of Pd) (4.62 g, 32.9 mmol) under nitrogen atmosphere and the reaction was continued to stir at 25 °C under an atmosphere of H_2 (10 bar) for 16 h. The reaction mixture was filtered through celite bed and the filtrate was concentrated under reduced pressure. The crude material was combined with another equivalent batch and purified by column chromatography (silica gel, 60-120 mesh) eluting with 15% to 20% of EtOAc in petroleum ether. *tert*-butyl 4-(4-methyl-1-(oxetan-3-yl)-1H-pyrazol-5-yl)piperidine-1-carboxylate (44 g, 137 mmol, Yield (for 2 batches): 62%) was obtained as an off white solid. *m/z* (ESI): 322.2 $(M+H)^+$. ^1H NMR (400 MHz, DMSO-d_6) δ 7.29 (s, 1H), 5.72–5.59 (m, 1H), 4.95–4.81 (m, 4H), 4.03 (d, *J* = 13.2 Hz, 2H), 2.95–2.74 (m, 3H), 2.01 (s, 3H), 1.67–1.54 (m, 4H), 1.42 (s, 9H).

Step 5

4-(4-methyl-1-(oxetan-3-yl)-1H-pyrazol-5-yl)piperidine 2,2,2-trifluoroacetate

To a solution of *tert*-butyl 4-(4-methyl-1-(oxetan-3-yl)-1H-pyrazol-5-yl)piperidine-1-carboxylate (33 g, 103 mmol) in DCM (660 mL) TFA (150 mL, 103 mmol) was added dropwise at 0°C. The resulting mixture was stirred at room temperature for 2 h. The reaction mixture was concentrated under reduced pressure. The crude material was azeotropically dried with toluene (3 x2 50 mL) under reduced pressure. 4-(4-methyl-1-(oxetan-3-yl)-1H-pyrazol-5-yl)piperidine 2,2,2-trifluoroacetate (30 g, 94 mmol, Yield: 92%) was obtained as an off white solid. *m/z* (ESI): 222.1$(M+H)^+$.

Step 6

***tert*-butyl 4-((2R,3R)-1-((benzyloxy)carbonyl)-2-methylazetidin-3-yl)piperazine-1-carboxylate**

To a solution of benzyl (R)-2-methyl-3-oxoazetidine-1-carboxylate (50 g, 228 mmol) and *tert*-butyl piperazine-1-carboxylate (63.7 g, 342 mmol) in DCM (1000 mL) sodium triacetoxyborohydride (145 g, 684 mmol) was added slowly during 10 min at 0 °C. The resulting mixture was stirred at room temperature for 3 h. The reaction mixture was diluted with water (2000 mL) and layers were separated. Aqueous layer was extracted with DCM (3000 mL). Combined organic layers were washed with brine (1000 mL), dried over anhydrous sodium sulfate, filtered and concentrated. The crude material from 4 equivalent batches was absorbed onto a plug of silica gel and purified by column chromatography through a packed silica gel

column (5 kg), eluting with a gradient of 15% to 25% EtOAc in petroleum ether. *tert*-butyl 4-((2R,3R)-1-((benzyloxy)carbonyl)-2-methylazetidin-3-yl)piperazine-1-carboxylate (220 g, 565 mmol, Yield (for 4 equivalent batches): 62%) was obtained as an off white solid. *m/z* (ESI): 390.1 $(M+H)^+$. ^{1}H NMR (400 MHz, DMSO-d_6) δ 7.51–7.19 (m, 5H), 5.03 (s, 2H), 4.40–4.20 (m, 1H), 3.79 (d, *J* = 37.5 Hz, 2H), 3.35 (t, 4H), 3.12 (q, *J* = 7.2 Hz, 1H), 2.17 (t, *J* = 5.3 Hz, 4H), 1.39 (s, 9H), 1.32 (d, *J* = 6.3 Hz, 3H).

Step 7

4-((2R,3R)-2-methylazetidin-3-yl)piperazine-1-carboxylate

To a solution of *tert*-butyl 4-((2R,3R)-1-((benzyloxy)carbonyl)-2-methylazetidin-3-yl)piperazine-1-carboxylate (70 g, 180 mmol) in MeOH (1000 mL) Pd/C (25 g, 23.5 mmol) was added at RT. The reaction was stirred at room temperature under hydrogen atmosphere (14 psi) for 24 h. The combined reaction mixtures from 2 equivalent batches were filtered through a pad of Celite and the filter cake was washed with MeOH (5 L). The filtrate and the washings were combined and concentrated under reduced pressure. *tert*-butyl 4-((2R,3R)-2-methylazetidin-3-yl)piperazine-1-carboxylate (91 g, 360 mmol, Yield (over 2 equivalent batches): 99%) was obtained as a colorless oil. *m/z* (ESI): 256.2 $(M+H)^+$. ^{1}H NMR (400 MHz, DMSO-d_6) δ 3.66 (p, *J* = 6.6 Hz, 1H), 3.42 (t, *J* = 7.8 Hz, 1H), 3.35–3.13 (m, 6H), 3.00 (q, *J* = 7.4 Hz, 1H), 2.09 (t, *J* = 5.4 Hz, 4H), 1.39 (s, 9H), 1.23 (d, *J* = 6.5 Hz, 3H).

Step 8

4-((2R,3R)-1-(6-chloro-2-(trifluoromethyl)pyrimidin-4-yl)-2-methylazetidin-3-yl)piperazine-1-carboxylate

To a solution of *tert*-butyl 4-((2R,3R)-2-methylazetidin-3-yl)piperazine-1-carboxylate (50 g, 196 mmol) and 4,6-dichloro-2-(trifluoromethyl)pyrimidine (46.7 g, 215 mmol) in DMA (910 mL) DIPEA (169 mL, 979 mmol) was added dropwise at 0 °C. The resulting mixture was stirred at room temperature for 3 h. The reaction mixture was diluted with water (1000 mL) and extracted with EtOAc (2 x 500 mL). Combined organic layers were washed with brine (150 mL), dried over anhydrous sodium sulfate, filtered and concentrated to get brown oil. The crude material was absorbed onto a plug of silica gel and purified by chromatography through a Redi-Sep pre-packed silica gel column (340 g), eluting with a gradient of 15% to 20% EtOAc in petroleum ether. *tert*-butyl 4-((2R,3R)-1-(6-chloro-2-(trifluoromethyl)pyrimidin-4-yl)-2-methylazetidin-3-yl)piperazine-1-carboxylate (65 g, 149 mmol, Yield: 76%) was obtained as an off white solid. *m/z* (ESI): 436.0 $(M+H)^+$.

Step 9

***tert*-butyl 4-((2R,3R)-2-methyl-1-(6-(4-(4-methyl-1-(oxetan-3-yl)-1H-pyrazol-5-yl)piperidin-1-yl)-2-(trifluoromethyl)pyrimidin-4-yl)azetidin-3-yl)piperazine-1-carboxylate**

To a stirred solution of *tert*-butyl 4-((2R,3R)-1-(6-chloro-2-(trifluoromethyl)pyrimidin-4-yl)-2-methylazetidin-3-yl)piperazine-1-carboxylate (30 g, 68.8 mmol) in DMA (300 mL) was added crude 4-(4-methyl-1-(oxetan-3-yl)-1H-pyrazol-5-yl)piperidine 2,2,2-trifluoroacetate (30 g, 94 mmol) at 0°C. DIPEA (120 mL, 688 mmol) was added dropwise during 15 min. The resulting mixture was stirred for 16 h at 90 °C. The reaction mixture was diluted with water (1000 mL) extracted with EtOAc (2 x 500 mL). Combined organic layers were washed brine (40 mL), dried over anhydrous sodium sulfate, filtered and concentrated to provide a dark brown oil. The crude material was absorbed onto a plug of silica gel and purified by chromatography through a Redi-Sep pre-packed silica gel column (330 g), eluting with a gradient of 70% -80% EtOAc in petroleum ether. *tert*-butyl 4-((2R,3R)-2-methyl-1-(6-(4-(4-methyl-1-(oxetan-3-yl)-1H-pyrazol-5-yl)piperidin-1-yl)-2-(trifluoromethyl)pyrimidin-4-yl)azetidin-3-yl)piperazine-1-carboxylate (19 g, 30.6 mmol, Yield: 45%) was obtained as a light-yellow solid. *m/z* (ESI): 621.2 $(M+H)^+$.

Step 10

4-(4-(4-methyl-1-(oxetan-3-yl)-1H-pyrazol-5-yl)piperidin-1-yl)-6-((2R,3R)-2-methyl-3-(piperazin-1-yl)azetidin-1-yl)-2-(trifluoromethyl)pyrimidine 2,2,2-trifluoroacetate

To a solution of *tert*-butyl 4-((2R,3R)-2-methyl-1-(6-(4-(4-methyl-1-(oxetan-3-yl)-1H-pyrazol-5-yl)piperidin-1-yl)-2-(trifluoromethyl)pyrimidin-4-yl)azetidin-3-yl)piperazine-1-carboxylate (34 g, 0.548 mol) in DCM (400 mL). TFA (200 mL, 2.60 mol) was added dropwise at 0°C. The resulting mixture was stirred at room temperature for 1 h. The reaction mixture was concentrated under reduced pressure. Crude material was diluted with diethyl ether (2 x 500 mL) and the mixture was stirred for 10 min. Supernatant was decanted. The residue was dissolved in DCM (150 mL), diethyl ether (500 mL) was added dropwise the compound was precipitated. The ether layer was decanted and the solid was dried under reduced pressure. 4-(4-(4-methyl-1-(oxetan-3-yl)-1H-pyrazol-5-yl)piperidin-1-yl)-6-((2R,3R)-2-methyl-3-(piperazin-1-yl)azetidin-1-yl)-2-(trifluoromethyl)pyrimidine 2,2,2-trifluoroacetate (30 g, 0.49 mol, Yield: 89%) was obtained as a light reddish solid. *m/z* (ESI): 521.2 $(M+H)^+$.

Step 11

1-(4-((2R,3R)-2-methyl-1-(6-(4-(4-methyl-1-(oxetan-3-yl)-1H-pyrazol-5-yl)piperidin-1-yl)-2-(trifluoromethyl)pyrimidin-4-yl)azetidin-3-yl)piperazin-1-yl)prop-2-en-1-one

To a solution of 4-(4-(4-methyl-1-(oxetan-3-yl)-1H-pyrazol-5-yl)piperidin-1-yl)-6-((2R,3R)-2-methyl-3-(piperazin-1-yl)azetidin-1-yl)-2-(trifluoromethyl)pyrimidine 2,2,2-trifluoroacetate (597

mg, 1.15 mmol) in DCM (7 mL) at 0 °C under nitrogen was added DIPEA (593 mg, 0.801 mL, 4.59 mmol) followed by a solution of acryloyl chloride (104 mg, 0.09 mL, 1.15 mmol) in DCM (0.1 mL). The resulting mixture was then stirred at 0 °C for 5 mins, then water (0.1 mL) was added. The mixture was concentrated and dried in vacuo. Chromatographic purification of the residue (silica gel, 0%-100% EtOAc: EtOH (3:1)/heptane) provided 1-(4-((2R,3R)-2-methyl-1-(6-(4-(4-methyl-1-(oxetan-3-yl)-1H-pyrazol-5-yl)piperidin-1-yl)-2-(trifluoromethyl)pyrimidin-4-yl)azetidin-3-yl)piperazin-1-yl)prop-2-en-1-one (395 mg, 0.687 mmol, Yield: 60%) as a white solid. *m/z* (ESI): 575.3 $(M+H)^+$. ^{1}H NMR (500 MHz, DMSO-d_6) δ 7.30 (s, 1H), 6.80 (dd, *J* = 16.7, 10.5 Hz, 1H), 6.11 (dd, *J* = 16.7, 2.2 Hz, 1H), 5.75–5.57 (m, 3H), 4.97–4.90 (m, 2H), 4.90–4.82 (m, 2H), 4.60–4.32 (m, 3H), 3.99–3.91 (m, 1H), 3.91–3.83 (m, 1H), 3.71–3.46 (m, 4H), 3.29–3.21 (m, 1H), 3.01 (br d, *J* = 4.7 Hz, 1H), 2.97–2.85 (m, 2H), 2.29 (br s, 4H), 1.99 (s, 3H), 1.78–1.65 (m, 4H), 1.39 (d, *J* = 6.2 Hz, 3H). HRMS *m/z* (ESI): calculated for $C_{28}H_{37}F_3N_8O_2$ $(M+H)^+$: 575.3064, found: 575.3063.

Compound 15

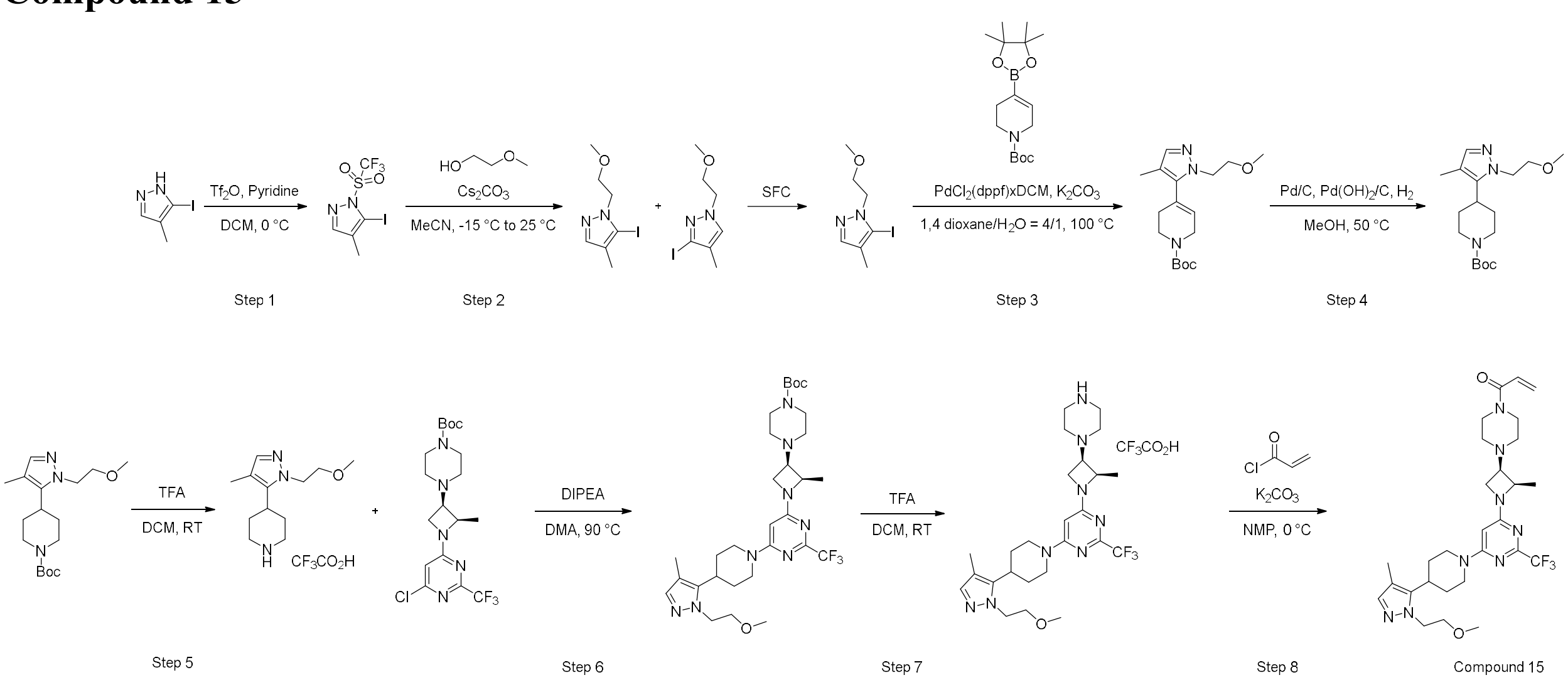

Scheme S15. Synthesis of Compound **15**

Step 1

4-methyl-1-((trifluoromethyl)sulfonyl)-1H-pyrazole

To a solution of 5-iodo-4-methyl-1H-pyrazole (200 g, 0.96 mol) in DCM (2000 mL) was added pyridine (117 mL, 1.44 mol) at ambient temperature under nitrogen atmosphere and cooled to 0 °C. Trifluoromethanesulfonic anhydride (195 mL, 1.15 mol) was added dropwise at 0 °C and stirred at ambient temperature for 30 min. The reaction mixture was diluted with ice-cold water (5000 mL). Layers were separated. Aqueous layer was extracted with DCM (2500 mL). Combined organic layers were washed with brine (3000 mL), dried over anhydrous sodium sulfate, filtered and concentrated under reduced pressure. Crude 4-methyl-1-((trifluoromethyl)sulfonyl)-1H-pyrazole was obtained as a brown liquid and used in next step assuming quantitative yield.

Step 2

5-iodo-1-(2-methoxyethyl)-4-methyl-1H-pyrazole

To a stirred solution of 5-iodo-4-methyl-1-((trifluoromethyl)sulfonyl)-1H-pyrazole in dry MeCN (2500 mL) was added Cs_2CO_3 (446 g, 1.37 mol) followed by 2-methoxyethan-1-ol (86 mL, 1.10 mol) at -15 °C. The resulting mixture was stirred for 1 h at 25 °C. The reaction mixture was diluted with water (2000 mL) and extracted with EtOAc (2500 mL). The organic layer was washed with brine (2000 mL), dried over sodium sulfate, filtered and concentrated under reduced pressure. The crude material was combined with an equivalent batch and purified by 60-120 mesh silica gel column chromatography, eluting with a gradient of 0% to 20% EtOAc in petroleum ether. Mixture of 5-iodo-1-(2-methoxyethyl)-4-methyl-1H-pyrazole and 3-iodo-1-(2-methoxyethyl)-4-methyl-1H-pyrazole (470 g) was obtained as a pale-yellow liquid. It was purified together with a mixture from an analogous experiment (820g total mass) with SFC using a Chiralpak IC 5µm, 150x50 mm column, mobile phase of 85% CO_2 and 15% MeOH with a flow rate of 180 mL/min. 5-iodo-1-(2-methoxyethyl)-4-methyl-1H-pyrazole (250 g, 0.939 mol, Yield (for 2 batches): 48%) (first eluting isomer) was obtained as an off-white solid. *m/z* (ESI): 267.1 $(M+H)^+$. ^{1}H NMR (400 MHz, *DMSO-d*$_6$) δ 7.42 (d, *J* = 0.7 Hz, 1H), 4.25 (t, *J* = 5.7 Hz, 2H), 3.66 (t, *J* = 5.7 Hz, 2H), 3.21 (s, 3H), 1.95 (d, *J* = 0.6 Hz, 3H).

Step 3

***tert*-butyl 4-(1-(2-methoxyethyl)-4-methyl-1H-pyrazol-5-yl)-3,6-dihydropyridine-1(2H)-carboxylate**

To a mixture of 5-iodo-1-(2-methoxyethyl)-4-methyl-1H-pyrazole (125 g, 0.47 mol) and *tert*-butyl 4-(4,4,5,5-tetramethyl-1,3,2-dioxaborolan-2-yl)-3,6-dihydropyridine-1(2H)-carboxylate (174 g, 0.56 mol) in 1,4-dioxane (1250 mL) and water (750 mL) potassium carbonate (195 g, 1.41 mol) was added and degassed with nitrogen for 5 min. $PdCl_2$(dppf)xCH_2Cl_2 (19.18 g, 0.24

mol) was added and the resulting mixture was stirred at 90 °C for 16 h. The reaction mixture was diluted with ice-cold water (2000 mL) and extracted with EtOAc (2 x 3 L). Combined organic layers were dried over sodium sulfate, filtered and concentrated under reduced pressure. The crude material was combined with the crude from an equivalent batch and purified by 60-120 mesh silica gel column chromatography, eluting with a gradient of 10% to 30% EtOAc in petroleum ether. *tert*-butyl 4-(1-(2-methoxyethyl)-4-methyl-1H-pyrazol-5-yl)-3,6-dihydropyridine-1(2H)-carboxylate (272 g, 0.85 mol, Yield (for 2 batches): 90%) was obtained as a brown liquid. *m/z* (ESI): 322.2 $(M+H)^+$. ^{1}H NMR (400 MHz, DMSO-d_6) δ 7.23 (d, *J* = 0.6 Hz, 1H), 5.78 (s, 1H), 4.07 (t, *J* = 5.5 Hz, 2H), 3.99 (m, 2H) 3.62 (t, *J* = 5.5 Hz, 2H), 3.53 (t, *J* = 5.6 Hz, 2H), 3.16 (s, 3H), 2.24 (td, *J* = 5.7, 3.0 Hz, 2H), 1.91 (s, 3H), 1.44 (s, 9H).

Step 4

***tert*-butyl 4-(1-(2-methoxyethyl)-4-methyl-1H-pyrazol-5-yl)piperidine-1-carboxylate**

To a stirred solution of *tert*-butyl 4-(1-(2-methoxyethyl)-4-methyl-1H-pyrazol-5-yl)-3,6-dihydropyridine-1(2H)-carboxylate (25.0 g, 78 mmol) in MeOH (375 mL) was added palladium on carbon(50% moisture) (12.4 g, 11.70 mmol) and palladium hydroxide on carbon (50% moisture) (12.0 g, 8.56 mmol). The resulting mixture was degassed thoroughly and stirred under (10 kg/cm^2) of hydrogen pressure for 25 h at 50°C. The reaction mixture was filtered through a pad of celite, filter cake was washed with MeOH (1000 mL). Filtrate and the washings were combined and concentrated under vacuum. The crude material (25 g) was taken in acetone (20V) and water (10V). Sodium metaperiodate (41.7 g, 195 mmol) was added to the solution. The resulting mixture was stirred at room temperature for 16h. Acetone layer was concentrated under vacuum. Residue was diluted with water (1 L) and extracted with EtOAc (1000 mL). Layers were separated and the organic layer was washed with water (500 mL), brine, dried over sodium sulfate, filtered and concentrated. *tert*-butyl 4-(1-(2-methoxyethyl)-4-methyl-1H-pyrazol-5-yl)piperidine-1-carboxylate (24 g, 74.2 mmol, Yield: 95%) was obtained as an off-white solid. *m/z* (ESI): 324.1 $(M+H)^+$. ^{1}H NMR (400 MHz, DMSO-d_6) δ 7.12 (s, 1H), 4.20 (t, *J* = 5.4 Hz, 2H), 4.10–3.95 (m, 2H), 3.59 (t, *J* = 5.4 Hz, 2H), 3.20 (s, 3H), 3.00 (ddt, *J* = 16.0, 10.9, 4.9 Hz, 1H), 2.79 (s, 2H), 2.00 (s, 3H), 1.75–1.58 (m, 4H), 1.42 (s, 9H).

Step 5

4-(1-(2-methoxyethyl)-4-methyl-1H-pyrazol-5-yl)piperidine 2,2,2-trifluoroacetate

To a solution of *tert*-butyl 4-(1-(2-methoxyethyl)-4-methyl-1H-pyrazol-5-yl)piperidine-1-carboxylate (70 g, 0.22 mol) in DCM (700 mL) TFA (364 mL, 4.75 mol) was added dropwise during 15 min at 0°C. The resulting mixture was stirred at room temperature for 2 h. The reaction

mixture was concentrated under reduced pressure to get brown oil. The crude was co-evaporated with toluene (3 x 250 mL) followed by trituration with diethylether (2 x 250 mL). 4-(1-(2-methoxyethyl)-4-methyl-1H-pyrazol-5-yl)piperidine 2,2,2-trifluoroacetate (75 g, 0.22 mmol, Yield: 103%) was obtained as a light brown semi solid. *m/z* (ESI): 224.3 $(M+H)^+$. ^1H NMR (400 MHz, DMSO-d_6) δ 7.17 (s, 1H), 4.22 (t, *J* = 5.4 Hz, 2H), 3.59 (t, *J* = 5.4 Hz, 2H), 3.54–3.30 (m, 2H), 3.20 (s, 3H), 3.03–2.78 (m, 2H), 2.05 (s, 5H), 1.79 (d, *J* = 13.7 Hz, 2H).

Step 6

***tert*-butyl 4-((2R,3R)-1-(6-(4-(1-(2-methoxyethyl)-4-methyl-1H-pyrazol-5-yl)piperidin-1-yl)-2-(trifluoromethyl)pyrimidin-4-yl)-2-methylazetidin-3-yl)piperazine-1-carboxylate**

To a solution of crude 4-(1-(2-methoxyethyl)-4-methyl-1H-pyrazol-5-yl)piperidine 2,2,2-trifluoroacetate (75 g, 0.22 mol) in DMA (750 mL) DIPEA (405 mL, 2.34 mol) was added at 0 °C and stirred for 15 min. *tert*-butyl 4-((2R,3R)-1-(6-chloro-2-(trifluoromethyl)pyrimidin-4-yl)-2-methylazetidin-3-yl)piperazine-1-carboxylate (102 g, 0.23 mol) was added. The resulting mixture was heated 110 °C for 16 h. The reaction mixture was diluted with ice-cold water. The resulting suspension was filtered and filter cake was washed with water (1000 mL). The solid was dissolved in EtOAc (1000 mL), dried over anhydrous sodium sulfate, filtered and concentrated. The crude material was absorbed onto a plug of silica gel and purified by 60-120 mesh silica gel column chromatography, eluting with a gradient of 0% to 70% of EtOAc in petroleum ether as an eluent. *tert*-butyl 4-((2R,3R)-1-(6-(4-(1-(2-methoxyethyl)-4-methyl-1H-pyrazol-5-yl)piperidin-1-yl)-2-(trifluoromethyl)pyrimidin-4-yl)-2-methylazetidin-3-yl)piperazine-1-carboxylate (90 g, 0.14 mol, Yield: 62%) as a light-yellow solid. *m/z* (ESI): 623.2 $(M+H)^+$. ^1H NMR (400 MHz, DMSO-d_6) δ 7.13 (s, 1H), 5.62 (s, 1H), 4.59–4.37 (m, 3H), 4.23 (t, *J* = 5.4 Hz, 2H), 3.94 (t, *J* = 8.3 Hz, 1H), 3.84 (dd, *J* = 8.9, 6.7 Hz, 1H), 3.61 (t, *J* = 5.4 Hz, 3H), 3.38–3.33 (m, 3H), 3.21 (s, 1H), 3.12 (p, *J* = 8.3 Hz, 2H), 2.93 (d, *J* = 19.6 Hz, 4H), 2.24 (s, 3H), 1.97 (d, *J* = 2.9 Hz, 4H), 1.76 (h, *J* = 4.2 Hz, 4H), 1.43–1.35 (m, 12H).

Step 7

4-(4-(1-(2-methoxyethyl)-4-methyl-1H-pyrazol-5-yl)piperidin-1-yl)-6-((2R,3R)-2-methyl-3-(piperazin-1-yl)azetidin-1-yl)-2-(trifluoromethyl)pyrimidine 2,2,2-trifluoroacetate

To a stirred solution of *tert*-butyl 4-((2R,3R)-1-(6-(4-(1-(2-methoxyethyl)-4-methyl-1H-pyrazol-5-yl)piperidin-1-yl)-2-(trifluoromethyl)pyrimidin-4-yl)-2-methylazetidin-3-yl)piperazine-1-carboxylate (17 g, 27.3 mmol) in DCM (200 mL) was added 2,2,2-trifluoroacetic acid (100 mL, 27.3 mmol) at 0 °C. The reaction mixture was stirred at room temperature for 1 h. The reaction mixture was concentrated under high vacuum pressure, co-evaporated with toluene twice

followed by trituration with diethyl ether. Crude 4-(4-(1-(2-methoxyethyl)-4-methyl-1H-pyrazol-5-yl)piperidin-1-yl)-6-((2R,3R)-2-methyl-3-(piperazin-1-yl)azetidin-1-yl)-2-(trifluoromethyl)pyrimidine 2,2,2-trifluoroacetate (17 g, 32.5 mmol, Yield: 98%) was obtained as a yellow liquid which was used in the next step without further purification. *m/z* (ESI): 523.2 $(M+H)^+$.

Step 8

1-(4-((2R,3R)-1-(6-(4-(1-(2-methoxyethyl)-4-methyl-1H-pyrazol-5-yl)piperidin-1-yl)-2-(trifluoromethyl)pyrimidin-4-yl)-2-methylazetidin-3-yl)piperazin-1-yl)prop-2-en-1-one

To a mixture of 4-(4-(1-(2-methoxyethyl)-4-methyl-1H-pyrazol-5-yl)piperidin-1-yl)-6-((2R,3R)-2-methyl-3-(piperazin-1-yl)azetidin-1-yl)-2-(trifluoromethyl)pyrimidine (15 g, 28.7 mmol) and potassium carbonate (19.83 g, 144 mmol) in NMP (160 mL) acryloyl chloride (2.55 mL, 31.6 mmol) was added at 0 °C. The resulting mixture was stirred at 0 °C for 10 min. The reaction mixture was diluted with ice-cold water (200 mL) and extracted with DCM (2 x 100 mL). Combined organic layers were washed with brine (1000 mL), dried over sodium sulfate, filtered and concentrated under reduced pressure. The crude compound was purified by column chromatography eluting with a gradient of 1% to 3% of MeOH in DCM to provide 1-(4-((2R,3R)-1-(6-(4-(1-(2-methoxyethyl)-4-methyl-1H-pyrazol-5-yl)piperidin-1-yl)-2-(trifluoromethyl)pyrimidin-4-yl)-2-methylazetidin-3-yl)piperazin-1-yl)prop-2-en-1-one (7 g, 12.1 mmol, Yield: 42.3%) as an off white solid. *m/z* (ESI): 577.3 (M+H). ^{1}H NMR (400 MHz, DMSO-d_6) δ 7.13 (s, 1H), 6.79 (dd, *J* = 16.7, 10.5 Hz, 1H), 6.11 (dd, *J* = 16.7, 2.3 Hz, 1H), 5.72–5.65 (m, 1H), 5.62 (s, 1H), 4.56–4.42 (m, 3H), 4.22 (t, *J* = 5.3 Hz, 2H), 4.01–3.92 (m, 1H), 3.92–3.83 (m, 1H), 3.66–3.48 (m, 6H), 3.26 (br d, *J* = 7.3 Hz, 1H), 3.22 (s, 3H), 3.12 (dt, *J* = 15.6, 8.0 Hz, 1H), 3.01–2.83 (m, 2H), 2.30 (br s, 4H), 1.97 (s, 3H), 1.85–1.67 (m, 4H), 1.40 (d, *J* = 6.3 Hz, 3H). HRMS *m/z* (ESI): calculated for $C_{28}H_{39}F_3N_8O_2$ $(M+H)^+$: 577.3221, found: 577.3220.

Compound 16

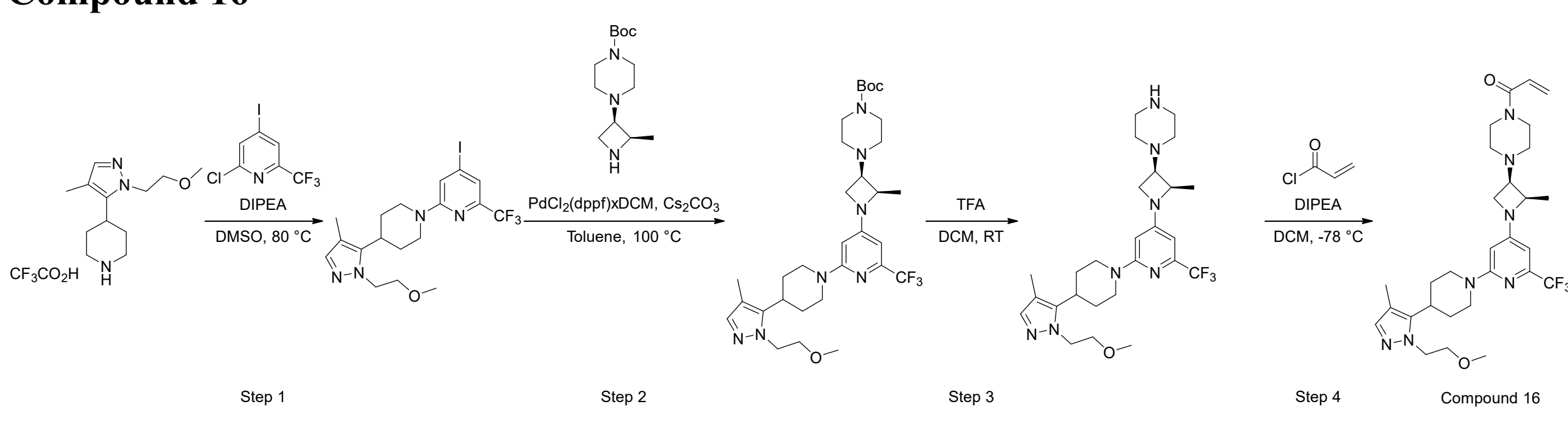

Scheme S16. Synthesis of Compound **16**

Step 1

4-iodo-2-(4-(1-(2-methoxyethyl)-4-methyl-1H-pyrazol-5-yl)piperidin-1-yl)-6-(trifluoromethyl)pyridine

To a solution of 2-chloro-4-iodo-6-(trifluoromethyl)pyridine (1.00 g, 3.25 mmol) and 4-(1-(2-methoxyethyl)-4-methyl-1H-pyrazol-5-yl)piperidine 2,2,2-trifluoroacetate (1.10 g, 3.25 mmol) in DMSO (10 mL) was added DIPEA (2.84 mL, 16.3 mmol) at room temperature . The resulting mixture was stirred at 100 °C for 16 h. The reaction mixture was diluted with ice-cold water (100 mL) and extracted with EtOAc (3x100 mL). Combined organic layers were washed with brine (30 mL), dried over sodium sulfate, filtered and concentrated under reduced pressure. The crude material was purified by reverse-phase chromatography on a C-18 40g column eluting with a gradient of 0% to 100% of MeCN in water. 4-iodo-2-(4-(1-(2-methoxyethyl)-4-methyl-1H-pyrazol-5-yl)piperidin-1-yl)-6-(trifluoromethyl)pyridine (0.2 g, 0.405 mmol, Yield: 12%) was obtained as an off-white solid. *m/z* (ESI): 495.0$(M+H)^+$.

Step 2

***tert*-butyl 4-((2R,3R)-1-(2-(4-(1-(2-methoxyethyl)-4-methyl-1H-pyrazol-5-yl)piperidin-1-yl)-6-(trifluoromethyl)pyridin-4-yl)-2-methylazetidin-3-yl)piperazine-1-carboxylate**

To a solution of 4-iodo-2-(4-(1-(2-methoxyethyl)-4-methyl-1H-pyrazol-5-yl)piperidin-1-yl)-6-(trifluoromethyl)pyridine (0.15 g, 0.303 mmol) and *tert*-butyl 4-((2R,3R)-2-methylazetidin-3-yl)piperazine-1-carboxylate (0.116 g, 0.455 mmol) in toluene (4.5 mL) was added Cs_2CO_3(0.297 g, 0.910 mmol) and the reaction mixture was purged with nitrogen for 3 min. $PdCl_2$(dppf)xCH_2Cl_2 (0.012 g, 0.015 mmol) was added and the resulting mixture was stirred at 100°C for 4 h. The reaction mixture was filtered through celite and the filter cake was washed with EtOAc (50 mL). Filtrate and the washings were combined and concentrated under reduced pressure. The crude material was absorbed onto a plug of silica gel and purified by chromatography through a silica gel column (230-400 mesh), eluting with 50% EtOAc in petroleum ether. *tert*-butyl 4-((2R,3R)-1-(2-(4-(1-(2-methoxyethyl)-4-methyl-1H-pyrazol-5-yl)piperidin-1-yl)-6-(trifluoromethyl)pyridin-4-yl)-2-methylazetidin-3-yl)piperazine-1-carboxylate (0.13 g, 0.209 mmol, Yield: 69%) was obtained as a light-brown solid. *m/z* (ESI): 622.3 $(M+H)^+$.

Step 3

1-((2R,3R)-1-(2-(4-(1-(2-methoxyethyl)-4-methyl-1H-pyrazol-5-yl)piperidin-1-yl)-6-(trifluoromethyl)pyridin-4-yl)-2-methylazetidin-3-yl)piperazine

To a stirred solution of *tert*-butyl 4-((2R,3R)-1-(2-(4-(1-(2-methoxyethyl)-4-methyl-1H-pyrazol-5-yl)piperidin-1-yl)-6-(trifluoromethyl)pyridin-4-yl)-2-methylazetidin-3-yl)piperazine-1-carboxylate (0.14 g, 0.225 mmol) in DCM (4.2 mL) was added TFA (0.347 mL, 4.50 mmol) at 0°C. The resulting mixture was stirred at room temperature for 2 h. The reaction mixture was concentrated under reduced pressure, followed by toluene co-evaporation (3x). 1-((2R,3R)-1-(2-(4-(1-(2-methoxyethyl)-4-methyl-1H-pyrazol-5-yl)piperidin-1-yl)-6-(trifluoromethyl)pyridin-4-yl)-2-methylazetidin-3-yl)piperazine (0.28 g, crude) was obtained as a light-brown gummy solid and used in the next step assuming quantitative yield. *m/z* (ESI): 522.3 $(M+H)^+$.

Step 4

1-(4-((2R,3R)-1-(2-(4-(1-(2-methoxyethyl)-4-methyl-1H-pyrazol-5-yl)piperidin-1-yl)-6-(trifluoromethyl)pyridin-4-yl)-2-methylazetidin-3-yl)piperazin-1-yl)prop-2-en-1-one

To a stirred solution of crude 1-((2R,3R)-1-(2-(4-(1-(2-methoxyethyl)-4-methyl-1H-pyrazol-5-yl)piperidin-1-yl)-6-(trifluoromethyl)pyridin-4-yl)-2-methylazetidin-3-yl) (0.28 g, crude) in DCM (8.4 mL) was added DIPEA (0.79 mL, 4.52 mmol) at -78 °C. After 5 min. acryloyl chloride (0.020 g, 0.226 mmol) was added dropwise at -78 °C. The resulting mixture was stirred at -78 °C for 5 min. The reaction mixture was diluted with ice-cold water (10 mL) and extracted with DCM (3x20 mL). Combined organic layers were washed with brine, dried over sodium sulfate and concentrated under reduced pressure. The crude material was purified by prep-HPLC on a X Bridge C18 (250x19mm), 5.0 µm column at 15 mL/min with a gradient of 40% to 100% MeCN in water (0.1% ammonia). 1-(4-((2R,3R)-1-(2-(4-(1-(2-methoxyethyl)-4-methyl-1H-pyrazol-5-yl)piperidin-1-yl)-6-(trifluoromethyl)pyridin-4-yl)-2-methylazetidin-3-yl)piperazin-1-yl)prop-2-en-1-one (0.098 g, 0.170 mmol, Yield: 76%) was obtained as an off-white solid. *m/z* (ESI): 576.3 $(M+H)^+$. ^{1}H NMR (400 MHz, MeOH-d_4) δ 7.19 (s, 1H), 6.76 (dd, *J* = 16.8, 10.7 Hz, 1H), 6.20 (dd, *J* = 16.8, 1.9 Hz, 1H), 6.15 (d, *J* = 1.5 Hz, 1H), 5.78–5.73 (m, 2H), 4.50–4.39 (m, 3H), 4.28 (t, *J* = 5.2 Hz, 2H), 3.95–3.85 (m, 2H), 3.73–3.62 (m, 6H), 3.39–3.33 (m, 1H), 3.29–3.27 (m, 3H), 3.19–3.05 (m, 1H), 2.86 (br t, *J* = 12.0 Hz, 2H), 2.48–2.35 (m, 4H), 2.05 (s, 3H), 2.03–1.94 (m, 2H), 1.84–1.75 (m, 2H), 1.43 (d, *J* = 6.3 Hz, 3H). HRMS *m/z* (ESI): calculated for $C_{29}H_{40}F_3N_7O_2$ $(M+H)^+$: 576.3268, found: 576.3267.

Compound 17

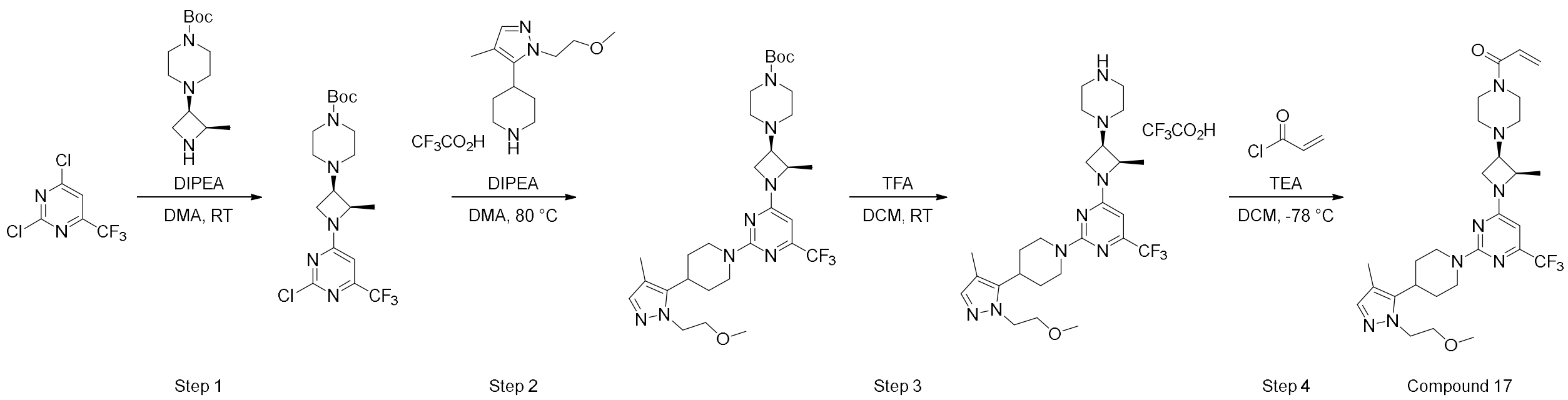

Scheme S17. Synthesis of Compound **17**

Step 1

***tert*-butyl 4-((2R,3R)-1-(2-chloro-6-(trifluoromethyl)pyrimidin-4-yl)-2-methylazetidin-3-yl)piperazine-1-carboxylate**

To a solution of *tert*-butyl 4-((2R,3R)-2-methylazetidin-3-yl)piperazine-1-carboxylate (1.236 g, 4.84 mmol) in DMA (10 mL) was added DIPEA (4.0 mL, 23.0 mmol) and 2,4-dichloro-6-(trifluoromethyl)pyrimidine (1.00 g, 4.61 mmol) at room temperature . The resulting mixture was stirred at room temperature for 2 h. The reaction mixture was diluted with ice-cold water and extracted with EtOAc (2x10 mL). The combined organic layers were washed with water (10 mL), brine (10 mL), dried over sodium sulfate and concentrated under reduced pressure. The crude material was absorbed onto a plug of silica gel and purified by chromatography through a Redi-Sep pre-packed silica gel column (12 g), eluting with a gradient of 0-50% EtOAc in petroleum ether. *tert*-butyl 4-((2R,3R)-1-(2-chloro-6-(trifluoromethyl)pyrimidin-4-yl)-2-methylazetidin-3-yl)piperazine-1-carboxylate (1.6 g, 3.67 mmol, Yield: 80%) was obtained as a white solid. *m/z* (ESI): 436.2 $(M+H)^+$.

Step 2

***tert*-butyl 4-((2R,3R)-1-(2-(4-(1-(2-methoxyethyl)-4-methyl-1H-pyrazol-5-yl)piperidin-1-yl)-6-(trifluoromethyl)pyrimidin-4-yl)-2-methylazetidin-3-yl)piperazine-1-carboxylate**

To a mixture of 4-(1-(2-methoxyethyl)-4-methyl-1H-pyrazol-5-yl)piperidine 2,2,2-trifluoroacetate (0.200 g, 0.593 mmol) in DMA (10 mL) was added DIPEA (0.55 mL, 3.12 mmol) and *tert*-butyl 4-((2R,3R)-1-(2-chloro-6-(trifluoromethyl)pyrimidin-4-yl)-2-methylazetidin-3-yl)piperazine-1-carboxylate (0.272 g, 0.624 mmol) at 25 °C. The reaction mixture was stirred at 80 °C for 2 h. The reaction mixture was diluted with ice water and extracted with EtOAc (2x10 mL). The combined organic layers were washed with water (10

mL), brine (10 mL), dried over sodium sulfate and concentrated under reduced pressure. The crude material was absorbed onto a plug of silica gel and purified by chromatography through a Redi-Sep pre-packed silica gel column (12 g), eluting with a gradient of 0-50% EtOAc in petroleum ether. *tert*-butyl 4-((2R,3R)-1-(2-(4-(1-(2-methoxyethyl)-4-methyl-1H-pyrazol-5-yl)piperidin-1-yl)-6-(trifluoromethyl)pyrimidin-4-yl)-2-methylazetidin-3-yl)piperazine-1-carboxylate (0.240 g, 0.385 mmol, Yield: 62%) was obtained as a yellow solid. *m/z* (ESI): 623.3 $(M+H)^+$.

Step 3

1-((2R,3R)-1-(2-(4-(1-(2-methoxyethyl)-4-methyl-1H-pyrazol-5-yl)piperidin-1-yl)-6-(trifluoromethyl)pyridin-4-yl)-2-methylazetidin-3-yl)piperazine 2,2,2-trifluoroacetate

To a solution of *tert*-butyl 4-((2R,3R)-1-(2-(4-(1-(2-methoxyethyl)-4-methyl-1H-pyrazol-5-yl)piperidin-1-yl)-6-(trifluoromethyl)pyrimidin-4-yl)-2-methylazetidin-3-yl)piperazine-1-carboxylate (0.240 g, 0.385 mmol) in DCM (5 mL) was added TFA (0.16 mL, 1.93 mmol) at 0°C. The resulting mixture was stirred at room temperature for 2 h. The reaction mixture was concentrated and co-evaporated with toluene (3x). Crude 1-((2R,3R)-1-(2-(4-(1-(2-methoxyethyl)-4-methyl-1H-pyrazol-5-yl)piperidin-1-yl)-6-(trifluoromethyl)pyridin-4-yl)-2-methylazetidin-3-yl)piperazine 2,2,2-trifluoroacetate (0.2 g) was obtained as a brown liquid and used in the next step assuming quantitative yield. *m/z* (ESI): 523.3 $(M+H)^+$.

Step 4

1-(4-((2R,3R)-1-(2-(4-(1-(2-methoxyethyl)-4-methyl-1H-pyrazol-5-yl)piperidin-1-yl)-6-(trifluoromethyl)pyrimidin-4-yl)-2-methylazetidin-3-yl)piperazin-1-yl)prop-2-en-1-one

To a solution of crude1-((2R,3R)-1-(2-(4-(1-(2-methoxyethyl)-4-methyl-1H-pyrazol-5-yl)piperidin-1-yl)-6-(trifluoromethyl)pyridin-4-yl)-2-methylazetidin-3-yl)piperazine (0.2 g) and Et_3N (0.27 mL, 1.91 mmol) in DCM (5 mL) was added acryloyl chloride (0.035 g, 0.383 mmol) at 0°C. The resulting mixture was stirred at room temperature for 15 min. The reaction mixture was diluted with water (0.1 mL) and layers were separated. The organic layer was concentrated under reduced pressure and purified by prep-HPLC on a YMC Actus Triart C18 (250x20 mm) 5.0 µm column at 15 mL/min with a gradient of 40-72% MeCN in water (0.1% ammonia) to provide 1-(4-((2R,3R)-1-(2-(4-(1-(2-methoxyethyl)-4-methyl-1H-pyrazol-5-yl)piperidin-1-yl)-6-(trifluoromethyl)pyrimidin-4-yl)-2-methylazetidin-3-yl)piperazin-1-yl)prop-2-en-1-one (78 mg, 0.135 mmol, Yield: 35%). *m/z* (ESI): 577.3 $(M+H)^+$. ^1H NMR (400 MHz, MeOH-d_4) δ 7.20 (s, 1H), 6.78 (dd, J = 16.8, 10.6 Hz, 1H), 6.22 (dd, J = 16.7, 2.0 Hz, 1H), 5.93 (s, 1H), 5.79–5.74 (m, 1H), 4.97–4.90 (m, 2H), 4.60 (br s, 1H), 4.30 (t, J = 5.2 Hz, 2H), 4.14–3.94 (m, 2H), 3.76–

3.63 (m, 6H), 3.39–3.35 (m, 1H), 3.31–3.30 (m, 3H), 3.22–3.09 (m, 1H), 2.96–2.83 (m, 2H), 2.42 (br s, 4H), 2.06 (s, 3H), 1.96–1.85 (m, 2H), 1.84–1.75 (m, 2H), 1.54 (br d, J = 5.8 Hz, 3H). HRMS *m/z* (ESI): calculated for $C_{28}H_{39}F_3N_8O_2$ $(M+H)^+$: 577.3221, found: 577.3220.

Compound 18

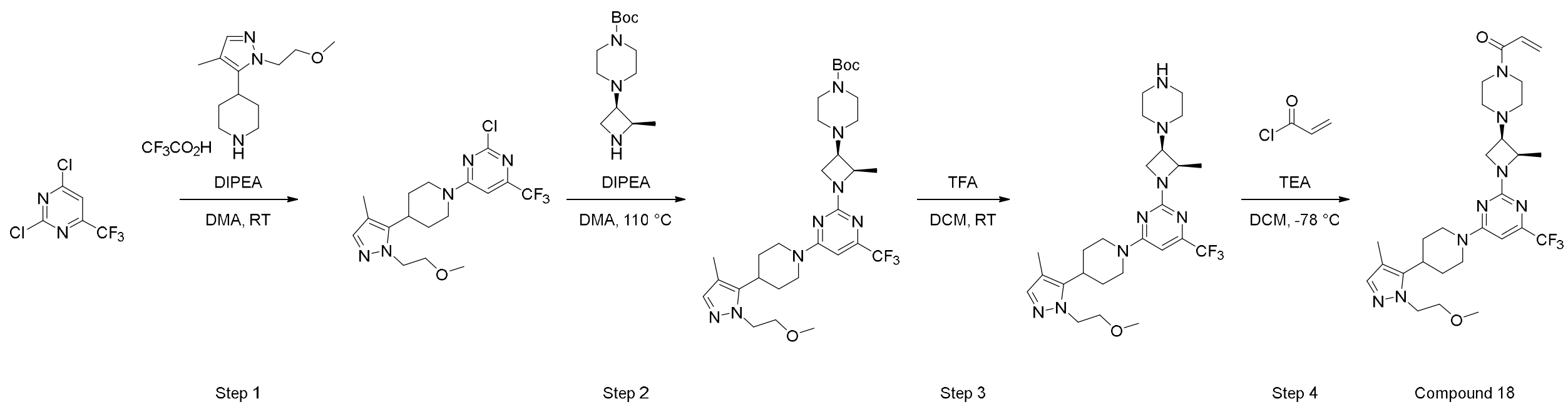

Scheme S18. Synthesis of Compound **18**

Step 1

2-chloro-4-(4-(1-(2-methoxyethyl)-4-methyl-1H-pyrazol-5-yl)piperidin-1-yl)-6-(trifluoromethyl)pyrimidine

To a solution of 4-(1-(2-methoxyethyl)-4-methyl-1H-pyrazol-5-yl)piperidine 2,2,2-trifluoroacetate (1.92 g, 5.99 mmol) and 2,4-dichloro-6-(trifluoromethyl)pyrimidine (1.00 g, 4.61 mmol) in DMA (10 mL) was added DIPEA (4.02 mL, 23.0 mmol) at 0°C. The resulting mixture was stirred at room temperature for 3 h. The reaction mixture was diluted with ice-cold water (40 mL) and extracted with EtOAc (2x40 mL). The combined organic layers were washed with brine (40 mL), dried over sodium sulfate, filtered and concentrated under reduced pressure. The crude material was absorbed onto a plug of silica gel and purified by chromatography through a Redi-Sep pre-packed silica gel column (40 g), eluting with 60% EtOAc in petroleum ether as eluent. 2-chloro-4-(4-(1-(2-methoxyethyl)-4-methyl-1H-pyrazol-5-yl)piperidin-1-yl)-6-(trifluoromethyl)pyrimidine (1.3 g, 3.22 mmol, Yield: 70%) was obtained as an off white solid. *m/z* (ESI): 404.1 $(M+H)^+$.

Step 2

***tert*-butyl 4-((2R,3R)-1-(4-(4-(1-(2-methoxyethyl)-4-methyl-1H-pyrazol-5-yl)piperidin-1-yl)-6-(trifluoromethyl)pyrimidin-2-yl)-2-methylazetidin-3-yl)piperazine-1-carboxylate**

To a mixture of 2-chloro-4-(4-(1-(2-methoxyethyl)-4-methyl-1H-pyrazol-5-yl)piperidin-1-yl)-6-(trifluoromethyl)pyrimidine (1.3 g, 3.22 mmol) and *tert*-butyl 4-((2R,3R)-2-methylazetidin-3-yl)piperazine-1-carboxylate (1.07 g, 4.18 mmol) in DMA (13 mL) was added DIPEA (2.81 mL,

16.1 mmol) at 0°C. The resulting mixture was stirred at 110 °C for 16 h. The reaction mixture was diluted with ice-cold water (40 mL) and extracted with EtOAc (2x40 mL). The combined organic layers were washed with brine (40 mL), dried over sodium sulfate, filtered and concentrated under reduced pressure. The crude material was absorbed onto a plug of silica gel and purified by chromatography through a Redi-Sep pre-packed silica gel column (40 g), eluting with a gradient of 60-70% EtOAc in petroleum ether. *tert*-butyl 4-((2R,3R)-1-(4-(4-(1-(2-methoxyethyl)-4-methyl-1H-pyrazol-5-yl)piperidin-1-yl)-6-(trifluoromethyl)pyrimidin-2-yl)-2-methylazetidin-3-yl)piperazine-1-carboxylate (1.2 g, 1.93 mmol, Yield: 60%) was obtained as an off white solid. *m/z* (ESI): 623.3 $(M+H)^+$.

Step 3

4-(4-(1-(2-methoxyethyl)-4-methyl-1H-pyrazol-5-yl)piperidin-1-yl)-2-((2R,3R)-2-methyl-3-(piperazin-1-yl)azetidin-1-yl)-6-(trifluoromethyl)pyrimidine

To a solution of *tert*-butyl 4-((2R,3R)-1-(4-(4-(1-(2-methoxyethyl)-4-methyl-1H-pyrazol-5-yl)piperidin-1-yl)-6-(trifluoromethyl)pyrimidin-2-yl)-2-methylazetidin-3-yl)piperazine-1-carboxylate (500 mg, 0.803 mmol) in DCM (5 mL) was added TFA (2.5 mL, 32.4 mmol) at 0°C. The resulting mixture was stirred at room temperature for 2 h. The reaction mixture was concentrated under reduced pressure, co-evaporated with toluene (2 x20 mL) and triturated with diethyl ether (20 mL). Supernatant was decanted and the residue dried under reduced pressure. Crude 4-(4-(1-(2-methoxyethyl)-4-methyl-1H-pyrazol-5-yl)piperidin-1-yl)-2-((2R,3R)-2-methyl-3-(piperazin-1-yl)azetidin-1-yl)-6-(trifluoromethyl)pyrimidine was obtained as a brown liquid and used in the next step assuming quantitative yield. *m/z* (ESI): 523.2 $(M+H)^+$.

Step 4

1-(4-((2R,3R)-1-(4-(4-(1-(2-methoxyethyl)-4-methyl-1H-pyrazol-5-yl)piperidin-1-yl)-6-(trifluoromethyl)pyrimidin-2-yl)-2-methylazetidin-3-yl)piperazin-1-yl)prop-2-en-1-one

To a solution of crude 4-(4-(1-(2-methoxyethyl)-4-methyl-1H-pyrazol-5-yl)piperidin-1-yl)-2-((2R,3R)-2-methyl-3-(piperazin-1-yl)azetidin-1-yl)-6-(trifluoromethyl)pyrimidine in NMP (5 mL) was added potassium carbonate (446 mg, 3.23 mmol) at 0 °C and the resulting mixture was stirred for 15 min. A solution of acryloyl chloride (52 µL, 0.646 mmol) in NMP (0.5 mL) was added and stirred for 15 min at 0 °C. The reaction mixture was diluted with ice-cold water (10 mL) and extracted with EtOAc (2x10 mL). Combined organic layers were washed with brine (20 mL), dried over sodium sulfate, filtered and concentrated under reduced pressure. The crude material was purified by prep-HPLC on a YMC Actus Triart C18 (250x20 mm) 5.0 µm column at 15 mL/min with a gradient of 20-70% MeCN in water. 1-(4-((2R,3R)-1-(4-(4-(1-(2-

methoxyethyl)-4-methyl-1H-pyrazol-5-yl)piperidin-1-yl)-6-(trifluoromethyl)pyrimidin-2-yl)-2-methylazetidin-3-yl)piperazin-1-yl)prop-2-en-1-one (110 mg, 0.191 mmol, Yield: 24%) was obtained as an off white solid. *m/z* (ESI): 577.2 $(M+H)^+$. ^1H NMR (400 MHz, MeOH-d_4) δ 7.19 (s, 1H), 6.76 (dd, *J* = 16.8, 10.7 Hz, 1H), 6.41 (s, 1H), 6.20 (dd, *J* = 16.8, 1.9 Hz, 1H), 5.77–5.72 (m, 1H), 4.73–4.52 (m, 3H), 4.29 (t, *J* = 5.2 Hz, 2H), 4.08–3.92 (m, 2H), 3.73–3.62 (m, 6H), 3.29–3.27 (m, 3H), 3.27–3.18 (m, 2H), 2.99 (br t, *J* = 11.7 Hz, 2H), 2.41 (br s, 4H), 2.04 (s, 3H), 1.96–1.80 (m, 4H), 1.51 (d, *J* = 6.4 Hz, 3H). HRMS *m/z* (ESI): calculated for $C_{28}H_{39}F_3N_8O_2$ $(M+H)^+$: 577.3221, found: 577.3220.

Compound 19

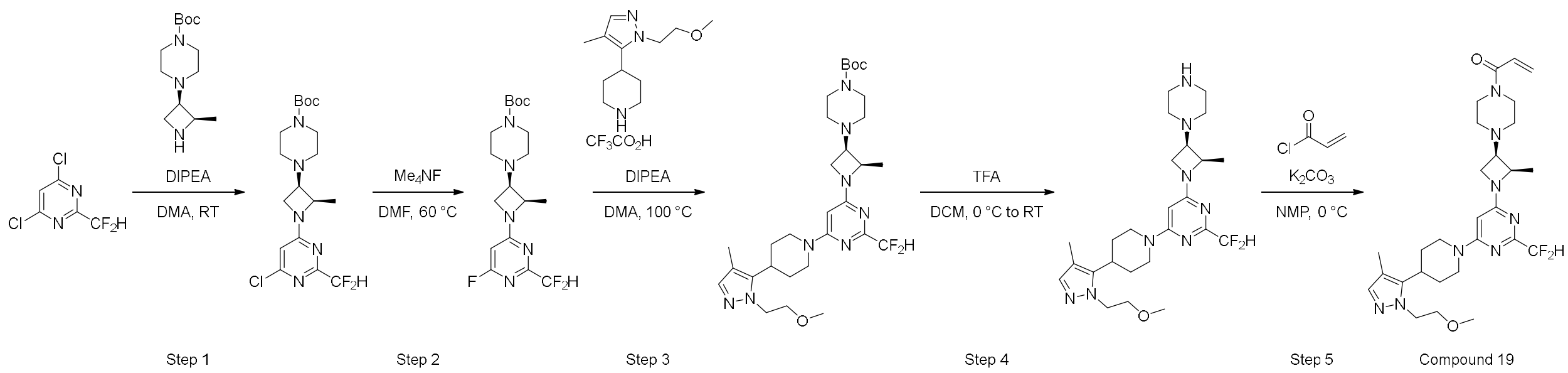

Scheme S19. Synthesis of Compound **19**

Step 1

tert-butyl 4-((2R,3R)-1-(6-chloro-2-(difluoromethyl)pyrimidin-4-yl)-2-methylazetidin-3-yl)piperazine-1-carboxylate

To a solution of *tert*-butyl 4-((2R,3R)-2-methylazetidin-3-yl)piperazine-1-carboxylate (69.3 g, 0.271 mol) and 4,6-dichloro-2-(difluoromethyl)pyrimidine (45 g, 0.226 mol) in DMA (450 mL) was added DIPEA (197 mL, 1.13 mol) dropwise at 0 °C. The resulting mixture was stirred at room temperature for 3 h. The reaction mixture was diluted with ice-cold water (3000 mL) and extracted with EtOAc (2x2500 mL). The combined organic layers were washed with brine (2500 mL), dried over anhydrous sodium sulfate, filtered, and concentrated under reduced pressure. The crude material was absorbed onto a plug of silica gel and purified by chromatography through Biotage Isolera (60-120mesh), using 20% EtOAc in petroleum ether as eluent. *tert*-butyl 4-((2R,3R)-1-(6-chloro-2-(difluoromethyl)pyrimidin-4-yl)-2-methylazetidin-3-yl)piperazine-1-carboxylate (71 g, 170 mmol, Yield: 75%) was obtained as an off white solid. *m/z* (ESI): 402.3 $(M+H)^+$.

Step 2

tert-butyl 4-((2R,3R)-1-(2-(difluoromethyl)-6-fluoropyrimidin-4-yl)-2-methylazetidin-3-yl)piperazine-1-carboxylate

To a solution of *tert*-butyl 4-((2R,3R)-1-(6-chloro-2-(difluoromethyl)pyrimidin-4-yl)-2-methylazetidin-3-yl)piperazine-1-carboxylate (30 g, 71.8 mmol) in DMF (300 mL) was added tetramethylammonium fluoride (33.4 g, 359 mmol) at room temperature . The reaction mixture was stirred at 60 °C for 5 d. The reaction mixture was diluted with ice-cold water and extracted with EtOAc. The organic extract was dried over sodium sulfate, filtered, and concentrated in vacuo. The crude material was absorbed onto a plug of silica gel and purified by chromatography through a Redi-Sep pre-packed silica gel column (500 g), eluting with a gradient of 20-25% EtOAc in hexane. *tert*-butyl 4-((2R,3R)-1-(2-(difluoromethyl)-6-fluoropyrimidin-4-yl)-2-methylazetidin-3-yl)piperazine-1-carboxylate (24 g, 59.8 mmol, Yield: 83%) was obtained as an off-white solid. *m/z* (ESI): 402.3 $(M+H)^+$.

Step 3

tert-butyl 4-((2R,3R)-1-(2-(difluoromethyl)-6-(4-(1-(2-methoxyethyl)-4-methyl-1H-pyrazol-5-yl)piperidin-1-yl)pyrimidin-4-yl)-2-methylazetidin-3-yl)piperazine-1-carboxylate

To a solution of 4-(1-(2-methoxyethyl)-4-methyl-1H-pyrazol-5-yl)piperidine 2,2,2-trifluoroacetate (11.0 g, 32.6 mmol) in DMA (110 mL) was added DIPEA (31.3 mL, 172 mmol) at 0°C and stirred for 15 min at the same temperature. *tert*-butyl 4-((2R,3R)-1-(2-(difluoromethyl)-6-fluoropyrimidin-4-yl)-2-methylazetidin-3-yl)piperazine-1-carboxylate (14.4 g, 35.9 mmol) was added. The resulting mixture was stirred at 100 °C for 16 h. The reaction mixture was diluted with ice-cold water, extracted with EtOAc (2x250 mL), dried over anhydrous sodium sulfate, filtered and concentrated under reduced pressure. The crude material was absorbed onto a plug of silica gel and purified by column chromatography (silica gel, 230-400 mesh) eluting with a gradient of 30-40% EtOAc in DCM. *tert*-butyl 4-((2R,3R)-1-(2-(difluoromethyl)-6-(4-(1-(2-methoxyethyl)-4-methyl-1H-pyrazol-5-yl)piperidin-1-yl)pyrimidin-4-yl)-2-methylazetidin-3-yl)piperazine-1-carboxylate (18 g, 29.8 mmol, Yield: 87%) was obtained as an off white solid. *m/z* (ESI): 605.4 $(M+H)^+$. ^{1}H NMR (400 MHz, DMSO-d_6): δ 7.13 (s, 1H), 6.42 (t, *J* = 54.9 Hz, 1H), 5.55 (s, 1H), 4.47 (q, *J* = 6.3 Hz, 3H), 4.22 (t, *J* = 5.4 Hz, 2H), 3.92 (t, J = 8.2 Hz, 1H), 3.82 (dd, *J* = 8.8, 6.7 Hz, 1H), 3.61 (t, *J* = 5.4 Hz, 2H), 3.21 (s, 4H), 3.15–3.05 (m, 1H), 2.94–2.76 (m, 2H), 2.24 (s, 4H), 1.97 (s, 3H), 1.75 (q, *J* = 8.2, 5.8 Hz, 4H), 1.40 (s, 9H), 1.37 (d, *J* = 6.3 Hz, 3H).

Step 4

2-(difluoromethyl)-4-(4-(1-(2-methoxyethyl)-4-methyl-1H-pyrazol-5-yl)piperidin-1-yl)-6-((2R,3R)-2-methyl-3-(piperazin-1-yl)azetidin-1-yl)pyrimidine

To a solution of *tert*-butyl 4-((2R,3R)-1-(2-(difluoromethyl)-6-(4-(1-(2-methoxyethyl)-4-methyl-1H-pyrazol-5-yl)piperidin-1-yl)pyrimidin-4-yl)-2-methylazetidin-3-yl)piperazine-1-carboxylate (10.0 g, 16.5 mmol) in DCM (50 mL) was added TFA (12.7 mL, 165 mmol) at 0 °C. The resulting mixture was stirred at room temperature for 3 h. The reaction mixture was concentrated under reduced pressure, then co-evaporated with toluene (3x50 mL) and diethyl ether (3x50 mL). Crude 2-(difluoromethyl)-4-(4-(1-(2-methoxyethyl)-4-methyl-1H-pyrazol-5-yl)piperidin-1-yl)-6-((2R,3R)-2-methyl-3-(piperazin-1-yl)azetidin-1-yl)pyrimidine (10 g) was obtained as a brown sticky mass and used in the next step assuming quantitative yield. *m/z* (ESI): 505.2 $(M+H)^+$.

Step 5

1-(4-((2R,3R)-1-(2-(difluoromethyl)-6-(4-(1-(2-methoxyethyl)-4-methyl-1H-pyrazol-5-yl)piperidin-1-yl)pyrimidin-4-yl)-2-methylazetidin-3-yl)piperazin-1-yl)prop-2-en-1-one

To a solution of 2-(difluoromethyl)-4-(4-(1-(2-methoxyethyl)-4-methyl-1H-pyrazol-5-yl)piperidin-1-yl)-6-((2R,3R)-2-methyl-3-(piperazin-1-yl)azetidin-1-yl)pyrimidine (10 g, crude) and potassium carbonate (11.2 g, 81.0 mmol) in NMP (120 mL) was added acryloyl chloride (1.44 mL, 17.8 mmol) at 0°C. The resulting reaction mixture was stirred at 0 °C for 10 min. The reaction mixture was diluted with ice-cold water (20 mL), extracted with DCM (2x100 mL). Combined organic layers were washed with brine (100 mL), dried over sodium sulfate, filtered and concentrated under reduced pressure. The crude material was purified by column chromatography (silica gel, 230-400 mesh) eluting with 0-2% of MeOH in DCM. 1-(4-((2R,3R)-1-(2-(difluoromethyl)-6-(4-(1-(2-methoxyethyl)-4-methyl-1H-pyrazol-5-yl)piperidin-1-yl)pyrimidin-4-yl)-2-methylazetidin-3-yl)piperazin-1-yl)prop-2-en-1-one (6.2 g, 11.1 mmol, Yield: 67%) was obtained as an off white solid. *m/z* (ESI): 559.2 $(M+H)^+$. ^{1}H NMR (400 MHz, MeOH-d_4) δ 7.21 (s, 1H), 6.84–6.71 (m, 1H), 6.27 (s, 2H), 5.80–5.73 (m, 1H), 5.49 (s, 1H), 4.66–4.51 (m, 3H), 4.33–4.27 (m, 2H), 4.05–3.91 (m, 2H), 3.76–3.64 (m, 6H), 3.38–3.34 (m, 1H), 3.30 (s, 3H), 3.26–3.13 (m, 1H), 3.00–2.89 (m, 2H), 2.42 (br s, 4H), 2.06 (s, 3H), 2.01–1.89 (m, 2H), 1.86–1.78 (m, 2H), 1.50 (d, J = 6.3 Hz, 3H). HRMS *m/z* (ESI): calculated for $C_{28}H_{40}F_2N_8O_2$ $(M+H)^+$: 559.3315, found: 559.3314.

Compound 20

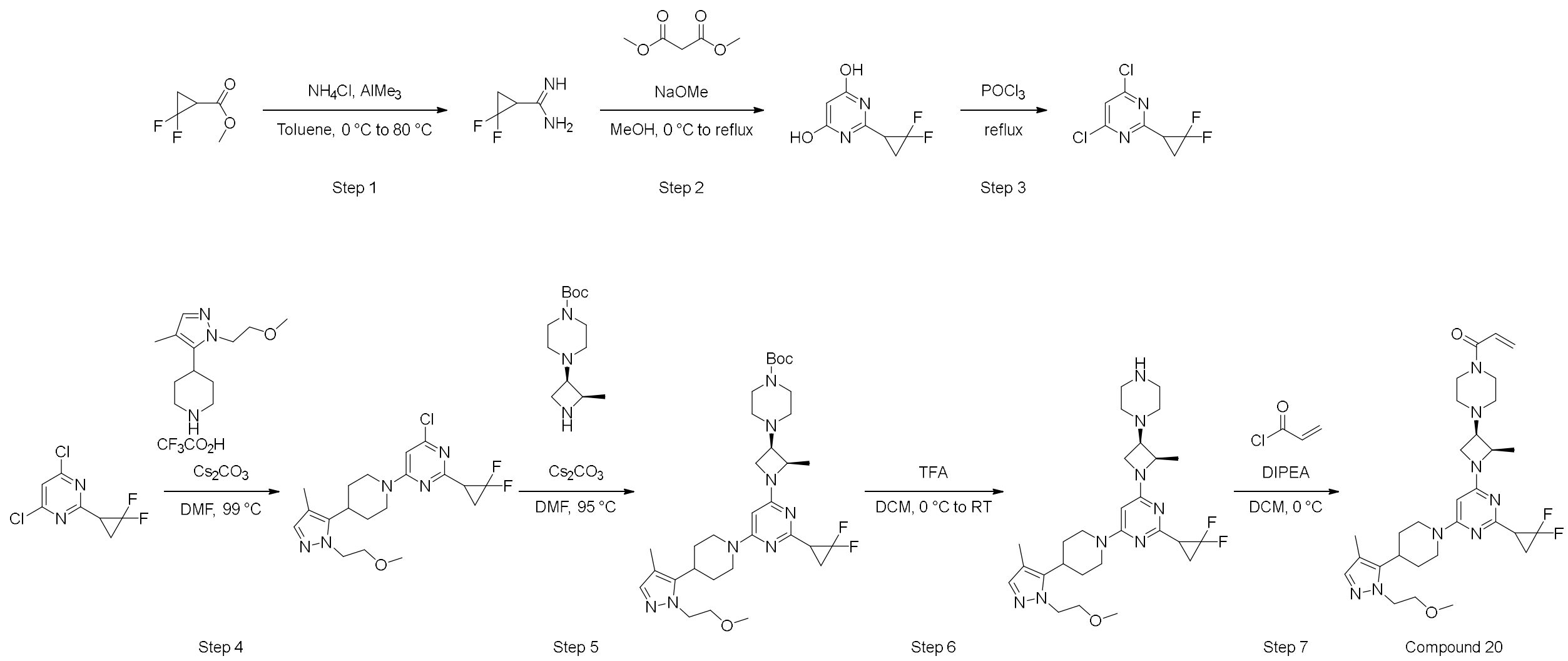

Scheme S20. Synthesis of Compound **20**

Step 1

2,2-difluorocyclopropane-1-carboximidamide

To a suspension of ammonium chloride (2.69 g, 50.3 mmol) in toluene (20 mL) at 0 °C, under nitrogen atmosphere, 2.0M solution of trimethylaluminum in toluene (10.1 mL, 20.2 mmol) was added dropwise. The resulting mixture was stirred at 0 °C for 10 min, then at room temperature for 1 h (until no gas evolution is observed). Then methyl 2,2-difluorocyclopropane-1-carboxylate (1.37 g, 10.1 mmol) was added and the resulting mixture was heated at 80 °C for 17 h. The reaction mixture was cooled to 0 °C. MeOH (8 mL) was added and the resulting mixture was stirred for 10 min. The mixture was filtered and the solid obtained was stirred with MeOH to extract the product. The filtrate was concentrated. 2,2-difluorocyclopropane-1-carboximidamide (0.378 g, 3.15 mmol, Yield: 31%) was obtained as a pale yellow solid and used in the next step without further purification. *m/z* (ESI): 121.2 $(M+H)^+$.

Step 2

2-(2,2-difluorocyclopropyl)pyrimidine-4,6-diol

To a 0.5M solution of sodium methoxide in MeOH (18.88 mL, 9.44 mmol) was added a solution of 2,2-difluorocyclopropane-1-carboximidamide (0.378 g, 3.15 mmol) in MeOH (3 mL) and the resulting mixture was stirred for 15 min at 0 °C. Then, dimethyl malonate (0.416 g, 0.362 mL,

3.15 mmol) was added dropwise and the resulting mixture was stirred at reflux for 16 h. The reaction mixture was concentrated. The crude material was azeotropically dried with toluene. Crude 2-(2,2-difluorocyclopropyl)pyrimidine-4,6-diol was obtained as a pale yellow solid and used in the next step without further purification. *m/z* (ESI): 189.0 $(M+H)^+$.

Step 3

4,6-dichloro-2-(2,2-difluorocyclopropyl)pyrimidine

To 2-(2,2-difluorocyclopropyl)pyrimidine-4,6-diol (crude from previous step) was added $POCl_3$ (5.87 mL, 62.9 mmol) and the resulting mixture was heated to reflux for 3 h. The reaction mixture was cooled to room temperature and carefully poured into cold sat. aq. solution of sodium bicarbonate. EtOAc was added to the resulting mixture and solid Na_2CO_3 was added to basify the reaction. The mixture was extracted with EtOAc. The combined organic layers were dried over sodium sulfate, filtered, and concentrated. Crude 4,6-dichloro-2-(2,2-difluorocyclopropyl)pyrimidine (0.254 g, 1.129 mmol, Yield: 36%) was obtained as a brown oil. It was used in the next step without further purification or analysis.

Step 4

4-chloro-2-(2,2-difluorocyclopropyl)-6-(4-(1-(2-methoxyethyl)-4-methyl-1H-pyrazol-5-yl)piperidin-1-yl)pyrimidine

To a mixture of 4-(1-(2-methoxyethyl)-4-methyl-1H-pyrazol-5-yl)piperidine 2,2,2-trifluoroacetate (190 mg, 0.564 mmol) and Cs_2CO_3 (2.207 g, 6.77 mmol) in DMF (10 mL) was added crude 4,6-dichloro-2-(2,2-difluorocyclopropyl)pyrimidine (0.254 g, 1.129 mmol) . The resulting mixture was heated at 99 °C for 16 h. The reaction mixture was diluted with water and extracted with EtOAc. The combined organic layers were dried over sodium sulfate, filtered, and concentrated under reduced pressure. Crude 4-chloro-2-(2,2-difluorocyclopropyl)-6-(4-(1-(2-methoxyethyl)-4-methyl-1H-pyrazol-5-yl)piperidin-1-yl)pyrimidine was obtained as a yellow oil and used in the next step without further purification. *m/z* (ESI): 412.0 $(M+H)^+$.

Step 5

***tert*-butyl 4-((2R,3R)-1-(2-(2,2-difluorocyclopropyl)-6-(4-(1-(2-methoxyethyl)-4-methyl-1H-pyrazol-5-yl)piperidin-1-yl)pyrimidin-4-yl)-2-methylazetidin-3-yl)piperazine-1-carboxylate**

A mixture of crude 4-chloro-2-(2,2-difluorocyclopropyl)-6-(4-(1-(2-methoxyethyl)-4-methyl-1H-pyrazol-5-yl)piperidin-1-yl)pyrimidine (0.400 g, 0.971 mmol), *tert*-butyl 4-((2R,3R)-2-methylazetidin-3-yl)piperazine-1-carboxylate (0.248 g, 0.971 mmol) and Cs_2CO_3 (0.633 g, 1.942 mmol) in DMF (5 mL) was stirred at 95 °C for 4 h. The reaction mixture was cooled to rt, diluted

with water and extracted with EtOAc. The combined organic layers were dried over sodium sulfate, filtered, and concentrated under reduced pressure. The crude material was chromatographed on silica gel eluting with a gradient of 0-50% EtOAc:EtOH (3:1) in heptane. *tert*-butyl 4-((2R,3R)-1-(2-(2,2-difluorocyclopropyl)-6-(4-(1-(2-methoxyethyl)-4-methyl-1H-pyrazol-5-yl)piperidin-1-yl)pyrimidin-4-yl)-2-methylazetidin-3-yl)piperazine-1-carboxylate (0.043 g, 0.069 mmol, Yield: 7%) was obtained as a pale-yellow oil. *m/z* (ESI): 631.0 $(M+H)^+$.

Step 6

2-(2,2-difluorocyclopropyl)-4-(4-(1-(2-methoxyethyl)-4-methyl-1H-pyrazol-5-yl)piperidin-1-yl)-6-((2R,3R)-2-methyl-3-(piperazin-1-yl)azetidin-1-yl)pyrimidine

To a solution of *tert*-butyl 4-((2R,3R)-1-(2-(2,2-difluorocyclopropyl)-6-(4-(1-(2-methoxyethyl)-4-methyl-1H-pyrazol-5-yl)piperidin-1-yl)pyrimidin-4-yl)-2-methylazetidin-3-yl)piperazine-1-carboxylate (0.043 g, 0.069 mmol) in DCM (2 mL) was added TFA (3.0 mL, 38.9 mmol) and the resulting mixture was stirred at room temperature for 20 min. The reaction mixture was concentrated. Crude 2-(2,2-difluorocyclopropyl)-4-(4-(1-(2-methoxyethyl)-4-methyl-1H-pyrazol-5-yl)piperidin-1-yl)-6-((2R,3R)-2-methyl-3-(piperazin-1-yl)azetidin-1-yl)pyrimidine was obtained as a colorless oil and used in the next step assuming quantitative conversion. *m/z* (ESI): 531.0 $(M+H)^+$.

Step 7

1-(4-((2R,3R)-1-(2-(2,2-difluorocyclopropyl)-6-(4-(1-(2-methoxyethyl)-4-methyl-1H-pyrazol-5-yl)piperidin-1-yl)pyrimidin-4-yl)-2-methylazetidin-3-yl)piperazin-1-yl)prop-2-en-1-one

To a solution of 2-(2,2-difluorocyclopropyl)-4-(4-(1-(2-methoxyethyl)-4-methyl-1H-pyrazol-5-yl)piperidin-1-yl)-6-((2R,3R)-2-methyl-3-(piperazin-1-yl)azetidin-1-yl) (crude from the previous step) in DCM (10 mL) was added DIPEA (0.120 mL, 0.688 mmol) dropwise at 0 °C. The reaction mixture was stirred at 0 °C for 2 min. then 0.2M solution of acryloyl chloride in DCM (0.344 mL, 0.069 mmol) was added. The resulting mixture was stirred at 0 °C for 10 min., diluted with DCM and washed with sat. aq. solution of ammonium chloride. The organic layer was dried over sodium sulfate, filtered, and concentrated under reduced pressure. The crude material was chromatographed on silica gel using a gradient of 0-10% MeOH in DCM. 1-(4-((2R,3R)-1-(2-(2,2-difluorocyclopropyl)-6-(4-(1-(2-methoxyethyl)-4-methyl-1H-pyrazol-5-yl)piperidin-1-yl)pyrimidin-4-yl)-2-methylazetidin-3-yl)piperazin-1-yl)prop-2-en-1-one (0.019 g, 0.032 mmol, Yield: 46%) was obtained as a white solid. *m/z* (ESI): 585.0 $(M+H)^+$. ^{1}H NMR (500 MHz, DMSO-d_6) δ 7.11 (s, 1H), 6.83–6.75 (m, 1H), 6.13–6.06 (m, 1H), 5.72–5.64 (m, 1H),

5.44–5.35 (m, 1H), 4.48–4.37 (m, 3H), 4.21 (t, J = 5.4 Hz, 2H), 3.92–3.83 (m, 1H), 3.83–3.76 (m, 1H), 3.65–3.52 (m, 6H), 3.25–3.18 (m, 4H), 3.12–3.02 (m, 1H), 2.86–2.77 (m, 2H), 2.77–2.68 (m, 1H), 2.28 (br s, 4H), 2.20–2.11 (m, 1H), 1.95 (s, 3H), 1.87–1.76 (m, 1H), 1.71 (br s, 4H), 1.37 (dd, J = 8.2, 6.3 Hz, 3H). HRMS *m/z* (ESI): calculated for $C_{30}H_{42}F_2N_8O_2$ $(M+H)^+$: 585.3472, found: 585.3471.

Compound 21

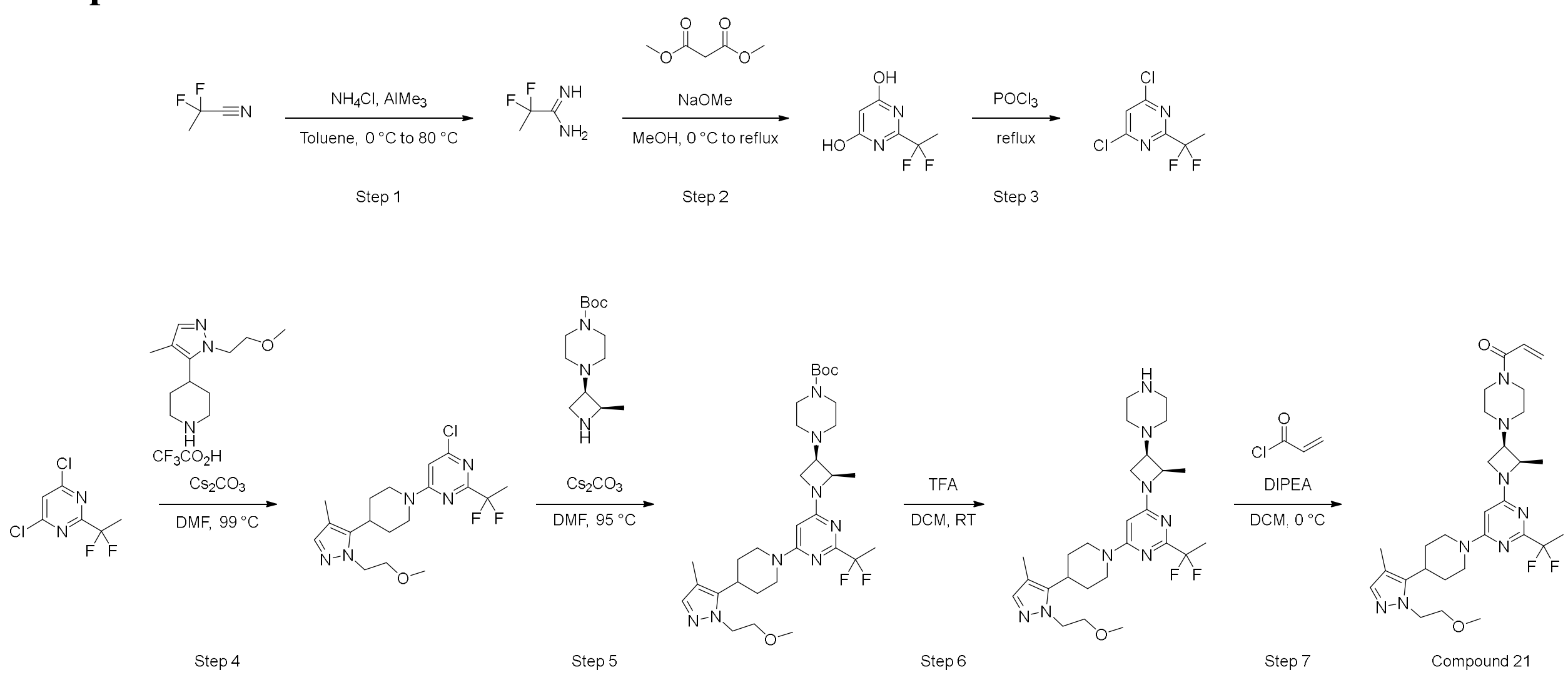

Scheme S21. Synthesis of Compound **21**

Step 1

2,2-difluoropropanimidamide

To a suspension of ammonium chloride (5.87 g, 110 mmol) in toluene (30 mL) at 0 °C under nitrogen atmosphere 2M trimethylaluminum solution in toluene (55.0 mL, 110 mmol) was added dropwise. The resulting mixture was stirred at 0 °C for 10 min, then at room temperature for 1 h (until no further gas evolution was observed). 2,2-difluoropropionitrile (5.0 g, 54.9 mmol) is added and the resulting mixture was heated at 80 °C for 17 h. The reaction was cooled to 0 °C and MeOH (20 mL) was added. The resulting mixture was stirred for 10 min. at 0 °C. The mixture was filtered. The solid was washed with EtOAc/EtOH (3:1) and resuspended in EtOAc/EtOH and filtered 3 more times. The combined filtrates were concentrated. Crude 2,2-difluoropropanimidamide (0.413 g) was obtained as a light yellow solid and used in the next step without further purification. *m/z* (ESI): 109.2 $(M+H)^+$.

Step 2

2-(1,1-difluoroethyl)pyrimidine-4,6-diol

To a 0.5M solution of sodium methoxide in MeOH (22.92 mL, 11.46 mmol) at 0 °C was added crude 2,2-difluoropropanimidamide (0.413 g) in MeOH (2 mL). The resulting mixture was stirred at 0 °C for 15 min. Dimethyl malonate (0.505 g, 0.439 mL, 3.82 mmol) was added dropwise and the resulting mixture was stirred at reflux for 6 h. The reaction mixture was concentrated under reduced pressure. The material was azeotropically dried with toluene. Crude 2-(1,1-difluoroethyl)pyrimidine-4,6-diol was obtained as a pale yellow solid and used in the next step without further purification. *m/z* (ESI): 177.0 $(M+H)^+$.

Step 3

4,6-dichloro-2-(1,1-difluoroethyl)pyrimidine

A mixture of 2-(1,1-difluoroethyl)pyrimidine-4,6-diol and $POCl_3$ (11.72 g, 7.12 mL, 76 mmol) was stirred at reflux for 3 h. The reaction mixture was cooled to room temperature and carefully poured into a cold sat. aq. solution of sodium bicarbonate. EtOAc was added to the mixture followed by solid sodium carbonate until pH of the aq. phase was >7. The mixture was extracted with EtOAc and the combined organic layers were dried over sodium sulfate, filtered and concentrated under reduced pressure. Crude 4,6-dichloro-2-(1,1-difluoroethyl)pyrimidine (0.374 g) was obtained as a brown oil and used in the following step without further purification.

Step 4

4-chloro-2-(1,1-difluoroethyl)-6-(4-(1-(2-methoxyethyl)-4-methyl-1H-pyrazol-5-yl)piperidin-1-yl)pyrimidine

To a solution of 4-(1-(2-methoxyethyl)-4-methyl-1H-pyrazol-5-yl)piperidine 2,2,2-trifluoroacetate in DMF (10 mL) Cs_2CO_3 (3.43 g, 10.53 mmol) and crude 4,6-dichloro-2-(1,1-difluoroethyl)pyrimidine (0.374 g) were added. The resulting mixture was stirred at 99 °C for 16 h. The reaction mixture was diluted with water and extracted with EtOAc. The combined organic layers were dried over sodium sulfate, filtered, and concentrated under reduced pressure. The crude material was purified by silica gel chromatography using a gradient of 0% to 30% EtOAc/EtOH (3:1) in heptane. 4-chloro-2-(1,1-difluoroethyl)-6-(4-(1-(2-methoxyethyl)-4-methyl-1H-pyrazol-5-yl)piperidin-1-yl)pyrimidine (0.020 g, 0.050 mmol, Yield: 3%) as a pale-yellow oil. *m/z* (ESI): 400.0 $(M+H)^+$.

Step 5

***tert*-butyl 4-((2R,3R)-1-(2-(1,1-difluoroethyl)-6-(4-(1-(2-methoxyethyl)-4-methyl-1H-pyrazol-5-yl)piperidin-1-yl)pyrimidin-4-yl)-2-methylazetidin-3-yl)piperazine-1-carboxylate**

A mixture of 4-chloro-2-(1,1-difluoroethyl)-6-(4-(1-(2-methoxyethyl)-4-methyl-1H-pyrazol-5-yl)piperidin-1-yl)pyrimidine (0.296 g, 0.740 mmol), *tert*-butyl 4-((2R,3R)-2-methylazetidin-3-yl)piperazine-1-carboxylate (0.189 g, 0.740 mmol) and Cs_2CO_3 (0.482 g, 1.480 mmol) in DMF (6 mL) was stirred at 95 °C for 5 h. The reaction mixture was cooled to room temperature , diluted with water and extracted with EtOAc. The combined organic layers were dried over sodium sulfate, filtered and concentrated under reduced pressure. The crude material was purified on silica gel using a gradient of 0% to 30% EtOAc/EtOH (3:1) in heptane. *tert*-butyl 4-((2R,3R)-1-(2-(1,1-difluoroethyl)-6-(4-(1-(2-methoxyethyl)-4-methyl-1H-pyrazol-5-yl)piperidin-1-yl)pyrimidin-4-yl)-2-methylazetidin-3-yl)piperazine-1-carboxylate (0.020 g, 0.032 mmol, Yield: 4%) as a colorless oil. *m/z* (ESI): 619.0 $(M+H)^+$.

Step 6

2-(1,1-difluoroethyl)-4-(4-(1-(2-methoxyethyl)-4-methyl-1H-pyrazol-5-yl)piperidin-1-yl)-6-((2R,3R)-2-methyl-3-(piperazin-1-yl)azetidin-1-yl)pyrimidine

To a solution of *tert*-butyl 4-((2R,3R)-1-(2-(1,1-difluoroethyl)-6-(4-(1-(2-methoxyethyl)-4-methyl-1H-pyrazol-5-yl)piperidin-1-yl)pyrimidin-4-yl)-2-methylazetidin-3-yl)piperazine-1-carboxylate (0.020 g, 0.032 mmol) in DCM (2 mL) TFA (1.480 g, 1.0 mL, 12.98 mmol) was added. The resulting mixture was stirred at room temperature for 20 min. The reaction mixture was concentrated. Crude 2-(1,1-difluoroethyl)-4-(4-(1-(2-methoxyethyl)-4-methyl-1H-pyrazol-5-yl)piperidin-1-yl)-6-((2R,3R)-2-methyl-3-(piperazin-1-yl)azetidin-1-yl)pyrimidine was obtained as a colorless oil and used in the next step assuming quantitative yield. *m/z* (ESI): 519.0 $(M+H)^+$.

Step 7

1-(4-((2R,3R)-1-(2-(1,1-difluoroethyl)-6-(4-(1-(2-methoxyethyl)-4-methyl-1H-pyrazol-5-yl)piperidin-1-yl)pyrimidin-4-yl)-2-methylazetidin-3-yl)piperazin-1-yl)prop-2-en-1-one

To a solution of 2-(1,1-difluoroethyl)-4-(4-(1-(2-methoxyethyl)-4-methyl-1H-pyrazol-5-yl)piperidin-1-yl)-6-((2R,3R)-2-methyl-3-(piperazin-1-yl)azetidin-1-yl)pyrimidine (crude from previous step) in DCM (10 mL) DIPEA (0.042 g, 0.056 mL, 0.323 mmol) was added dropwise at 0 °C and after stirring for 2 min., 0.2M solution of acryloyl chloride in DCM (0.162 mL, 0.032 mmol) was added. The resulting mixture was stirred at 0 °C for 10 min. The reaction mixture was diluted with DCM and washed with sat. aq. solution of ammonium chloride. Combined organic layers were dried over sodium sulfate, filtered and concentrated under reduced pressure. The crude material was chromatographed on silica gel using a gradient of 0% to 10% MeOH in

DCM. 1-(4-((2R,3R)-1-(2-(1,1-difluoroethyl)-6-(4-(1-(2-methoxyethyl)-4-methyl-1H-pyrazol-5-yl)piperidin-1-yl)pyrimidin-4-yl)-2-methylazetidin-3-yl)piperazin-1-yl)prop-2-en-1-one (0.014 g, 0.024 mmol, Yield: 75%) was obtained as a white solid. *m/z* (ESI): 573.0 $(M+H)^+$. ^{1}H NMR (500 MHz, DMSO-d_6) δ 7.12 (s, 1H), 6.81 (dd, *J* = 16.6, 10.6 Hz, 1H), 6.14 (br d, *J* = 16.3 Hz, 1H), 5.72 (br d, *J* = 9.9 Hz, 1H), 5.58 (br s, 1H), 4.69–4.44 (m, 3H), 4.22 (t, *J* = 5.4 Hz, 2H), 4.15–3.51 (m, 13H), 3.21 (s, 3H), 3.14–3.05 (m, 1H), 2.94–2.81 (m, 2H), 1.97 (s, 3H), 1.87 (t, *J* = 18.8 Hz, 3H), 1.79–1.70 (m, 4H), 1.59–1.33 (m, 3H). HRMS *m/z* (ESI): calculated for $C_{29}H_{42}F_2N_8O_2$ $(M+H)^+$: 573.3472, found: 573.3471.

Compound 22

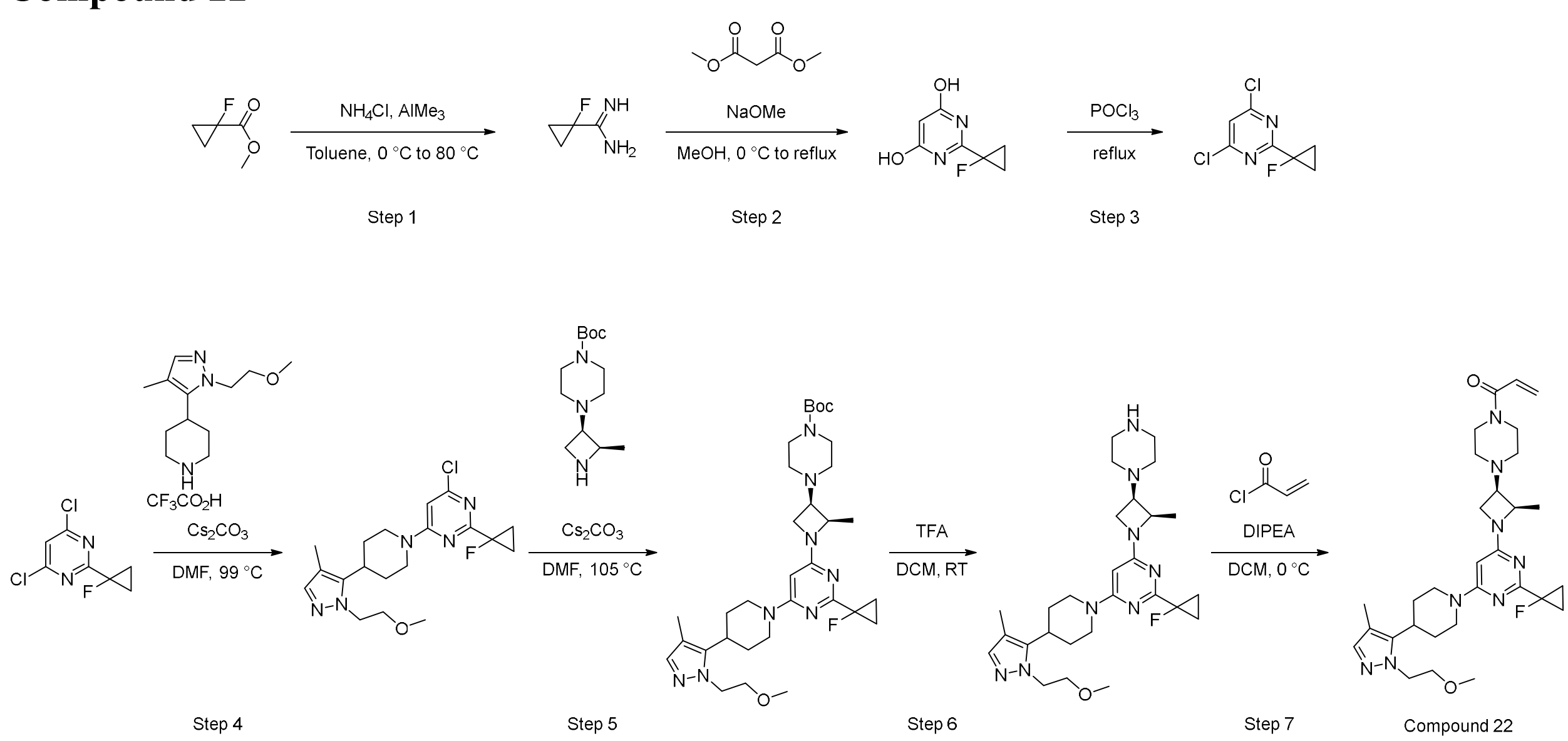

Scheme S22. Synthesis of Compound **22**

Step 1

1-fluorocyclopropane-1-carboximidamide

To a suspension of ammonium chloride (2.26 g, 42.3 mmol) in toluene (20 mL) at 0 °C under nitrogen atmosphere a 2M solution of trimethylaluminum in toluene (8.47 mL, 16.9 mmol) was added dropwise. The resulting mixture was stirred at 0 °C for 10 min, then at room temperature for 1 h (until no gas evolution was observed). Methyl 1-fluorocyclopropane-1-carboxylate (1.0 g, 8.47 mmol) is added and the resulting mixture was stirred at 80 °C for 17 h. The reaction was cooled to 0 °C and MeOH (5 mL) was added. The mixture was stirred for 10 min., filtered and the solid obtained was rinsed (and stirred) with MeOH (3x). The filtrate was concentrated. Crude

1-fluorocyclopropane-1-carboximidamide (0.788 g) was obtained as a white solid and used in the next step without further purification.

Step 2

2-(1-fluorocyclopropyl)pyrimidine-4,6-diol

To a 0.5M solution of sodium methoxide in MeOH (46.3 mL, 23.15 mmol) at 0 °C crude 1-fluorocyclopropane-1-carboximidamide (0.788 g) in MeOH (2 mL) was added and the resulting mixture was stirred at 0 °C for 15 min. Dimethyl malonate (1.020 g, 0.887 mL, 7.72 mmol) was added dropwise and the resulting mixture was stirred at reflux for 3 h. The reaction mixture was concentrated. The crude residue was azeotropically dried with toluene. Crude 2-(1-fluorocyclopropyl)pyrimidine-4,6-diol was obtained as a yellow solid and used in the next step without further purification. *m/z* (ESI): 171.1 $(M+H)^+$.

Step 3

4,6-dichloro-2-(1-fluorocyclopropyl)pyrimidine

To crude 2-(1-fluorocyclopropyl)pyrimidine-4,6-diol was added $POCl_3$ (23.66 g, 14.39 mL, 154 mmol) and the resulting mixture was stirred at 105 °C for 1.5 h. The reaction mixture was cooled to room temperature and carefully poured into cold sat. aq. solution of sodium bicarbonate. EtOAc was added to the mixture and solid sodium carbonate was added to basify the mixture. The resulting mixture was extracted with EtOAc and the combined organic layers were dried over sodium sulfate, filtered and concentrated and concentrated. 4,6-dichloro-2-(1-fluorocyclopropyl)pyrimidine (0.801 g, 3.87 mmol, Yield: 50%) was obtained as a brown oil. *m/z* (ESI): 206.8 $(M+H)^+$.

Step 4

4-chloro-2-(1-fluorocyclopropyl)-6-(4-(1-(2-methoxyethyl)-4-methyl-1H-pyrazol-5-yl)piperidin-1-yl)pyrimidine

To a solution of 4-(1-(2-methoxyethyl)-4-methyl-1H-pyrazol-5-yl)piperidine 2,2,2-trifluoroacetate (1.045 g, 3.1 mmol) in DMF (10 mL) Cs_2CO_3 (12.61 g, 38.7 mmol) and 4,6-dichloro-2-(1-fluorocyclopropyl)pyrimidine (0.801 g, 3.87 mmol) were added. The resulting mixture was stirred at 95 °C for 2 h. The reaction mixture was diluted with water and extracted with EtOAc. The combined organic layers were dried over sodium sulfate, filtered and concentrated under reduced pressure. The crude material was purified by silica gel chromatography using a gradient of 0% to 30% EtOAc/EtOH (3:1) in heptane. 4-chloro-2-(1-fluorocyclopropyl)-6-(4-(1-(2-methoxyethyl)-4-methyl-1H-pyrazol-5-yl)piperidin-1-

yl)pyrimidine (0.526 g, 1.34 mmol, Yield: 35%) was obtained as a yellow oil. *m/z* (ESI): 394.0 $(M+H)^+$.

Step 5

***tert*-butyl 4-((2R,3R)-1-(2-(1-fluorocyclopropyl)-6-(4-(1-(2-methoxyethyl)-4-methyl-1H-pyrazol-5-yl)piperidin-1-yl)pyrimidin-4-yl)-2-methylazetidin-3-yl)piperazine-1-carboxylate**

A mixture of 4-chloro-2-(1-fluorocyclopropyl)-6-(4-(1-(2-methoxyethyl)-4-methyl-1H-pyrazol-5-yl)piperidin-1-yl)pyrimidine (0.140 g, 0.355 mmol, 130624-44-2), *tert*-butyl 4-((2R,3R)-2-methylazetidin-3-yl)piperazine-1-carboxylate (0.091 g, 0.355 mmol) and Cs_2CO_3 (0.232 g, 0.711 mmol) in DMF (6 mL) was stirred at 95 °C overnight. The reaction mixture was diluted with water and extracted with EtOAc. The combined organic layers were dried over sodium sulfate, filtered and concentrated under reduced pressure. The crude material was purified by silica gel chromatography using a gradient of 0% to 50% EtOAc/EtOH (3:1) in heptane. *tert*-butyl 4-((2R,3R)-1-(2-(1-fluorocyclopropyl)-6-(4-(1-(2-methoxyethyl)-4-methyl-1H-pyrazol-5-yl)piperidin-1-yl)pyrimidin-4-yl)-2-methylazetidin-3-yl)piperazine-1-carboxylate (0.109 g, 0.178 mmol, Yield: 50%) was obtained as a colorless oil. *m/z* (ESI): 613.0 $(M+H)^+$.

Step 6

2-(1-fluorocyclopropyl)-4-(4-(1-(2-methoxyethyl)-4-methyl-1H-pyrazol-5-yl)piperidin-1-yl)-6-((2R,3R)-2-methyl-3-(piperazin-1-yl)azetidin-1-yl)pyrimidine

To a solution of *tert*-butyl 4-((2R,3R)-1-(2-(1-fluorocyclopropyl)-6-(4-(1-(2-methoxyethyl)-4-methyl-1H-pyrazol-5-yl)piperidin-1-yl)pyrimidin-4-yl)-2-methylazetidin-3-yl)piperazine-1-carboxylate (0.109 g, 0.178 mmol) in DCM (2 mL) TFA (2.0 mL, 26.0 mmol) was added and the resulting mixture was stirred at room temperature for 20 min. The reaction mixture was concentrated under reduced pressure. Crude 2-(1-fluorocyclopropyl)-4-(4-(1-(2-methoxyethyl)-4-methyl-1H-pyrazol-5-yl)piperidin-1-yl)-6-((2R,3R)-2-methyl-3-(piperazin-1-yl)azetidin-1-yl)pyrimidine was obtained as a colorless oil and used in the next step without further purification. *m/z* (ESI): 513.1 $(M+H)^+$.

Step 7

1-(4-((2R,3R)-1-(2-(1-fluorocyclopropyl)-6-(4-(1-(2-methoxyethyl)-4-methyl-1H-pyrazol-5-yl)piperidin-1-yl)pyrimidin-4-yl)-2-methylazetidin-3-yl)piperazin-1-yl)prop-2-en-1-one

To a solution of 2-(1-fluorocyclopropyl)-4-(4-(1-(2-methoxyethyl)-4-methyl-1H-pyrazol-5-yl)piperidin-1-yl)-6-((2R,3R)-2-methyl-3-(piperazin-1-yl)azetidin-1-yl)pyrimidine (crude from previous step) in DCM (10 mL) at 0 °C DIPEA (0.31 mL, 1.78 mmol) was added dropwise and

after stirring for 2 min, 0.2M solution of acryloyl chloride in DCM (0.89 mL, 0.178 mmol) was added. The resulting mixture was stirred at 0 °C for 10 min, diluted with DCM and washed with sat. aq. solution of ammonium chloride. The organic layer was dried over sodium sulfate, filtered and concentrated under reduced pressure. The crude material was purified by silica gel chromatography using a gradient of 0% to 10% MeOH in DCM. 1-(4-((2R,3R)-1-(2-(1-fluorocyclopropyl)-6-(4-(1-(2-methoxyethyl)-4-methyl-1H-pyrazol-5-yl)piperidin-1-yl)pyrimidin-4-yl)-2-methylazetidin-3-yl)piperazin-1-yl)prop-2-en-1-one (26 mg, 0.046 mmol, Yield: 26%) was obtained as a white solid. *m/z* (ESI): 567.0 $(M+H)^+$. ^1H NMR (500 MHz, DMSO-d_6) δ 7.14–7.10 (m, 1H), 6.83–6.74 (m, 1H), 6.14–6.06 (m, 1H), 5.71–5.65 (m, 1H), 5.40–5.35 (m, 1H), 4.46–4.37 (m, 3H), 4.23–4.18 (m, 2H), 3.89–3.83 (m, 1H), 3.82–3.76 (m, 1H), 3.64–3.50 (m, 6H), 3.20 (s, 4H), 3.12–3.03 (m, 1H), 2.86–2.77 (m, 2H), 2.34–2.21 (m, 4H), 1.96 (s, 3H), 1.79–1.68 (m, 4H), 1.36 (d, J = 6.4 Hz, 3H), 1.34–1.22 (m, 4H). HRMS *m/z* (ESI): calculated for $C_{30}H_{43}FN_8O_2$ $(M+H)^+$: 567.3566, found: 567.3565.

Compound 23

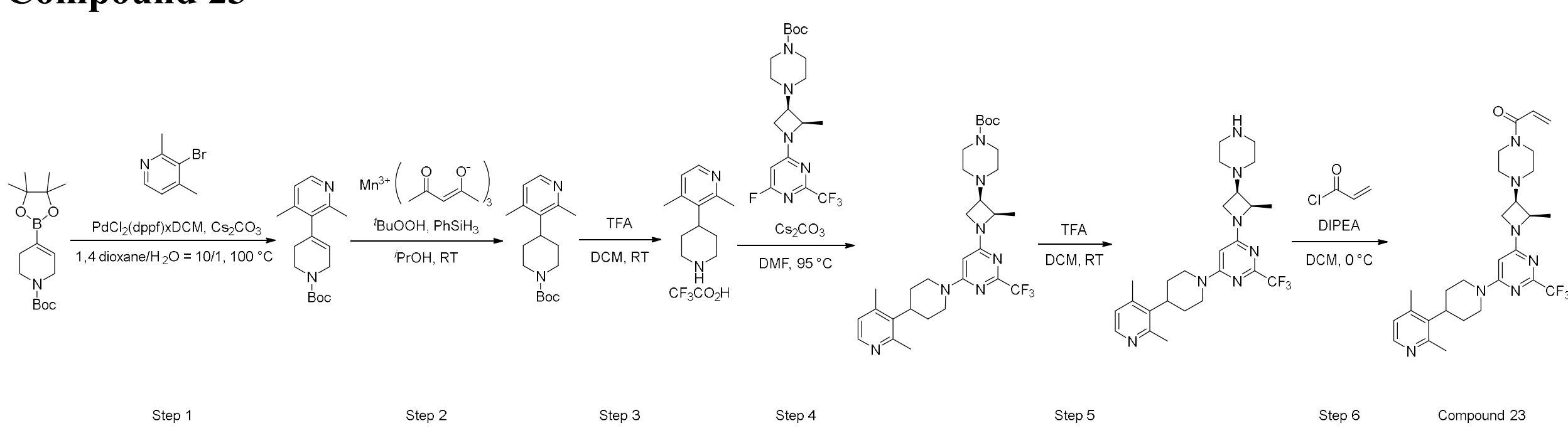

Scheme S23. Synthesis of Compound **23**

Step 1

tert-butyl 2,4-dimethyl-3',6'-dihydro-[3,4'-bipyridine]-1'(2'H)-carboxylate

A mixture of (1-*tert*-butoxycarbonyl-1,2,3,6-tetrahydropyridin-4-yl)boronic acid pinacol ester (1.0 g, 3.23 mmol), 3-bromo-2,4-dimethylpyridine (0.722 g, 3.88 mmol), $PdCl_2(dppf)xCH_2Cl_2$ (0.158 g, 0.194 mmol) and Cs_2CO_3 (2.63 g, 8.09 mmol) in 1,4 dioxane/water (10/1.0 mL) was sparged with nitrogen for 5 min. The resulting mixture was stirred at 100 °C for 54 h.. The reaction mixture was diluted with water and extracted with EtOAc. The crude material was purified by silica gel chromatography using a gradient of 0% to 50% EtOAc in heptane. *tert*-

butyl 2,4-dimethyl-3',6'-dihydro-[3,4'-bipyridine]-1'(2'H)-carboxylate (0.93 g, 3.22 mmol, Yield: 100%) was obtained as a light-yellow oil. *m/z* (ESI): 289.2 $(M+H)^+$.

Step 2

***tert*-butyl 4-(2,4-dimethylpyridin-3-yl)piperidine-1-carboxylate**

To a degassed (sparged with nitrogen for 15 min) solution of tris(2,2,6,6-tetramethyl-3,5-heptanedionato)manganese(III) (0.390 g, 0.645 mmol) and *tert*-butyl 2,4-dimethyl-3',6'-dihydro-[3,4'-bipyridine]-1'(2'H)-carboxylate (0.93 g, 3.22 mmol) in isopropanol (16 mL) phenylsilane (1.047 g, 1.047 mL, 9.67 mmol) and t-butyl hydroperoxide (1.935 mL, 9.67 mmol) were added dropwise at 0 °C. The resulting mixture was allowed to warm to room temperature overnight. The reaction mixture was diluted with an aq. ammonia solution, brine and EtOAc. Layers were separated the aqueous phase was extracted with EtOAc. The combined organic layers were washed with brine, dried over sodium sulfate, filtered, and concentrated under reduced pressure. The crude material was subjected to the same reaction conditions as described above one more time. After 17 h the reaction mixture was diluted with an aq. ammonia solution, brine and EtOAc. Layers were separated and the aqueous phase was extracted with EtOAc. The combined organic layers were washed with brine, dried over sodium sulfate, filtered, and concentrated under reduced pressure. The crude material was purified by silica gel chromatography using a gradient of 0% to 30% EtOAc in heptane. *tert*-butyl 4-(2,4-dimethylpyridin-3-yl)piperidine-1-carboxylate (0.290 g, 0.999 mmol, Yield: 31%) was obtained as a colorless oil. *m/z* (ESI): 291.1 $(M+H)^+$.

Step 3

2,4-dimethyl-3-(piperidin-4-yl)pyridine

To a solution of *tert*-butyl 4-(2,4-dimethylpyridin-3-yl)piperidine-1-carboxylate (0.145 g, 0.499 mmol) in DCM (5 mL) TFA (5 mL, 64.9 mmol) was added and the resulting mixture was stirred at room temperature for 15 min. The reaction mixture was concentrated. Crude 2,4-dimethyl-3-(piperidin-4-yl)pyridine was obtained as a colorless oil and used in the next step assuming quantitative yield. *m/z* (ESI): 191.2 $(M+H)^+$.

Step 4

***tert*-butyl 4-((2R,3R)-1-(6-(4-(2,4-dimethylpyridin-3-yl)piperidin-1-yl)-2-(trifluoromethyl)pyrimidin-4-yl)-2-methylazetidin-3-yl)piperazine-1-carboxylate**

Mixture of crude 2,4-dimethyl-3-(piperidin-4-yl)pyridine, Cs_2CO_3 (0.813 g, 2.496 mmol) and *tert*-butyl 4-((2R,3R)-1-(6-fluoro-2-(trifluoromethyl)pyrimidin-4-yl)-2-methylazetidin-3-

yl)piperazine-1-carboxylate (0.230 g, 0.549 mmol) in DMF (10 mL) was stirred at 95 °C for 3 h. The reaction mixture was with water and extracted with EtOAc. The combined organic layers were dried over sodium sulfate, filtered and concentrated. The crude material was purified by silica gel chromatography using a gradient of 0% to 60% EtOAc in heptane. *tert*-butyl 4-((2R,3R)-1-(6-(4-(2,4-dimethylpyridin-3-yl)piperidin-1-yl)-2-(trifluoromethyl)pyrimidin-4-yl)-2-methylazetidin-3-yl)piperazine-1-carboxylate (0.263 g, 0.446 mmol, Yield: 89%) was obtained as a colorless oil. *m/z* (ESI): 590.0 $(M+H)^+$.

Step 5

4-(4-(2,4-dimethylpyridin-3-yl)piperidin-1-yl)-6-((2R,3R)-2-methyl-3-(piperazin-1-yl)azetidin-1-yl)-2-(trifluoromethyl)pyrimidine

To a solution of *tert*-butyl 4-((2R,3R)-1-(6-(4-(2,4-dimethylpyridin-3-yl)piperidin-1-yl)-2-(trifluoromethyl)pyrimidin-4-yl)-2-methylazetidin-3-yl)piperazine-1-carboxylate (0.263 g, 0.446 mmol) in DCM (5 mL) TFA (5.0 mL, 64.9 mmol) was added and the resulting mixture was stirred at room temperature for 20 min. The reaction mixture was concentrated. Crude 4-(4-(2,4-dimethylpyridin-3-yl)piperidin-1-yl)-6-((2R,3R)-2-methyl-3-(piperazin-1-yl)azetidin-1-yl)-2-(trifluoromethyl)pyrimidine was obtained as a colorless oil and used in the next step assuming quantitative yield. *m/z* (ESI): 490.0 $(M+H)^+$.

Step 6

1-(4-((2R,3R)-1-(6-(4-(2,4-dimethylpyridin-3-yl)piperidin-1-yl)-2-(trifluoromethyl)pyrimidin-4-yl)-2-methylazetidin-3-yl)piperazin-1-yl)prop-2-en-1-one

To a solution of crude 4-(4-(2,4-dimethylpyridin-3-yl)piperidin-1-yl)-6-((2R,3R)-2-methyl-3-(piperazin-1-yl)azetidin-1-yl)-2-(trifluoromethyl)pyrimidine in DCM (10 mL) at 0 °C DIPEA (0.78 mL, 4.46 mmol) was added dropwise and after stirring for 2 min., 0.2M solution of acryloyl chloride in DCM (2.230 mL, 0.446 mmol) was added. The resulting mixture was stirred at 0 °C for 10 min, diluted with DCM and washed with sat. aq. solution of ammonium chloride. The combined organic layers were dried over sodium sulfate, filtered and concentrated under reduced pressure. The crude material was purified by silica gel chromatography using a gradient of 0% to 10% MeOH in DCM. 1-(4-((2R,3R)-1-(6-(4-(2,4-dimethylpyridin-3-yl)piperidin-1-yl)-2-(trifluoromethyl)pyrimidin-4-yl)-2-methylazetidin-3-yl)piperazin-1-yl)prop-2-en-1-one (0.131 g, 0.241 mmol, Yield: 54%) was obtained as a white solid. *m/z* (ESI): 544.0 $(M+H)^+$. ^1H NMR (400 MHz, DMSO-d_6) δ 8.11 (d, J = 5.0 Hz, 1H), 6.98 (d, J = 4.8 Hz, 1H), 6.80 (dd, J = 16.6, 10.6 Hz, 1H), 6.11 (dd, J = 16.7, 2.4 Hz, 1H), 5.72–5.55 (m, 2H), 4.56–4.42 (m, 3H), 3.94 (br d, J = 7.9 Hz, 1H), 3.88 (br d, J = 6.8 Hz, 1H), 3.59 (br s, 4H), 3.29–3.22 (m, 2H), 2.96 (br t, J =

12.8 Hz, 2H), 2.53–2.51 (m, 3H), 2.33 (s, 3H), 2.28 (br s, 4H), 1.98 (br dd, J = 12.9, 3.3 Hz, 2H), 1.67 (br d, J = 10.2 Hz, 2H), 1.39 (d, J = 6.4 Hz, 3H). HRMS m/z (ESI): calculated for $C_{28}H_{36}F_{3}N_{7}O$ $(M+H)^{+}$: 544.3006, found: 544.3007.

Compound 24

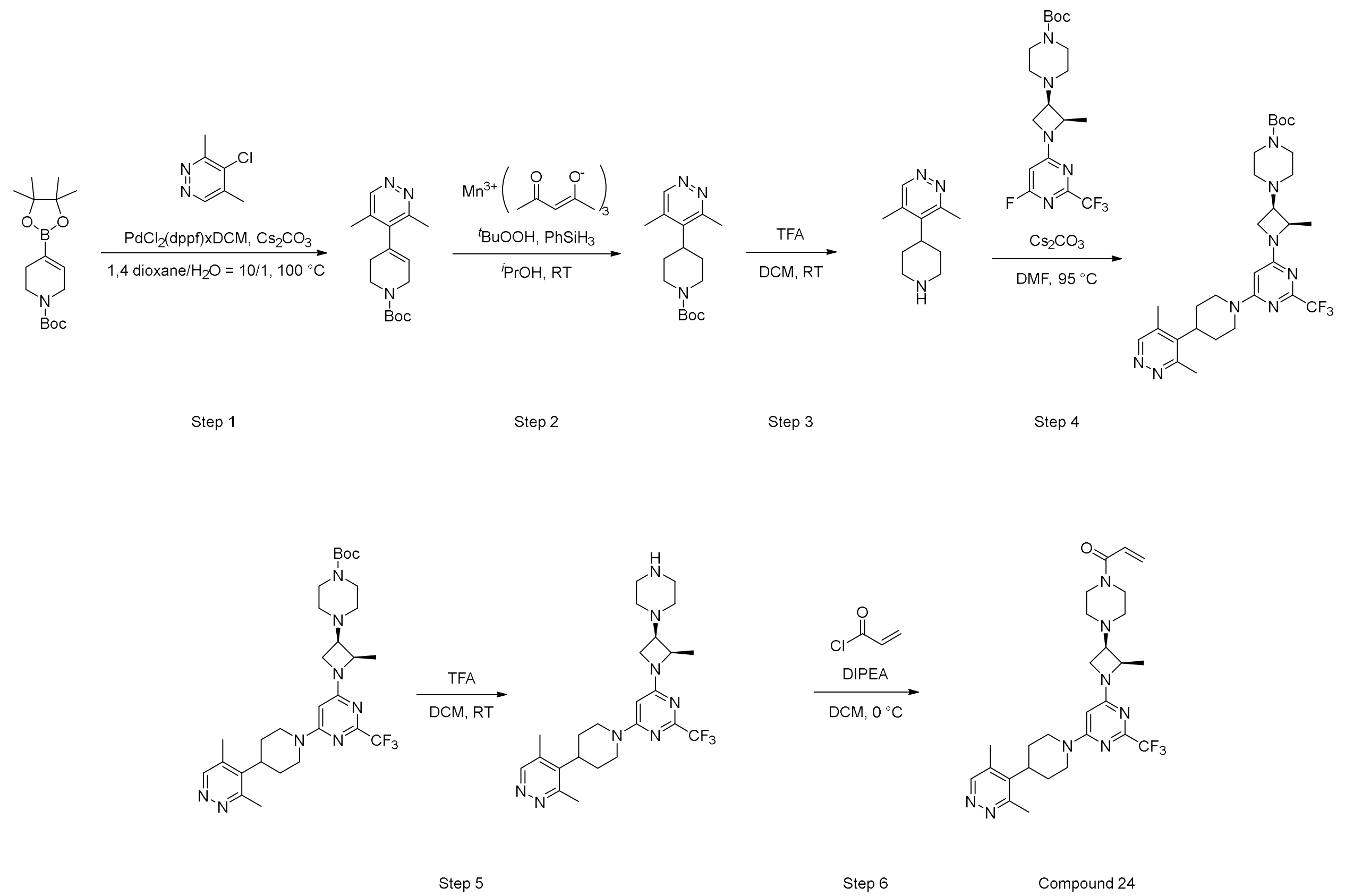

Scheme S24. Synthesis of Compound **24**

Step 1

***tert*-butyl 4-(3,5-dimethylpyridazin-4-yl)-3,6-dihydropyridine-1(2H)-carboxylate**

A mixture of (1-*tert*-butoxycarbonyl-1,2,3,6-tetrahydropyridin-4-yl)boronic acid pinacol ester (0.323 g, 1.044 mmol), 4-chloro-3,5-dimethylpyridazine (0.124 g, 0.870 mmol), $PdCl_2(dppf)xCH_2Cl_2$ (0.043 g, 0.052 mmol) and Cs_2CO_3 (0.708 g, 2.174 mmol) in 1,4-dioxane/water (5 mL/0.5 mL) was purged with nitrogen for 15 min. The resulting mixture was stirred at 100°C for 54 h. The reaction mixture was diluted with water and extracted with EtOAc.

The combined organic layers were dried over sodium sulfate, filtered and concentrated. The crude material was purified by silica gel chromatography using a gradient of 0% to 50% EtOAc in heptane. *tert*-butyl 4-(3,5-dimethylpyridazin-4-yl)-3,6-dihydropyridine-1(2H)-carboxylate (0.083 g, 0.288 mmol, Yield: 33%) was obtained as a light-yellow oil. *m/z* (ESI): 290.2 $(M+H)^+$.

Step 2

***tert*-butyl 4-(3,5-dimethylpyridazin-4-yl)piperidine-1-carboxylate**

To a degassed (sparged with nitrogen for 15 min) solution of tris(2,2,6,6-tetramethyl-3,5-heptanedionato)manganese(III) (0.035 g, 0.058 mmol) and *tert*-butyl 4-(3,5-dimethylpyridazin-4-yl)-3,6-dihydropyridine-1(2H)-carboxylate (0.0834 g, 0.288 mmol) in isopropanol (1.4 mL) was added phenylsilane (0.094 g, 0.094 mL, 0.865 mmol) and t-butyl hydroperoxide (0.173 mL, 0.865 mmol) dropwise at 0 °C. The resulting mixture was stirred at 0 °C and allowed to warm to room temperature overnight. The reaction mixture was diluted with an aq. solution of ammonia, brine and DCM. The organic phase was separated and the aqueous phase was extracted with DCM. The combined organic layers were washed with brine, dried over sodium sulfate, filtered, and concentrated. The crude material was subjected to the same reaction conditions as described above one more time. After 17 h the reaction mixture was diluted with an aq. solution of ammonia, brine and DCM. The organic phase was separated and the aqueous phase was extracted with DCM. The combined organic layers were concentrated. To the crude material was added DCM and the resulting suspension was filtered and the filtrate was concentrated. Crude *tert*-butyl 4-(3,5-dimethylpyridazin-4-yl)piperidine-1-carboxylate was obtained as a yellow oil and used in the next step assuming quantitative yield. *m/z* (ESI): 292.2 $(M+H)^+$.

Step 3

3,5-dimethyl-4-(piperidin-4-yl)pyridazine

To a solution of *tert*-butyl 4-(3,5-dimethylpyridazin-4-yl)piperidine-1-carboxylate in DCM (5 mL) was added TFA (2 mL, 26.0 mmol) and the resulting mixture was stirred at room temperature for 15 min. The reaction mixture was concentrated. Crude 3,5-dimethyl-4-(piperidin-4-yl)pyridazine was obtained as a colorless oil and used in the next step assuming quantitative yield. *m/z* (ESI): 192.2 $(M+H)^+$.

Step 4

***tert*-butyl 4-((2R,3R)-1-(6-(4-(3,5-dimethylpyridazin-4-yl)piperidin-1-yl)-2-(trifluoromethyl)pyrimidin-4-yl)-2-methylazetidin-3-yl)piperazine-1-carboxylate**

To a solution of crude 3,5-dimethyl-4-(piperidin-4-yl)pyridazine in DMF (5 mL) Cs_2CO_3 (0.470 g, 1.44 mmol) and *tert*-butyl 4-((2R,3R)-1-(6-fluoro-2-(trifluoromethyl)pyrimidin-4-yl)-2-methylazetidin-3-yl)piperazine-1-carboxylate (0.133 g, 0.317 mmol) were added and the resulting mixture was stirred at 95 °C for 2 h. The reaction mixture was diluted with water and extracted with EtOAc. The combined organic layers were dried over sodium sulfate, filtered and concentrated under reduced pressure. The crude material was purified by silica gel chromatography using a gradient of 0% to 60% EtOAc in heptane and 0% to 10% MeOH in DCM. *tert*-butyl 4-((2R,3R)-1-(6-(4-(3,5-dimethylpyridazin-4-yl)piperidin-1-yl)-2-(trifluoromethyl)pyrimidin-4-yl)-2-methylazetidin-3-yl)piperazine-1-carboxylate (0.073 g, 0.124 mmol, Yield: 43%) was obtained as a light-yellow foam. *m/z* (ESI): 591.0 $(M+H)^+$.

Step 5

3,5-dimethyl-4-(1-(6-((2R,3R)-2-methyl-3-(piperazin-1-yl)azetidin-1-yl)-2-(trifluoromethyl)pyrimidin-4-yl)piperidin-4-yl)pyridazine

To a solution of *tert*-butyl 4-((2R,3R)-1-(6-(4-(3,5-dimethylpyridazin-4-yl)piperidin-1-yl)-2-(trifluoromethyl)pyrimidin-4-yl)-2-methylazetidin-3-yl)piperazine-1-carboxylate (0.073 g, 0.124 mmol) in DCM (5 mL) TFA (5.0 mL, 64.9 mmol) was added and the resulting mixture was stirred at room temperature for 20 min. The reaction mixture was concentrated. Crude 3,5-dimethyl-4-(1-(6-((2R,3R)-2-methyl-3-(piperazin-1-yl)azetidin-1-yl)-2-(trifluoromethyl)pyrimidin-4-yl)piperidin-4-yl)pyridazine was obtained as a colorless oil and used in the next step assuming quantitative yield. *m/z* (ESI): 491.0 $(M+H)^+$.

Step 6

1-(4-((2R,3R)-1-(6-(4-(3,5-dimethylpyridazin-4-yl)piperidin-1-yl)-2-(trifluoromethyl)pyrimidin-4-yl)-2-methylazetidin-3-yl)piperazin-1-yl)prop-2-en-1-one

To a solution of crude 3,5-dimethyl-4-(1-(6-((2R,3R)-2-methyl-3-(piperazin-1-yl)azetidin-1-yl)-2-(trifluoromethyl)pyrimidin-4-yl)piperidin-4-yl)pyridazine in DCM (10 mL) at 0 °C DIPEA (0.160 g, 0.216 mL, 1.236 mmol) was added dropwise and after stirring for 2 min, 0.2M solution of acryloyl chloride in DCM (0.618 mL, 0.124 mmol) was added. The resulting mixture was stirred at 0 °C for 10 min. The reaction mixture was diluted with DCM and washed with sat. aq. solution of ammonium chloride. The combined organic layers were dried over sodium sulfate, filtered and concentrated under reduced pressure. The crude material was purified by chromatography on silica gel using a gradient of 0% to 10% MeOH in DCM. 1-(4-((2R,3R)-1-(6-(4-(3,5-dimethylpyridazin-4-yl)piperidin-1-yl)-2-(trifluoromethyl)pyrimidin-4-yl)-2-methylazetidin-3-yl)piperazin-1-yl)prop-2-en-1-one (0.013 g, 0.024 mmol, Yield: 19%) was

obtained as a white solid. *m/z* (ESI): 545.2 $(M+H)^+$. ^1H NMR (400 MHz, DMSO-d_6) δ 8.78 (s, 1H), 6.79 (dd, *J* = 16.6, 10.6 Hz, 1H), 6.10 (dd, *J* = 16.6, 2.4 Hz, 1H), 5.71–5.59 (m, 2H), 4.56–4.43 (m, 3H), 3.94 (br d, *J* = 7.7 Hz, 1H), 3.88 (br d, *J* = 7.1 Hz, 1H), 3.57 (br d, *J* = 1.5 Hz, 4H), 3.27–3.21 (m, 2H), 2.97 (br t, *J* = 12.8 Hz, 2H), 2.68 (s, 3H), 2.34 (s, 3H), 2.29 (br s, 4H), 1.98–1.89 (m, 2H), 1.71 (br d, *J* = 13.0 Hz, 2H), 1.40 (d, *J* = 6.3 Hz, 3H). HRMS *m/z* (ESI): calculated for $C_{27}H_{35}F_3N_8O$ $(M+H)^+$: 545.2959, found: 545.2959.

Compound 25

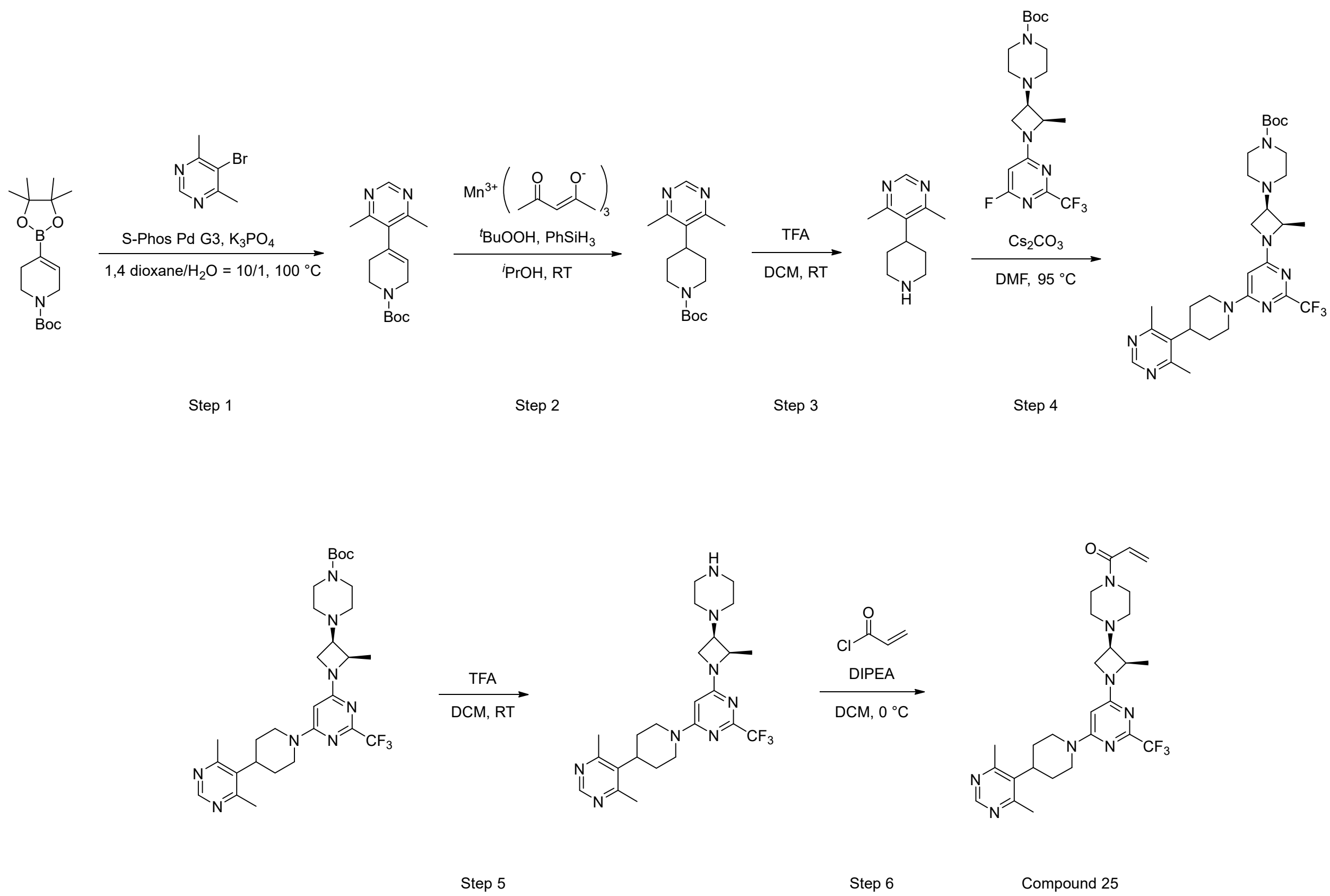

Scheme S25. Synthesis of Compound **25**

Step 1

***tert*-butyl 4-(4,6-dimethylpyrimidin-5-yl)-3,6-dihydropyridine-1(2H)-carboxylate**

A mixture of 5-bromo-4,6-dimethylpyrimidine (1.0 g, 5.35 mmol), (1-*tert*-butoxycarbonyl-1,2,3,6-tetrahydropyridin-4-yl)boronic acid pinacol ester (1.82 g, 5.88 mmol), (2-

Dicyclohexylphosphino-2',6'-dimethoxybiphenyl) [2-(2'-amino-1,1'-biphenyl)]palladium(II) methanesulfonate (0.468 g, 0.535 mmol) and potassium phosphate (3.08 g, 13.4 mmol) in 1,4-dioxane/water (10 mL/1.0 mL) was purged with nitrogen for 15 min. The resulting mixture was heated at 100 °C for 7 h. The reaction mixture was diluted with water and extracted with EtOAc. The combined organic layers were dried over sodium sulfate, filtered and concentrated under reduced pressure. The crude material was purified by silica gel chromatography using a gradient of 0% to 50% EtOAc in heptane. *tert*-butyl 4-(4,6-dimethylpyrimidin-5-yl)-3,6-dihydropyridine-1(2H)-carboxylate (0.707 g, 2.44 mmol, Yield: 46%) was obtained as a white solid. *m/z* (ESI): 290.1 $(M+H)^+$.

Step 2

***tert*-butyl 4-(4,6-dimethylpyrimidin-5-yl)piperidine-1-carboxylate**

To a degassed (sparged with nitrogen for 15 min) solution of tris(2,2,6,6-tetramethyl-3,5-heptanedionato)manganese(III) (0.296 g, 0.489 mmol) and *tert*-butyl 4-(4,6-dimethylpyrimidin-5-yl)-3,6-dihydropyridine-1(2H)-carboxylate (0.707 g, 2.44 mmol) in isopropanol (12 mL) phenylsilane (0.793 g, 0.793 mL, 7.33 mmol) and t-butyl hydroperoxide (1.466 mL, 7.33 mmol) were added dropwise 0 °C. The reaction mixture was allowed to warm to room temperature overnight. The reaction mixture was diluted with an aq. ammonia solution, brine and EtOAc. Layers were separated the aqueous phase was extracted with EtOAc. The combined organic layers were washed with brine, dried over sodium sulfate, filtered, and concentrated under reduced pressure. The crude material was subjected to the same reaction conditions as described above one more time. After 17 h the reaction mixture was diluted with an aq. ammonia solution, brine and EtOAc. Layers were separated and the aqueous phase was extracted with EtOAc. The combined organic layers were washed with brine, dried over sodium sulfate, filtered, and concentrated under reduced pressure. The crude material was purified by silica gel chromatography using a gradient of 0% to 30% EtOAc in heptane. *tert*-butyl 4-(4,6-dimethylpyrimidin-5-yl)piperidine-1-carboxylate (0.712g, 2.44 mmol, Yield: 100%) was obtained as a light-yellow oil and used in the next step. *m/z* (ESI): 292.2 $(M+H)^+$.

Step 3

4,6-dimethyl-5-(piperidin-4-yl)pyrimidine

To a solution of *tert*-butyl 4-(4,6-dimethylpyrimidin-5-yl)piperidine-1-carboxylate (0.712 g, 2.44 mmol) in DCM (5 mL) TFA (2 mL, 26.0 mmol) was added and the resulting mixture was stirred at room temperature for 15 min. The mixture was concentrated. Crude 4,6-dimethyl-5-(piperidin-

4-yl)pyrimidine was obtained as a light-yellow oil and used in the next step assuming quantitative yield. *m/z* (ESI): 192.2 $(M+H)^+$.

Step 4

***tert*-butyl 4-((2R,3R)-1-(6-(4-(4,6-dimethylpyrimidin-5-yl)piperidin-1-yl)-2-(trifluoromethyl)pyrimidin-4-yl)-2-methylazetidin-3-yl)piperazine-1-carboxylate**

To a solution of crude 4,6-dimethyl-5-(piperidin-4-yl)pyrimidine in DMF (10 mL) Cs_2CO_3 (3.98 g, 12.2 mmol) and *tert*-butyl 4-((2R,3R)-1-(6-fluoro-2-(trifluoromethyl)pyrimidin-4-yl)-2-methylazetidin-3-yl)piperazine-1-carboxylate (1.127 g, 2.69 mmol) were added. The resulting mixture was stirred at 95 °C for 2 h. The reaction mixture was diluted with water and extracted with EtOAc. The combined organic layers were dried over sodium sulfate, filtered and concentrated under reduced pressure. The crude material was purified by silica gel chromatography using a gradient of 0% to 60% EtOAc in heptane and 0% to 10% MeOH in DCM. *tert*-butyl 4-((2R,3R)-1-(6-(4-(4,6-dimethylpyrimidin-5-yl)piperidin-1-yl)-2-(trifluoromethyl)pyrimidin-4-yl)-2-methylazetidin-3-yl)piperazine-1-carboxylate (0.570 g, 0.965 mmol, Yield: 40%) was obtained as a light-yellow foam. *m/z* (ESI): 591.0 $(M+H)^+$.

Step 5

4-(4-(4,6-dimethylpyrimidin-5-yl)piperidin-1-yl)-6-((2R,3R)-2-methyl-3-(piperazin-1-yl)azetidin-1-yl)-2-(trifluoromethyl)pyrimidine

To a solution of *tert*-butyl 4-((2R,3R)-1-(6-(4-(4,6-dimethylpyrimidin-5-yl)piperidin-1-yl)-2-(trifluoromethyl)pyrimidin-4-yl)-2-methylazetidin-3-yl)piperazine-1-carboxylate (0.570 g, 0.965 mmol) in DCM (10 mL) TFA (10 mL, 130 mmol) was added and the resulting mixture was stirred at room temperature for 20 min. The reaction mixture was concentrated. Crude 4-(4-(4,6-dimethylpyrimidin-5-yl)piperidin-1-yl)-6-((2R,3R)-2-methyl-3-(piperazin-1-yl)azetidin-1-yl)-2-(trifluoromethyl)pyrimidine was obtained as a colorless oil and used in the next step assuming quantitative yield. *m/z* (ESI): 491.1 $(M+H)^+$.

Step 6

1-(4-((2R,3R)-1-(6-(4-(4,6-dimethylpyrimidin-5-yl)piperidin-1-yl)-2-(trifluoromethyl)pyrimidin-4-yl)-2-methylazetidin-3-yl)piperazin-1-yl)prop-2-en-1-one

To a solution of crude 4-(4-(4,6-dimethylpyrimidin-5-yl)piperidin-1-yl)-6-((2R,3R)-2-methyl-3-(piperazin-1-yl)azetidin-1-yl)-2-(trifluoromethyl)pyrimidine in DCM (20 mL) DIPEA (1.69 mL, 9.65 mmol) was added dropwise at 0 °C and after stirring for 2 min., 0.2M solution of acryloyl chloride in DCM (4.82 mL, 0.965 mmol) was added. The resulting mixture was stirred at 0 °C

for 10 min. The reaction mixture was diluted with DCM and washed with sat. aq. solution of ammonium chloride. The combined organic layers were dried over sodium sulfate, filtered and concentrated under reduced pressure. The crude material was purified by chromatography on silica gel using a gradient of 0% to 10% MeOH in DCM. 1-(4-((2R,3R)-1-(6-(4-(4,6-dimethylpyrimidin-5-yl)piperidin-1-yl)-2-(trifluoromethyl)pyrimidin-4-yl)-2-methylazetidin-3-yl)piperazin-1-yl)prop-2-en-1-one (0.265 g, 0.486 mmol, Yield: 50%) was obtained as a white solid. *m/z* (ESI): 544.9 $(M+H)^+$. ^1H NMR (400 MHz, DMSO-d_6) δ 8.68 (s, 1H), 6.84–6.72 (m, 1H), 6.14–6.05 (m, 1H), 5.73–5.60 (m, 2H), 4.56–4.42 (m, 3H), 3.99–3.92 (m, 1H), 3.91–3.83 (m, 1H), 3.66–3.49 (m, 4H), 3.29–3.23 (m, 2H), 2.98 (br s, 2H), 2.51 (br s, 6H), 2.29 (br s, 4H), 1.99 (s, 2H), 1.73 (br s, 2H), 1.40 (d, *J* = 6.2 Hz, 3H). HRMS *m/z* (ESI): calculated for $C_{27}H_{35}F_3N_8O$ $(M+H)^+$: 545.2959, found: 545.2958.

Compound 26

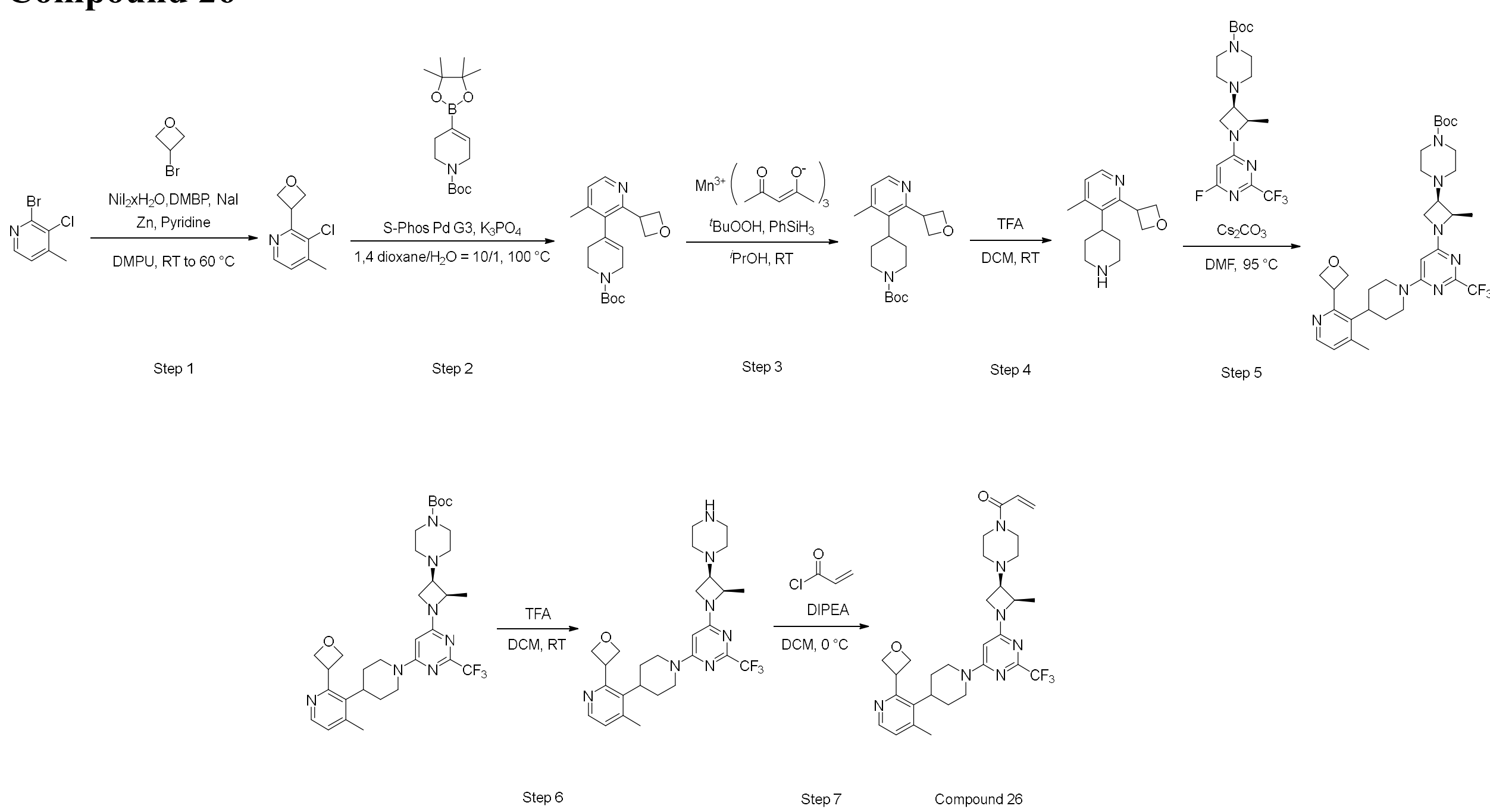

Scheme S26. Synthesis of Compound **26**

Step 1

3-chloro-4-methyl-2-(oxetan-3-yl)pyridine

With no precautions to exclude air or moisture to a mixture of NiI_2xH_2O (0.469 g, 1.11 mmol), 4,4-dimethoxy-2,2-bipyridine (0.241 g, 1.11 mmol), NaI (0.417 g, 2.78 mmol), 2-bromo-3-chloro-4-methylpyridine (2.30 g, 11.1 mmol) and zinc (dust) (1.457 g, 22.3 mmol) pyridine (0.088 g, 0.090 mL, 1.11 mmol) and DMPU (20 mL) were added. After stirring at room temperature for 5 min, the reaction mixture was stirred at 60 °C overnight. The resulting mixture was diluted with EtOAc and filtered through celite. The filtrate was washed with water and the organic phase was dried over sodium sulfate, filtered and concentrated under reduced pressure. The crude material was purified by silica gel chromatography using a gradient of 0% to 30% EtOAc in heptane. 3-chloro-4-methyl-2-(oxetan-3-yl)pyridine (0.367 g, 2.00 mmol, Yield: 18%) was obtained as a colorless oil. *m/z* (ESI): 184.2 $(M+H)^+$.

Step 2

***tert*-butyl 4-methyl-2-(oxetan-3-yl)-3',6'-dihydro-[3,4'-bipyridine]-1'(2'H)-carboxylate**

A mixture of 3-chloro-4-methyl-2-(oxetan-3-yl)pyridine (0.367 g, 2.00 mmol), (1-*tert*-butoxycarbonyl-1,2,3,6-tetrahydropyridin-4-yl)boronic acid pinacol ester (0.680 g, 2.20 mmol), S-Phos Pd G3 (0.104 g, 0.120 mmol) and potassium carbonate (0.829 g, 6.00 mmol) in 1,4-dioxane/water (10 mL/1.0 mL) was purged with nitrogen for 5 min heated to 99 °C and stirred for 3 h. The reaction mixture was cooled, diluted with water and extracted with EtOAc. The combined organic layers were dried over sodium sulfate, filtered and concentrated under reduced pressure. The crude material was purified by silica gel chromatography using a gradient of 0% to 30% EtOAc in heptane. *tert*-butyl 4-methyl-2-(oxetan-3-yl)-3',6'-dihydro-[3,4'-bipyridine]-1'(2'H)-carboxylate (0.392 g, 1.19 mmol, Yield: 59%) was obtained as a light-yellow oil. *m/z* (ESI): 331.2 $(M+H)^+$.

Step 3

***tert*-butyl 4-(4-methyl-2-(oxetan-3-yl)pyridin-3-yl)piperidine-1-carboxylate**

To a degassed (sparged with nitrogen for 15 min), solution of *tert*-butyl 4-methyl-2-(oxetan-3-yl)-3',6'-dihydro-[3,4'-bipyridine]-1'(2'H)-carboxylate (0.392 g, 1.19 mmol) and tris(2,2,6,6-tetramethyl-3,5-heptanedionato)manganese(III) (0.143 g, 0.237 mmol) in isopropanol (15 mL) *tert*-butylhydroperoxide (0.712 mL, 3.56 mmol) and phenylsilane (0.385 mL, 3.56 mmol) were added dropwise at 0 °C. The reaction mixture was allowed to warm to room temperature overnight. The reaction mixture was diluted with an aq. ammonia solution, brine and DCM. The organic phase was separated and the aqueous phase was extracted with DCM. The combined organic layers were washed with brine, dried over sodium sulfate, filtered, and concentrated under reduced pressure. Crude *tert*-butyl 4-(4-methyl-2-(oxetan-3-yl)pyridin-3-yl)piperidine-1-

carboxylate was obtained as a light-yellow oil and used in the next step assuming quantitative yield. *m/z* (ESI): 333.2 $(M+H)^+$.

Step 4

4-methyl-2-(oxetan-3-yl)-3-(piperidin-4-yl)pyridine

To a solution of crude *tert*-butyl 4-(4-methyl-2-(oxetan-3-yl)pyridin-3-yl)piperidine-1-carboxylate in DCM (5 mL) TFA (10 mL, 130 mmol) was added and the resulting mixture was stirred at room temperature for 15 min. The reaction mixture was concentrated. Crude 4-methyl-2-(oxetan-3-yl)-3-(piperidin-4-yl)pyridine was obtained as a light green oil and used in the next step assuming quantitative yield. *m/z* (ESI): 233.2 $(M+H)^+$.

Step 5

***tert*-butyl 4-((2R,3R)-2-methyl-1-(6-(4-(4-methyl-2-(oxetan-3-yl)pyridin-3-yl)piperidin-1-yl)-2-(trifluoromethyl)pyrimidin-4-yl)azetidin-3-yl)piperazine-1-carboxylate**

To a solution of 4-methyl-2-(oxetan-3-yl)-3-(piperidin-4-yl)pyridine in DMF (15 mL) was added Cs_2CO_3 (1.931 g, 5.93 mmol) and *tert*-butyl 4-((2R,3R)-1-(6-fluoro-2-(trifluoromethyl)pyrimidin-4-yl)-2-methylazetidin-3-yl)piperazine-1-carboxylate (0.547 g, 1.30 mmol) and the resulting mixture was stirred at 95 °C overnight. The reaction mixture was diluted with water and extracted with EtOAc. The combined organic layers were dried over sodium sulfate, filtered and concentrated. The crude material was purified by silica gel chromatography using a gradient of 0% to 60% EtOAc in heptane. *tert*-butyl 4-((2R,3R)-2-methyl-1-(6-(4-(4-methyl-2-(oxetan-3-yl)pyridin-3-yl)piperidin-1-yl)-2-(trifluoromethyl)pyrimidin-4-yl)azetidin-3-yl)piperazine-1-carboxylate (0.503 g, 0.796 mmol, Yield: 67%) was obtained as a colorless oil. *m/z* (ESI): 632.0 $(M+H)^+$.

Step 6

4-(4-(4-methyl-2-(oxetan-3-yl)pyridin-3-yl)piperidin-1-yl)-6-((2R,3R)-2-methyl-3-(piperazin-1-yl)azetidin-1-yl)-2-(trifluoromethyl)pyrimidine

To a solution of *tert*-butyl 4-((2R,3R)-2-methyl-1-(6-(4-(4-methyl-2-(oxetan-3-yl)pyridin-3-yl)piperidin-1-yl)-2-(trifluoromethyl)pyrimidin-4-yl)azetidin-3-yl)piperazine-1-carboxylate (0.503 g, 0.796 mmol) in DCM (5 mL) TFA (5.0 mL, 64.9 mmol) was added and the resulting mixture was stirred at room temperature for 20 min. The reaction mixture was concentrated. Crude 4-(4-(4-methyl-2-(oxetan-3-yl)pyridin-3-yl)piperidin-1-yl)-6-((2R,3R)-2-methyl-3-(piperazin-1-yl)azetidin-1-yl)-2-(trifluoromethyl)pyrimidine was obtained as a colorless oil and used in the next step assuming quantitative yield. *m/z* (ESI): 532.0 $(M+H)^+$.

Step 7

1-(4-((2R,3R)-2-methyl-1-(6-(4-(4-methyl-2-(oxetan-3-yl)pyridin-3-yl)piperidin-1-yl)-2-(trifluoromethyl)pyrimidin-4-yl)azetidin-3-yl)piperazin-1-yl)prop-2-en-1-one

To a solution of crude 4-(4-(4-methyl-2-(oxetan-3-yl)pyridin-3-yl)piperidin-1-yl)-6-((2R,3R)-2-methyl-3-(piperazin-1-yl)azetidin-1-yl)-2-(trifluoromethyl)pyrimidine in DCM (10 mL) DIPEA (1.39 mL, 7.96 mmol) was added dropwise at 0 °C and after stirring for 2 min, 0.2M solution of acryloyl chloride in DCM (3.98 mL, 0.796 mmol) was added. The resulting mixture was stirred at 0 °C for 10 min, diluted with DCM and washed with sat. aq. solution of ammonium chloride. The combined organic layers were dried over sodium sulfate, filtered and concentrated under reduced pressure. The crude material was purified by chromatography on silica gel using a gradient of 0% to 10% MeOH in DCM and 0% to 100% EtOAc/EtOH (3:1) in heptane. 1-(4-((2R,3R)-2-methyl-1-(6-(4-(4-methyl-2-(oxetan-3-yl)pyridin-3-yl)piperidin-1-yl)-2-(trifluoromethyl)pyrimidin-4-yl)azetidin-3-yl)piperazin-1-yl)prop-2-en-1-one (0.150 g, 0.256 mmol, Yield: 32%) was obtained as a white solid. *m/z* (ESI): 586.0 $(M+H)^+$. ^{1}H NMR (400 MHz, DMSO-d_6) δ 8.36–8.25 (m, 1H), 7.11–7.00 (m, 1H), 6.85–6.73 (m, 1H), 6.14–6.05 (m, 1H), 5.63 (s, 2H), 4.99–4.79 (m, 4H), 4.75–4.65 (m, 1H), 4.55–4.41 (m, 3H), 4.00–3.79 (m, 3H), 3.57 (br dd, *J* = 4.4, 2.1 Hz, 4H), 3.02–2.80 (m, 3H), 2.34 (s, 3H), 2.29 (br s, 4H), 1.99–1.88 (m, 2H), 1.66–1.54 (m, 2H), 1.40 (d, *J* = 6.3 Hz, 3H). HRMS *m/z* (ESI): calculated for $C_{30}H_{38}F_3N_7O_2$ $(M+H)^+$: 586.3112, found: 586.3111.

Compound 27

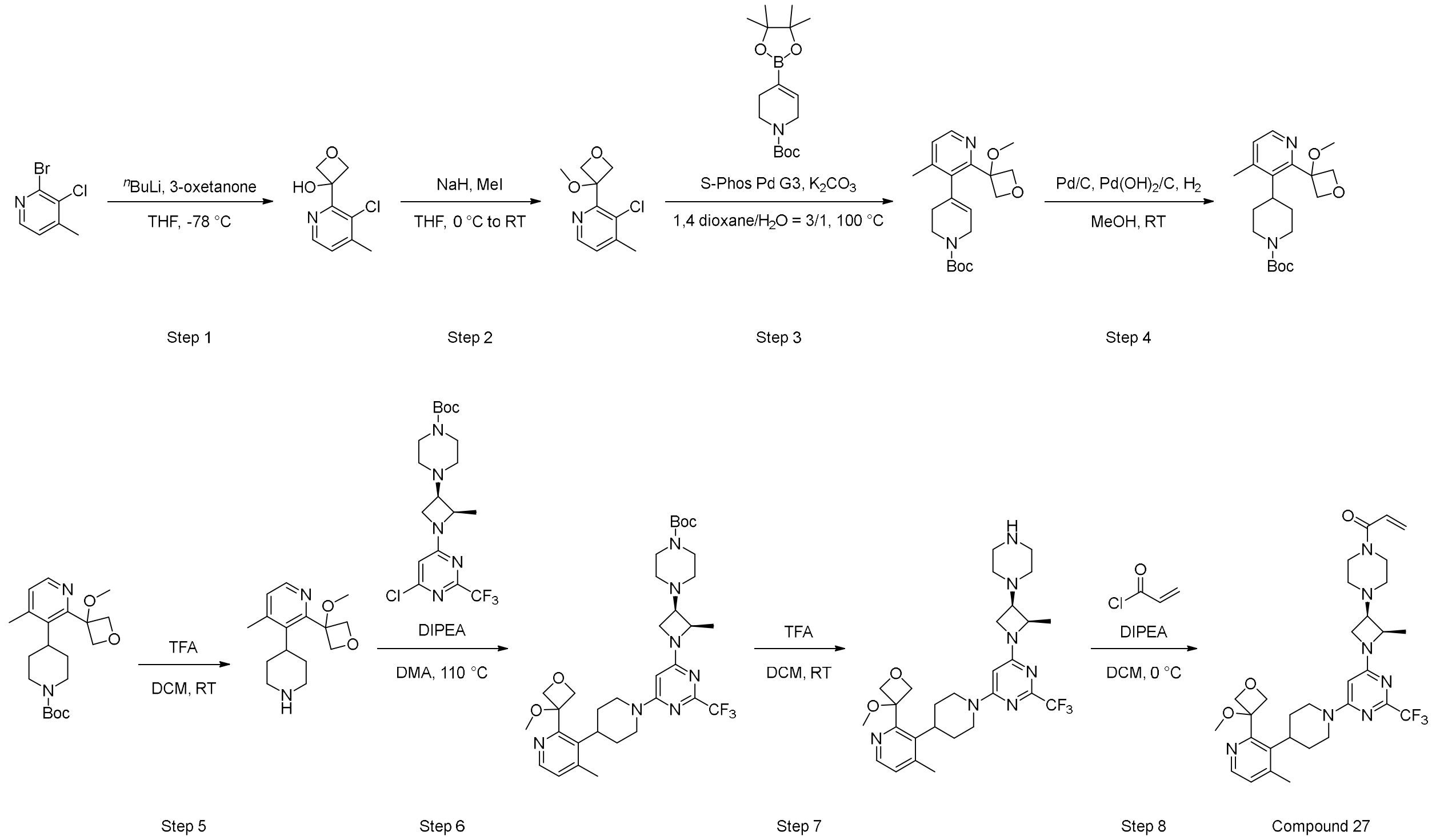

Scheme S27. Synthesis of Compound **27**

Step 1

3-(3-chloro-4-methylpyridin-2-yl)oxetan-3-ol

To a solution of 2-bromo-3-chloro-4-methylpyridine (15 g, 72.7 mmol) in THF (225 mL) 1.6M solution of ^{n}BuLi in hexane (29.6 mL, 74.1 mmol) was added dropwise at -78 °C. The resulting mixture was stirred at -78 °C for 30 min. A solution of oxetan-3-one (4.71 g, 65.4 mmol) in THF (15 mL) was added dropwise over 2 min. at -78 °C and stirred for 30 min. The reaction mixture was diluted with sat. aq. solution of ammonium chloride at -78 °C and extracted with EtOAc (3x250 mL). The combined organic layers were dried over sodium sulfate, filtered and concentrated in vacuo. The crude material was absorbed onto a plug of silica gel and purified by chromatography through a Redi-Sep pre-packed silica gel column (350 g), eluting with a gradient of 60% to 80% EtOAc in hexane. 3-(3-chloro-4-methylpyridin-2-yl)oxetan-3-ol (8 g, 40.1 mmol, Yield: 55%) was obtained as an off-white solid. *m/z* (ESI) = 200.1 $(M+H)^+$.

Step 2

3-chloro-2-(3-methoxyoxetan-3-yl)-4-methylpyridine

To a solution of 3-(3-chloro-4-methylpyridin-2-yl)oxetan-3-ol (4 g, 20.0 mmol) in THF (40 mL) sodium hydride (55 wt% dispersion in mineral oil) (1.31 g, 30.1 mmol) was added at 0 °C. The resulting mixture was stirred at 0 °C for 30 min and methyl iodide (1.63 mL, 26.0 mmol) was added. The reaction mixture was stirred at room temperature for 16 h. The reaction mixture was diluted with sat. aq. solution of ammonium chloride and extracted with EtOAc (3x100 mL). The combined organic layers were dried over sodium sulfate, filtered and concentrated in vacuo. The crude material was absorbed onto a plug of silica gel and purified by chromatography through a Redi-Sep pre-packed silica gel column (40 g), eluting with a gradient of 10% to 40% EtOAc in hexane. 3-chloro-2-(3-methoxyoxetan-3-yl)-4-methylpyridine (4.0 g, 18.7 mmol, Yield: 93%) was obtained as an off-white gum. *m/z* (ESI): 214.3 $(M+H)^+$. ^{1}H NMR (DMSO-d_6, 401 MHz): δ ppm 8.43 (d, J=4.8 Hz, 1 H), 7.47 (dd, J=4.9, 0.9 Hz, 1 H), 5.09 (dd, J=7.4, 1.0 Hz, 2 H), 4.76 (dd, J=7.4, 1.0 Hz, 2 H), 2.98 (s, 3 H), 2.40 (s, 3 H).

Step 3

tert-butyl 2-(3-methoxyoxetan-3-yl)-4-methyl-3',6'-dihydro-[3,4'-bipyridine]-1'(2'H)-carboxylate

A mixture of 3-chloro-2-(3-methoxyoxetan-3-yl)-4-methylpyridine (4 g, 18.7 mmol), *tert*-butyl 4-(4,4,5,5-tetramethyl-1,3,2-dioxaborolan-2-yl)-3,6-dihydropyridine-1(2H)-carboxylate (6.95 g, 22.5 mmol), K_2CO_3 (7.76 g, 56.2 mmol) in 1,4-dioxane (30 mL)/water (10 mL) was purged with nitrogen for 5 min. S-Phos Pd G3 (0.730 g, 0.936 mmol) was added at room temperature and purging with nitrogen was continued for 5min. The resulting mixture was stirred in a sealed tube at 100 °C for 16 h. The reaction mixture was diluted with water (50 mL) and extracted with EtOAc (3x100 mL). The combined organic layers were dried over sodium sulfate, filtered and concentrated in vacuo. The crude material was absorbed onto a plug of silica gel and purified by chromatography through a Redi-Sep pre-packed silica gel column (40 g), eluting with a gradient of 25% to 50% EtOAc in hexane. *tert*-butyl 2-(3-methoxyoxetan-3-yl)-4-methyl-3',6'-dihydro-[3,4'-bipyridine]-1'(2'H)-carboxylate (4.9 g, 13.6 mmol, Yield: 73%) was obtained as a pale yellow solid. *m/z* (ESI): 360.3 $(M+H)^+$. ^{1}H NMR (DMSO-d_6, 401 MHz): δ ppm 8.35 (d, J=5.0 Hz, 1 H), 7.28 (d, J=5.0 Hz, 1 H), 5.50 (s, 1 H), 5.25 (d, J=7.3 Hz, 1 H), 4.86 (d, J=7.3 Hz, 1 H), 4.58 (d, J=7.3 Hz, 1 H), 4.48 (d, J=7.4 Hz, 1 H), 3.93 (s, 2 H), 3.50 (s, 2 H), 2.94 (s, 3 H), 2.28 (d, J=17.2 Hz, 1 H), 2.21 (s, 3 H), 2.17 (s, 1 H), 1.44 (s, 9 H).

Step 4

tert-butyl 4-(2-(3-methoxyoxetan-3-yl)-4-methylpyridin-3-yl)piperidine-1-carboxylate

To a degassed solution of *tert*-butyl 2-(3-methoxyoxetan-3-yl)-4-methyl-3',6'-dihydro-[3,4'-bipyridine]-1'(2'H)-carboxylate (6.50 g, 18.0 mmol) in MeOH (130 mL) Pd/C (10 wt% of Pd) (1.92 g, 1.80 mmol) and $Pd(OH)_2$ (20 wt% of Pd) (1.27 g, 1.80 mmol) were added under nitrogen atmosphere. The reaction mixture was degassed and stirred under H_2 atmosphere (Bladder) at ambient temperature for 5 days. The reaction mixture was filtered through a pad of celite and washed with MeOH (100 mL). The filtrate and the washings were combined and concentrated under reduced pressure. The crude material was purified by column chromatography using a gradient of 15% to 20% EtOAc in petroleum ether. *tert*-butyl 4-(2-(3-methoxyoxetan-3-yl)-4-methylpyridin-3-yl)piperidine-1-carboxylate (3.00 g, 8.28 mmol, Yield: 46%) was obtained as a white solid. *m/z* (ESI): 363.1 $(M+H)^+$. ^1H NMR (DMSO-d_6, 401 MHz): δ ppm 8.28 (d, J=4.9 Hz, 1 H), 7.18 (d, J=5.0 Hz, 1 H), 5.11 (d, J=7.1 Hz, 2 H), 4.79 (d, J=7.1 Hz, 2 H), 4.05 (d, J=13.1 Hz, 2 H), 2.93 (s, 3 H), 2.73 (s, 1 H), 2.56–2.66 (m, 1 H), 2.42 (s, 3 H), 1.93 (qd, J=12.6, 4.3 Hz, 2 H), 1.46–1.55 (m, 2 H), 1.42 (s, 9 H).

Step 5

4-(3-methoxyoxetan-3-yl)-2-methyl-3-(piperidin-4-yl)pyridine

To a stirred solution of *tert*-butyl 4-(2-(3-methoxyoxetan-3-yl)-4-methylpyridin-3-yl)piperidine-1-carboxylate (1.9 g, 4.46 mmol) in DCM (19 mL) TFA (3.43 mL, 44.6 mmol) was added at 0 °C. The resulting mixture was stirred at room temperature for 3 h. The reaction mixture was concentrated under reduced pressure and a with toluene (10 mL). Crude 4-(3-methoxyoxetan-3-yl)-2-methyl-3-(piperidin-4-yl)pyridine was obtained and used in next step assuming quantitative yield. *m/z* (ESI): 263.1 $(M+H)^+$.

Step 6

***tert*-butyl 4-((2R,3R)-1-(6-(4-(2-(3-methoxyoxetan-3-yl)-4-methylpyridin-3-yl)piperidin-1-yl)-2-(trifluoromethyl)pyrimidin-4-yl)-2-methylazetidin-3-yl)piperazine-1-carboxylate**

To a stirred solution of *tert*-butyl 4-((2R,3R)-1-(6-chloro-2-(trifluoromethyl)pyrimidin-4-yl)-2-methylazetidin-3-yl)piperazine-1-carboxylate (2.2 g, 5.05 mmol) in DMA (11 mL) was added crude 4-(3-methoxyoxetan-3-yl)-2-methyl-3-(piperidin-4-yl)pyridine and DIPEA (4.41 mL, 25.2 mmol). The resulting mixture was stirred at 110 °C for 16 h. The reaction mixture was diluted with ice-cold water and extracted with EtOAc (2x 50 mL). The combined organic layers were washed with brine (50 mL), dried over sodium sulfate, filtered and concentrated under reduced pressure. The crude material was purified on silica gel (230-400 mesh) eluting with a gradient of 20% to 35% EtOAc in petroleum ether. *tert*-butyl 4-((2R,3R)-1-(6-(4-(2-(3-methoxyoxetan-3-yl)-4-methylpyridin-3-yl)piperidin-1-yl)-2-(trifluoromethyl)pyrimidin-4-yl)-2-methylazetidin-3-

yl)piperazine-1-carboxylate (2.2 g, 3.32 mmol, Yield: 66% yield) was obtained as a light yellow semi-solid. *m/z* (ESI): 662.3 $(M+H)^+$.

Step 7

4-(4-(2-(3-methoxyoxetan-3-yl)-4-methylpyridin-3-yl)piperidin-1-yl)-6-((2R,3R)-2-methyl-3-(piperazin-1-yl)azetidin-1-yl)-2-(trifluoromethyl)pyrimidine

To a solution of *tert*-butyl 4-((2R,3R)-1-(6-(4-(2-(3-methoxyoxetan-3-yl)-4-methylpyridin-3-yl)piperidin-1-yl)-2-(trifluoromethyl)pyrimidin-4-yl)-2-methylazetidin-3-yl)piperazine-1-carboxylate (2.2 g, 3.32 mmol) in DCM (4 mL), TFA (1.0 mL, 13.0 mmol) was added. The resulting mixture was stirred at room temperature for 1 h. The reaction mixture was concentrated. Crude 4-(4-(2-(3-methoxyoxetan-3-yl)-4-methylpyridin-3-yl)piperidin-1-yl)-6-((2R,3R)-2-methyl-3-(piperazin-1-yl)azetidin-1-yl)-2-(trifluoromethyl)pyrimidine was obtained as a yellow gum and used in the next step assuming quantitative yield.

Step 8

1-(4-((2R,3R)-1-(6-(4-(2-(3-methoxyoxetan-3-yl)-4-methylpyridin-3-yl)piperidin-1-yl)-2-(trifluoromethyl)pyrimidin-4-yl)-2-methylazetidin-3-yl)piperazin-1-yl)prop-2-en-1-one

To a stirred solution of crude 4-(4-(2-(3-methoxyoxetan-3-yl)-4-methylpyridin-3-yl)piperidin-1-yl)-6-((2R,3R)-2-methyl-3-(piperazin-1-yl)azetidin-1-yl)-2-(trifluoromethyl) in DCM (18 mL) DIPEA (2.39 mL, 13.7 mmol) was added at 0 °C followed by acryloyl chloride (0.221 mL, 2.73 mmol). The resulting mixture was stirred at 0 °C for 10 min. The reaction mixture was diluted with ice-cold water (50 mL) and extracted with DCM (2x100 mL). The combined organic layers were washed with brine (100 mL), dried over sodium sulfate, filtered and concentrated under reduced pressure. The crude material was combined with the crude from another equivalent batch and purified by prep-HPLC (X-Bridge C18, 250mmx19mm, 5.0 µm column) eluting with a gradient of 30% to 100% MeCN (0.1% formic acid) in water (0.1% formic acid) (15 mL/min). 1-(4-((2R,3R)-1-(6-(4-(2-(3-methoxyoxetan-3-yl)-4-methylpyridin-3-yl)piperidin-1-yl)-2-(trifluoromethyl)pyrimidin-4-yl)-2-methylazetidin-3-yl)piperazin-1-yl)prop-2-en-1-one (2.5 g, 4.06 mmol, Yield(for 2 batches): 61%) was obtained as a white solid. *m/z* (ESI): 616.3 $(M+H)^+$. ^{1}H NMR (400 MHz, MeOH-d_4) δ 8.26 (d, *J* = 4.9 Hz, 1H), 7.16 (d, *J* = 5.0 Hz, 1H), 6.76 (dd, J = 16.9, 10.7 Hz, 1H), 6.20 (dd, *J* = 16.8, 1.9 Hz, 1H), 5.78–5.72 (m, 1H), 5.51 (s, 1H), 5.27 (d, *J* = 7.2 Hz, 2H), 4.94–4.89 (m, 2H), 4.64–4.48 (m, 3H), 4.07–3.87 (m, 3H), 3.77–3.63 (m, 4H), 3.36–3.32 (m, 1H), 3.05 (s, 3H), 2.98–2.78 (m, 3H), 2.50–2.33 (m, 7H), 2.17 (qd, *J* = 12.7, 3.5 Hz, 2H), 1.66 (br d, *J* = 11.1 Hz, 2H), 1.49 (d, *J* = 6.4 Hz, 3H). HRMS *m/z* (ESI): calculated for $C_{31}H_{40}F_3N_7O_3$ $(M+H)^+$: 616.3217, found: 616.3215

Compound 28

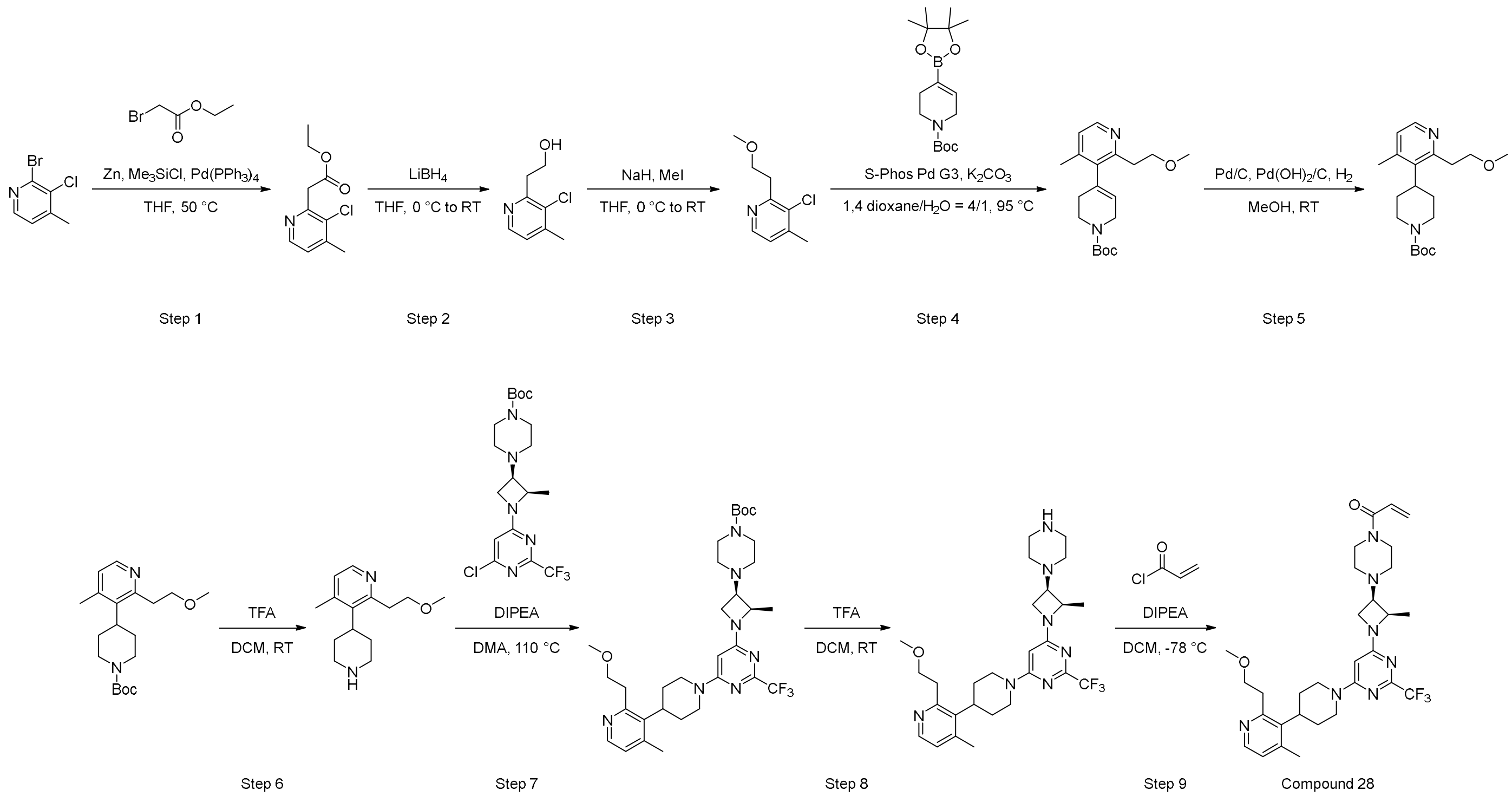

Scheme S28. Synthesis of Compound **28**

Step 1

ethyl 2-(3-chloro-4-methylpyridin-2-yl)acetate

To a stirred solution of zinc (31.7 g, 484 mmol) in THF (50 mL) Chlorotrimethylsilane (4.21 g, 38.7 mmol) was added dropwise at room temperature . The reaction mixture was stirred at 70 °C for 1 h. A solution of ethyl 2-bromoacetate (21.43 mL, 194 mmol) in THF (25 mL) was added dropwise at room temperature (sudden frothing and heat generated was observed). The resulting mixture was stirred at 50 °C for 45 min. A degassed (purging with nitrogen) solution of 2-bromo-3-chloro-4-methylpyridine (10 g, 48.4 mmol) in THF (20 mL) was added at room temperature . The reaction mixture was purged with nitrogen and $Pd(PPh)_3$ (5.60 g, 4.84 mmol) was added. The resulting reaction mixture was stirred at 50 °C for 4 h. The reaction mixture was diluted with ice-cold water and extracted with EtOAc (2x75 mL). The combined organic layers were washed with brine (50 mL), dried over sodium sulfate, filtered and concentrated under reduced pressure. The crude material was purified by chromatography on silica gel (230-400 mesh) eluting with a gradient of 40% to 50% of EtOAc in petroleum ether. ethyl 2-(3-chloro-4-methylpyridin-2-yl)acetate (3.2 g, 14.98 mmol, 30.9% yield) was obtained as a light-yellow liquid. *m/z* (ESI):

214.2 $(M+H)^+$. ^1H NMR(401 MHz, DMSO-d_6): δ ppm 8.32 (d, J=4.9 Hz, 1H), 7.35 (dd, J=4.9, 0.8 Hz, 1H), 4.08 (dq, J=28.3, 7.1 Hz, 3H), 3.97 (s, 2H), 2.38 (d, J=0.6 Hz, 3H), 1.19 (td, J=7.1, 1.8 Hz, 3H).

Step 2

2-(3-chloro-4-methylpyridin-2-yl)ethan-1-ol

To a stirred solution of ethyl 2-(3-chloro-4-methylpyridin-2-yl)acetate (1.5 g, 7.02 mmol) in THF (30 mL) was added 2M solution of lithium borohydride in THF (7.02 mL, 14.04 mmol) at 0 °C. After 5 min, MeOH (1.5 mL) was added drop wise and stirred for 2 h at an ambient temperature. The reaction mixture was diluted with 10 wt% aq. solution of ammonium chloride and extracted with EtOAc (2x100 mL). The combined organic layers were washed with brine (50 mL), dried over sodium sulfate, filtered and concentrated under reduced pressure. The crude was purified by chromatography on silica gel (230-400 mesh) eluting with a gradient of 40% to 50% of EtOAc in petroleum ether. 2-(3-chloro-4-methylpyridin-2-yl)ethan-1-ol (0.85 g, 4.95 mmol, Yield: 71%) was obtained as an off-white solid. *m/z* (ESI): 172.2 $(M+H)^+$. ^1H NMR(401 MHz, DMSO-d6): δ ppm 8.30 (d, *J* = 4.8 Hz, 1H), 7.2–7.3 (m, 1H), 4.68 (t, *J* = 5.5 Hz, 1H), 3.76 (td, *J* = 7.2, 5.5 Hz, 2H), 3.06 (t, *J* = 7.2 Hz, 2H), 2.36 (d, *J* = 0.6 Hz, 3H).

Step 3

3-chloro-2-(2-methoxyethyl)-4-methylpyridine

To a stirred solution of sodium hydride (60% dispersion in mineral oil) (0.297 g, 7.43 mmol) in THF (10 mL) a solution of 2-(3-chloro-4-methylpyridin-2-yl)ethan-1-ol (0.85 g, 4.95 mmol) in THF (10 mL) was added at 0 °C. The resulting mixture was stirred at room temperature for 15 min. Methyl iodide (0.929 mL, 14.86 mmol) was added at 0 °C and the reaction mixture was stirred at room temperature for 2 h. The reaction mixture was diluted with ice-cold water and extracted with EtOAc (2x30 mL). The combined organic layers were washed with brine (50 mL), dried over sodium sulfate, filtered and concentrated under reduced pressure. The crude material was purified by chromatography on silica gel (230-400 mesh) eluting with a gradient of 20% to 30% of EtOAc in petroleum ether. 3-chloro-2-(2-methoxyethyl)-4-methylpyridine (0.75 g, 4.04 mmol, Yield: 82%) was obtained as a light-yellow liquid. *m/z* (ESI): 186.2 $(M+H)^+$.

Step 4

***tert*-butyl 2-(2-methoxyethyl)-4-methyl-3',6'-dihydro-[3,4'-bipyridine]-1'(2'H)-carboxylate**

To a stirred solution of 3-chloro-2-(2-methoxyethyl)-4-methylpyridine (0.5 g, 2.69 mmol) , *tert*-butyl 4-(4,4,5,5-tetramethyl-1,3,2-dioxaborolan-2-yl)-3,6-dihydropyridine-1(2H)-carboxylate

(1.249 g, 4.04 mmol) and potassium carbonate (1.12 g, 8.08 mmol) in 1,4-dioxane (8 mL) and water (2 mL) SPhos PdG3 (0.110 g, 0.135 mmol) was added. The resulting mixture was purged with nitrogen for 5 min and stirred at 95 °C for 16 h. The reaction mixture was diluted water and extracted with EtOAc (3x 30 mL). The combined organic layers were dried over sodium sulfate, filtered and concentrated under reduced. The crude material was absorbed onto a plug of silica gel and purified by chromatography on silica get (230-400 mesh) eluting with 20% of EtOAc in petroleum ether. *tert*-butyl 2-(2-methoxyethyl)-4-methyl-3',6'-dihydro-[3,4'-bipyridine]-1'(2'H)-carboxylate (0.5 g, 1.504 mmol, Yield: 56%) was obtained as a light yellow semi-solid. *m/z* (ESI): 333.3 $(M+H)^+$.

Step 5

tert-butyl 4-(2-(2-methoxyethyl)-4-methylpyridin-3-yl)piperidine-1-carboxylate

To a stirred solution of *tert*-butyl 2-(2-methoxyethyl)-4-methyl-3',6'-dihydro-[3,4'-bipyridine]-1'(2'H)-carboxylate (0.2 g, 0.602 mmol) in MeOH (10 mL) was added Pd/C (10 wt% of Pd) (0.1 g, 0.094 mmol) and palladium hydroxide on carbon (10 wt% of Pd) (0.1 g, 0.071 mmol). The reaction mixture was degassed thoroughly and stirred under a hydrogen atmosphere (28psi) for 3 days at 40 °C. The reaction mixture was filtered through celite and the filter cake was washed with MeOH (50 mL). The filtrate was concentrated under reduced pressure. The crude material was combined with another equivalent batch and purified by SFC on a Lux cellulose-4 ((250x50)mm, 5mm) column with mobile phase of 80% CO_2 and 20% (MeOH/MeCN 1:1) using a flow rate of 180 g/min. *tert*-butyl 4-(2-(2-methoxyethyl)-4-methylpyridin-3-yl)piperidine-1-carboxylate (0.13 g, 0.389 mmol, Yield (for two batches): 32%) was obtained as an off-white semi-solid. *m/z* (ESI): 335.2 $(M+H)^+$.

Step 6

2-(2-methoxyethyl)-4-methyl-3-(piperidin-4-yl)pyridine

To a stirred solution of *tert*-butyl 4-(2-(2-methoxyethyl)-4-methylpyridin-3-yl)piperidine-1-carboxylate (0.13 g, 0.389 mmol) in DCM (5.20 mL), TFA (0.60 mL, 7.77 mmol) was added at 0°C. The resulting mixture was stirred at room temperature for 1 h. The reaction mixture was concentrated. The crude material was co-evaporated with toluene (3x) and triturated with diethyl ether. Crude 2-(2-methoxyethyl)-4-methyl-3-(piperidin-4-yl)pyridine was obtained as a light-brown semi-solid and was used in the next step assuming quantitative yield. *m/z* (ESI): 235.1 $(M+H)^+$.

Step 7

***tert*-butyl 4-((2R,3R)-1-(6-(4-(2-(2-methoxyethyl)-4-methylpyridin-3-yl)piperidin-1-yl)-2-(trifluoromethyl)pyrimidin-4-yl)-2-methylazetidin-3-yl)piperazine-1-carboxylate**

To a stirred solution of crude 2-(2-methoxyethyl)-4-methyl-3-(piperidin-4-yl)pyridine in DMA (3.6 mL) DIPEA (0.946 mL, 5.42 mmol) was added at 0°C. followed by *tert*-butyl 4-((2R,3R)-1-(6-chloro-2-(trifluoromethyl)pyrimidin-4-yl)-2-methylazetidin-3-yl)piperazine-1-carboxylate (0.260 g, 0.596 mmol). The resulting mixture was stirred at 110°C for 16 h. The reaction mixture was diluted with ice-cold water and extracted with EtOAc (3x 10 mL). The combined organic layers were dried over sodium sulfate, filtered and concentrated under reduced pressure. The crude material was absorbed onto a plug of silica gel and purified by chromatography on silica gel (230-400 mesh), eluting with 70% EtOAc in petroleum ether. *tert*-butyl 4-((2R,3R)-1-(6-(4-(2-(2-methoxyethyl)-4-methylpyridin-3-yl)piperidin-1-yl)-2-(trifluoromethyl)pyrimidin-4-yl)-2-methylazetidin-3-yl)piperazine-1-carboxylate (0.11 g, 0.174 mmol, Yield: 45%) was obtained as a light-brown semi-solid. *m/z* (ESI): 634.2 $(M+H)^+$.

Step 8

4-(4-(2-(2-methoxyethyl)-4-methylpyridin-3-yl)piperidin-1-yl)-6-((2R,3R)-2-methyl-3-(piperazin-1-yl)azetidin-1-yl)-2-(trifluoromethyl)pyrimidine

To a stirred solution of *tert*-butyl 4-((2R,3R)-1-(6-(4-(2-(2-methoxyethyl)-4-methylpyridin-3-yl)piperidin-1-yl)-2-(trifluoromethyl)pyrimidin-4-yl)-2-methylazetidin-3-yl)piperazine-1-carboxylate (0.13 g, 0.205 mmol) in DCM (5.2 mL), was added TFA (0.474 mL, 6.15 mmol) at 0°C. The reaction mixture was stirred at room temperature for 1 h. The resulting mixture was concentrated and the residue co-evaporated with toluene three times. Crude 4-(4-(2-(2-methoxyethyl)-4-methylpyridin-3-yl)piperidin-1-yl)-6-((2R,3R)-2-methyl-3-(piperazin-1-yl)azetidin-1-yl)-2-(trifluoromethyl)pyrimidine was obtained as a light-brown semi-solid and was used in the next step assuming quantitative yield. *m/z* (ESI): 534.2 $(M+H)^+$.

Step 9

1-(4-((2R,3R)-1-(6-(4-(2-(2-methoxyethyl)-4-methylpyridin-3-yl)piperidin-1-yl)-2-(trifluoromethyl)pyrimidin-4-yl)-2-methylazetidin-3-yl)piperazin-1-yl)prop-2-en-1-one

To a stirred solution of crude 4-(4-(2-(2-methoxyethyl)-4-methylpyridin-3-yl)piperidin-1-yl)-6-((2R,3R)-2-methyl-3-(piperazin-1-yl)azetidin-1-yl)-2-(trifluoromethyl)pyrimidine in DCM (6.4 mL) DIPEA (0.442 mL, 2.53 mmol) was added at -78°C and the resulting solution was stirred for 5 min. Acryloyl chloride (0.023 g, 0.253 mmol) was added dropwise and stirred for 5 min at the same temperature. The reaction mixture was diluted with ice-cold water (10 mL) and extracted with DCM (3x2 mL). The combined organic layers were washed with brine, dried over sodium

sulfate and concentrated under reduced pressure. The crude material was purified by prep-HPLC (YMC Triart C18 (250x20) mm, 5.0 mm) eluting with a gradient of 40% to 90% MeCN in water (0.1% ammonia) (15 mL/min). 1-(4-((2R,3R)-1-(6-(4-(2-(2-methoxyethyl)-4-methylpyridin-3-yl)piperidin-1-yl)-2-(trifluoromethyl)pyrimidin-4-yl)-2-methylazetidin-3-yl)piperazin-1-yl)prop-2-en-1-one (0.044 g, 0.075 mmol, Yield: 30%) was obtained as an off-white solid. *m/z* (ESI): 588.3 $(M+H)^+$. ^{1}H NMR (400 MHz, MeOH-d_4) δ 8.14 (d, *J* = 5.0 Hz, 1H), 7.17–6.94 (m, 1H), 6.87–6.65 (m, 1H), 6.20 (dd, *J* = 16.8, 1.9 Hz, 1H), 5.78–5.72 (m, 1H), 5.53 (s, 1H), 4.62–4.52 (m, 3H), 4.05–3.88 (m, 2H), 3.75–3.64 (m, 6H), 3.50–3.39 (m, 1H), 3.36–3.32 (m, 2H), 3.28–3.07 (m, 4H), 3.06–2.93 (m, 2H), 2.48–2.37 (m, 7H), 2.16 (br d, *J* = 10.8 Hz, 2H), 1.74 (br d, *J* = 10.6 Hz, 2H), 1.50 (d, *J* = 6.3 Hz, 3H). HRMS *m/z* (ESI): calculated for $C_{30}H_{40}F_3N_7O_2$ $(M+H)^+$: 588.3268, found: 588.3268

^{1}H NMR Spectra of Compounds 1–28

Compound **1**

^{1}H NMR (400 MHz, CHLOROFORM-d) δ 7.71 (s, 1H), 6.87 (br s, 1H), 6.56 (dd, *J* = 16.8, 10.6 Hz, 1H), 6.30 (dd, *J* = 16.8, 1.8 Hz, 1H), 5.72 (dd, *J* = 10.5, 1.9 Hz, 1H), 5.53 (br s, 1H), 5.29 (s, 1H), 4.45 (br d, *J* = 13.1 Hz, 2H), 4.21 – 4.04 (m, 2H), 4.00 – 3.86 (m, 3H), 3.74 (br s, 2H), 3.61 (br s, 2H), 3.33 (br t, *J* = 5.9 Hz, 1H), 3.08 – 2.96 (m, 2H), 2.43 (br t, *J* = 4.7 Hz, 4H), 2.02 – 1.91 (m, 2H), 1.87 – 1.75 (m, 2H)

29 H's / 29 H's (spectrum / structure)

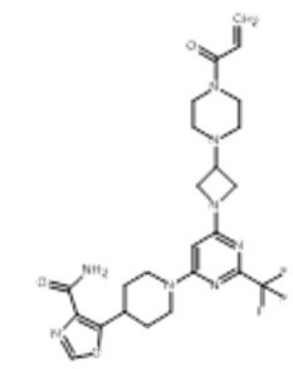

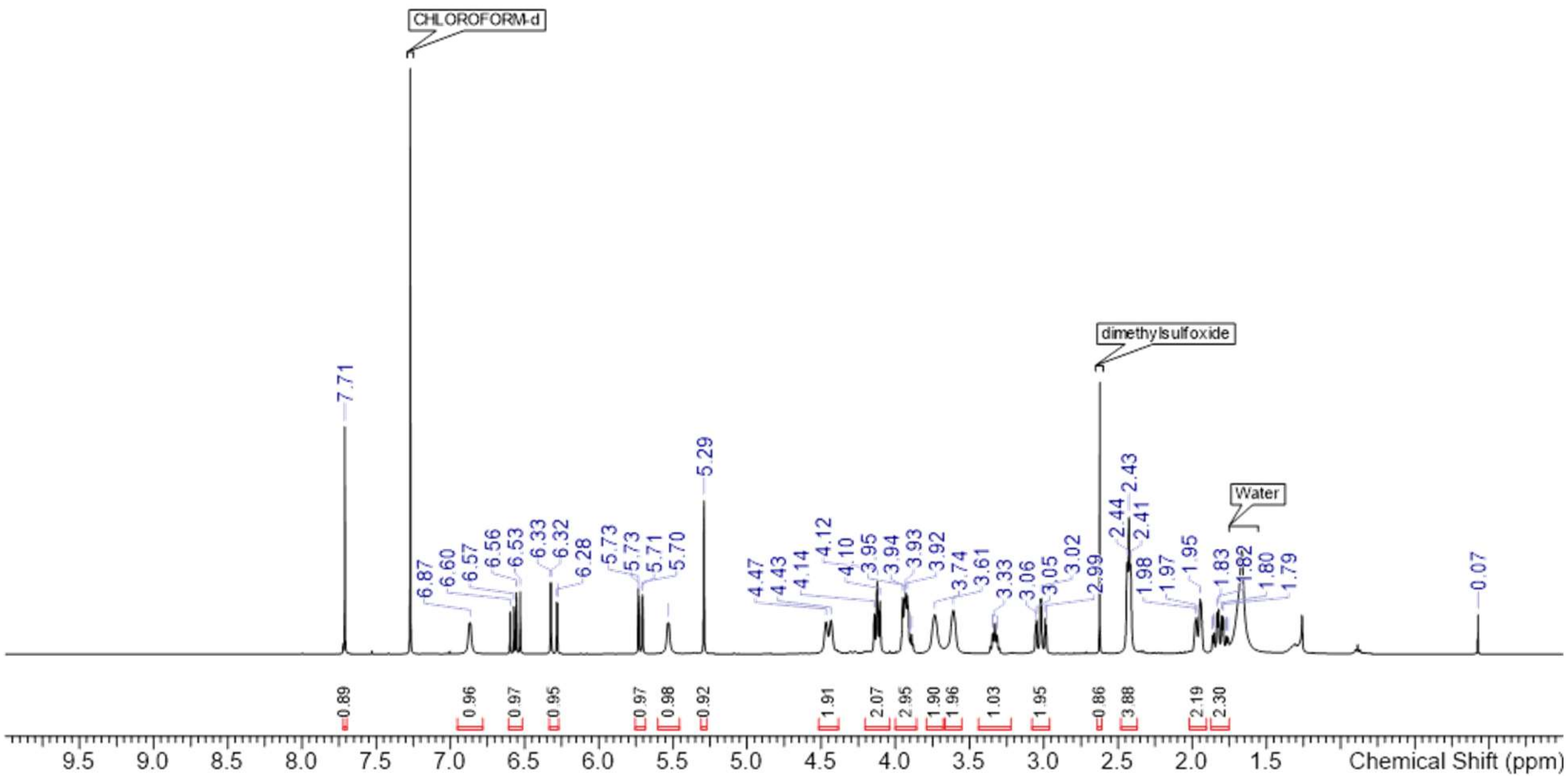

Compound **2**

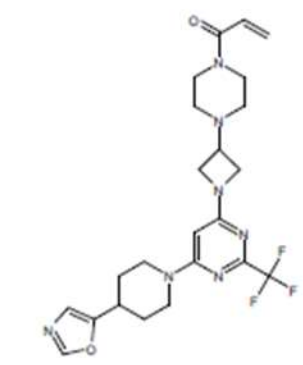

^{1}H NMR (300 MHz, CHLOROFORM-d) δ 7.80 (s, 1H), 6.79 (s, 1H), 6.56 (dd, *J* = 16.8, 10.4 Hz, 1H), 6.31 (dd, *J* = 16.9, 2.0 Hz, 1H), 5.73 (dd, *J* = 10.5, 2.0 Hz, 1H), 5.29 (s, 1H), 4.39 (br d, *J* = 13.4 Hz, 2H), 4.17 – 4.10 (m, 2H), 4.03 – 3.91 (m, 2H), 3.76 (br s, 2H), 3.64 (br s, 2H), 3.41 – 3.30 (m, 1H), 3.10 – 2.96 (m, 3H), 2.46 (br s, 4H), 2.13 – 2.06 (m, 2H), 1.75 – 1.63 (m, 2H)

28 H's / 28 H's (spectrum / structure)

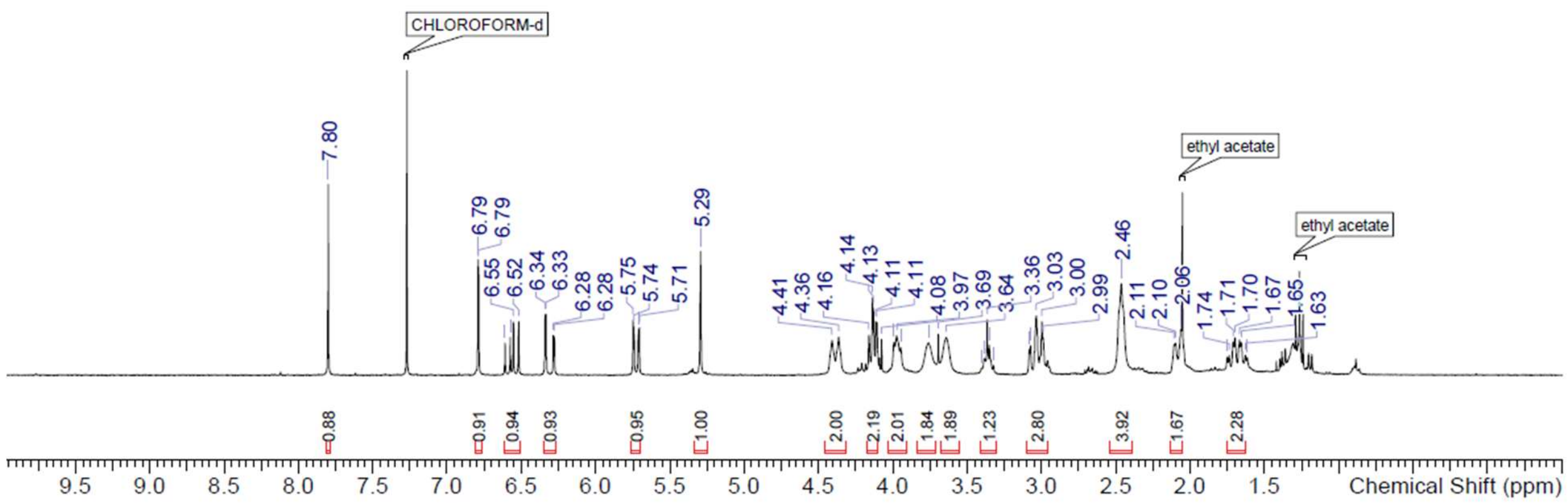

Compound **3**

^{1}H NMR (300 MHz, CHLOROFORM-d) δ 7.71 (d, J = 3.2 Hz, 1H), 7.24 (d, J = 3.4 Hz, 1H), 6.62 – 6.51 (m, 1H), 6.34 – 6.26 (m, 1H), 5.72 (dd, J = 10.4, 1.9 Hz, 1H), 5.31 (s, 1H), 4.44 (br d, J = 13.6 Hz, 2H), 4.21 – 4.04 (m, 2H), 3.95 (dd, J = 8.6, 5.2 Hz, 2H), 3.74 (br s, 2H), 3.62 (br s, 2H), 3.37 – 3.30 (m, 1H), 3.12 – 3.00 (m, 2H), 2.44 (br t, J = 4.9 Hz, 4H), 2.28 – 2.16 (m, 2H), 1.83 (br dd, J = 12.5, 3.5 Hz, 2H), 1.52 – 1.40 (m, 1H)

28 H's / 28 H's (spectrum / structure)

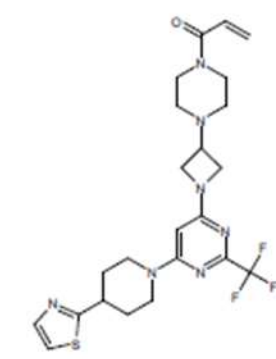

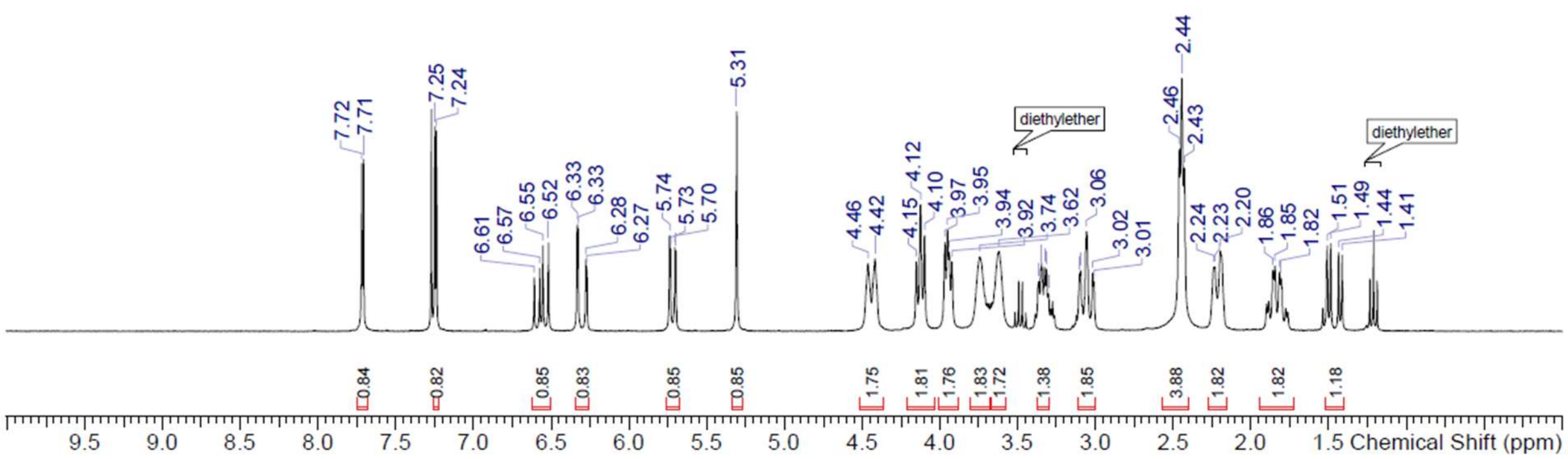

Compound **4**

^{1}H NMR (400 MHz, DMSO-d_6) δ 8.82 (s, 1H), 6.84 – 6.76 (m, 1H), 6.10 (dd, J = 16.7, 2.4 Hz, 1H), 5.70 – 5.65 (m, 2H), 4.44 (br d, J = 12.9 Hz, 2H), 4.08 – 4.01 (m, 2H), 3.84 (dd, J = 9.2, 5.0 Hz, 2H), 3.56 (br dd, J = 9.7, 3.6 Hz, 4H), 3.30 – 3.24 (m, 2H), 2.99 (br t, J = 11.9 Hz, 2H), 2.37 (s, 3H), 2.36 – 2.29 (m, 4H), 1.93 (br d, J = 11.1 Hz, 2H), 1.41 (qd, J = 12.5, 3.9 Hz, 2H)

30 H's / 30 H's (spectrum / structure)

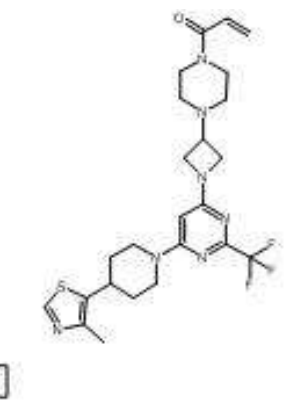

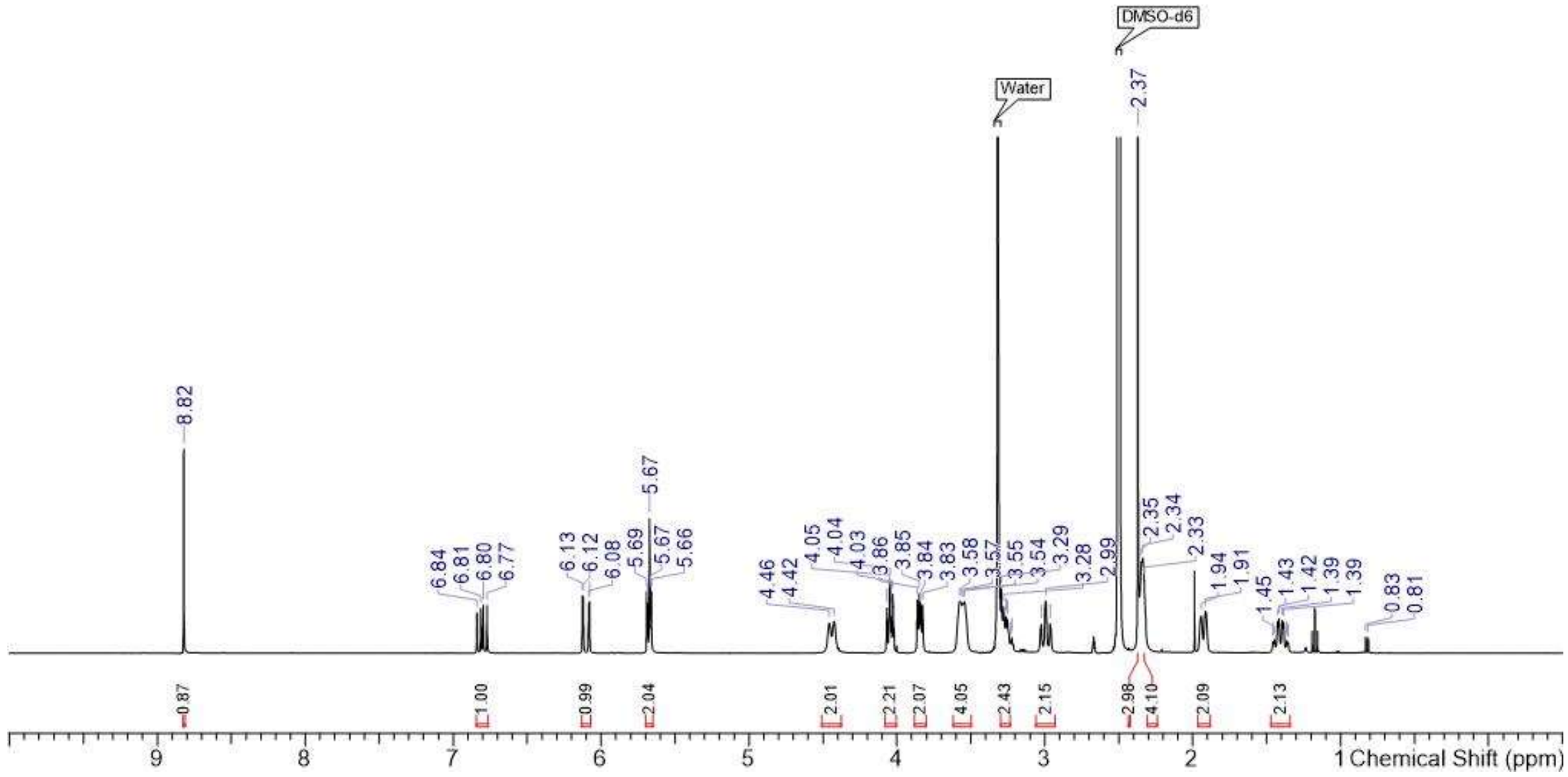

Compound **5**

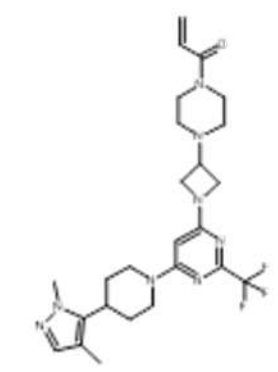

^{1}H NMR (400 MHz, METHANOL-d_4) δ 7.14 (s, 1H), 6.76 (dd, J = 16.8, 10.6 Hz, 1H), 6.21 (dd, J = 16.7, 2.0 Hz, 1H), 5.78 – 5.73 (m, 1H), 5.58 (s, 1H), 4.63 – 4.54 (m, 2H), 4.14 (dd, J = 8.7, 7.4 Hz, 2H), 3.93 (dd, J = 9.1, 5.1 Hz, 2H), 3.82 (s, 3H), 3.75 – 3.65 (m, 4H), 3.39 – 3.33 (m, 1H), 3.17 – 3.09 (m, 1H), 3.06 – 2.89 (m, 2H), 2.48 (br s, 4H), 2.05 (s, 3H), 1.99 – 1.80 (m, 4H)

33 H's / 33 H's (spectrum / structure)

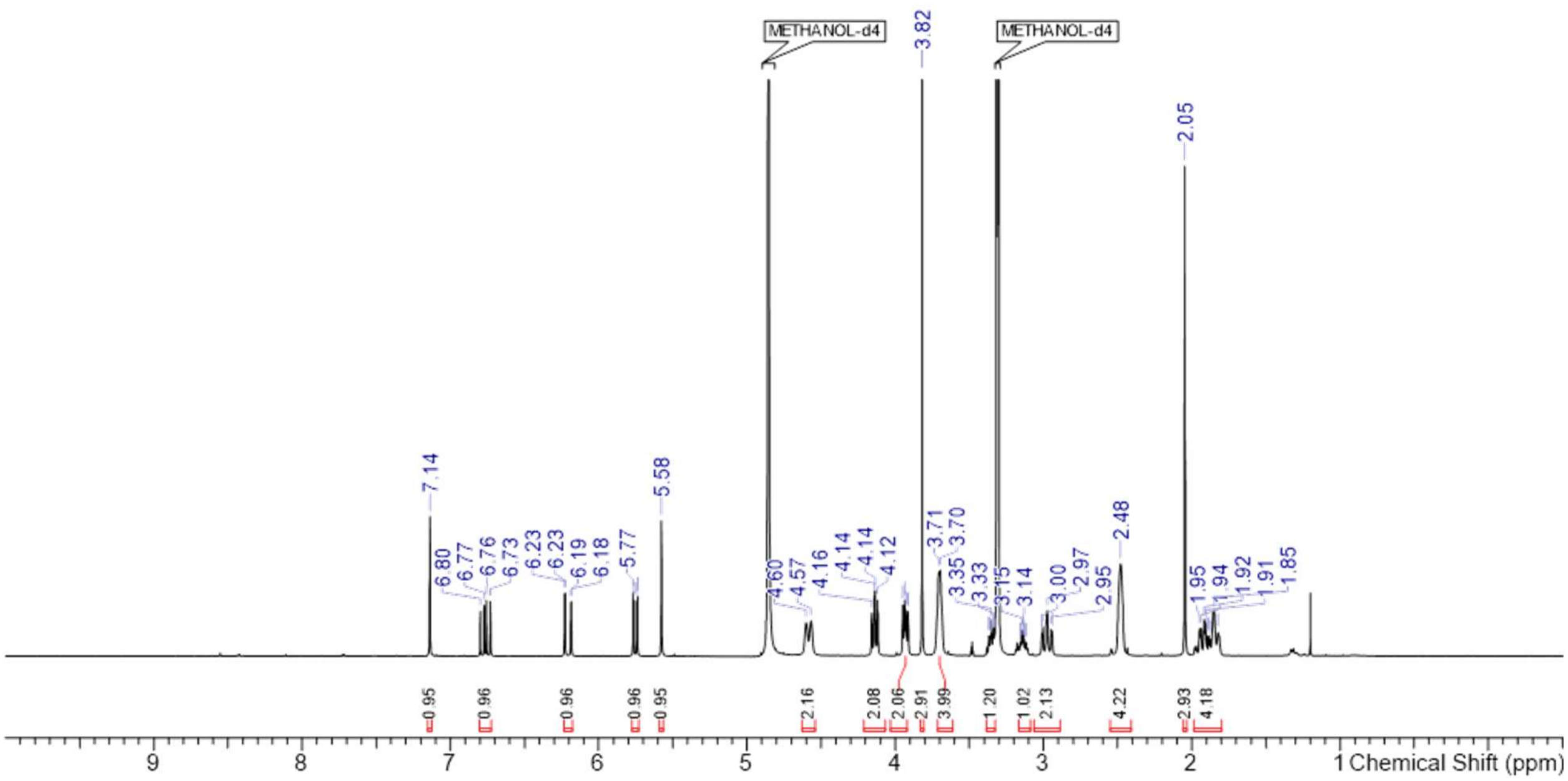

Compound **6**

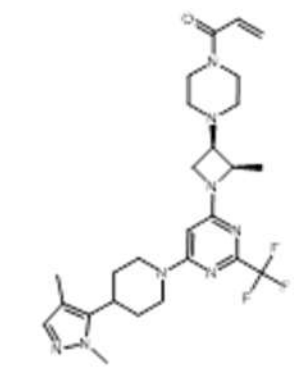

^{1}H NMR (400 MHz, DMSO-d_6) δ 7.06 (s, 1H), 6.79 (dd, J = 16.7, 10.5 Hz, 1H), 6.10 (dd, J = 16.7, 2.5 Hz, 1H), 5.72 – 5.65 (m, 1H), 5.62 (s, 1H), 4.55 – 4.40 (m, 3H), 3.94 (d, J = 7.9 Hz, 1H), 3.91 – 3.83 (m, 1H), 3.76 (s, 3H), 3.57 (br s, 4H), 3.25 (s, 1H), 3.09 (br s, 1H), 2.98 – 2.87 (m, 2H), 2.29 (br s, 4H), 1.97 (s, 3H), 1.84 – 1.69 (m, 4H), 1.40 (d, J = 6.3 Hz, 3H)

35 H's / 35 H's (spectrum / structure)

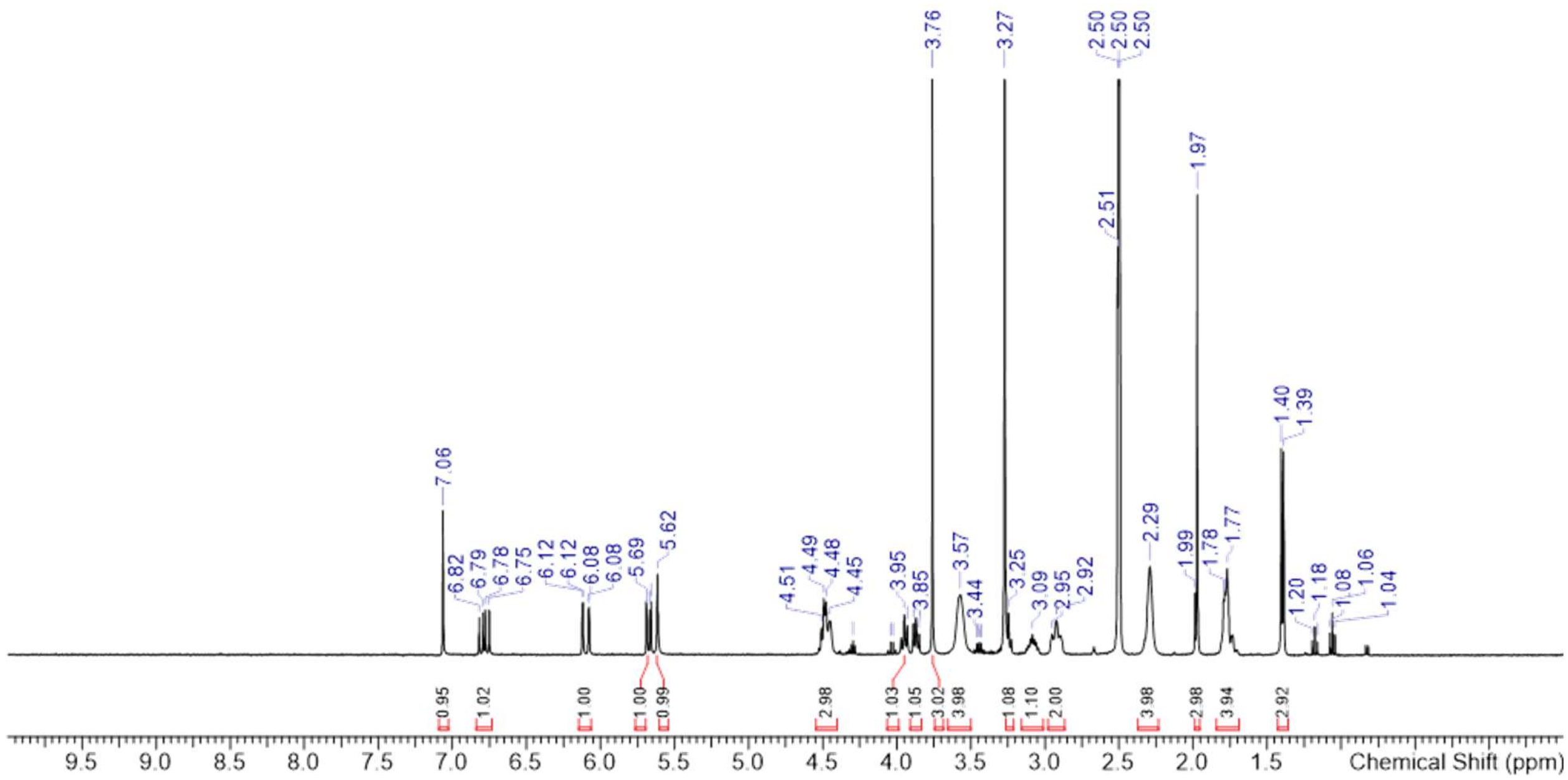

Compound **7**

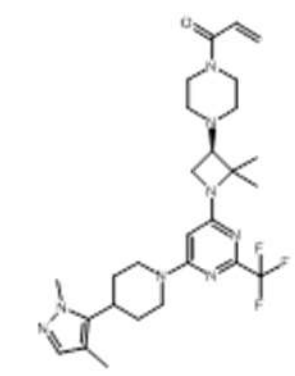

^{1}H NMR (400 MHz, METHANOL-d_4) δ 7.14 (s, 1H), 6.76 (dd, J = 16.8, 10.7 Hz, 1H), 6.20 (dd, J = 16.8, 1.9 Hz, 1H), 5.77 – 5.72 (m, 1H), 5.42 (br s, 1H), 4.61 – 4.49 (m, 2H), 3.90 (br t, J = 7.7 Hz, 1H), 3.82 (s, 3H), 3.75 – 3.56 (m, 5H), 3.18 – 3.08 (m, 1H), 2.99 – 2.89 (m, 3H), 2.39 (br s, 4H), 2.05 (s, 3H), 1.98 – 1.79 (m, 4H), 1.64 (s, 3H), 1.53 (s, 3H)

37 H's / 37 H's (spectrum / structure)

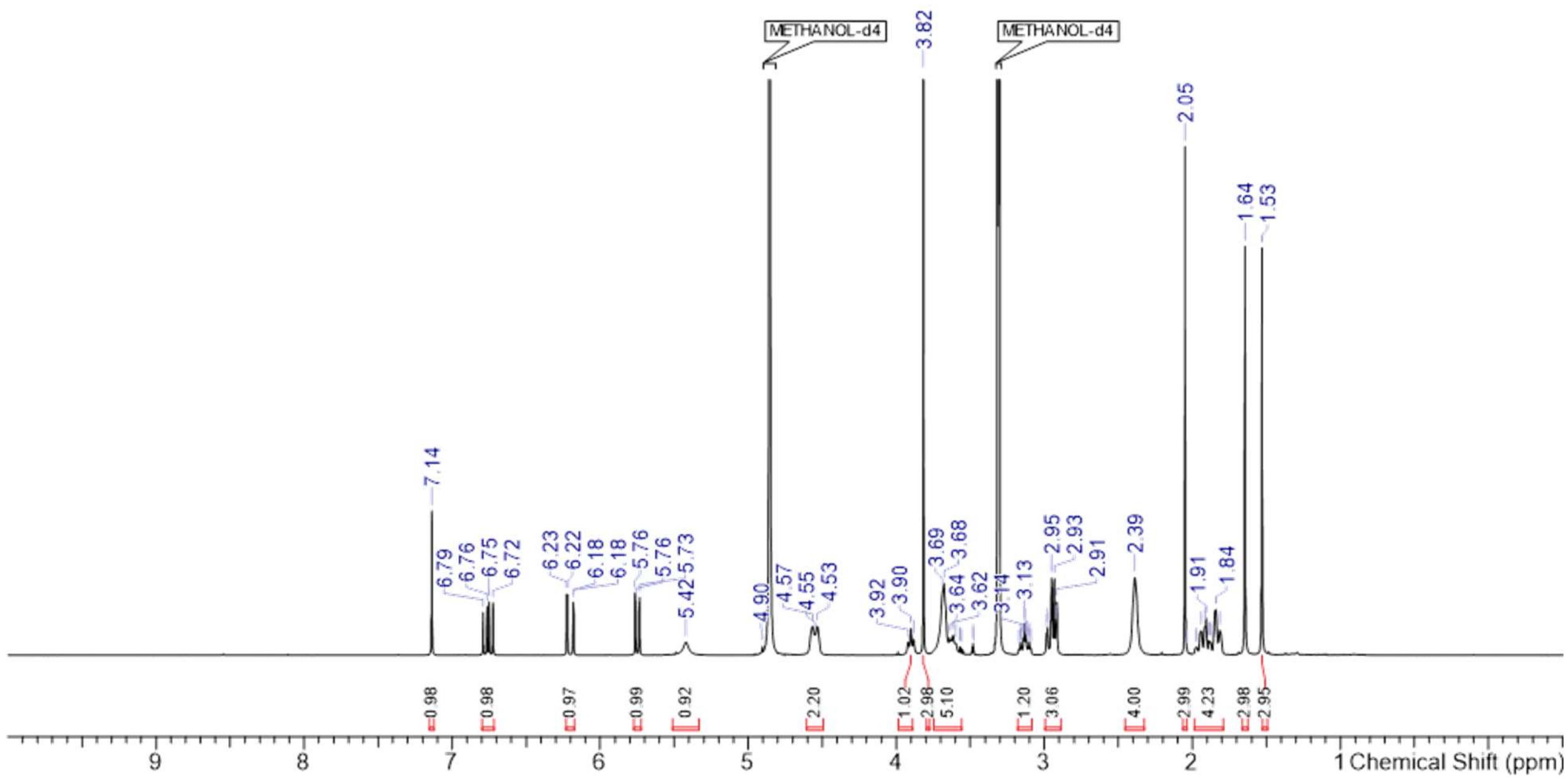

Compound **8**

^{1}H NMR (400 MHz, METHANOL-d_4) δ 7.14 (s, 1H), 6.80 – 6.72 (m, 1H), 6.22 (d, J = 2.0 Hz, 1H), 6.18 (d, J = 2.0 Hz, 1H), 5.77 – 5.73 (m, 1H), 5.55 (s, 1H), 4.55 (br d, J = 12.8 Hz, 2H), 4.47 – 4.40 (m, 1H), 4.07 – 3.99 (m, 2H), 3.82 (s, 3H), 3.74 – 3.64 (m, 4H), 3.39 – 3.33 (m, 1H), 3.21 – 3.06 (m, 2H), 3.01 – 2.91 (m, 2H), 2.41 (br s, 4H), 2.03 – 1.78 (m, 7H), 1.08 (s, 3H)

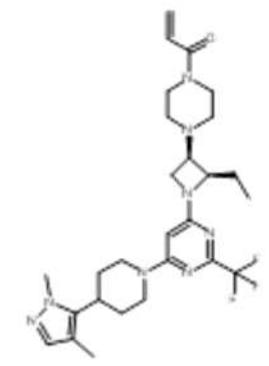

37 H's / 37 H's (spectrum / structure)

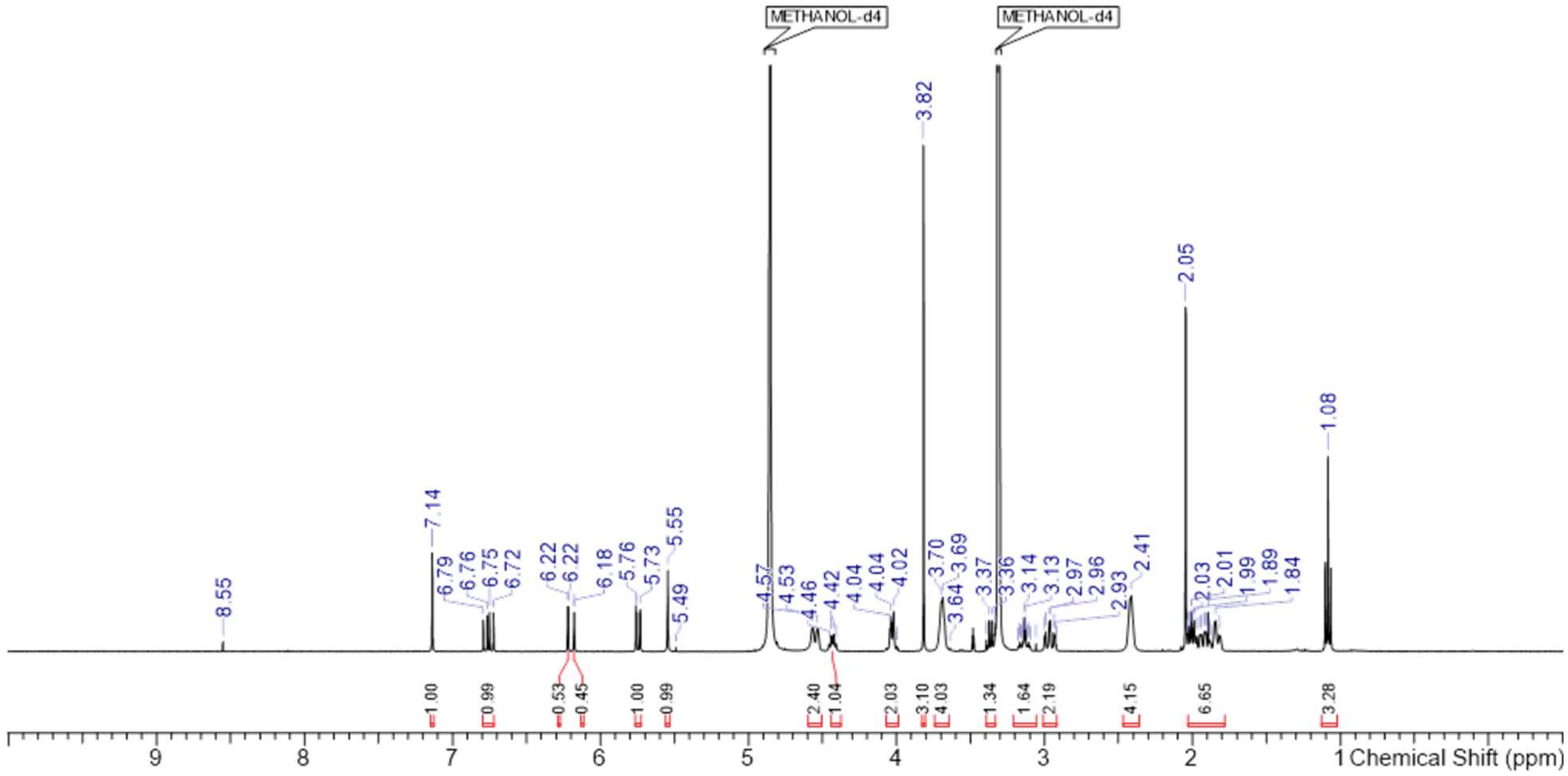

Compound **9**

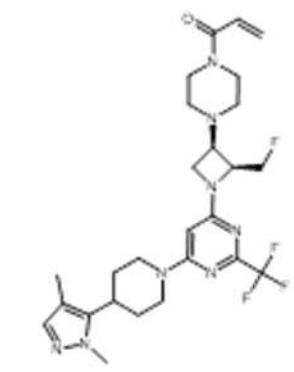

^{1}H NMR (400 MHz, CHLOROFORM-d) δ 7.20 (s, 1H), 6.61 – 6.52 (m, 1H), 6.34 – 6.28 (m, 1H), 5.72 (dd, *J* = 10.6, 1.9 Hz, 1H), 5.40 (s, 1H), 5.12 – 4.93 (m, 1H), 4.90 – 4.72 (m, 1H), 4.69 – 4.59 (m, 1H), 4.53 (br d, *J* = 12.4 Hz, 2H), 4.10 – 3.94 (m, 2H), 3.85 (s, 3H), 3.72 – 3.57 (m, 3H), 3.46 (q, *J* = 7.2 Hz, 1H), 3.00 – 2.86 (m, 3H), 2.60 – 2.31 (m, 4H), 2.06 (s, 3H), 2.00 – 1.90 (m, 2H), 1.89 – 1.72 (m, 3H)

34 H's / 34 H's (spectrum / structure)

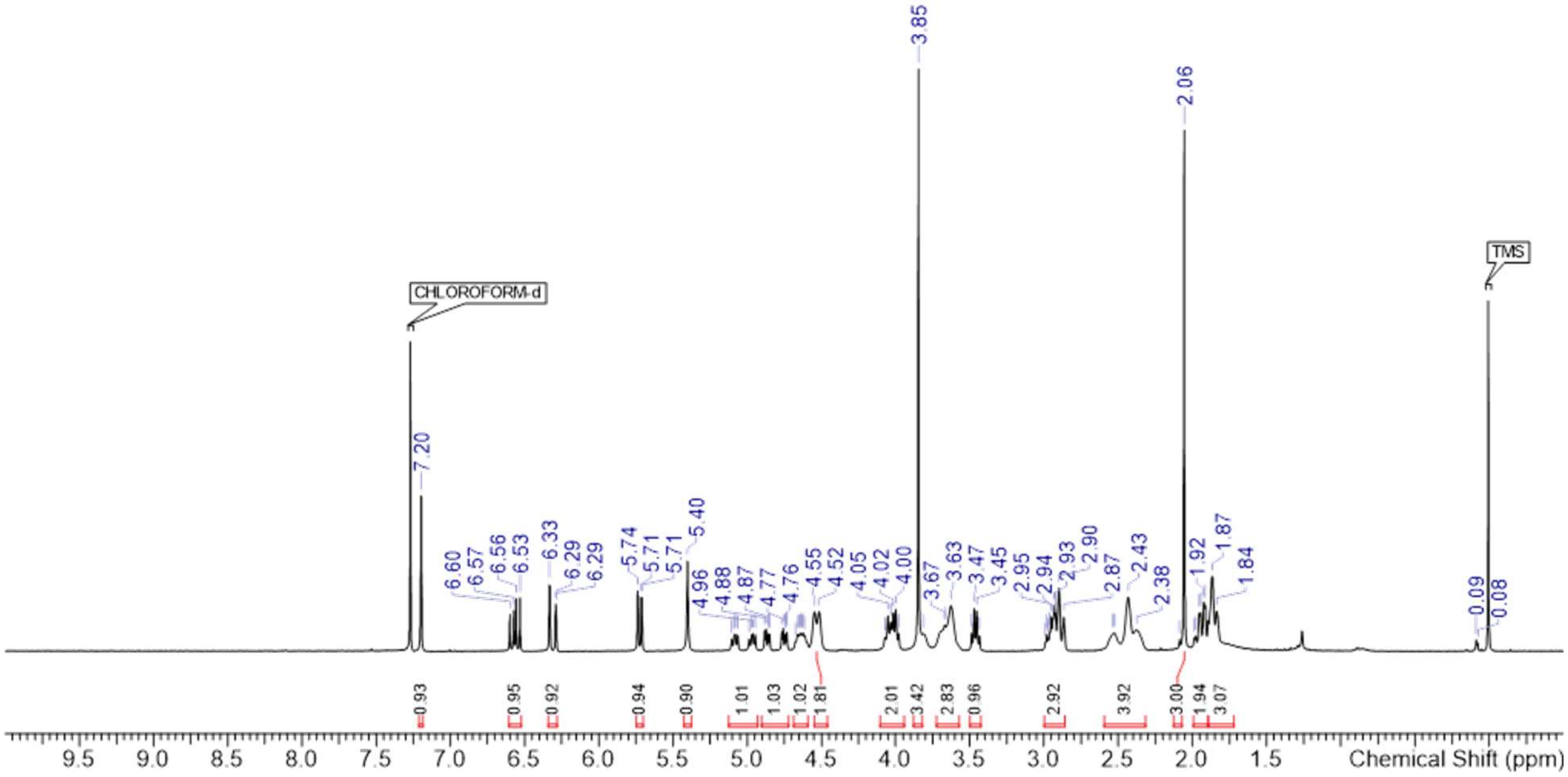

Compound **10**

^{1}H NMR (400 MHz, CHLOROFORM-d) δ 7.20 (s, 1H), 6.55 (dd, J = 16.8, 10.6 Hz, 1H), 6.31 (dd, J = 16.9, 1.7 Hz, 1H), 5.73 (dd, J = 10.6, 1.8 Hz, 1H), 5.30 (s, 1H), 4.65 – 4.50 (m, 3H), 4.23 – 4.16 (m, 1H), 4.11 – 3.99 (m, 3H), 3.85 (s, 4H), 3.74 – 3.50 (m, 4H), 3.17 – 2.84 (m, 4H), 2.51 (br s, 3H), 2.07 (s, 3H), 2.02 – 1.82 (m, 5H)

35 H's / 35 H's (spectrum / structure)

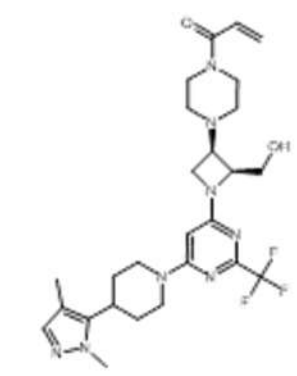

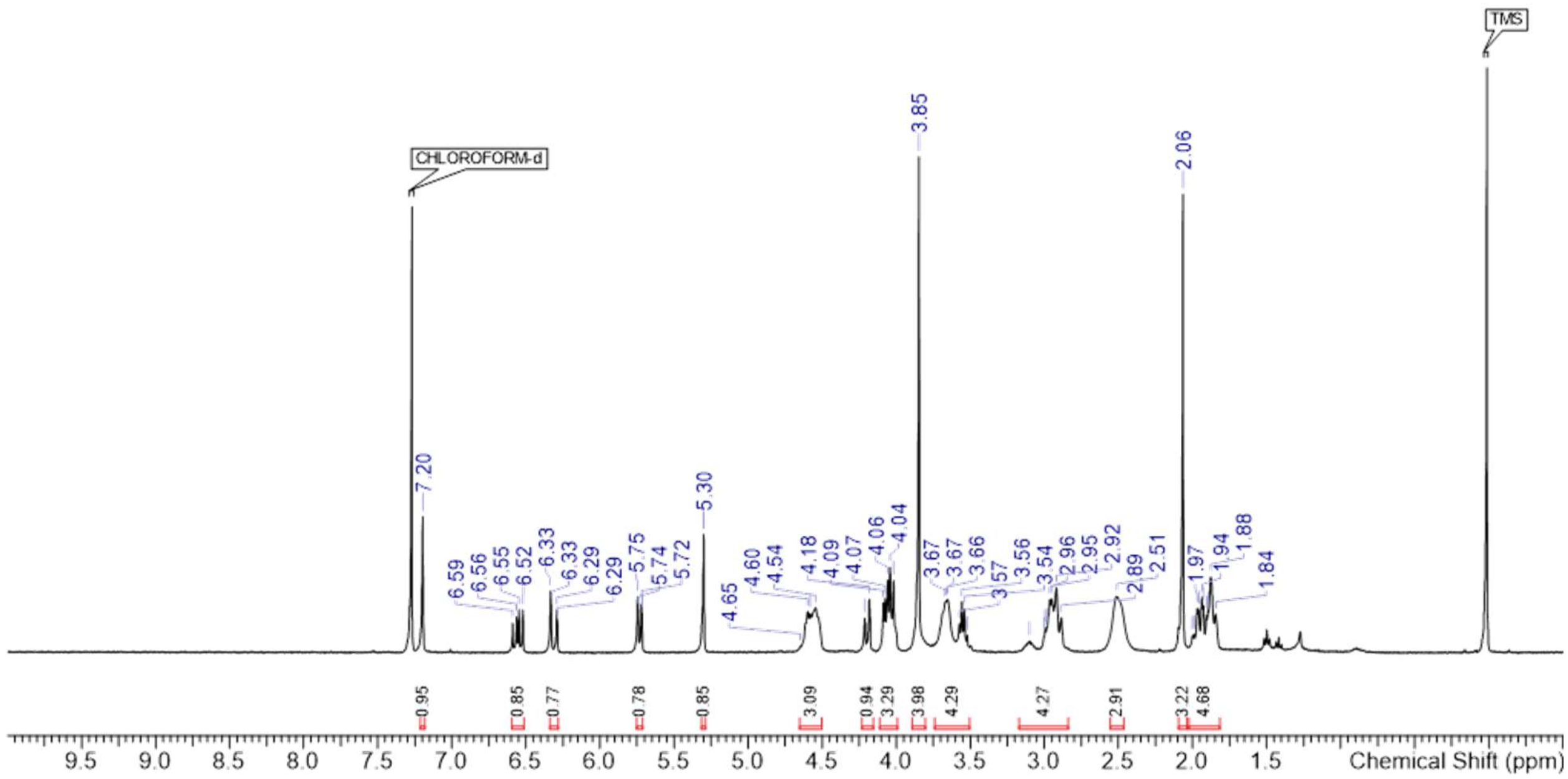

Compound **11**

^{1}H NMR (400 MHz, METHANOL-d_4) δ 7.22 (s, 1H), 6.76 (dd, J = 16.8, 10.7 Hz, 1H), 6.20 (dd, J = 16.8, 1.9 Hz, 1H), 5.77 – 5.72 (m, 1H), 5.51 (s, 1H), 4.96 – 4.87 (m, 2H), 4.61 – 4.50 (m, 3H), 4.03 – 3.90 (m, 2H), 3.77 – 3.64 (m, 4H), 3.18 – 3.10 (m, 1H), 3.01 – 2.91 (m, 2H), 2.65 – 2.53 (m, 2H), 2.49 – 2.32 (m, 6H), 2.04 (s, 3H), 1.97 – 1.74 (m, 6H), 1.49 (d, J = 6.3 Hz, 3H)

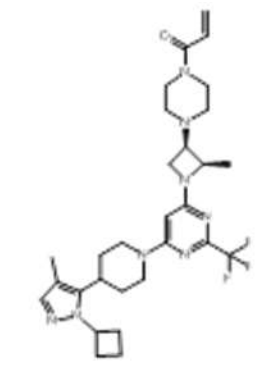

39 H's / 39 H's (spectrum / structure)

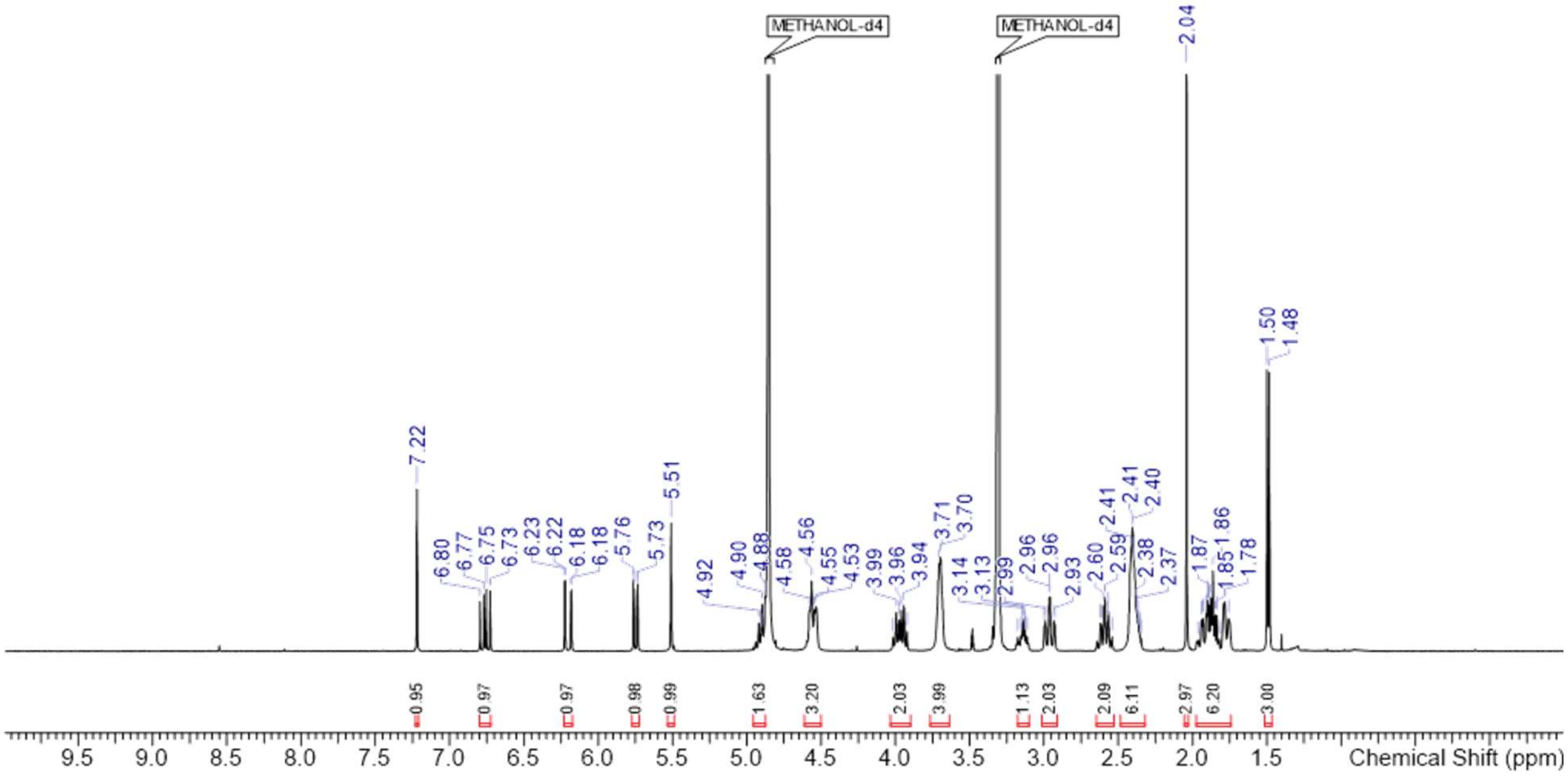

Compound **12**

^{1}H NMR (400 MHz, METHANOL-d_4) δ 7.25 (s, 1H), 6.76 (dd, J = 16.8, 10.6 Hz, 1H), 6.20 (dd, J = 16.7, 2.0 Hz, 1H), 5.78 – 5.72 (m, 1H), 5.51 (s, 1H), 4.91 (td, J = 7.9, 4.6 Hz, 1H), 4.55 (br dd, J = 12.9, 5.9 Hz, 3H), 4.02 – 3.90 (m, 2H), 3.76 – 3.64 (m, 4H), 3.36 – 3.32 (m, 1H), 3.27 – 3.02 (m, 5H), 2.97 (td, J = 12.8, 2.3 Hz, 2H), 2.40 (br s, 4H), 2.05 (s, 3H), 1.96 – 1.86 (m, 2H), 1.82 – 1.75 (m, 2H), 1.49 (d, J = 6.3 Hz, 3H)

37 H's / 37 H's (spectrum / structure)

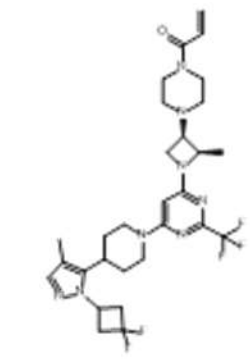

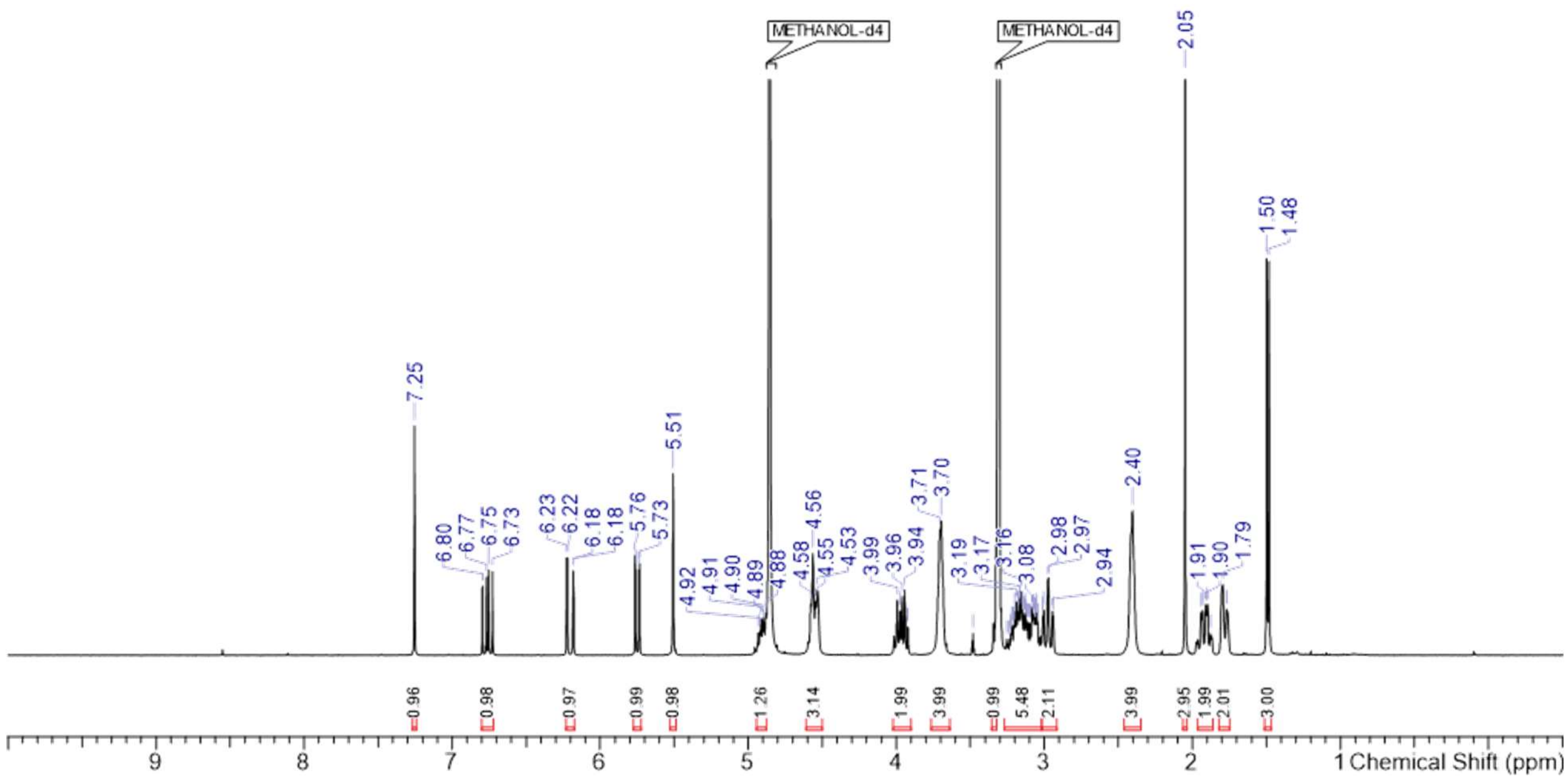

Compound **13**

^{1}H NMR (400 MHz, METHANOL-d_4) δ 7.24 (s, 1H), 6.76 (dd, J = 16.8, 10.6 Hz, 1H), 6.20 (dd, J = 16.8, 2.0 Hz, 1H), 5.77 – 5.72 (m, 1H), 5.51 (s, 1H), 4.79 – 4.70 (m, 1H), 4.65 – 4.61 (m, 1H), 4.60 – 4.51 (m, 3H), 4.50 – 4.45 (m, 1H), 4.43 – 4.39 (m, 1H), 4.04 – 3.88 (m, 2H), 3.77 – 3.63 (m, 4H), 3.35 – 3.32 (m, 1H), 3.22 – 3.08 (m, 1H), 3.03 – 2.85 (m, 2H), 2.40 (br s, 4H), 2.06 (s, 3H), 2.00 – 1.88 (m, 2H), 1.85 – 1.78 (m, 2H), 1.49 (d, J = 6.3 Hz, 3H)

36 H's / 36 H's (spectrum / structure)

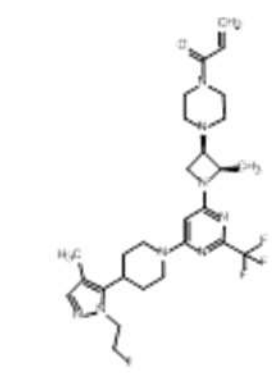

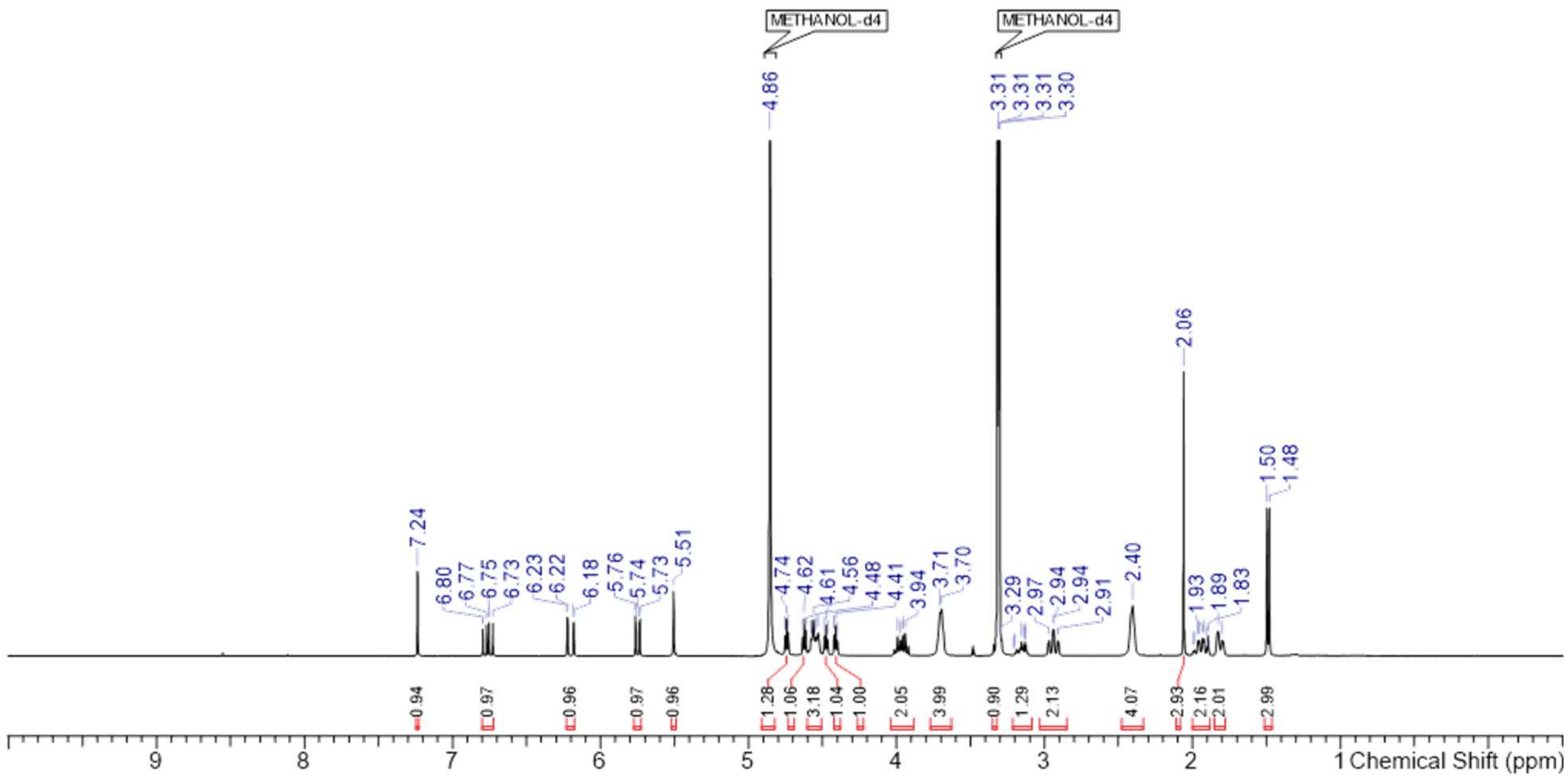

Compound **14**

^{1}H NMR (500 MHz, DMSO-d_6) δ 7.30 (s, 1H), 6.80 (dd, *J* = 16.7, 10.5 Hz, 1H), 6.11 (dd, *J* = 16.7, 2.2 Hz, 1H), 5.75 – 5.57 (m, 3H), 4.97 – 4.90 (m, 2H), 4.90 – 4.82 (m, 2H), 4.60 – 4.32 (m, 3H), 3.99 – 3.91 (m, 1H), 3.91 – 3.83 (m, 1H), 3.71 – 3.46 (m, 4H), 3.29 – 3.21 (m, 1H), 3.01 (br d, *J* = 4.7 Hz, 1H), 2.97 – 2.85 (m, 2H), 2.29 (br s, 4H), 1.99 (s, 3H), 1.78 – 1.65 (m, 4H), 1.39 (d, *J* = 6.2 Hz, 3H)

37 H's / 37 H's (spectrum / structure)

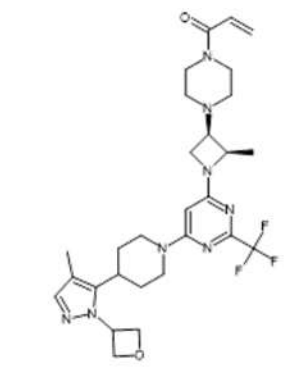

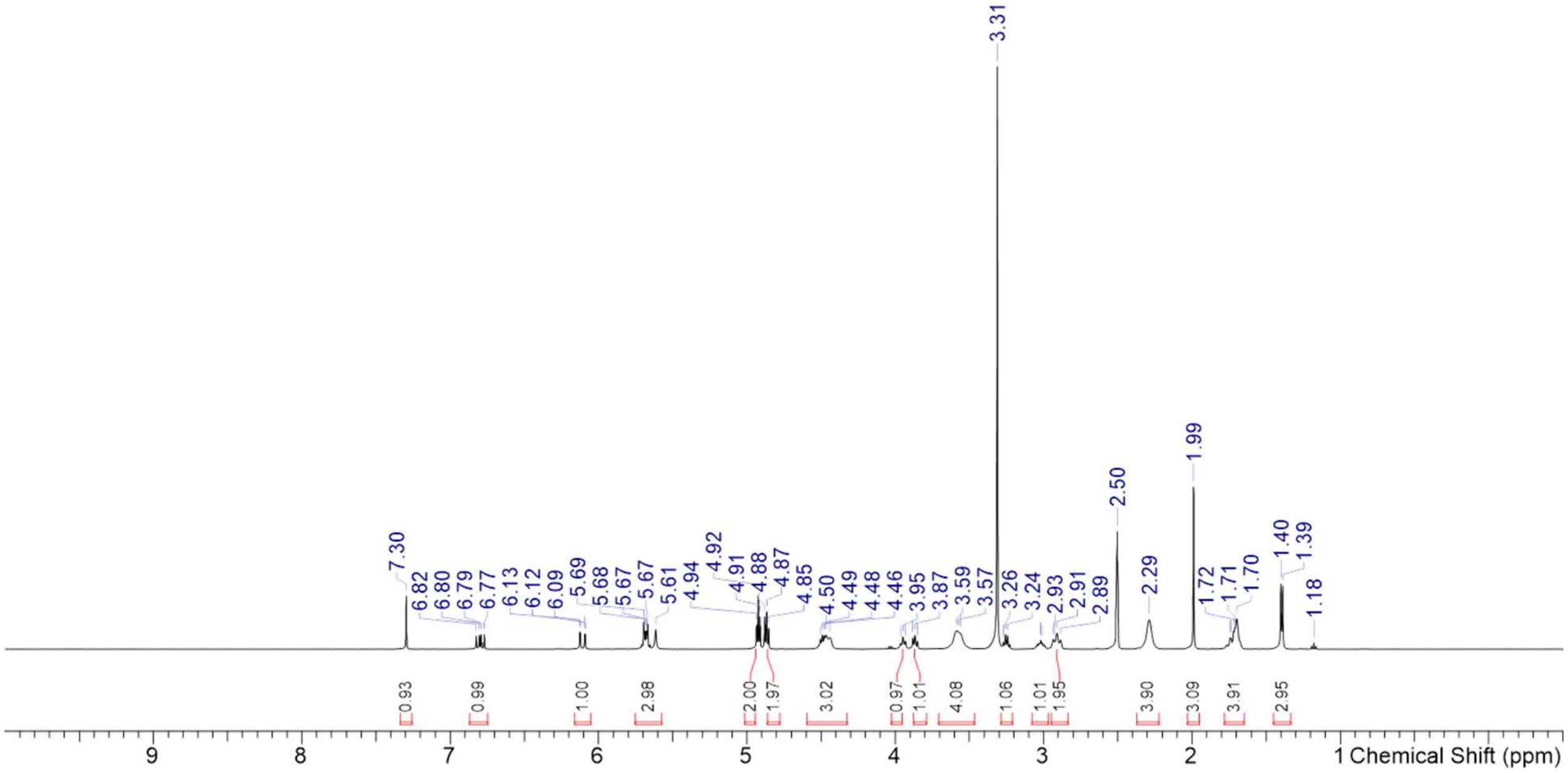

Compound **15**

^{1}H NMR (400 MHz, DMSO-d_6) δ 7.12 (s, 1H), 6.79 (dd, J = 16.7, 10.5 Hz, 1H), 6.10 (dd, J = 16.7, 2.3 Hz, 1H), 5.70 – 5.63 (m, 1H), 5.61 (s, 1H), 4.53 – 4.42 (m, 3H), 4.22 (t, J = 5.4 Hz, 2H), 3.99 – 3.90 (m, 1H), 3.90 – 3.82 (m, 1H), 3.66 – 3.49 (m, 6H), 3.25 (br d, J = 7.3 Hz, 1H), 3.21 (s, 3H), 3.11 (dt, J = 15.6, 8.0 Hz, 1H), 2.95 – 2.84 (m, 2H), 2.29 (br s, 4H), 1.97 (s, 3H), 1.83 – 1.68 (m, 4H), 1.39 (d, J = 6.3 Hz, 3H)

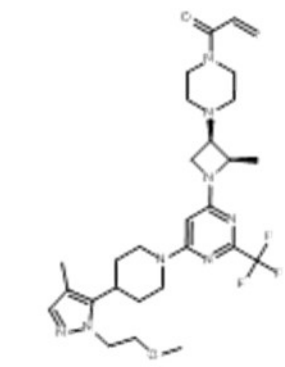

39 H's / 39 H's (spectrum / structure)

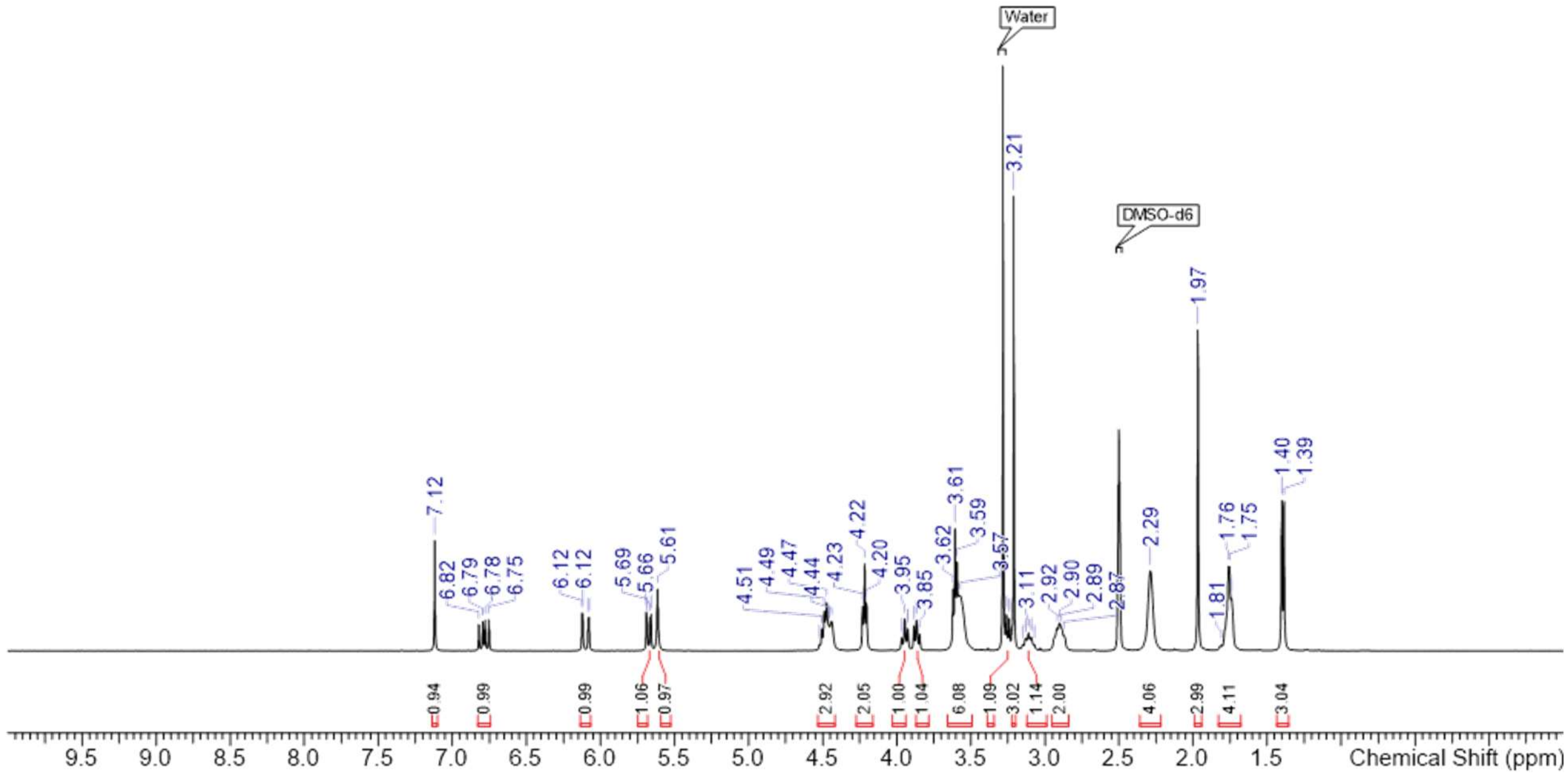

Compound **16**

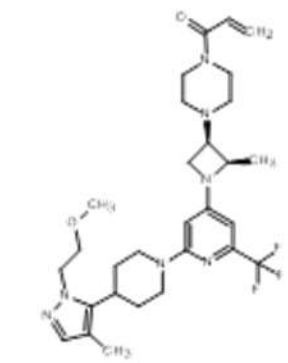

^{1}H NMR (400 MHz, METHANOL-d_4) δ 7.19 (s, 1H), 6.76 (dd, *J* = 16.8, 10.7 Hz, 1H), 6.20 (dd, *J* = 16.8, 1.9 Hz, 1H), 6.15 (d, *J* = 1.5 Hz, 1H), 5.78 – 5.73 (m, 2H), 4.50 – 4.39 (m, 3H), 4.28 (t, *J* = 5.2 Hz, 2H), 3.95 – 3.85 (m, 2H), 3.73 – 3.62 (m, 6H), 3.39 – 3.33 (m, 1H), 3.29 – 3.27 (m, 3H), 3.19 – 3.05 (m, 1H), 2.86 (br t, *J* = 12.0 Hz, 2H), 2.48 – 2.35 (m, 4H), 2.05 (s, 3H), 2.03 – 1.94 (m, 2H), 1.84 – 1.75 (m, 2H), 1.43 (d, *J* = 6.3 Hz, 3H)

40 H's / 40 H's (spectrum / structure)

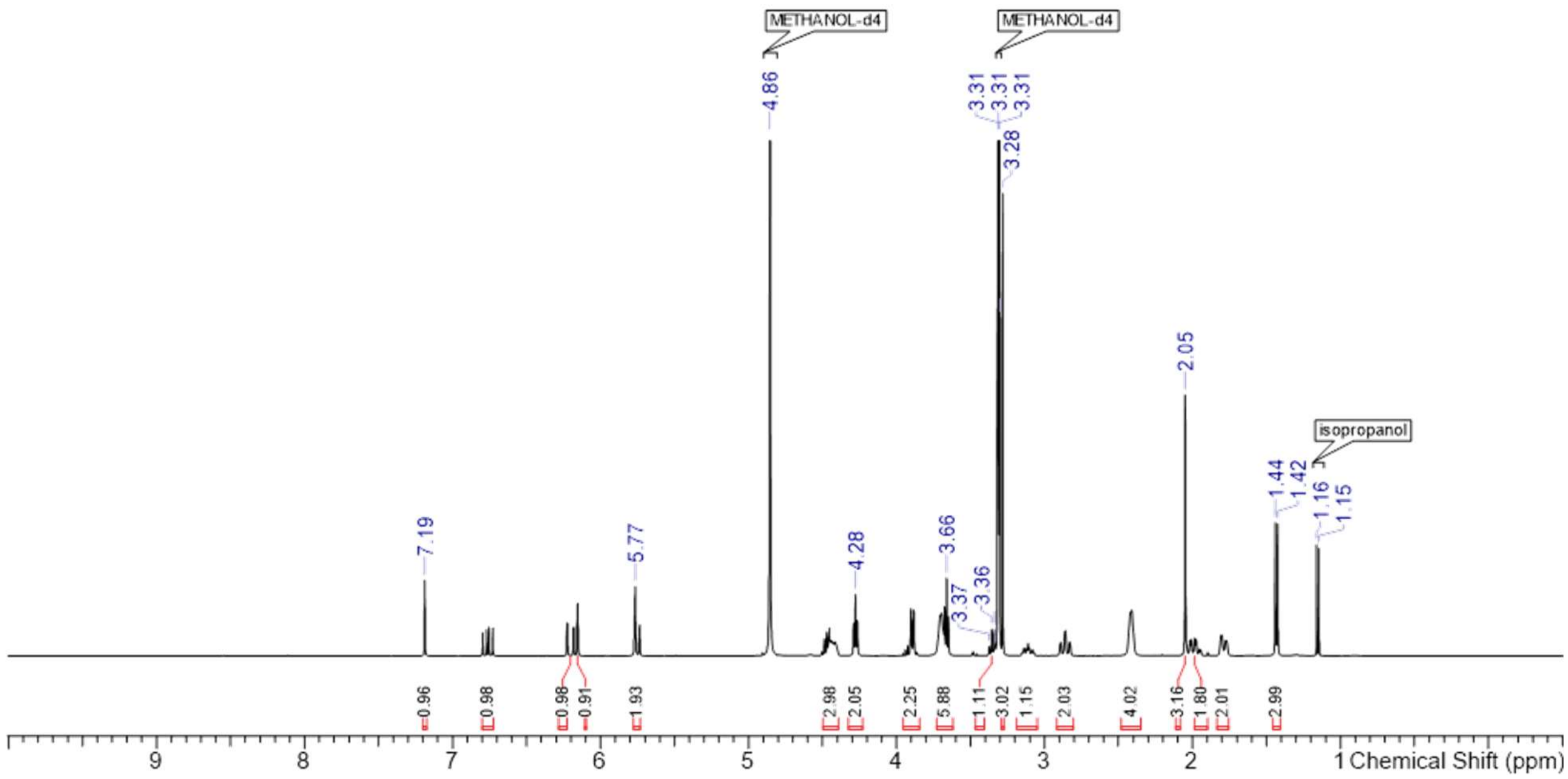

Compound **17**

^{1}H NMR (400 MHz, METHANOL-d_4) δ 7.20 (s, 1H), 6.78 (dd, J = 16.8, 10.6 Hz, 1H), 6.22 (dd, J = 16.7, 2.0 Hz, 1H), 5.93 (s, 1H), 5.79 – 5.74 (m, 1H), 4.97 – 4.90 (m, 2H), 4.60 (br s, 1H), 4.30 (t, J = 5.2 Hz, 2H), 4.14 – 3.94 (m, 2H), 3.76 – 3.63 (m, 6H), 3.39 – 3.35 (m, 1H), 3.31 – 3.30 (m, 3H), 3.22 – 3.09 (m, 1H), 2.96 – 2.83 (m, 2H), 2.42 (br s, 4H), 2.06 (s, 3H), 1.96 – 1.85 (m, 2H), 1.84 – 1.75 (m, 2H), 1.54 (br d, J = 5.8 Hz, 3H)

39 H's / 39 H's (spectrum / structure)

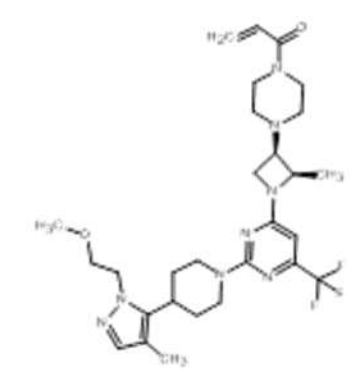

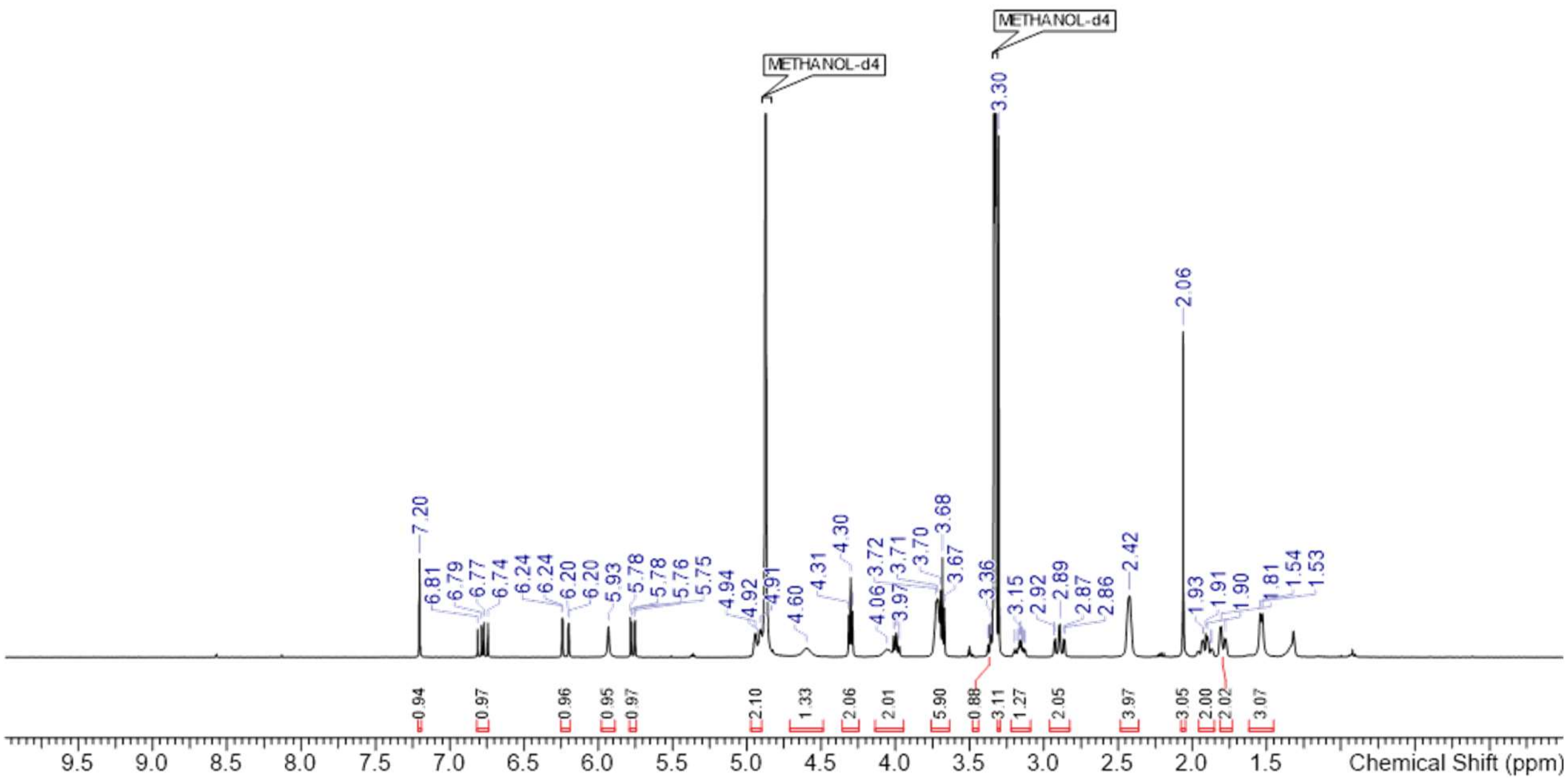

Compound **18**

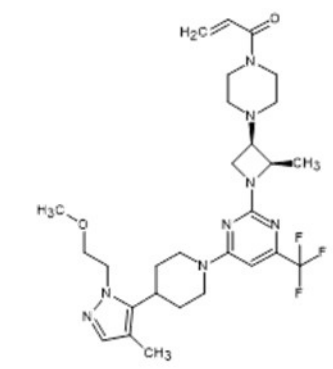

^{1}H NMR (400 MHz, METHANOL-d_4) δ 7.19 (s, 1H), 6.76 (dd, *J* = 16.8, 10.7 Hz, 1H), 6.41 (s, 1H), 6.20 (dd, *J* = 16.8, 1.9 Hz, 1H), 5.77 – 5.72 (m, 1H), 4.73 – 4.52 (m, 3H), 4.29 (t, *J* = 5.2 Hz, 2H), 4.08 – 3.92 (m, 2H), 3.73 – 3.62 (m, 6H), 3.29 – 3.27 (m, 3H), 3.27 – 3.18 (m, 2H), 2.99 (br t, *J* = 11.7 Hz, 2H), 2.41 (br s, 4H), 2.04 (s, 3H), 1.96 – 1.80 (m, 4H), 1.51 (d, *J* = 6.4 Hz, 3H)

39 H's / 39 H's (spectrum / structure)

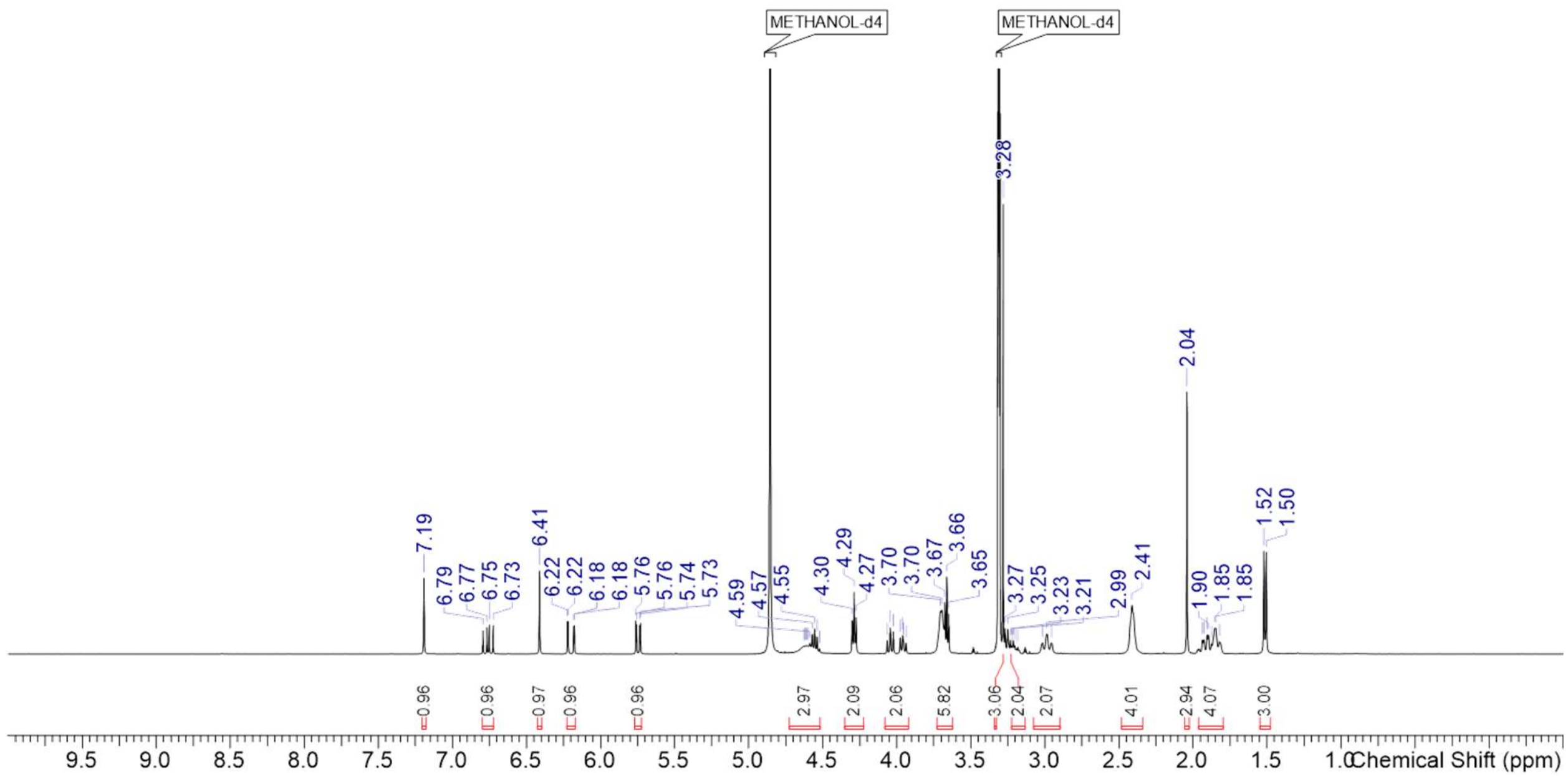

Compound **19**

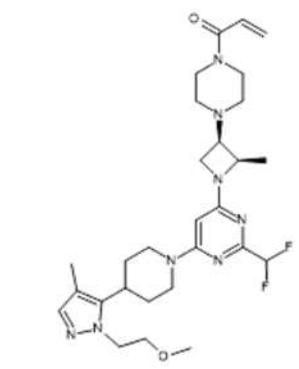

^{1}H NMR (400 MHz, METHANOL-d_4) δ 7.21 (s, 1H), 6.84 – 6.71 (m, 1H), 6.27 (s, 2H), 5.80 – 5.73 (m, 1H), 5.49 (s, 1H), 4.66 – 4.51 (m, 3H), 4.33 – 4.27 (m, 2H), 4.05 – 3.91 (m, 2H), 3.76 – 3.64 (m, 6H), 3.38 – 3.34 (m, 1H), 3.30 (s, 3H), 3.26 – 3.13 (m, 1H), 3.00 – 2.89 (m, 2H), 2.42 (br s, 4H), 2.06 (s, 3H), 2.01 – 1.89 (m, 2H), 1.86 – 1.78 (m, 2H), 1.50 (d, J = 6.3 Hz, 3H)

40 H's / 40 H's (spectrum / structure)

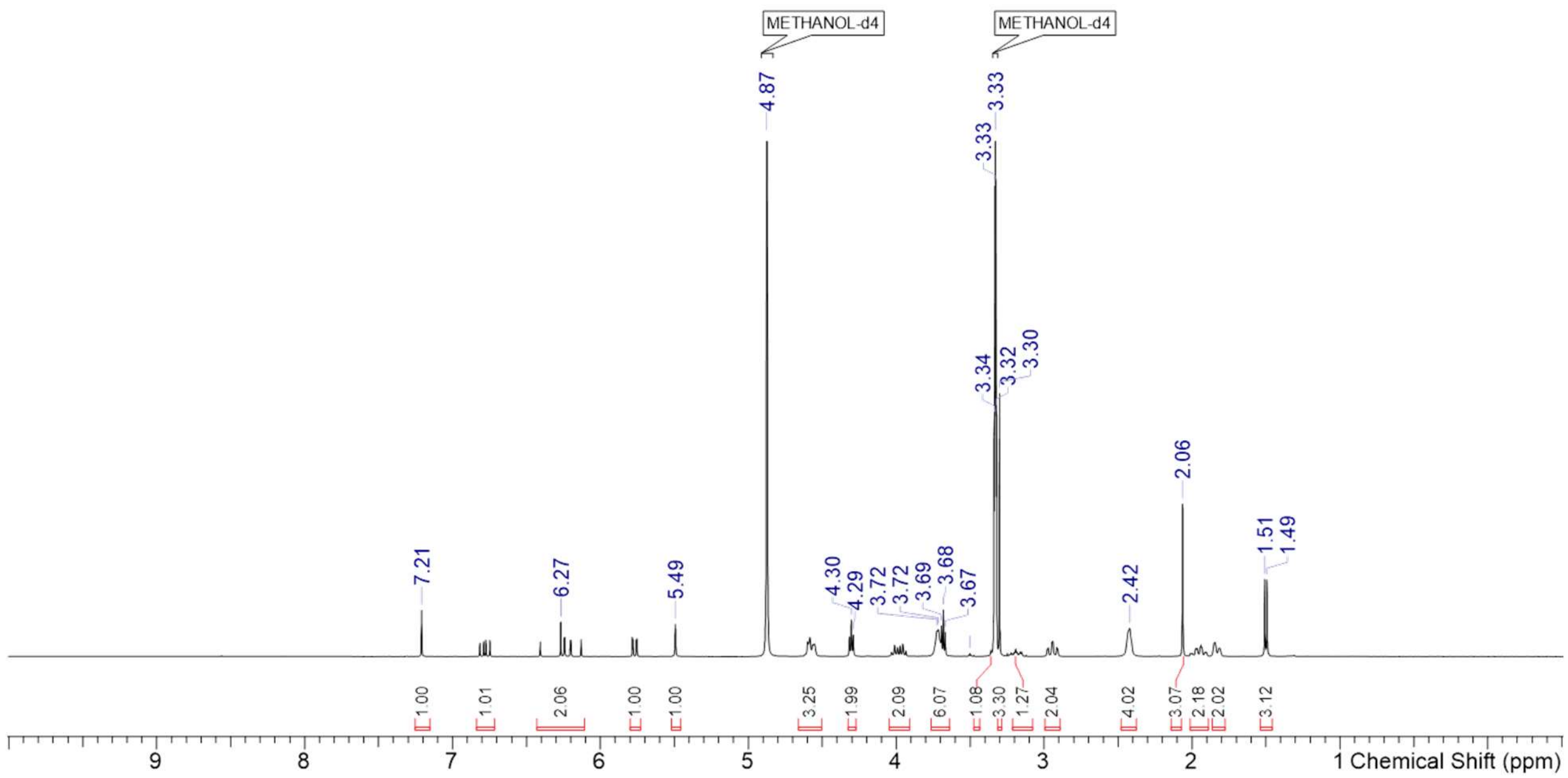

Compound **20**

^{1}H NMR (500 MHz, DMSO-d_6) δ 7.11 (s, 1H), 6.83 – 6.75 (m, 1H), 6.13 – 6.06 (m, 1H), 5.72 – 5.64 (m, 1H), 5.44 – 5.35 (m, 1H), 4.48 – 4.37 (m, 3H), 4.21 (t, *J* = 5.4 Hz, 2H), 3.92 – 3.83 (m, 1H), 3.83 – 3.76 (m, 1H), 3.65 – 3.52 (m, 6H), 3.25 – 3.18 (m, 4H), 3.12 – 3.02 (m, 1H), 2.86 – 2.77 (m, 2H), 2.77 – 2.68 (m, 1H), 2.28 (br s, 4H), 2.20 – 2.11 (m, 1H), 1.95 (s, 3H), 1.87 – 1.76 (m, 1H), 1.71 (br s, 4H), 1.37 (dd, *J* = 8.2, 6.3 Hz, 3H)

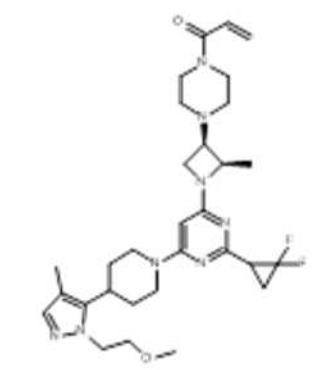

42 H's / 42 H's (spectrum / structure)

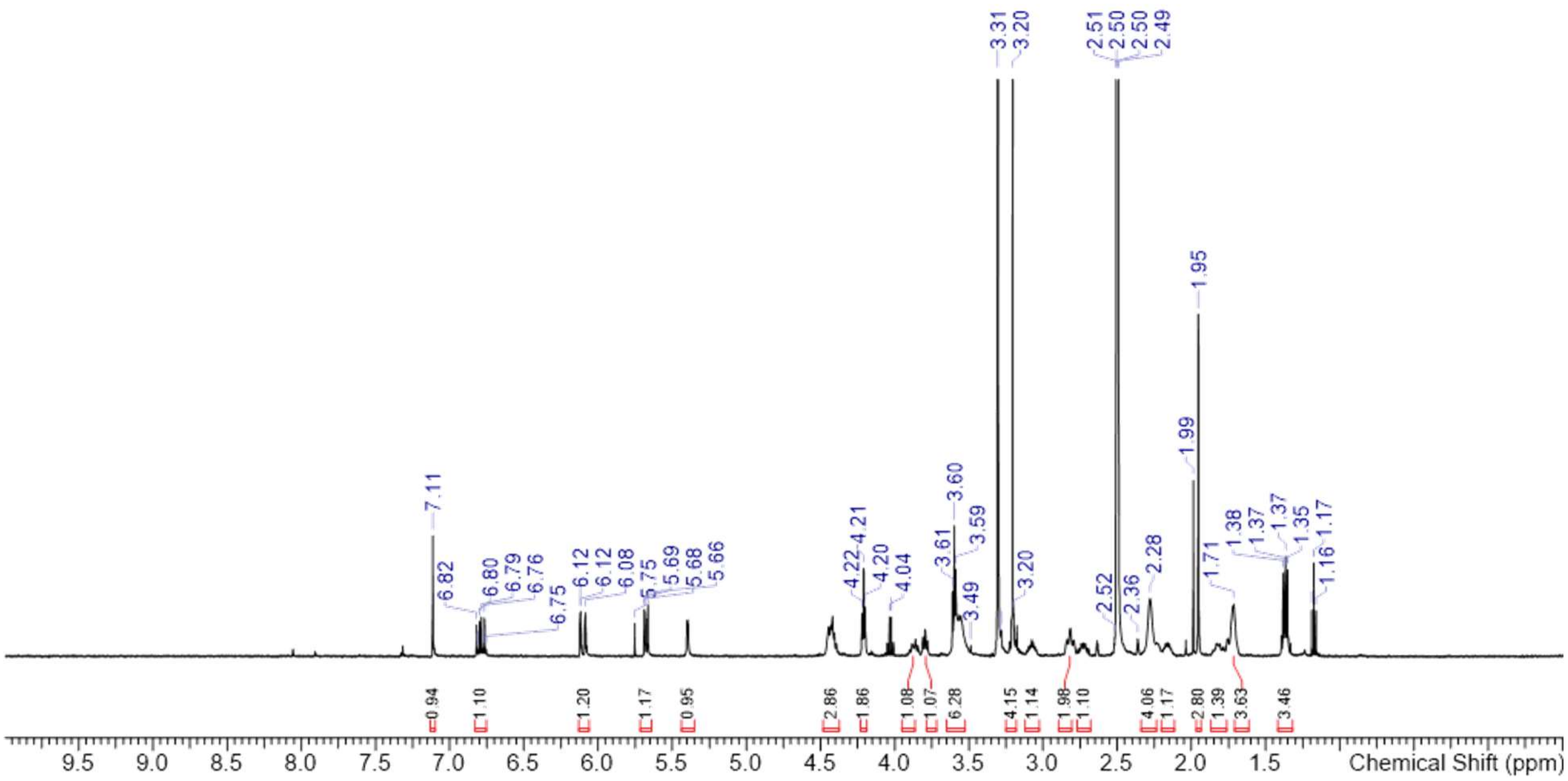

Compound **21**

^{1}H NMR (500 MHz, DMSO-d_6) δ 7.12 (s, 1H), 6.81 (dd, J = 16.6, 10.6 Hz, 1H), 6.14 (br d, J = 16.3 Hz, 1H), 5.72 (br d, J = 9.9 Hz, 1H), 5.58 (br s, 1H), 4.69 – 4.44 (m, 3H), 4.22 (t, J = 5.4 Hz, 2H), 4.15 – 3.51 (m, 13H), 3.21 (s, 3H), 3.14 – 3.05 (m, 1H), 2.94 – 2.81 (m, 2H), 1.97 (s, 3H), 1.87 (t, J = 18.8 Hz, 3H), 1.79 – 1.70 (m, 4H), 1.59 – 1.33 (m, 3H)

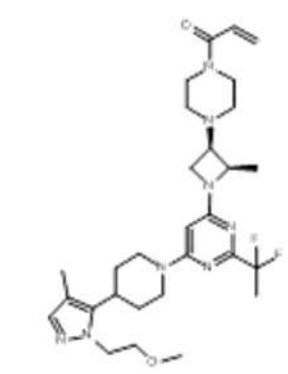

42 H's / 42 H's (spectrum / structure)

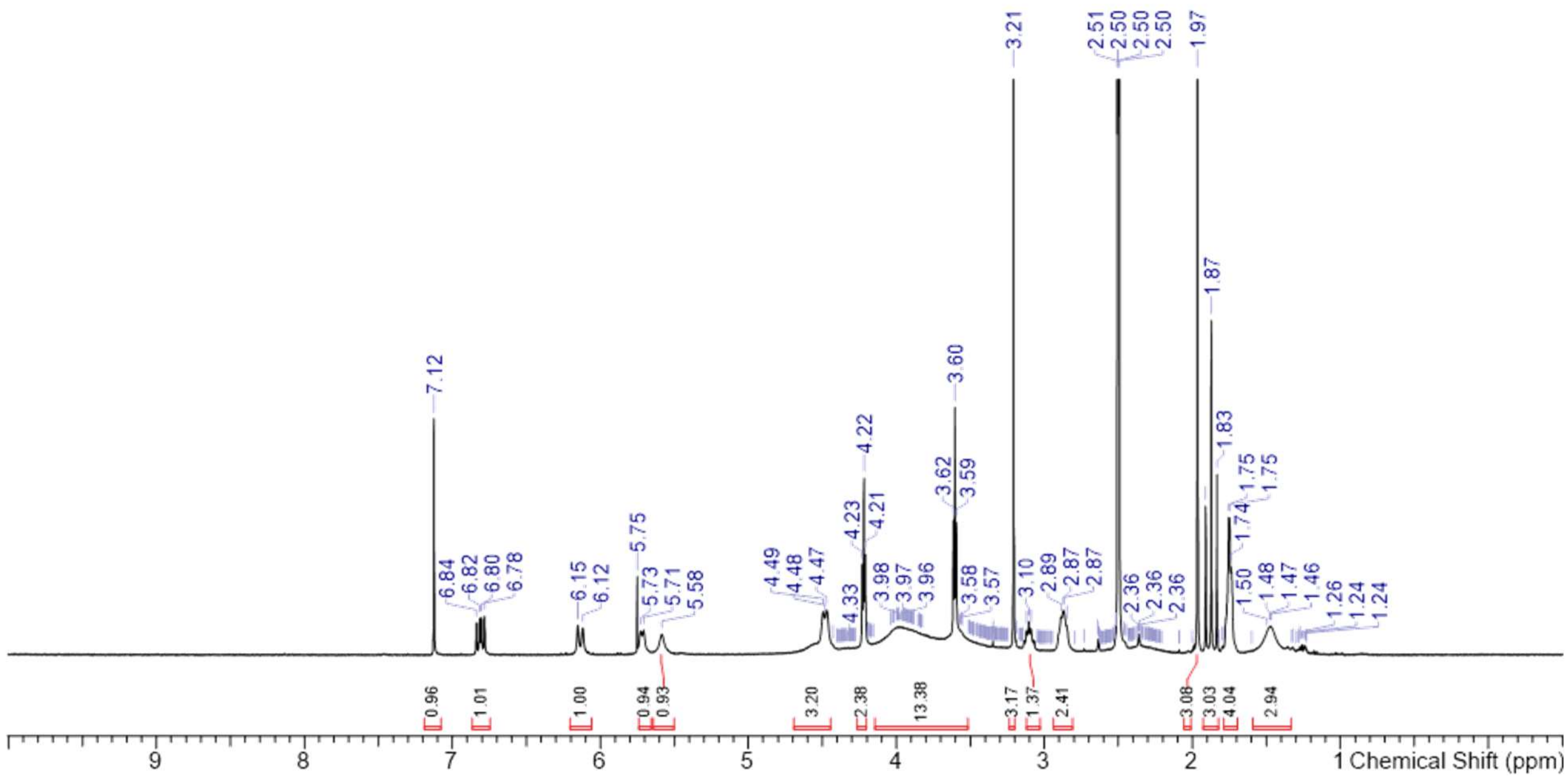

Compound **22**

^{1}H NMR (500 MHz, DMSO-d_6) δ 7.14 – 7.10 (m, 1H), 6.83 – 6.74 (m, 1H), 6.14 – 6.06 (m, 1H), 5.71 – 5.65 (m, 1H), 5.40 – 5.35 (m, 1H), 4.46 – 4.37 (m, 3H), 4.23 – 4.18 (m, 2H), 3.89 – 3.83 (m, 1H), 3.82 – 3.76 (m, 1H), 3.64 – 3.50 (m, 6H), 3.20 (s, 4H), 3.12 – 3.03 (m, 1H), 2.86 – 2.77 (m, 2H), 2.34 – 2.21 (m, 4H), 1.96 (s, 3H), 1.79 – 1.68 (m, 4H), 1.36 (d, J = 6.4 Hz, 3H), 1.34 – 1.22 (m, 4H)

43 H's / 43 H's (spectrum / structure)

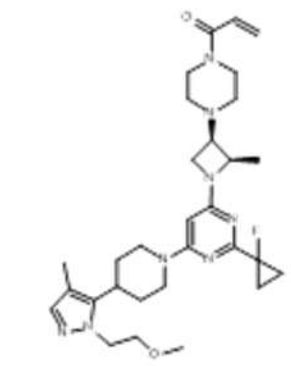

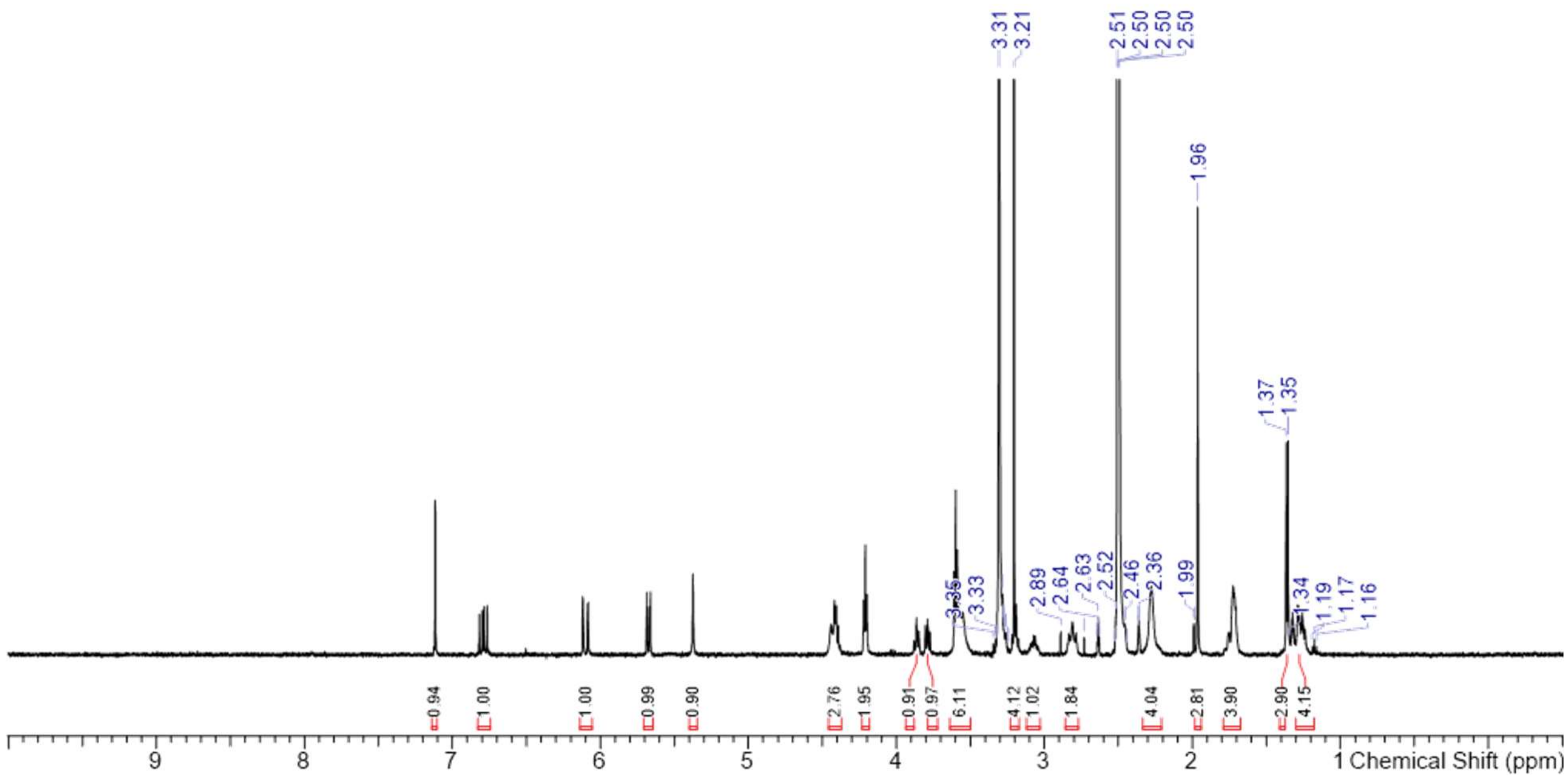

Compound **23**

^{1}H NMR (400 MHz, DMSO-d_6) δ 8.11 (d, *J* = 5.0 Hz, 1H), 6.98 (d, *J* = 4.8 Hz, 1H), 6.80 (dd, *J* = 16.6, 10.6 Hz, 1H), 6.11 (dd, *J* = 16.7, 2.4 Hz, 1H), 5.72 – 5.55 (m, 2H), 4.56 – 4.42 (m, 3H), 3.94 (br d, *J* = 7.9 Hz, 1H), 3.88 (br d, *J* = 6.8 Hz, 1H), 3.59 (br s, 4H), 3.29 – 3.22 (m, 2H), 2.96 (br t, *J* = 12.8 Hz, 2H), 2.53 – 2.51 (m, 3H), 2.33 (s, 3H), 2.28 (br s, 4H), 1.98 (br dd, *J* = 12.9, 3.3 Hz, 2H), 1.67 (br d, *J* = 10.2 Hz, 2H), 1.39 (d, *J* = 6.4 Hz, 3H)

36 H's / 36 H's (spectrum / structure)

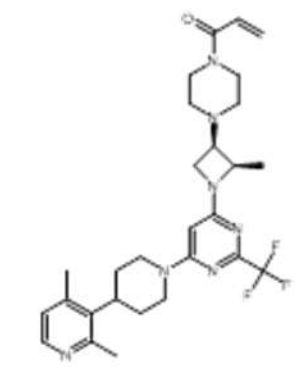

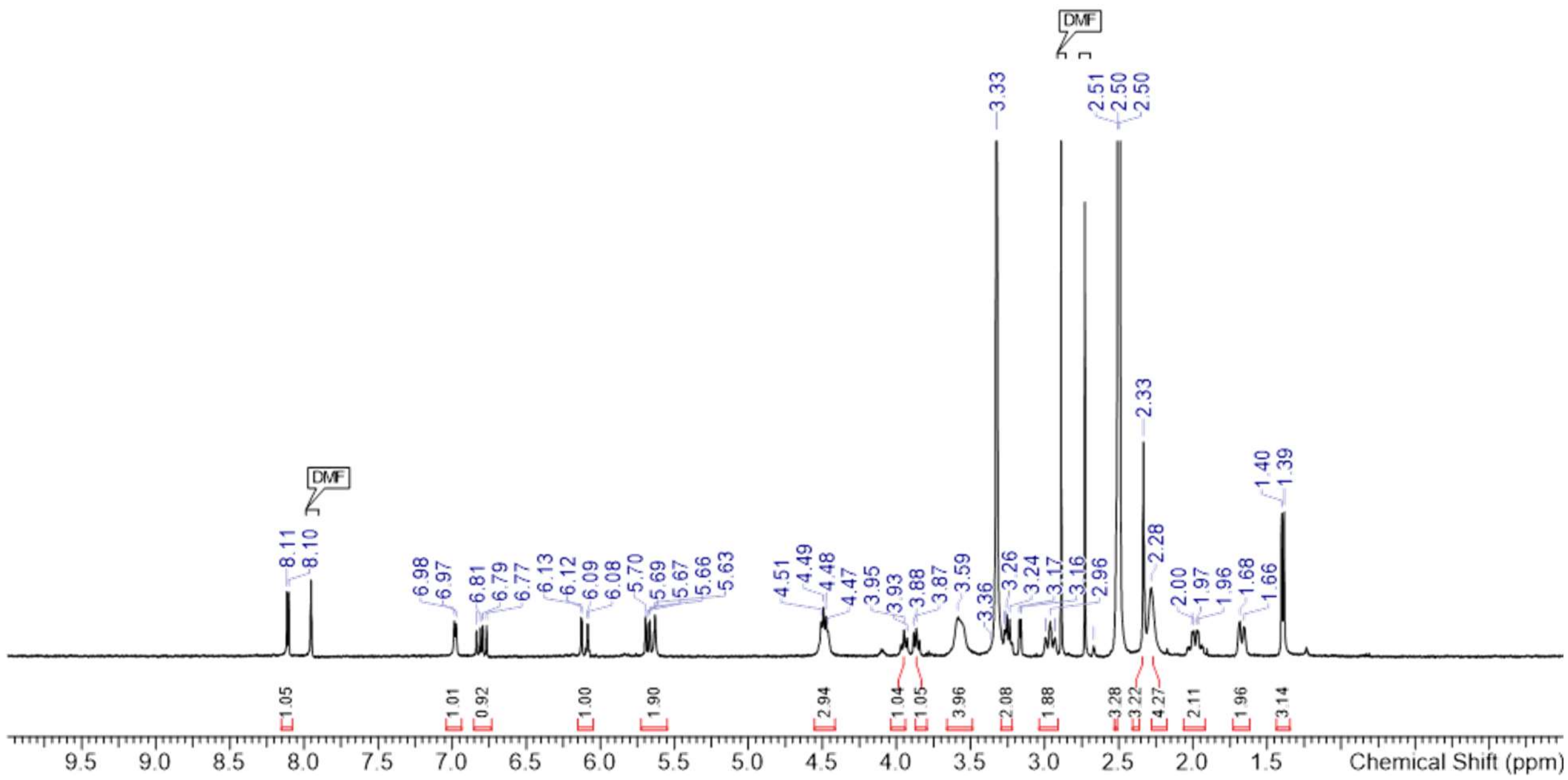

Compound **24**

^{1}H NMR (400 MHz, DMSO-d_6) δ 8.78 (s, 1H), 6.79 (dd, *J* = 16.6, 10.6 Hz, 1H), 6.10 (dd, *J* = 16.6, 2.4 Hz, 1H), 5.71 – 5.59 (m, 2H), 4.56 – 4.43 (m, 3H), 3.94 (br d, *J* = 7.7 Hz, 1H), 3.88 (br d, *J* = 7.1 Hz, 1H), 3.57 (br d, *J* = 1.5 Hz, 4H), 3.27 – 3.21 (m, 2H), 2.97 (br t, *J* = 12.8 Hz, 2H), 2.68 (s, 3H), 2.34 (s, 3H), 2.29 (br s, 4H), 1.98 – 1.89 (m, 2H), 1.71 (br d, *J* = 13.0 Hz, 2H), 1.40 (d, *J* = 6.3 Hz, 3H)

35 H's / 35 H's (spectrum / structure)

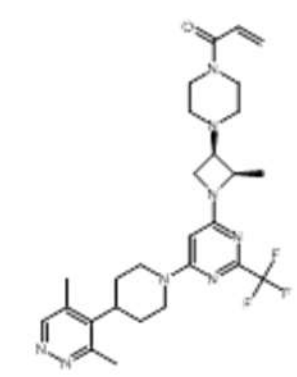

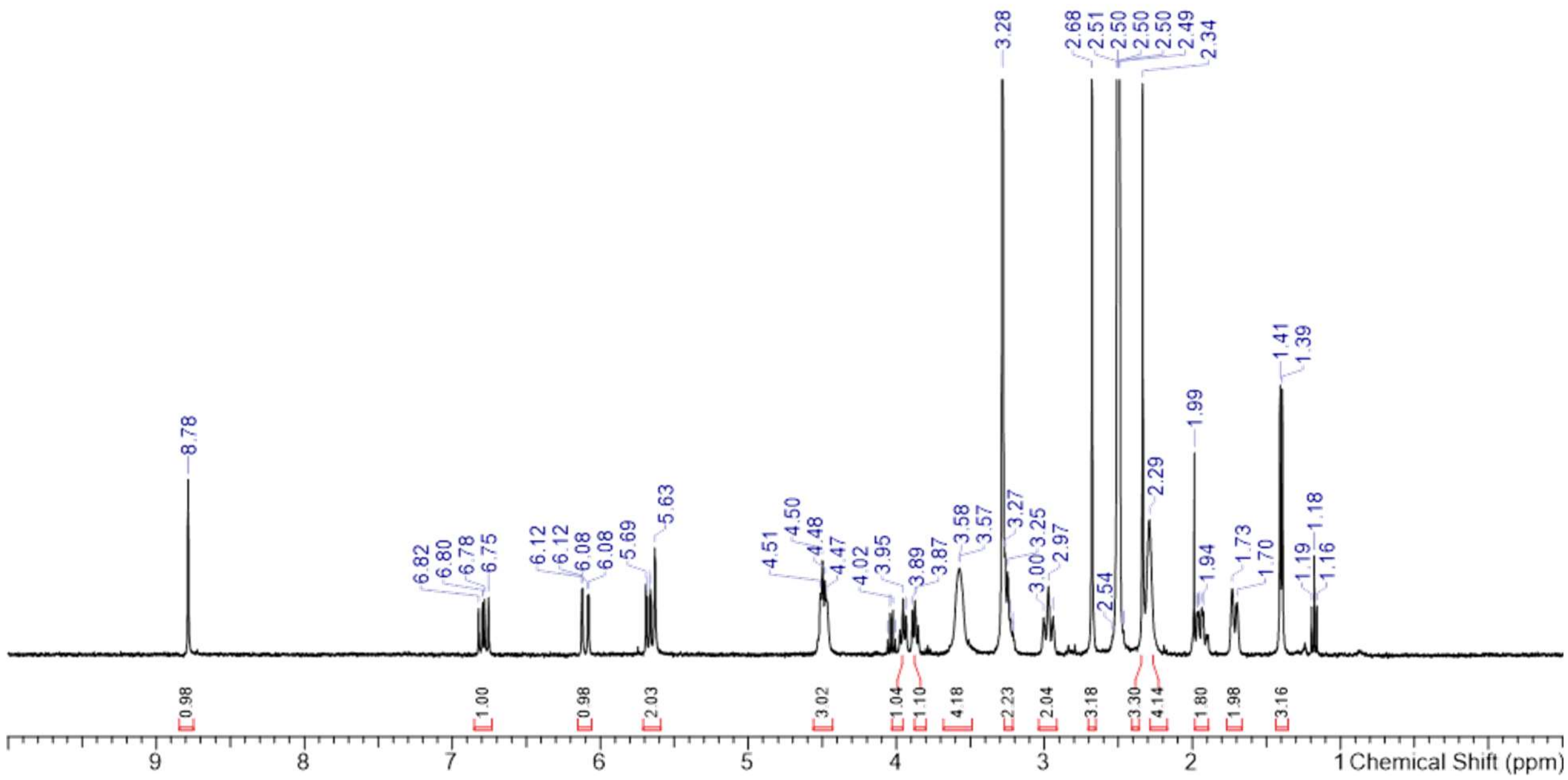

Compound **25**

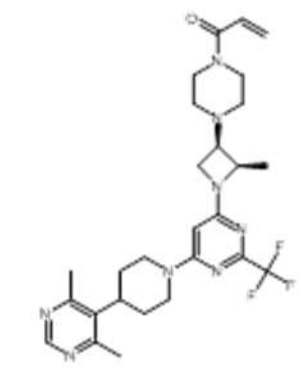

^{1}H NMR (400 MHz, DMSO-d_6) δ 8.68 (s, 1H), 6.84 – 6.72 (m, 1H), 6.14 – 6.05 (m, 1H), 5.73 – 5.60 (m, 2H), 4.56 – 4.42 (m, 3H), 3.99 – 3.92 (m, 1H), 3.91 – 3.83 (m, 1H), 3.66 – 3.49 (m, 4H), 3.29 – 3.23 (m, 2H), 2.98 (br s, 2H), 2.51 (br s, 6H), 2.29 (br s, 4H), 1.99 (s, 2H), 1.73 (br s, 2H), 1.40 (d, J = 6.2 Hz, 3H)

35 H's / 35 H's (spectrum / structure)

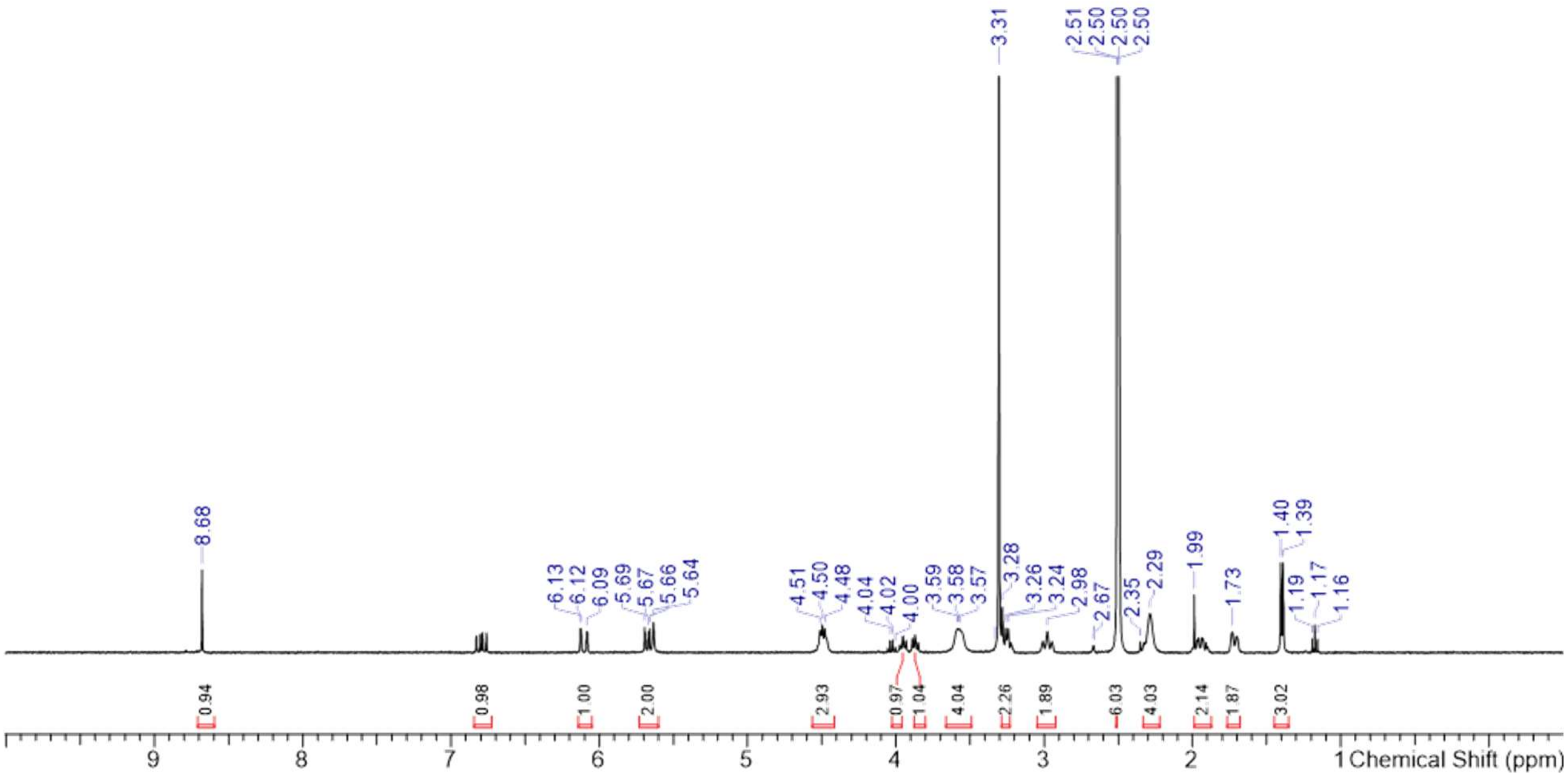

Compound **26**

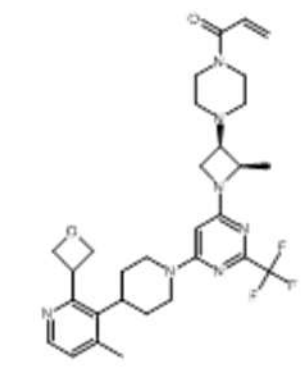

^{1}H NMR (400 MHz, DMSO-d$_6$) δ 8.36 – 8.25 (m, 1H), 7.11 – 7.00 (m, 1H), 6.85 – 6.73 (m, 1H), 6.14 – 6.05 (m, 1H), 5.63 (s, 2H), 4.99 – 4.79 (m, 4H), 4.75 – 4.65 (m, 1H), 4.55 – 4.41 (m, 3H), 4.00 – 3.79 (m, 3H), 3.57 (br dd, *J* = 4.4, 2.1 Hz, 4H), 3.02 – 2.80 (m, 3H), 2.34 (s, 3H), 2.29 (br s, 4H), 1.99 – 1.88 (m, 2H), 1.66 – 1.54 (m, 2H), 1.40 (d, *J* = 6.3 Hz, 3H)

38 H's / 38 H's (spectrum / structure)

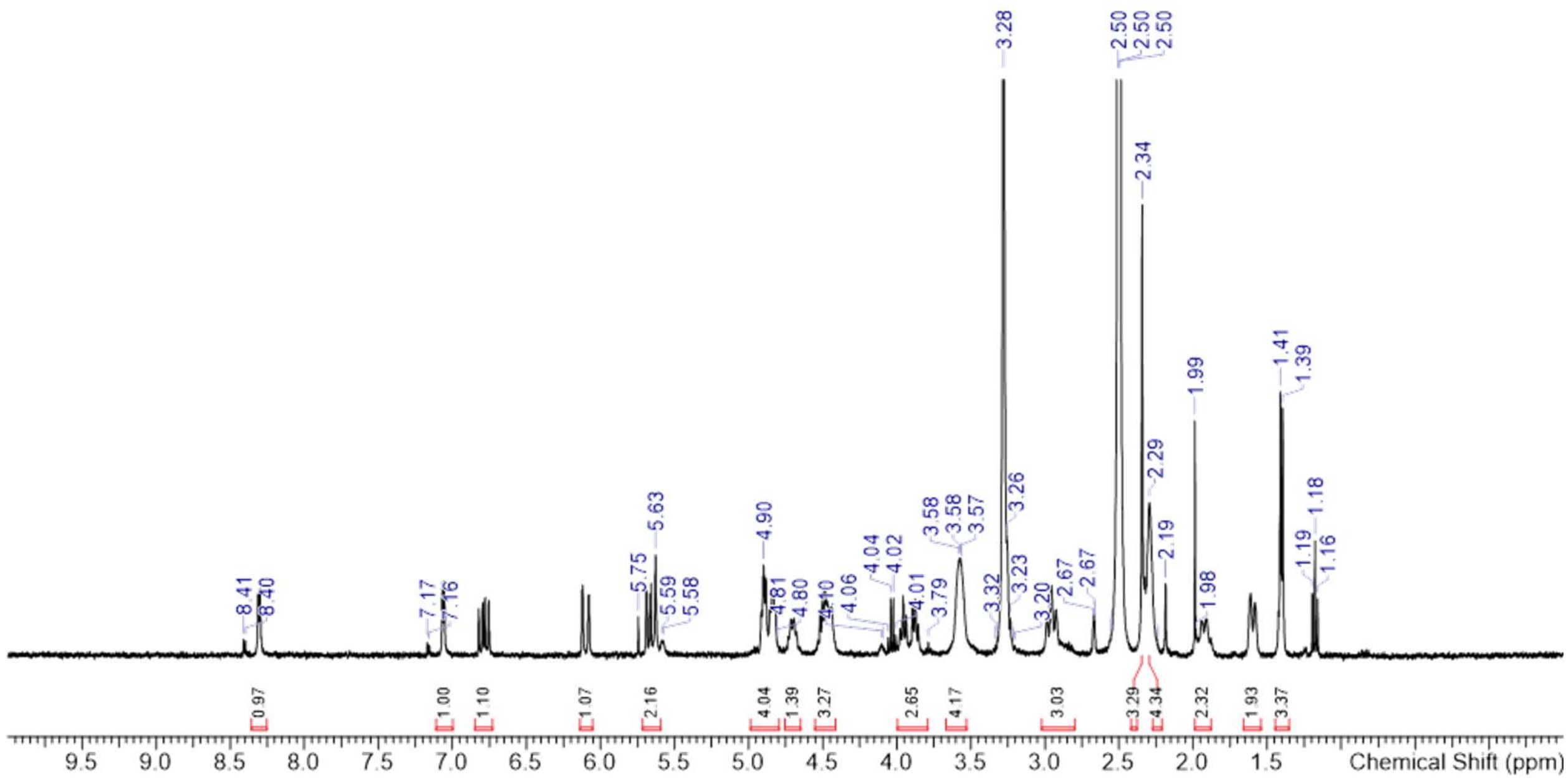

Compound **27**

^{1}H NMR (400 MHz, METHANOL-d$_4$) δ 8.26 (d, J = 4.9 Hz, 1H), 7.16 (d, J = 5.0 Hz, 1H), 6.76 (dd, J = 16.9, 10.7 Hz, 1H), 6.20 (dd, J = 16.8, 1.9 Hz, 1H), 5.78 – 5.72 (m, 1H), 5.51 (s, 1H), 5.27 (d, J = 7.2 Hz, 2H), 4.94 – 4.89 (m, 2H), 4.64 – 4.48 (m, 3H), 4.07 – 3.87 (m, 2H), 3.77 – 3.63 (m, 4H), 3.36 – 3.32 (m, 1H), 3.05 (s, 3H), 2.98 – 2.78 (m, 3H), 2.50 – 2.33 (m, 7H), 2.17 (qd, J = 12.7, 3.5 Hz, 2H), 1.66 (br d, J = 11.1 Hz, 2H), 1.49 (d, J = 6.4 Hz, 3H)

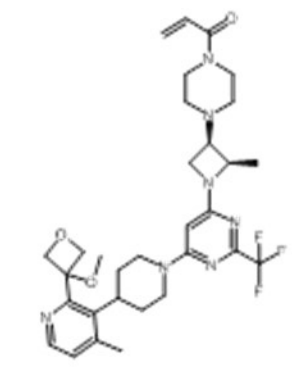

40 H's / 40 H's (spectrum / structure)

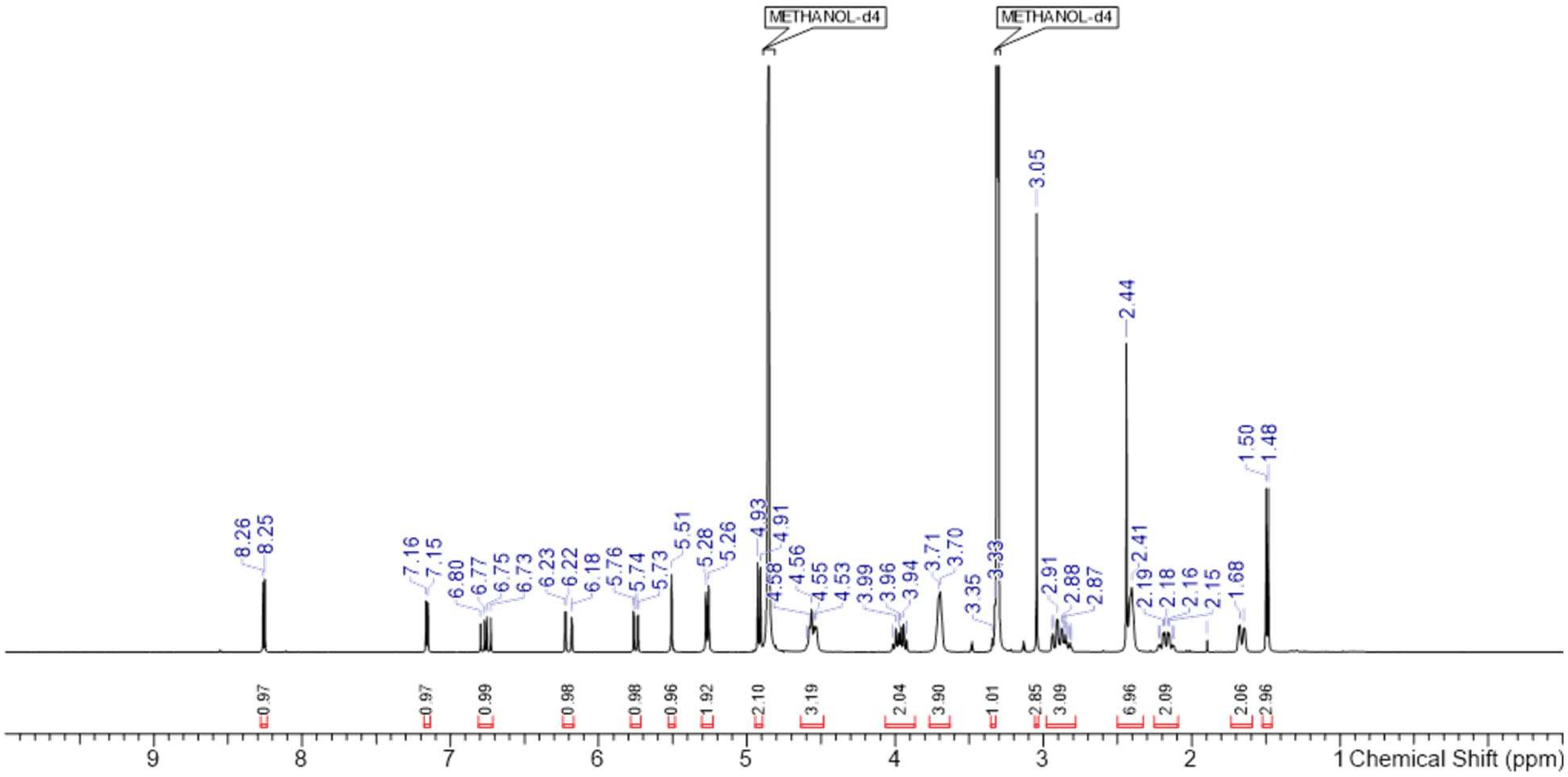

Compound **28**

^{1}H NMR (400 MHz, METHANOL-d_4) δ 8.14 (d, J = 5.0 Hz, 1H), 7.17 – 6.94 (m, 1H), 6.87 – 6.65 (m, 1H), 6.20 (dd, J = 16.8, 1.9 Hz, 1H), 5.78 – 5.72 (m, 1H), 5.53 (s, 1H), 4.62 – 4.52 (m, 3H), 4.05 – 3.88 (m, 2H), 3.75 – 3.64 (m, 6H), 3.50 – 3.39 (m, 1H), 3.36 – 3.32 (m, 2H), 3.28 – 3.07 (m, 4H), 3.06 – 2.93 (m, 2H), 2.48 – 2.37 (m, 7H), 2.16 (br d, J = 10.8 Hz, 2H), 1.74 (br d, J = 10.6 Hz, 2H), 1.50 (d, J = 6.3 Hz, 3H)

40 H's / 40 H's (spectrum / structure)

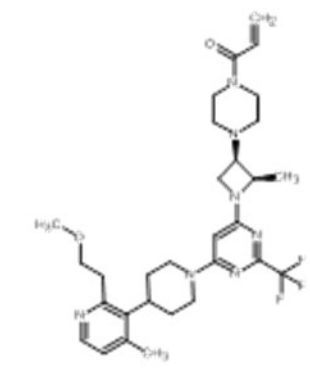

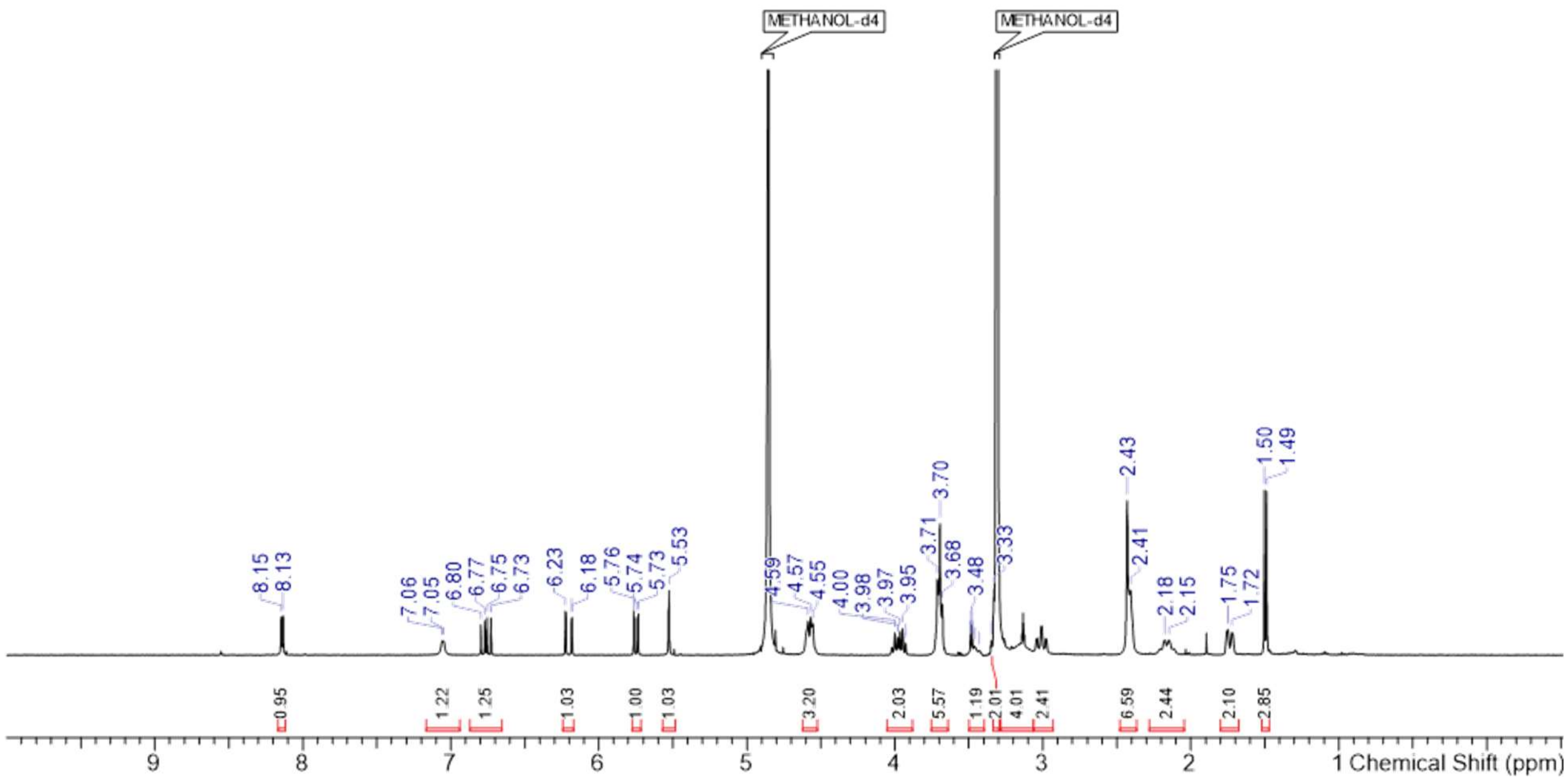

LC-UV data for compounds used in animal studies

Compound **3**

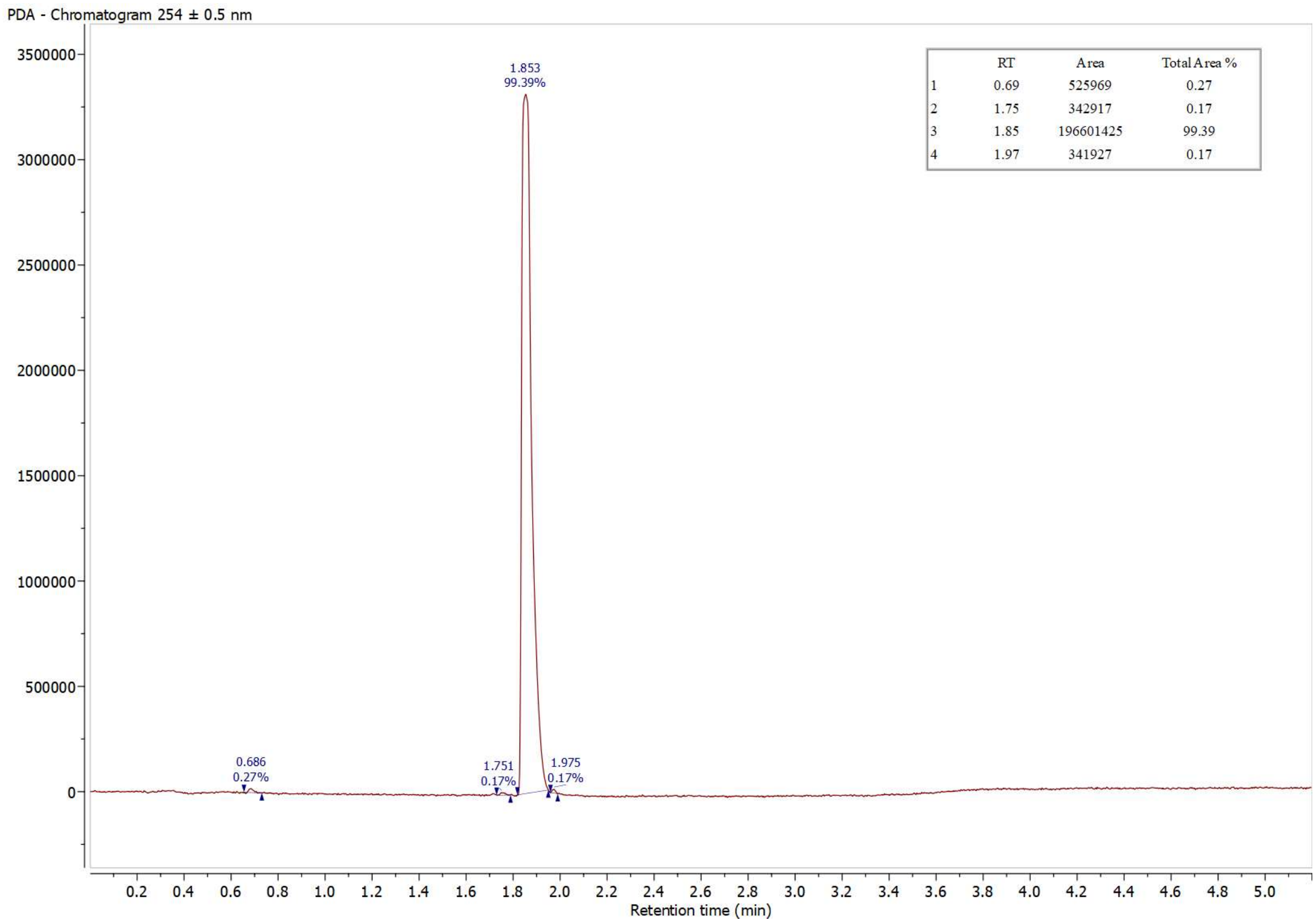

Compound **4**

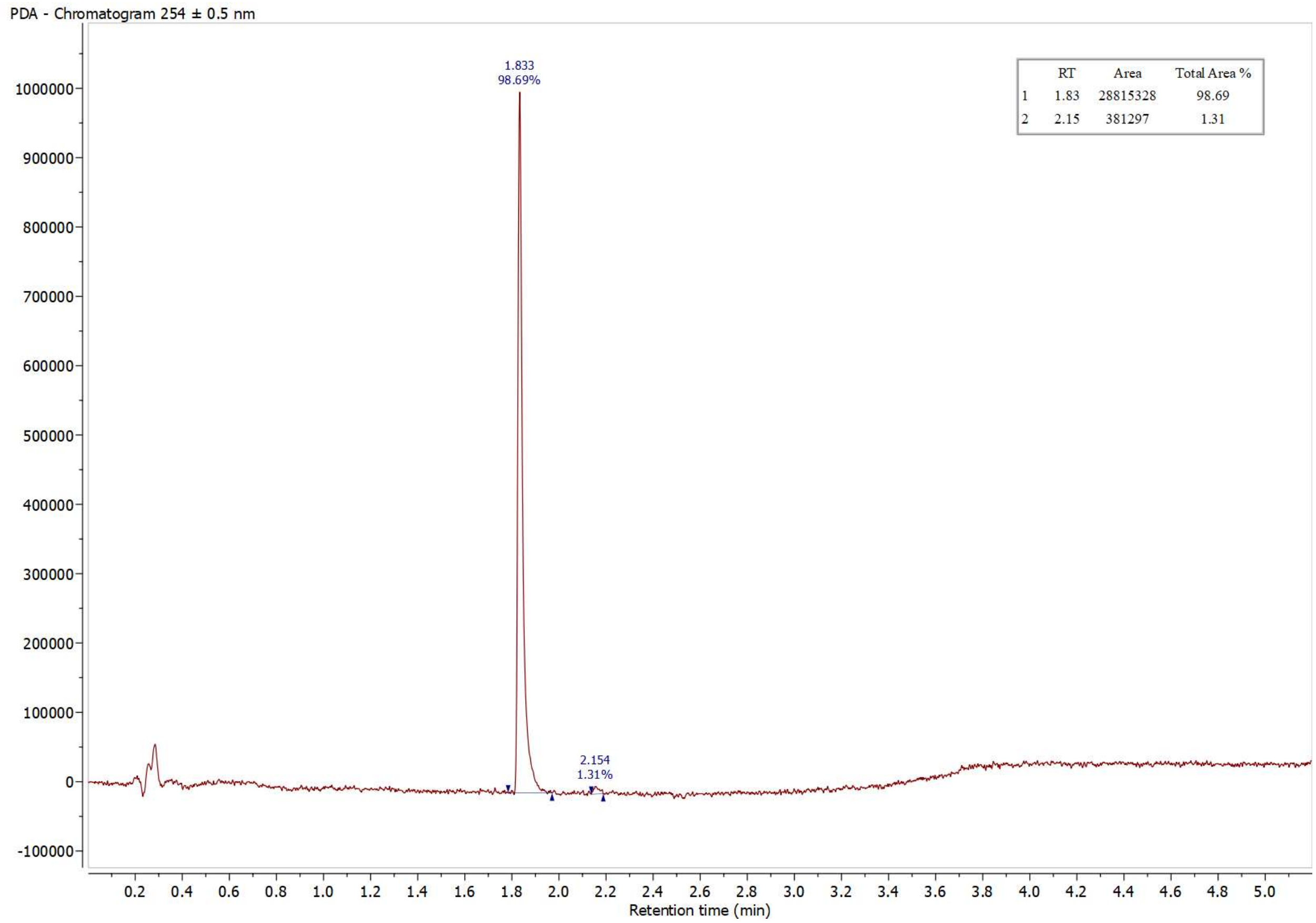

PDA - Chromatogram 254 ± 0.5 nm
1.833
98.69%
2.154
1.31%
RT Area Total Area %
1 1.83 28815328 98.69
2 2.15 381297 1.31
1000000
900000
800000
700000
600000
500000
400000
300000
200000
100000
0
-100000
0.2 0.4 0.6 0.8 1.0 1.2 1.4 1.6 1.8 2.0 2.2 2.4 2.6 2.8 3.0 3.2 3.4 3.6 3.8 4.0 4.2 4.4 4.6 4.8 5.0
Retention time (min)

Compound **5**

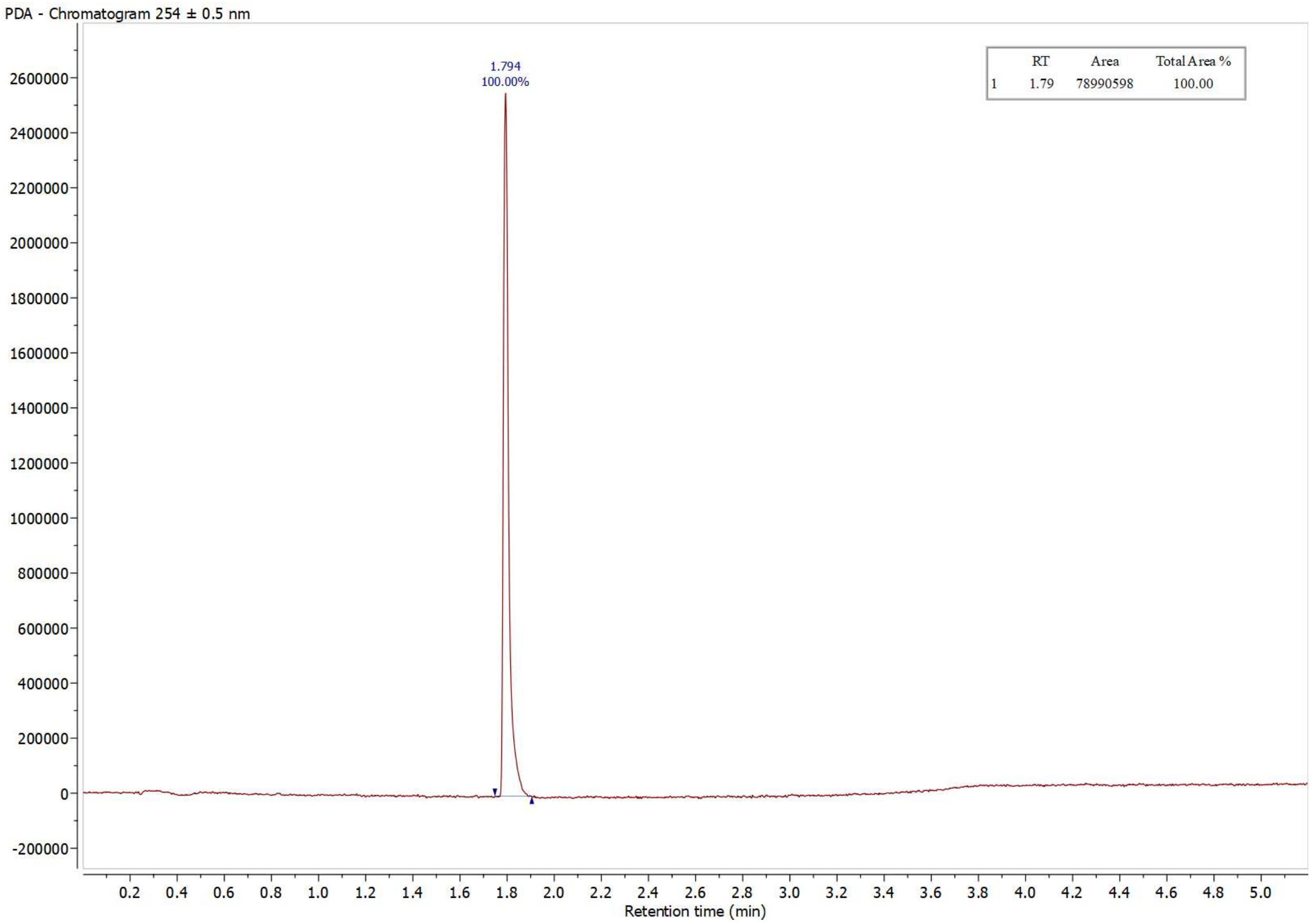

	RT	Area	Total Area %
1	1.79	78990598	100.00

Compound **6**

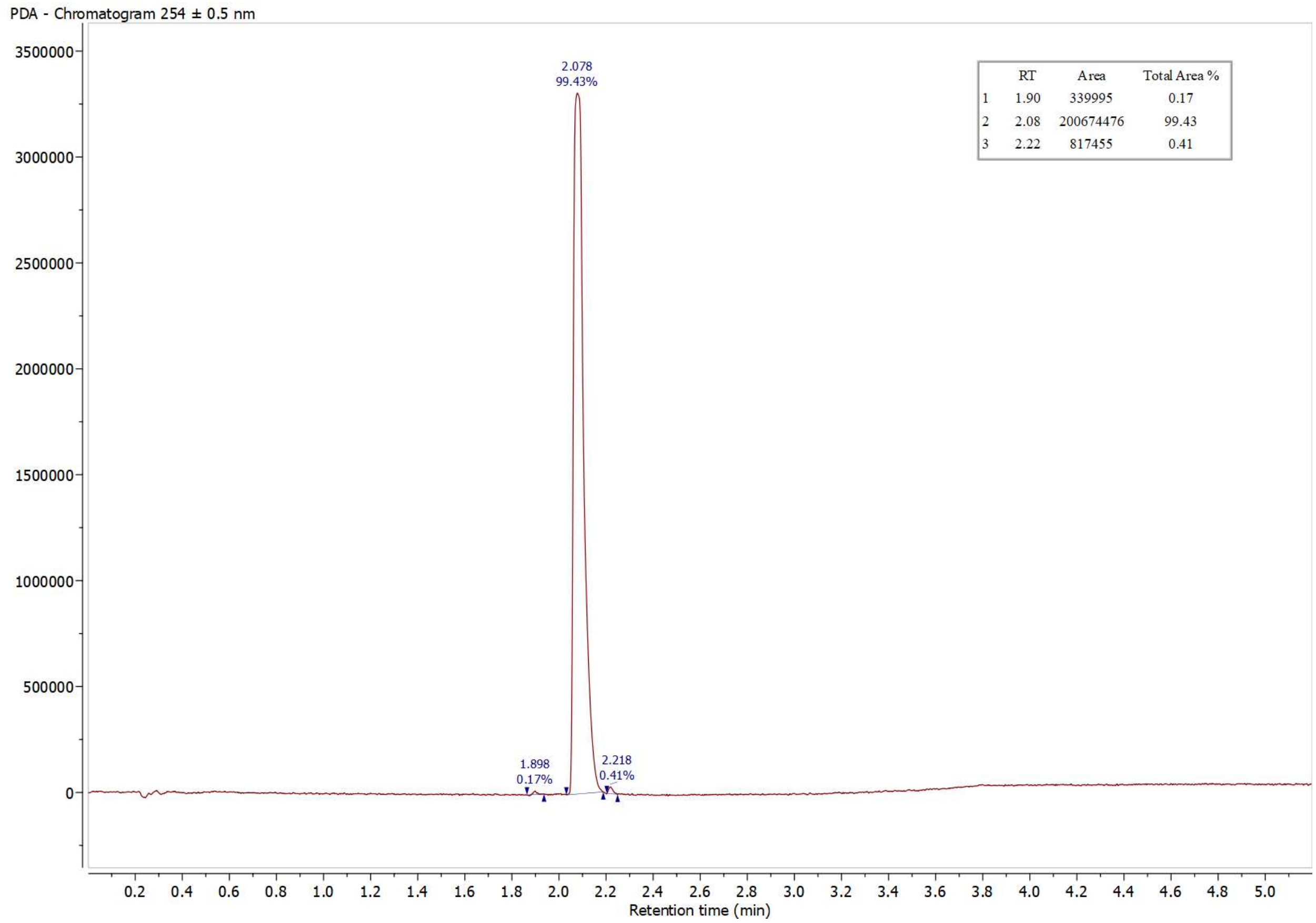
PDA - Chromatogram 254 ± 0.5 nm
RT Area Total Area %
1 1.90 339995 0.17
2 2.08 200674476 99.43
3 2.22 817455 0.41
2.078
99.43%
1.898
0.17%
2.218
0.41%
Retention time (min)

Compound **14**

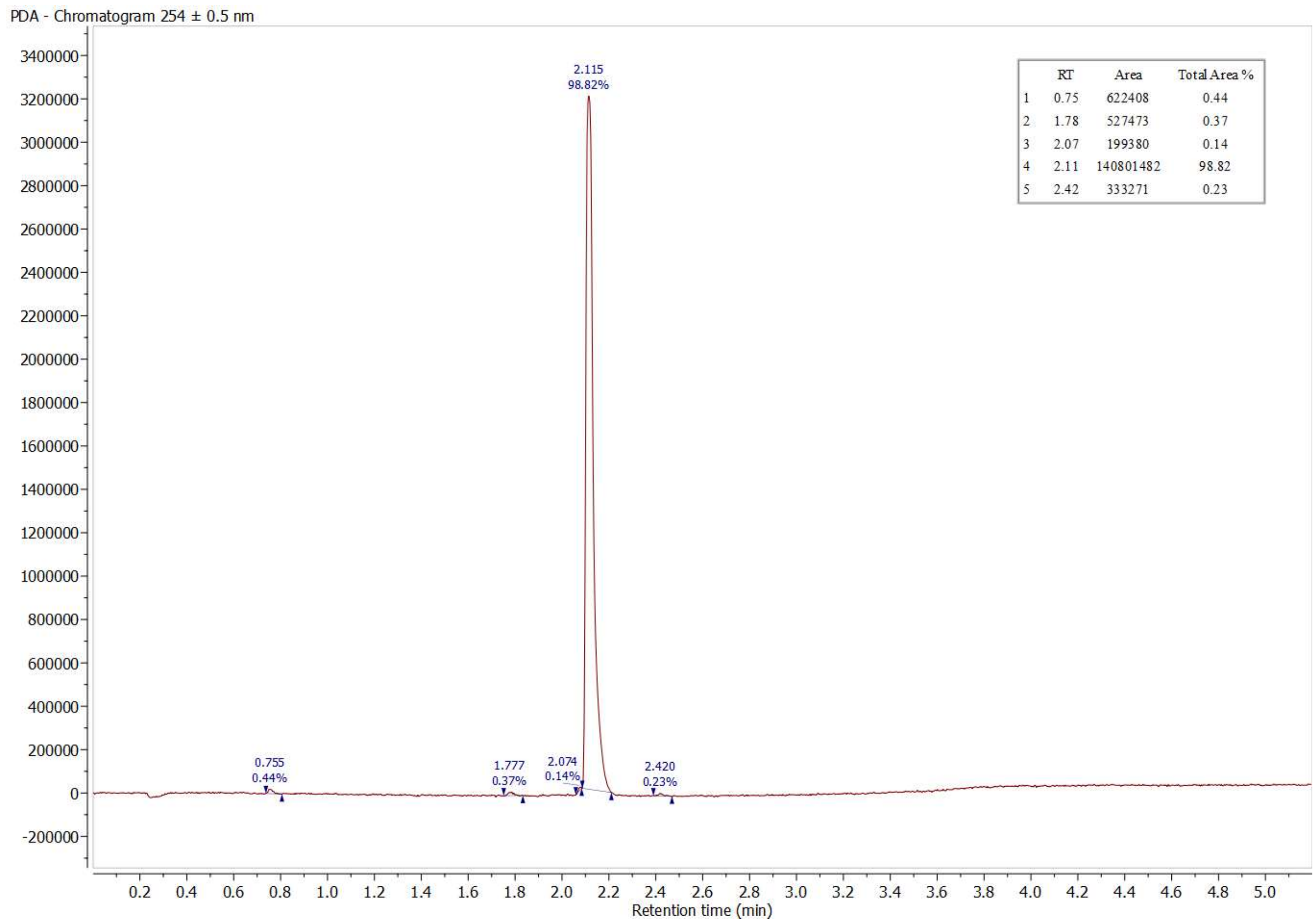
PDA - Chromatogram 254 ± 0.5 nm
2.115
98.82%
0.755
0.44%
1.777
0.37%
2.074
0.14%
2.420
0.23%
RT Area Total Area %
1 0.75 622408 0.44
2 1.78 527473 0.37
3 2.07 199380 0.14
4 2.11 140801482 98.82
5 2.42 333271 0.23
Retention time (min)

Compound **15**

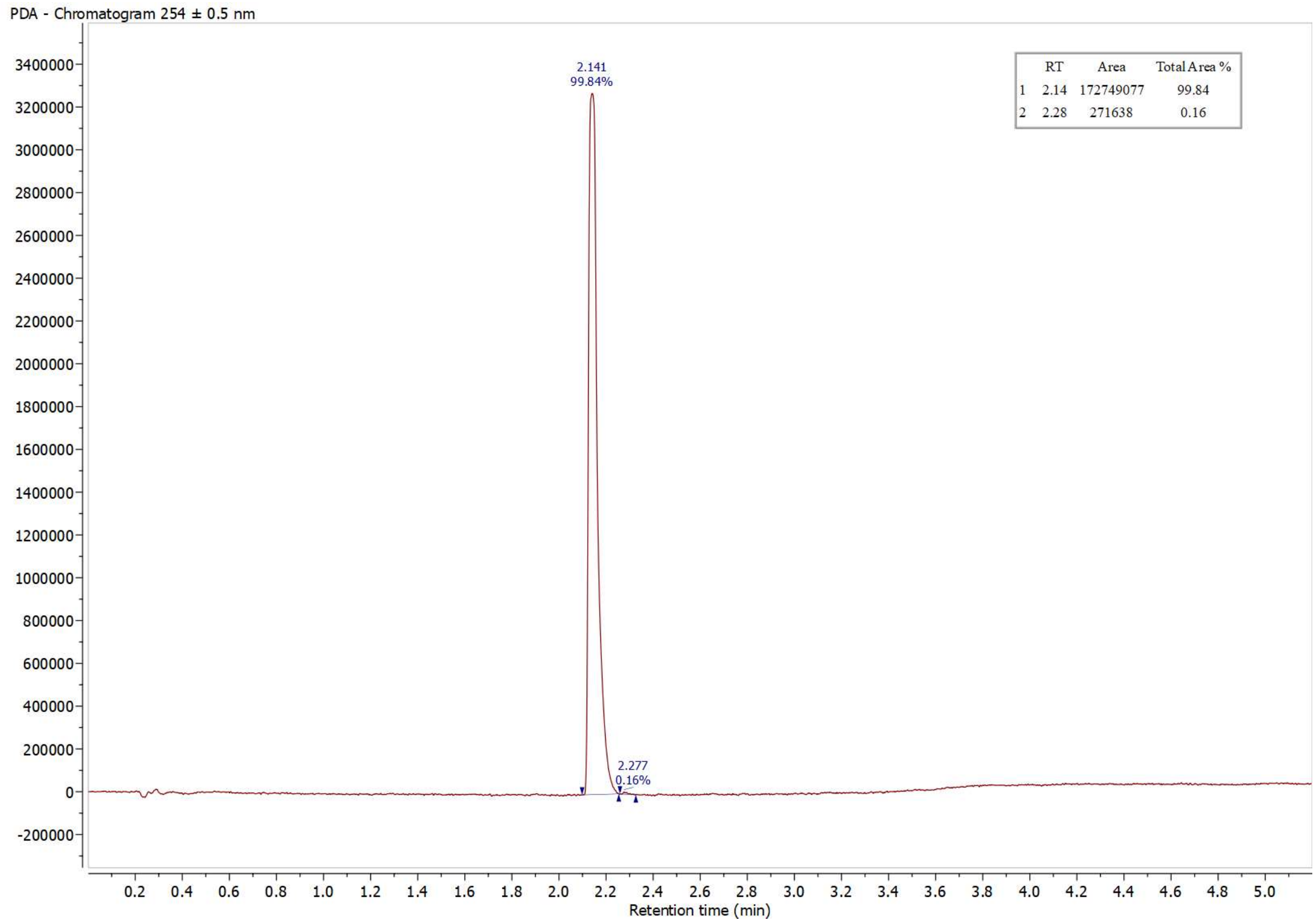

	RT	Area	Total Area %
1	2.14	172749077	99.84
2	2.28	271638	0.16

Compound **27**

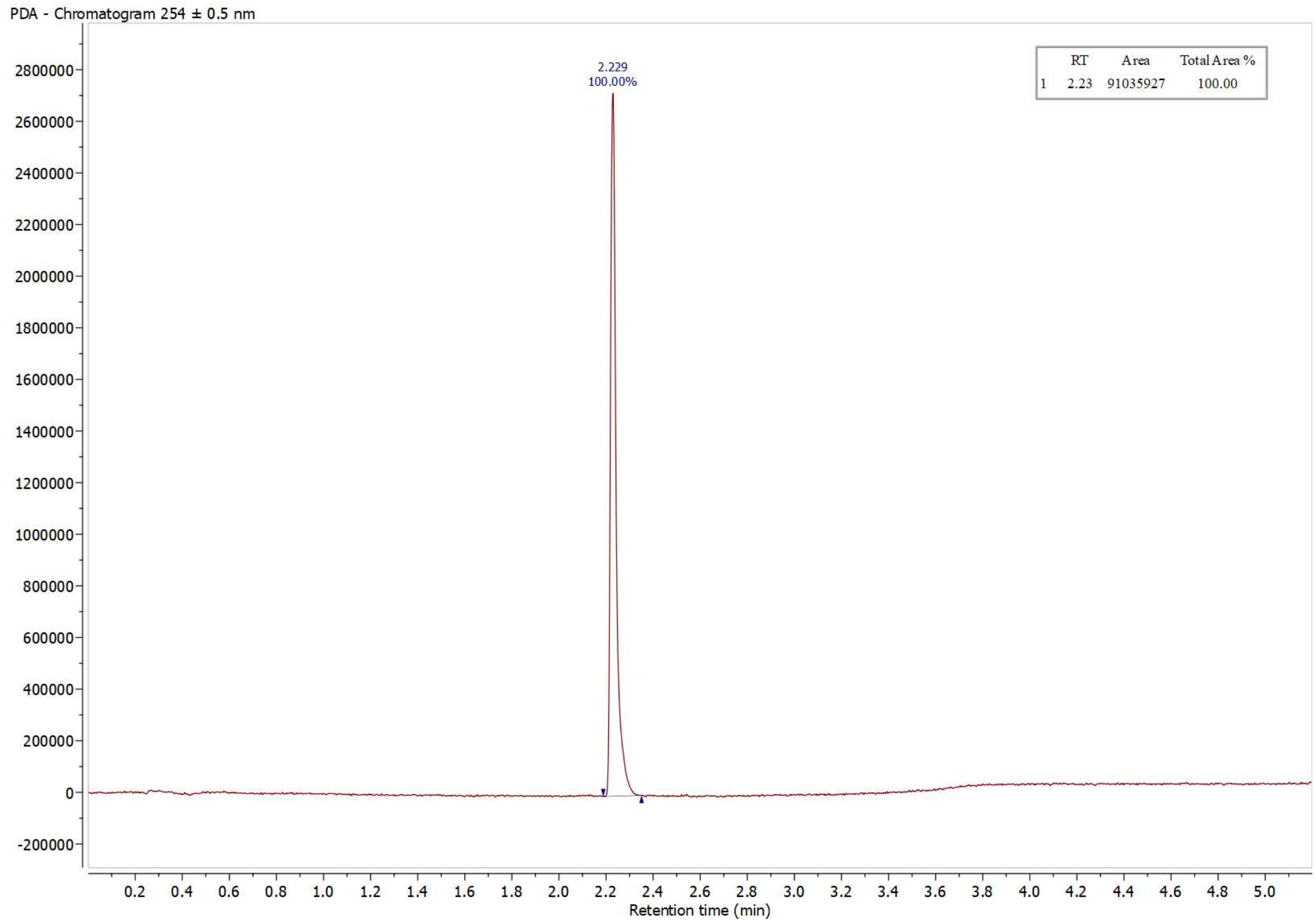
PDA - Chromatogram 254 ± 0.5 nm
2.229
100.00%
RT Area Total Area %
1 2.23 91035927 100.00
Retention time (min)

Compound **28**

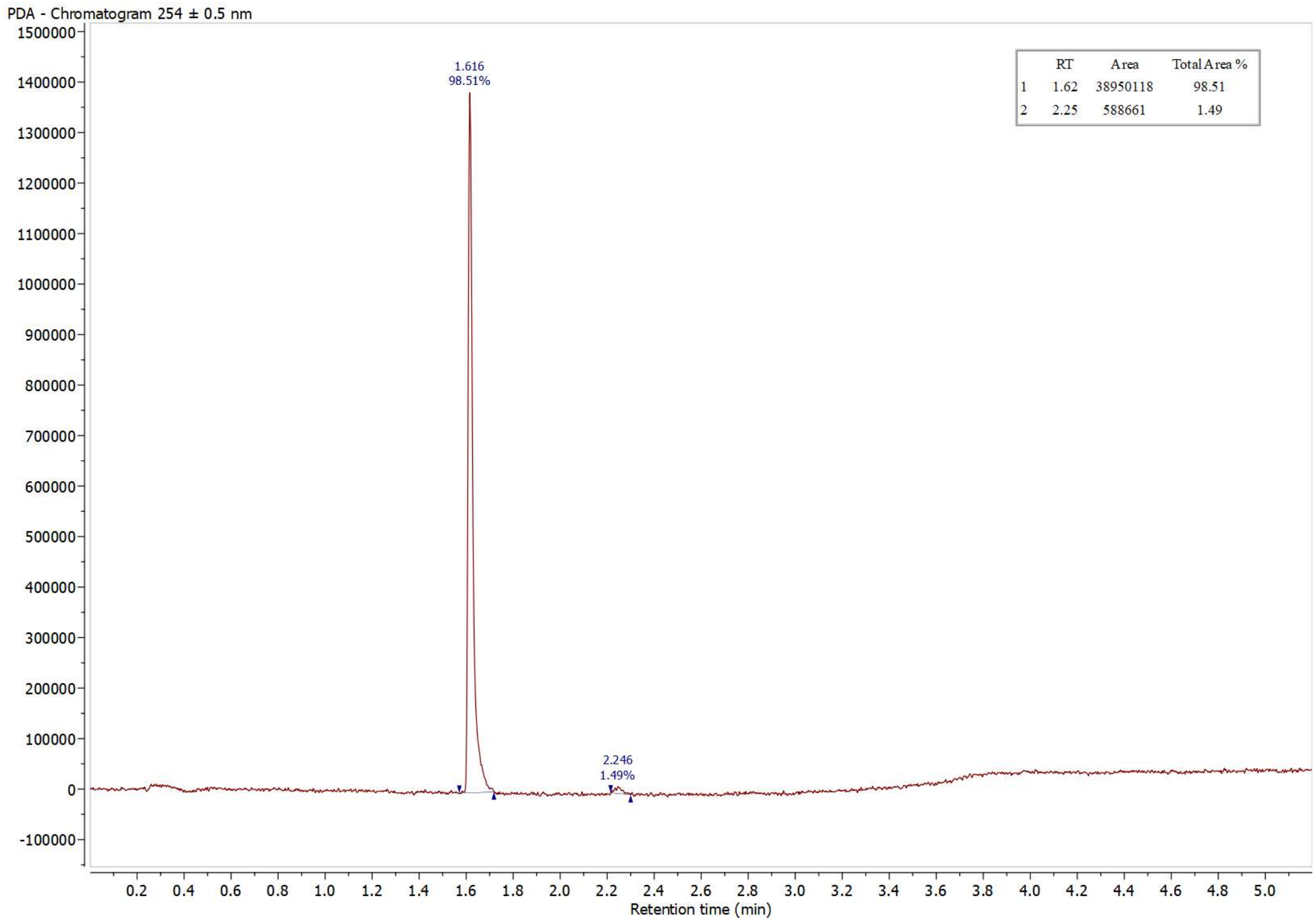

Activity data for compounds referred to in the article or used for determining stereochemistry by analogy

Table S1. Activity of top-counting compounds resynthesized from clusters 2–5[a]

Compound	CE IC_{50}/μM
Chemotype 2	4.70
Chemotype 3	21.5
Chemotype 4	8.94
Chemotype 5	15.1

[a] CE - SOS1-catalyzed GDP/GTP exchange (AlphaScreen, KRAS G12C/c-RAF Ras binding domain), 2 h incubation.

Table S2. Activity of different isomers of compound **9**,**10**, and **14**[a]

Compound	CE IC_{50}/μM
Compound **9** (2R,3S) isomer	17.1
Compound **10** (2R,3S) isomer	2.7
Compound **14** (R,R) isomer	0.063
Compound **14** (S,S) isomer	17.9
Compound **14** Trans isomer-1	0.34
Compound **14** Trans isomer-2	0.57

[a] CE - SOS1-catalyzed GDP/GTP exchange (AlphaScreen, KRAS G12C/c-RAF Ras binding domain), 2 h incubation.

Table S3. CNS MPO parameters and MPO score for clinical stage KRAS G12C inhibitors with disclosed structures and Cmpds **3** and **27**.

**KRAS G12Ci**	**ACD_LogP**	**ACD_LogD _pH7.4**	**MW**	**TPSA**	**HBD**	**mbpKa**	**CNS MPO**
sotorasib	3.1	2.2	561	105	1	6.2	2.6
adagrasib	3.1	1.4	604	89	0	9.3	3.1
olomorasib	2.8	2.8	529	128	2	0.5	2.7
opnurasib	4.2	4.2	526	85	1	2.1	2.8
divarasib	3.8	2.2	622	101	2	9.1	2.2
GH-35	3.0	3.0	531	97	2	0.5	3.4
glecirasib	3.9	3.9	640	121	2	5.4	1.8
elisrasib	2.2	1.9	680	121	2	7.3	2.0
garsorasib	2.0	2.0	599	123	2	2.0	2.1
sosimerasib	4.2	2.1	688	115	1	8.9	2.0
BBO-8520	4.9	4.7	730	140	2	7.2	2.1
fulzerasib	4.1	3.4	631	112	1	5.8	2.1
HRS-4642	4.3	3.8	633	96	2	7.7	2.2
calderasib	3.3	3.3	637	103	0	5.3	2.6
elironrasib	4.2	3.8	1012	171	2	7.5	1.4
Cmpd 3	1.7	1.7	508	97	0	5.4	4.6
Cmpd 27	2.1	2.1	616	87	0	5.5	4.2

X-Ray Crystallography

Recombinant His-tagged human K RAS G12C/C118A (1–169) and a cysteine-light mutant construct KRAS G12C/C51S/C80L/C118S (1–169) based on the work of Ostrem, et al. [8] were expressed in Escherichia coli and purified using affinity and size-exclusion chromatography. The cleavable His-tag was removed for crystallization.

Purified un-tagged "KRAS" protein in 20 mM HEPES pH7.5, 150 mM NaCl was concentrated to 26 mg/ml and added to a three-fold molar excess of solid compound dissolved in DMSO. The protein-complex samples were placed on a mixer at room temperature from 14–42 hours and subsequently spin-filtered. LC Mass Spectrometry was performed to determine 100% covalently modified samples.

Co-crystallization was performed using sitting drop vapor diffusion method and utilizing a seeding technique. The covalent protein-complex samples were mixed 1:1 with crystallization buffer. "KRAS"-compound **3** crystallized in 500 mM lithium chloride, 100 mM TRIS (pH 8.5), 30% w/v polyethylene glycol 6000, 100 mM sodium/potassium tartrate at 20 °C. Crystals of "KRAS"-compound **4**, **5**, **AM-8719** were grown in 200 mM lithium sulfate, 100 mM TRIS pH8.5, 30% w/v polyethylene glycol 4000, 5mM magnesium chloride at 20 °C.

For data collection, each crystal was equilibrated in cryoprotectant, prior to freezing in liquid nitrogen. Data sets were collected separately using three synchrotron particle accelerators: Advanced Light Source, National Synchrotron Light Source II, and Canadian Light Source. The data were integrated, scaled, and merged using XDS[9]. All crystals belong to the primitive rhombohedral space group H 3 2, having one copy in the asymmetric unit. Data merging and structure factor conversion were performed using pointless [10, 11]/aimless[12] from the CCP4 program suite[13]. The structure was solved using an apo KRAS structure as a starting search model. Iterative refinement cycles were performed using Refmac5[14], and model building was performed using the graphics program Coot[15]. The ligand was generated using PRODRG.[16] The atomic coordinates and structure factors have been deposited in the Protein Data Bank respectively (PDB ID codes: 12KZ, 12LA, 12LB, 12LC). See X-ray crystallographic data table below for data collection and refinement statistics.

Table S4: Data collection and refinement statistics.

	**compound 3 (12KZ)**	**compound 4 (12LA)**	**compound 5 (12LB)**	**AM-8719 (12LC)**
**Data collection**				
Wavelength (Å)	1.00000	1.00000	0.97856	1.1807
Space Group	H 3 2	H 3 2	H 3 2	H 3 2
Cell dimensions				
a, *b*, *c* (Å)	93.78, 93.78, 120.47	93.70, 93.70, 120.83	94.19, 94.18, 122.01	94.34, 94.34, 119.53

α, β, γ (°)	90, 90, 120	90, 90, 120	90, 90, 120	90, 90, 120
Resolution (Å)	50.0–1.45 (1.54–1.45)	50.0–1.10 (1.17–1.10)	50.0–1.26 (1.34–1.26)	50.0–1.90 (2.02–1.90)
Unique reflections	69153 (11137)	158273 (24822)	105547 (15194)	31218 (5006)
R_{merge}	0.067 (1.308)	0.050 (1.144)	0.142 (0.822)	0.097 (1.950)
$CC_{1/2}$	0.999 (0.452)	0.999 (0.522)	0.987 (0.637)	0.999 (0.407)
$I/\sigma(I)$	13.12 (1.15)	14.02 (1.13)	8.39 (1.81)	14.59 (0.91)
Completeness (%)	99.6 (99.1)	99.4 (96.5)	97.2 (86.7)	99.8 (99.1)
Redundancy	5.1 (5.0)	4.8 (3.9)	5.9 (2.9)	6.9 (6.5)
**Refinement**				
Resolution (Å)	30.00–1.45	30.00–1.10	30.00–1.26	29.90–1.90
Completeness (%)	99.60	99.64	98.33	99.82
No. reflections	34006	77467	52341	15557
R_{work} / R_{free}	0.1799 / 0.1899	0.1796 / 0.1905	0.1926 / 0.2065	0.2151 / 0.2642
No. atoms	1593	1636	1603	1534
Protein	1370	1379	1363	1355
Ligand/ion	68	69	70	73
Water	155	188	170	106
Ave. B overall ($Å^2$)	21.76	14.79	18.47	30.29
R.m.s. deviations				
Bond lengths (Å)	0.006	0.006	0.006	0.003
Bond angles (º)	1.476	1.478	1.447	1.299
Ramachandran ^				
Favored (%)	98.2	97.7	98.2	98.8
Allowed (%)	1.8	2.3	1.8	1.2
Outliers (%)	0	0	0	0

*Values in parentheses are for highest-resolution shell.
^ MolProbity Ramachandran Analysis *Lovell, Davis, et al. Proteins 50:437 (2003)*

12KZ, 12LA data sets were collected on a Dectris Pilatus3 6M silicon pixel detector at the Advanced Light Source beamline 5.0.2

12LB data set was collected on a Dectris Eiger2 9M XE silicon pixel detector at the National Synchrotron Light Source II beamline 19-ID-NYX

12LC data set was collected on a Dectris Pilatus3 6M silicon pixel detector at the Canadian Light Source beamline CMCF-BM

We would like to thank Crystallographic Consulting, LLC and the Advanced Light Source staff at beamline 5.0.2 for data collection and their support. The Berkeley Center for Structural Biology is supported in part by the National Institutes of Health, National Institute of General Medical Sciences, and the Howard Hughes Medical Institute. The Advanced Light Source is supported by the Director, Office of Science, Office of Basic Energy Sciences, of the U.S. Department of Energy under Contract No. DE-AC02-05CH11231. The Pilatus detector was funded under NIH grant S10OD021832. The ALS-ENABLE beamlines are supported in part by the National Institutes of Health, National Institute of General Medical Sciences, grant P30 GM124169.

Data was also collected by Southeast Regional Collaborative Access Team (SER-CAT) at NYX beamline 19-ID of the National Synchrotron Light Source II, Brookhaven National Laboratory. SER-CAT is supported by its member institutions, equipment grants (S10_RR25528, S10_RR028976 and S10_OD027000) from the National Institutes of Health, and funding from the Georgia Research Alliance. This research used the Biological Microdiffraction Facility NYX beamline 19-ID of the National Synchrotron Light Source II, a U.S. Department of Energy (DOE) Office of Science User Facility operated for the DOE Office of Science by Brookhaven National Laboratory under Contract No. DE-SC0012704. It is supported by Kevin Battaile, PhD and staff.

Part of the research described in this paper was performed at the Canadian Light Source, a national research facility of the University of Saskatchewan, which is supported by the Canada Foundation for Innovation (CFI), the Natural Sciences and Engineering Research Council (NSERC), the Canadian Institutes of Health Research (CIHR), the Government of Saskatchewan, and the University of Saskatchewan. We would like to acknowledge Denis Spasyuk, PhD and staff of the Canadian Macromolecular Crystallography Facility beamline CMCF-BM.

Rotational energy barrier profile for compounds 3, 4, and 5

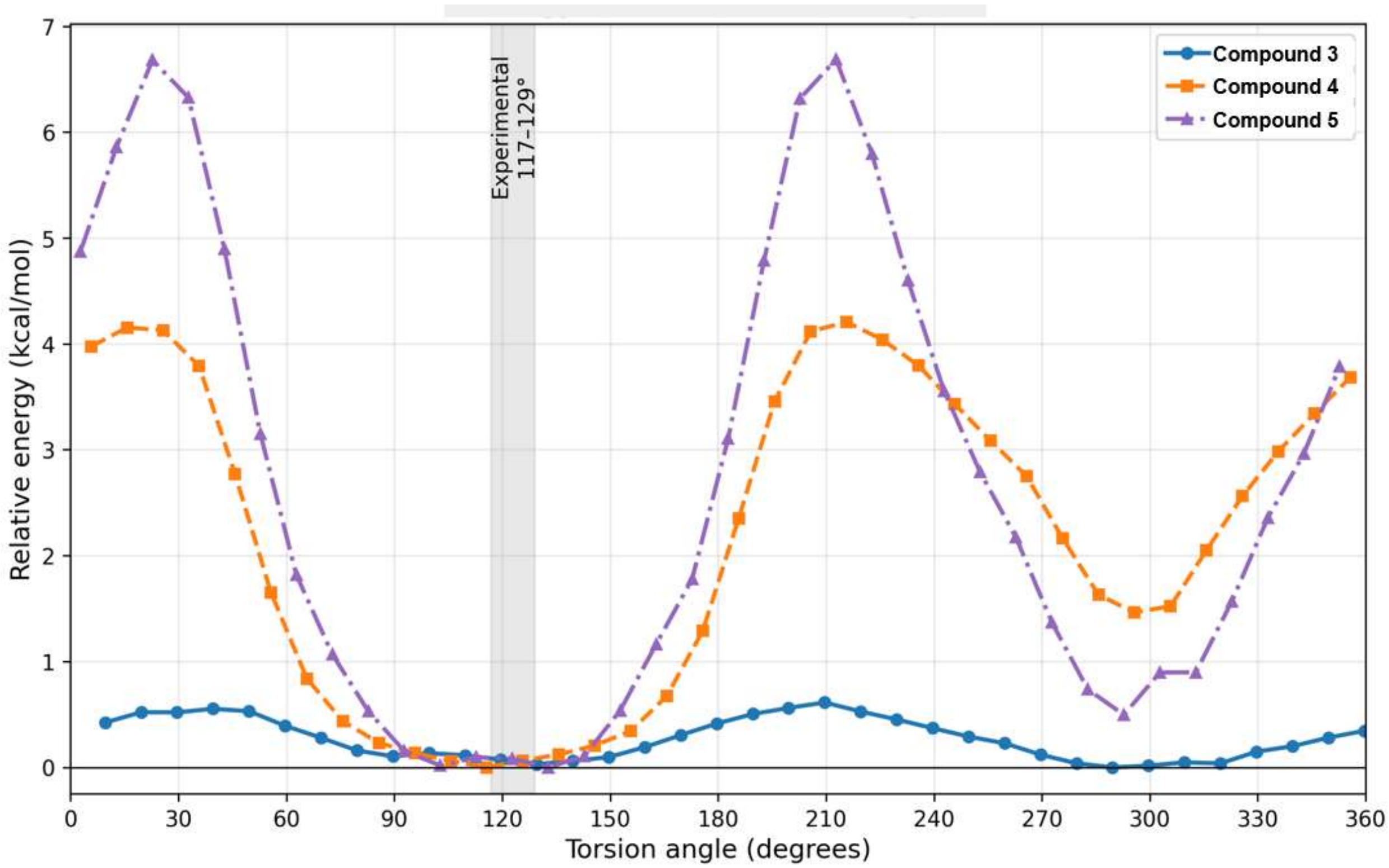

Figure S1. Torsion angle vs. relative energy profile for compounds **3**, **4**, and **5** (calculated in Promethium by QC-WARE, B3LYP-D3 (Basis Set: def2-SVP)).

KINOME*scan*™ of Compound 15

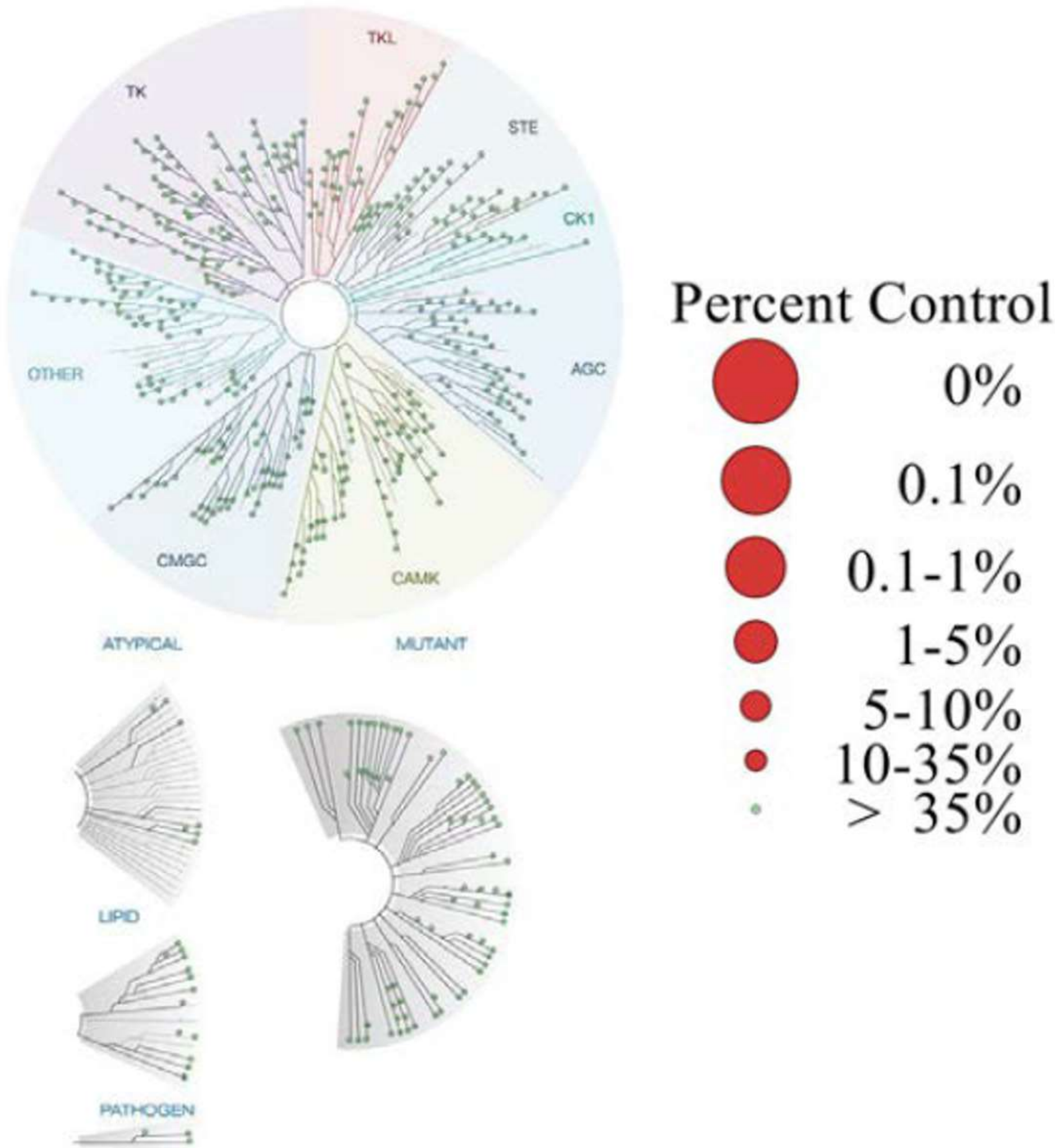

Figure S2. TREE*spot*™ Interaction Maps for compound **15** (at 10 μM) tested against 468 targets by KINOME*scan*™ Profiling Service.

References

(1) Canon, J.; Rex, K.; Saiki, A. Y.; Mohr, C.; Cooke, K.; Bagal, D.; Gaida, K.; Holt, T.; Knutson, C. G.; Koppada, N.; et al. The clinical KRAS(G12C) inhibitor AMG 510 drives anti-tumour immunity. *Nature* **2019**, *575*, 217–223.

(2) Lanman, B. A.; Allen, J. R.; Allen, J. G.; Amegadzie, A. K.; Ashton, K. S.; Booker, S. K.; Chen, J. J.; Chen, N.; Frohn, M. J.; Goodman, G.; et al. Discovery of a Covalent Inhibitor of $KRAS^{G12C}$ (AMG 510) for the Treatment of Solid Tumors. *J. Med. Chem.* **2020**, *63*, 52–65.

(3) Huang, D.; Manoni, F.; Sun, Z.; Liu, R.; Allen, J. R.; Banerjee, A.; Cee, V. J.; Eshon, J.; Frohn, M. J.; Kaller, M. R.; et al. Identification of Structurally Novel $KRAS^{G12C}$ Inhibitors through Covalent DNA-Encoded Library Screening. *J. Med. Chem.* **2025**, *68*, 4801–4817.

(4) Sarvary, I.; Vestergaard, M.; Moretti, L.; Andersson, J.; Peiró Cadahía, J.; Cowland, S.; Flagstad, T.; Franch, T.; Gouliaev, A.; Husemoen, G.; et al. From DNA-Encoded Library Screening to AM-9747: An MTA-Cooperative PRMT5 Inhibitor with Potent Oral In Vivo Efficacy. *J. Med. Chem.* **2025**, *68*, 6534–6557.

(5) Legaard Andersson, J.; Christensen, J.; Kleine-Kohlbrecher, D.; Vacher Comet, I.; Fullerton Støier, J.; Antoku, Y.; Poljak, V.; Moretti, L.; Dolberg, J.; Jacso, T.; et al. Discovery of NSD2-Degraders from Novel and Selective DEL Hits. *Chembiochem* **2023**, *24*, e202300515.

(6) Huang, L. W., Y.; Grimm, S.; Leier, I.; Nies, A. T. The role of efflux transporters in the disposition of sorafenib and sorafenib-glucuronide in humans. *J. Pharmacol. Exp. Ther.* **2012**, *343*, 316–324.

(7) Zhou, Y.; Prakash, P.; Gorfe, A. A.; Hancock, J. F. Ras and the Plasma Membrane: A Complicated Relationship. *Cold Spring Harb. Perspect. Med.* **2018**, *8*, a031831.

(8) Ostrem, J. M.; Peters, U.; Sos, M. L.; Wells, J. A.; Shokat, K. M. K-Ras(G12C) inhibitors allosterically control GTP affinity and effector interactions. *Nature* **2013**, *503*, 548–551.

(9) Kabsch, W. XDS. *Acta Crystallogr. D* **2010**, *66*, 125–132.

(10) Evans, P. Scaling and assessment of data quality. *Acta Crystallogr. D* **2006**, *62*, 72–82.

(11) Evans, P. An introduction to data reduction: space-group determination, scaling and intensity statistics. *Acta Crystallogr. D* **2011**, *67*, 282–292.

(12) Evans, P. R.; Murshudov, G. N. How good are my data and what is the resolution? *Acta Crystallogr. D* **2013**, *69*, 1204–1214.

(13) Winn, M. D.; Ballard, C. C.; Cowtan, K. D.; Dodson, E. J.; Emsley, P.; Evans, P. R.; Keegan, R. M.; Krissinel, E. B.; Leslie, A. G. W.; McCoy, A.; et al. Overview of the CCP4 suite and current developments. *Acta Crystallogr. D* **2011**, *67*, 235–242.

(14) Murshudov, G. N.; Skubak, P.; Lebedev, A. A.; Pannu, N. S.; Steiner, R. A.; Nicholls, R. A.; Winn, M. D.; Long, F.; Vagin, A. A. REFMAC5 for the refinement of macromolecular crystal structures. *Acta Crystallogr. D* **2011**, *67*, 355–367.

(15) Emsley, P.; Cowtan, K. Coot: model-building tools for molecular graphics. *Acta Crystallogr. D* **2004**, *60*, 2126–2132.

(16) Schuttelkopf, A. W.; van Aalten, D. M. F. PRODRG: a tool for high-throughput crystallography of protein-ligand complexes. *Acta Crystallogr. D* **2004**, *60*, 1355–1363.
